# Access to P-chiral phosphine oxides by enantioselective allylic alkylation of bisphenols†
†Electronic supplementary information (ESI) available: Experimental procedures, characterization data and computational details. CCDC 1579041. For ESI and crystallographic data in CIF or other electronic format see DOI: 10.1039/c8sc05439h


**DOI:** 10.1039/c8sc05439h

**Published:** 2019-03-12

**Authors:** Guo-Hui Yang, Yao Li, Xin Li, Jin-Pei Cheng

**Affiliations:** a State Key Laboratory of Elemento-Organic Chemistry , College of Chemistry , Nankai University , Tianjin 300071 , China . Email: xin_li@nankai.edu.cn

## Abstract

A novel biscinchona alkaloid-catalyzed highly enantioselective desymmetrization reaction of bisphenol compounds with achiral Morita–Baylis–Hillman carbonate agents was developed. Through the asymmetric allylic alkylation strategy, a broad range of optically active P-stereogenic phosphine oxides were generated with excellent to good yields (up to 99%) and high enantioselectivities (up to 98.5 : 1.5 e.r.). The reaction was further investigated by the linear free energy relationship (LFER) analysis. A possible transition state was proposed and furthered verified by theoretical calculations.

## Abstract 2

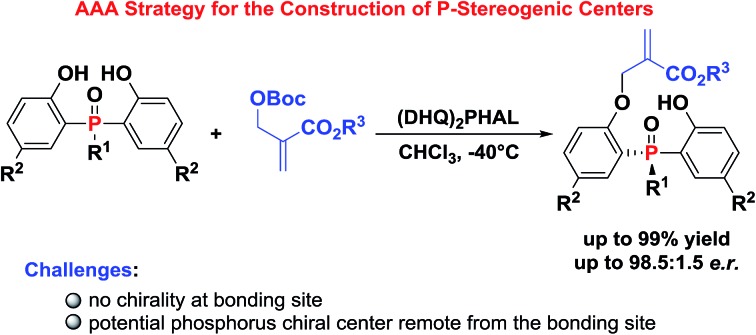
A biscinchona alkaloid-catalyzed AAA reaction for the construction of P-stereogenic center compounds was developed.

## Introduction

**Scheme 1:**
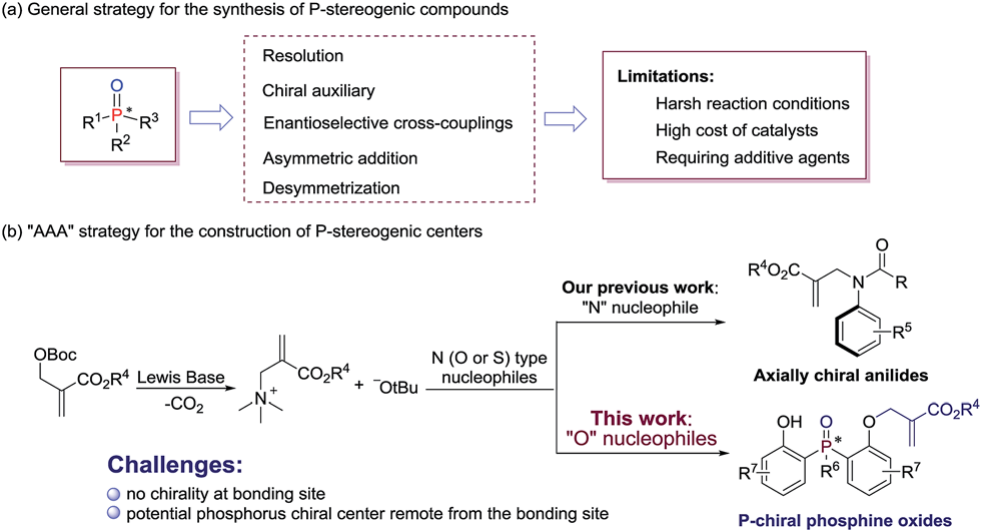
(a) Methods for the synthesis of P-stereogenic compounds and (b) our design.

P-stereogenic compounds, in which the chirality is on the phosphorus atom, have been widely used as biologically active compounds,^1^ chiral ligands^2^ and useful building blocks.^3^ The significance of this privileged structural motif with P-stereogenic centers has led to a great demand for efficient synthetic methods. However, the methodologies used to synthesize such chiral structures were largely limited.^4^ Early reports on P-stereogenic center synthesis comprise the resolution of diastereoisomers,^5^ chiral auxiliary controlled asymmetric reactions,^6^ transition-metal-catalyzed enantioselective cross-coupling,^7^ asymmetric addition reactions of phosphorus nucleophiles,^8^ and desymmetrization of prochiral phosphorus derivatives (Scheme 1a).^9^ Among the above mentioned synthetic strategies of P-stereogenic centers, only two cases were achieved through organocatalysis.^10^ Therefore, it is currently desirable and challenging to develop an organocatalysis-based synthetic methodology for P-stereogenic centers, and to expand the facile and extensive substrate adaptability.

Lewis base catalyzed asymmetric allylic alkylation (AAA) reaction with Morita–Baylis–Hillman (MBH) carbonates as electrophile precursors has been considered as one of the most attractive approaches to build stereogenic centers. However, the AAA strategy currently is restricted to the building of chiral carbon centers, because most frequently used allylation reagents are racemic MBH adducts.^11^ In fact, it is possible to expand the usage of the AAA strategy to building other non-carbon center chirality when the reactions proceed between achiral MBH adducts and N (O or S) type nucleophiles. Our group recently developed a general and efficient method for atroposelective construction of axially chiral anilides (Scheme 1b).^12^

In continuation of our ongoing interest in the AAA strategy, we speculated that the privileged phosphorus compounds with P-stereogenic centers could be generated *via* the AAA reaction between bis(2-hydroxyphenyl)phosphinates and achiral MBH carbonates (Scheme 1b). Thus, two challenges need to be solved in this scenario: (i) the difficulty in controlling stereoselectivities due to the lack of chirality at the bonding site; (ii) the difficulty in designing a proper chiral catalyst to induce enantiocontrol during the course of desymmetrization, because the targeted phosphorus center is remote from the enantiotopic site. Herein, we report a biscinchona alkaloid-catalyzed enantioselective desymmetrization of bis(2-hydroxyphenyl)phosphine oxides. Utilizing the AAA strategy based method, a number of phosphine oxides possessing chiral phosphorus(v) atoms were synthesized with good enantioselectivities.

## Results and discussion

### Reaction optimization

Our initial investigation was carried out with bis(2-hydroxyphenyl)phosphinate **1a** and Boc protected MBH product **2a** as the model substrates, 10 mol% of cinchona alkaloid **4a** as the catalyst in CH_2_Cl_2_ at room temperature. The reaction gave the desymmetrization product **3a** in 96% yield, albeit with a racemic result (Table 1, entry 1). To improve the enantioselectivity, we next evaluated different types of cinchona alkaloid catalysts. As shown in Table 1, the catalysts' backbone demonstrated remarkable impacts towards the outcome of the reaction (Table 1, entries 2–8), in which biscinchona alkaloid catalyst **4f** gave the best 66 : 34 e.r (Table 1, entry 6). To further improve the enantioselectivity, we then screened different bisphenol substrates. To our delight, substrates **1b–1d**, which had large sized ester groups, showed improved enantioselectivities (Table 1, entries 9–11). This result indicated that the enantioselectivity of the studied desymmetrization reaction may be closely related to the steric hindrance of the substituent linked to the pre-stereogenic P-center. This hypothesis was furthermore supported by the reaction with substrate phosphine oxide **1e**, in which the enantioselectivity was obtained as 84 : 16 e.r (Table 1, entry 12). A series of subsequent screenings (for example, temperature, solvent, and substrate ratio) showed enhancement of the enantioselectivity. Lowering the reaction temperature to –40 °C can further increase the e.r. value to 94 : 6, while increasing the catalyst loading and prolonging the reaction time were necessary to ensure the conversion of the reaction (Table 1, entry 13). The evaluation of the solvents showed that CHCl_3_ was the best reaction medium in terms of reactivity and enantioselectivity (Table 1, entries 13–17). Increasing amount of substrate **2a** obtained an improved yield of 84% with retention of 94.5 : 5.5 e.r (Table 1, entry 18).

**Table 1:** Reaction optimization^*a*^

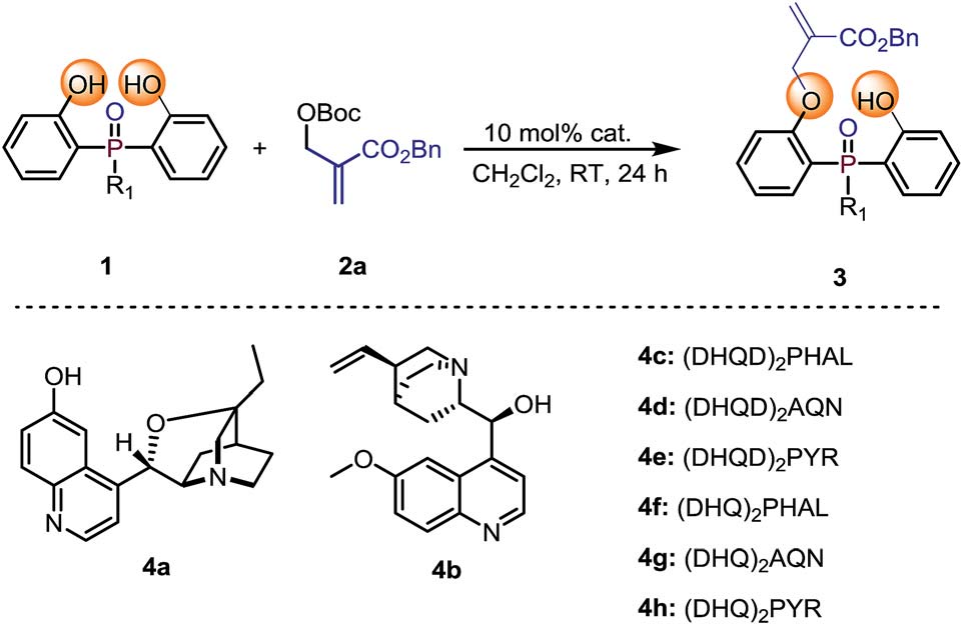					
Entry	*R*_1_	Cat.	Solvent	Yield^*b*^ (%)	e.r.^*c*^
1	**1a**: OMe	**4a**	CH_2_Cl_2_	**3a**: 96	50 : 50
2	**1a**: OMe	**4b**	CH_2_Cl_2_	**3a**: 99	54.5 : 45.5
3	**1a**: OMe	**4c**	CH_2_Cl_2_	**3a**: 60	51.5 : 48.5
4	**1a**: OMe	**4d**	CH_2_Cl_2_	**3a**: 60	52 : 48
5	**1a**: OMe	**4e**	CH_2_Cl_2_	**3a**: 73	53.5 : 46.5
6	**1a**: OMe	**4f**	CH_2_Cl_2_	**3a**: 73	66 : 34
7	**1a**: OMe	**4g**	CH_2_Cl_2_	**3a**: 68	53 : 47
8	**1a**: OMe	**4h**	CH_2_Cl_2_	**3a**: 68	53.5 : 46.5
9	**1b**: OEt	**4f**	CH_2_Cl_2_	**3b**: 65	68.5 : 31.5
10	**1c**: O*i*Pr	**4f**	CH_2_Cl_2_	**3c**: 64	70.5 : 31.5
11	**1d**: OAd	**4f**	CH_2_Cl_2_	**3d**: 90	71.5 : 28.5
12	**1e**: Ad	**4f**	CH_2_Cl_2_	**3e**: 90	84 : 16
13^*d*^	**1e**: Ad	**4f**	CH_2_Cl_2_	**3e**: 46	94 : 6
14^*d*^	**1e**: Ad	**4f**	CHCl_3_	**3e**: 60	94.5 : 5.5
15^*d*^	**1e**: Ad	**4f**	THF	**3e**: 36	94 : 6
16^*d*^	**1e**: Ad	**4f**	Toluene	Trace	Nd^*f*^
17^*d*^	**1e**: Ad	**4f**	Ether	Trace	Nd^*f*^
18^*d*^ ^,^^*e*^	**1e**: Ad	**4f**	CHCl_3_	**3e**: 84	94.5 : 5.5

^*a*^Reaction conditions: phosphinate **1** (0.1 mmol), MBH carbonate **2a** (0.12 mmol), catalyst (10 mol%), in 1 mL of solvent.

^*b*^Isolated yields.

^*c*^Determined by chiral HPLC analysis.

^*d*^The reaction was conducted at –40 °C with 20 mol% (DHQ)_2_PHAL and the reaction time was 6 days.

^*e*^With 0.2 mmol **2a**.

^*f*^Not determined.

### Substrate evaluation

With the optimal reaction conditions in hand, we set out to explore the substrate generality of this desymmetrization strategy. Firstly, different substituted phosphine oxides were investigated. As shown in Table 2, substrates with electron-donating groups and electron-withdrawing groups on the phenyl ring were well tolerated in this reaction. The corresponding products **3e–3k** and **3m–3o** were afforded in 34–91% yields and 91.0 : 9.0–98.5 : 1.5 e.r. values. When the substituted atom was F, the enantioselectivity decreased to a moderate level (**3l**, 65% yield and 88.5 : 11.5 e.r.). It is valuable to note that the steric hindrance of the substituent seems to also influence on the reactivity. For example, the substrate with R_2_ as the *tert*-butyl group only provided the corresponding product **3j** with 34% yield under the optimized reaction conditions. Two other substrates with large steric hindrance phosphine oxides, in which the groups linked to the P-center were *tert*-butyl and triphenyl methyl, were also examined. As a result, **3p** was obtained in 81% yield with 93 : 7 e.r. and **3q** was obtained in 81% yield with 88.5 : 11.5 e.r.

**Table 2:** Substrate scope of phosphine oxide substrates^*a*^

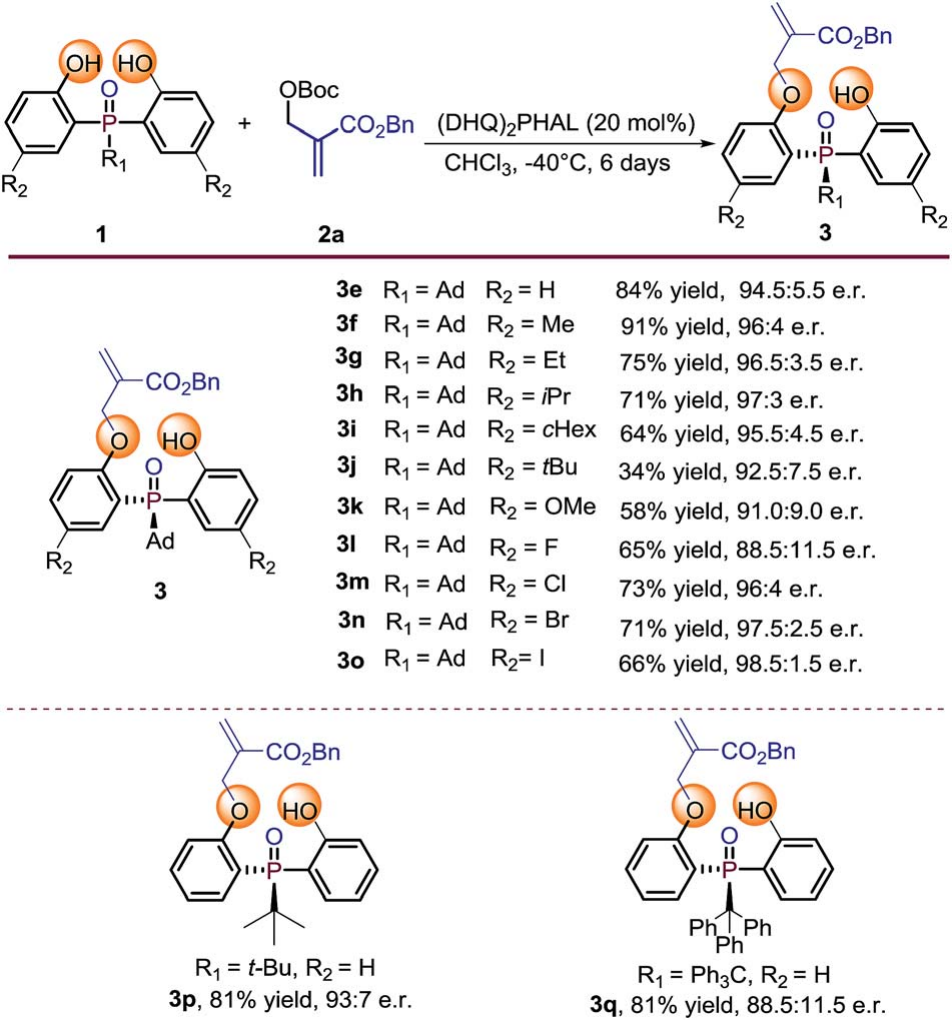

^*a*^Reaction conditions: phosphine oxides **1** (0.1 mmol), MBH carbonate **2a** (0.2 mmol), catalyst (20 mol%), 1 mL CHCl_3_. Isolated yields. e.r. values were determined by chiral HPLC analysis.

Further investigation of the substrate scope was focused on the Boc protected MBH carbonate substrates **2** (Table 3). The electronic nature or position of the substituent on the benzyl ring does not appear to affect the results, all the benzyl-substituted MBH carbonates afforded the target products **3t–3e′** in moderate to excellent yields (41–99%) with excellent enantioselectivities (95.5 : 4.5 to 97.5 : 2.5 e.r.). The 1-methylnaphthyl type MBH carbonate tolerated well under the optimal condition, gave **3s** in 93% yield with 95.5 : 4.5 e.r. The *tert*-butyl substituted MBH carbonate is also transformed to the desired product **3r** in 61% yield and 92 : 8 e.r. by running the reaction at 0 °C albeit with large steric hindrance. The absolute configuration of **3r** was determined by X-ray diffraction analysis and those of other products were assigned by analogy.^13^

**Table 3:** Substrate scope of MBH carbonate substrates^*a*^

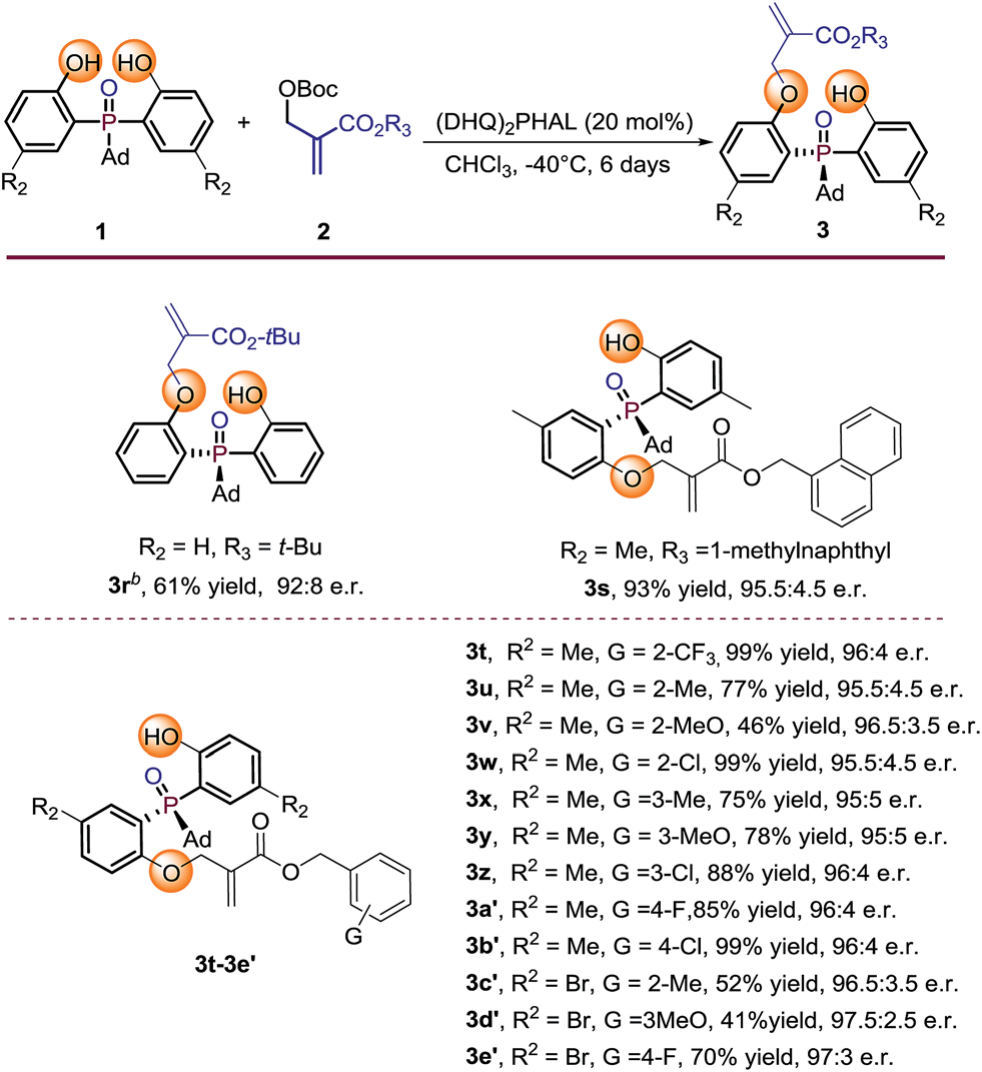

^*a*^Reaction conditions: phosphine oxides **1** (0.1 mmol), MBH carbonates **2** (0.2 mmol), catalyst (20 mol%), 1 mL CHCl_3_. Isolated yields. e.r. values were determined by chiral HPLC analysis.

^*b*^The reaction was conducted at 0 °C.

### LFER research studies

The substrate scope study reveals that, in general, products with large sized substituents on the aromatic ring of phosphine have better enantioselectivity. To investigate the effect of the steric factor on the enantioselectivity, we plotted the enantioselectivities against Charton values.^14^ Preliminary analysis revealed that substrates having large steric substituents R_2_ gave better enantioselectivities (For R_2_ = H, Me, Et, *i*Pr, see Fig. S1 in the ESI†). This rule could also be expanded to substrates containing halogen substituents (R_2_ = F, Cl, Br, I, see Fig. S2 in the ESI†). It seemed that the steric factor may play a role in enantioselectivity control. However, the steric correlation could not explain the stereoselectivity for the substrates containing other large hindrance substituents, such as R_2_ = *t*Bu or *c*Hex. Thus, other factors, such as the electronic effect, should also be considered.

**Fig. 1:**
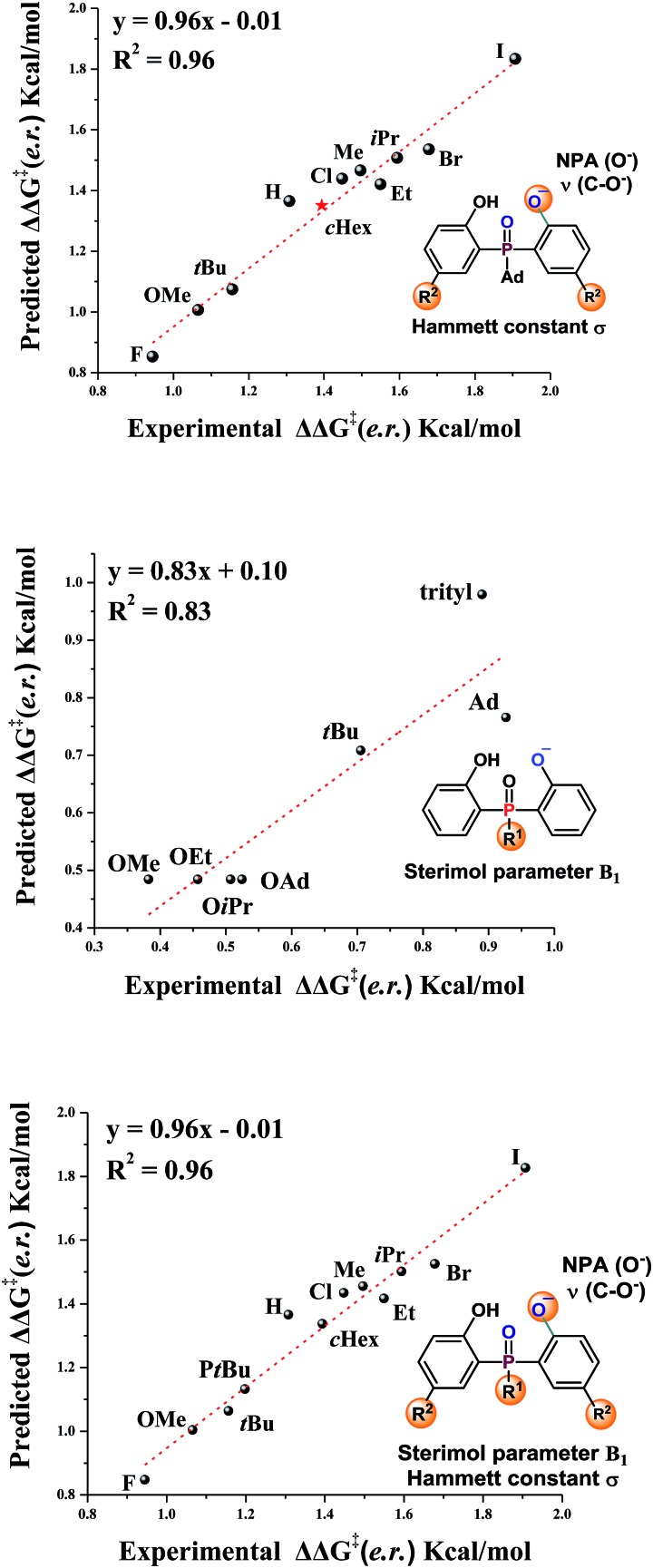
Correlation of enantioselectivity with substrate parameters.

Recently, Sigman and coworkers utilized multivariate linear regression (MLR) models to effectively predict the experimental reaction outcome based on both experimentally derived and calculated physical organic molecular descriptors.^15^ Inspired by their work and our previous related study,^12^ we explored the factors that govern the stereoselectivity. The regression analysis was made with ten data sets and tested with one data set. After evaluating various parameters, the Hammett constant (*σ*), NPA charge (O^–^) and *ν* (C–O^–^),15b,c which can be used to embody the steric and electronic effects, were found to accurately predict the stereoselectivity (eqn (1), Fig. 1, slope = 0.96, intercept = –0.01, *R*^2^ = 0.96). Besides, the effects of substituents linked to the pre-stereogenic P-center were evaluated. The stereoselectivity is in good correlation with Sterimol parameter *B*_1_ (eqn (2)).^16^ The steric factor of substituents linked to the phosphorus atom significantly affected the enantioselectivity. Finally, based on eqn (2), eqn (1) could be expanded to substrates that have different substituents linked to the pre-stereogenic P-center (eqn (3), slope = 0.96, intercept = –0.01, *R*^2^ = 0.96).

1 ΔΔ*G*^‡^ (e.r.) = –3.04*σ* + 78.6NPA_(O^–^)_ – 0.014*ν*_(C–O^–^)_ + 88.2

2 ΔΔ*G*^‡^ (e.r.) = 0.16*B*_1_ + 0.24

3 ΔΔ*G*^‡^ (e.r.) = –3.03*σ* + 78.1NPA_(O^–^)_ – 0.0144*ν*_(C–O^–^)_ + 0.828*B*_1_ + 85.7

### Theoretical calculations

**Fig. 2:**
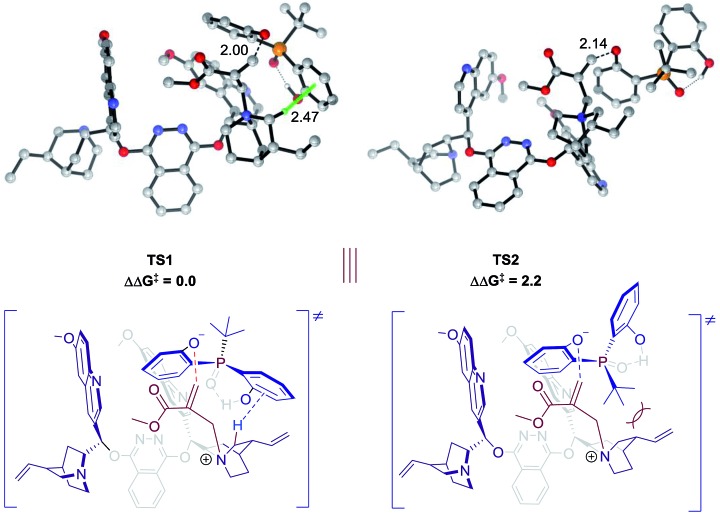
Transition state structures and relative free energies (in kcal mol^–1^) for desymmetrization catalyzed by **4f**. Noncritical hydrogen atoms have been omitted for clarity. The C–H···π interaction in TS1 is highlighted.

Theoretical calculations were conducted to support the above analysis.^17^,^18^ Extensive explorations of a variety of catalytic arrangements show that the two most stable transition state structures are TS1 and TS2 (Fig. 2). The free energy difference between TS1 and TS2 is 2.2 kcal mol^–1^, which agrees with experimental data, 85 : 15 e.r. As shown in Fig. 2, TS1 is stabilized by a C–H···π interaction between the methylene of the quinuclidine ring and the aromatic ring of phenol. However, this interaction is missing in TS2, which would be a key factor that contributes to the energy difference between TS1 and TS2. Previous studies of C–H···π interactions showed that the interaction energies correlated with Hammett constant (*σ*_m_),^19^ which supported our LFER analysis. The steric factor of substituents linked to the pre-stereogenic P-center could also be explained by transition states. If the *tert*-butyl group linked to the phosphorus atom is replaced by an adamantyl group, the steric effect between the catalyst and substrate will destabilize TS2, which is crucial for excellent enantioselectivity control.

### Large scale reaction and product transformation

**Scheme 2:**
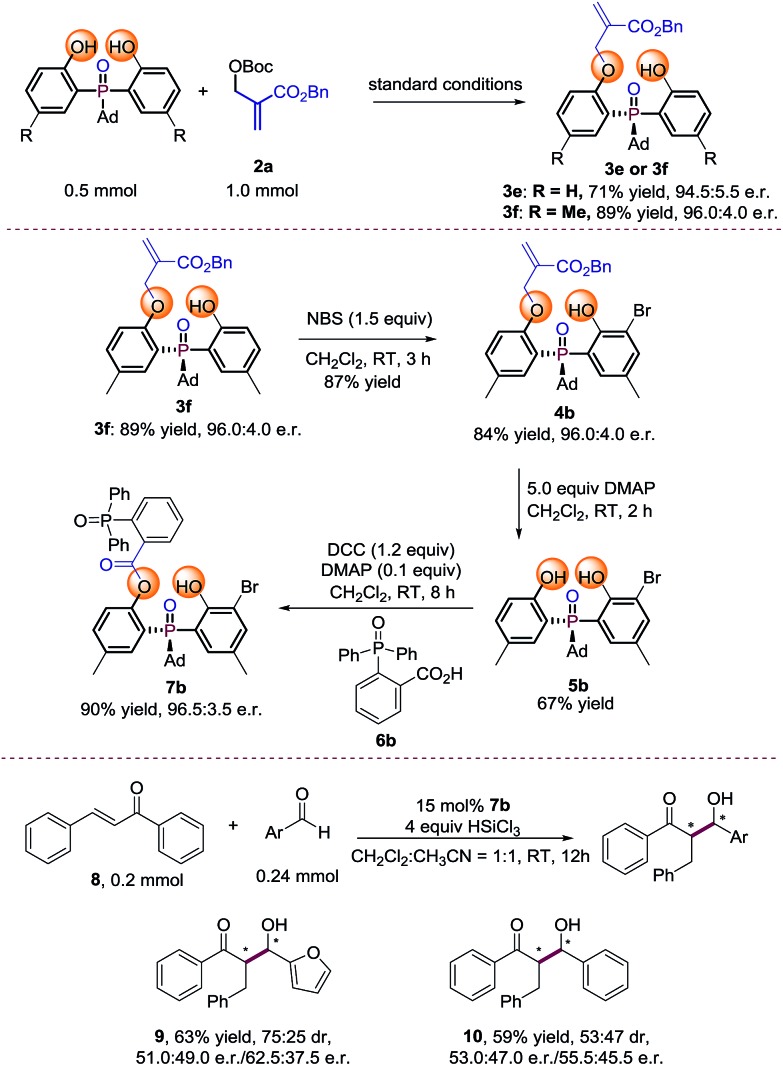
Large scale reaction and synthetic transformations of the product.^21^

To probe the efficiency of the current studied desymmetrization strategy in preparative synthesis, large scale reactions were investigated under the optimal conditions. To our delight, the desired products **3e** and **3f** were obtained without any loss of enantioselectivity (Scheme 2). Further bromination of **3f** by 1.5 equiv. NBS afforded **4b**. Treating **4b** with 5 equivalents of DMAP afforded **5b** in moderate yield with retention of enantioselectivity. Finally, product **7b** can be obtained in 90% yield with 96.5 : 3.5 e.r. under the conditions shown in Scheme 2. The preliminary application of the synthesized bidentate chiral phosphine oxide indicated that **7b** could be used as a catalyst in asymmetric reactions between enones and aldehydes (Scheme 2).^20^

**Scheme 3:**
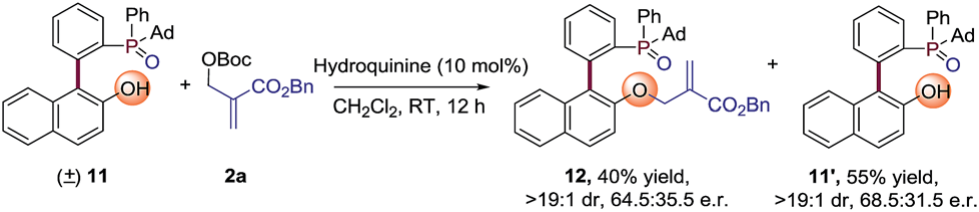
Kinetic resolution experiment using the AAA strategy.

Moreover, we also made another kinetic resolution experiment with substrate (±)-**11**, which contains both axial and phosphorus prochirality (Scheme 3). The reaction of (±)-**11** with **2a** proceeds smoothly in the presence of 10 mol% hydroquinine under the standard conditions, resulting in **12** in 40% yield with 64.5 : 35.5 e.r. and **11’** in 55% yield with 68.5 : 31.5 e.r. Further optimization of reaction conditions may lead to better enantioselectivities. This result again proves the universality of the strategy for the synthesis of P-stereogenic compounds.

## Conclusions

In summary, we have developed a catalytic enantioselective desymmetrization of bisphenols that hold pre-stereogenic P-centers, using a biscinchona alkaloid catalyst. This AAA strategy based method provides a novel and highly efficient way to the synthesis of P-stereogenic compounds, affording the desired functionalized phosphine oxides in good yields (up to 99%) and high enantioselectivities (up to 98.5 : 1.5 e.r.). A range of functional groups were tolerated under the mild reaction conditions. A possible transition state was proposed based on the linear free energy relationship analysis, which was further verified by theoretical calculations.

## Conflicts of interest

There are no conflicts to declare.

## Supplementary Material

Supplementary information

Crystal structure data
