# Supplementary material for: Access to P-chiral phosphine oxides by enantioselective allylic alkylation of bisphenols
Source: Chem Sci. 2019 Mar 12;10(15):4322–7. doi: 10.1039/c8sc05439h (PMC6499109; doi:10.1039/c8sc05439h)
Supplement: Supplementary file 1 [file SC-010-C8SC05439H-s001.pdf]

Electronic Supplementary Material (ESI) for Chemical Science.
This journal is © The Royal Society of Chemistry 2019

Electronic Supplementary Information

Access to P-Chiral Phosphine Oxides by Enantioselective Allylic Alkylation of Bisphenols

Guo Hui Yang, Yao Li, Xin Li* and Jin-Pei Cheng

Abstract: A novel biscinchona alkaloid-catalyzed highly enantioselective desymmetrization reaction of bisphenol compounds by using achiral Morita–Baylis–Hillman carbonate agents was developed. Through the asymmetric allylic alkylation strategy, a broad range of optically active P-stereogenic phosphine oxides were generated with good yields (up to 99%) and high enantioselectivities (up to 98.5:1.5 e.r.). The linear free energy relationship (LFER) analysis was introduced to investigate the reaction. And a possible transition state was proposed, which was verified by theoretical calculations.

DOI:

Contents

1. General procedure for the synthesis of substrates 3
2. Linear free energy relation (LFER) researches 6
3. Computation studies 43
4. Kinetic resolution research 56
5. Crystal structure data of **3r** 58
6. Characterization of products and novel substrates 59
   6.1 Characterization of products 59
   6.2 Characterization of novel substrates. 74
7. NMR and HPLC spectra 77
   NMR spectra 77
   HPLC spectra 131

General information

Commercially available materials purchased was used as received. ^{1}H NMR were recorded on a Bruker Avance (400 MHz) spectrometer, and reported as δ in units of parts per million (ppm) relative to tetramethylsilane (δ 0.00), and splitting patterns are designated as singlet (s), doublet (d), triplet (t), quartet (q), dd (doublet of doublets), m (multiplets). ^{13}C NMR were reported on a Bruker Avance (101 MHz) spectrometer, and reported as δ in units of parts per million (ppm) relative to the signal of chloroform-d (δ 77.16 triplet). ^{31}P NMR were reported on a Bruker Avance (162 MHz) spectrometer. ^{19}F NMR were reported on a Bruker Avance (376 MHz) spectrometer. Mass spectra were obtained using electrospray ionization (ESI) mass spectrometer. And the novel substrates and products were characterized in this ESI.

Experimental Section

1. General procedure for the synthesis of substrates

1.1 General procedure for the synthesis of substrates 1

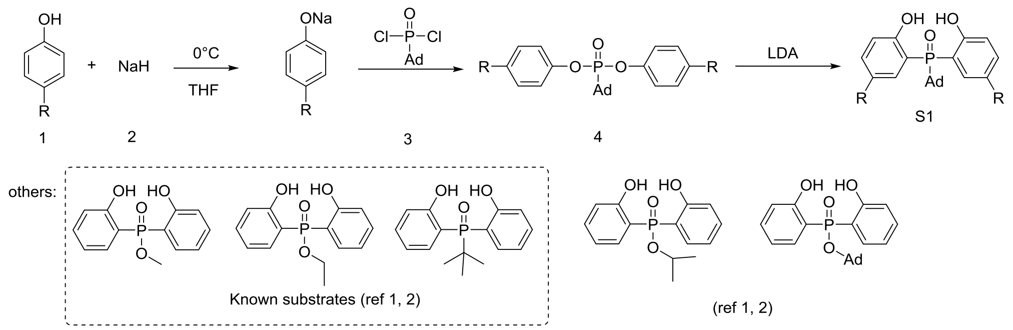

To a dry round bottomed flask equipped with a magnetic stir bar, added Phenols 1 (1 equiv) in THF, then 2 NaH (1.2 equiv) was added with nitrogen. The reaction was stirring at 0 °C for 30 minutes. When the reaction completed, 3 (0.5 equiv) was added to the mixture at 0 °C for 1h with nitrogen, and then 24h at room temperature. Extracted with $CHCl_3$ and the organic phase was dried over $MgSO_4$.The resulting crude residue was purified *via* column chromatography on silica gel to afford the desired products 4.

To a dry round bottomed flask equipped with a magnetic stir bar, added LDA (4 equiv) at -78 °C, 4 (1 equiv) dissolved in pure and dry THF was added in 60 min at -78 °C. The resulting reaction mixture was stirred at -78 °C for another 60 min, then it was allowed to warm up to rt and it was stirred at rt for 12 h. After the reaction was completed, quenched with saturated aqueous NH_4Cl solution, then extracted with $CHCl_3$. The organic phase was separated and the combined organic phase was dried over $MgSO_4$, filtered and the solvent was removed. The crude product was first purified by

chromatography on silica gel using an ethyl acetate/petroleum 1:10 to give pure S1.

And the novel substrates **S1** were were characterized in this ESI (Page 72-73).

1.2 General procedure for the synthesis of Boc protect MBH substrates 2.

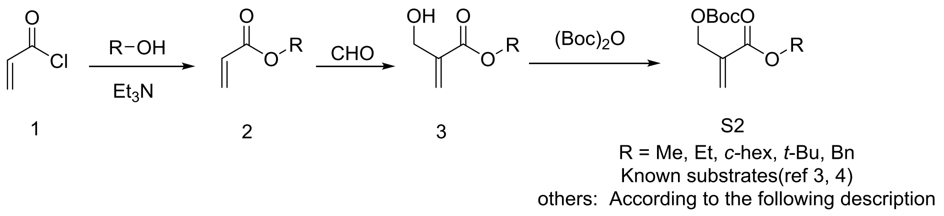

R-OH (1 equiv) and triethylamine (1.2 equiv) were dissolved in DCM in a flask and the flask was cooled down to 0 °C. Acrylyol chloride (1.2 equiv) was added slowly over 15 mins. The reaction was allowed to gradually warm to room temperature and stirred for another 2 hours. The reaction was quenched with aq. $NaHCO_3$ and the DCM phase was isolated and dried over anhydrous $MgSO_4$. The solvent was removed under reduced pressure and the resultant mixture was distilled under vacuum to give products 2.

And then a solution of formaldehyde solution (1 equiv) and products 2 (3 equiv) in 1,4-dioxane:water (1:1, v/v) were stirred at room temperature in the presence of 100 mol% DABCO, and the reaction progress was monitored by TLC, then extracted, the organic phase was washed with brine, dried over anhydrous $MgSO_4$, filtered, and concentrated under reduced pressure. The crude product was purified by flash column chromatography on silica to give the desired product

To an oven-dried round bottom flask equipped with a magnetic stir bar were added 3 (1 equiv) and ($(Boc)_2O$, 1 equiv), and DMAP (10 mol%), followed by DCM. The reaction mixture was stirred at room temperature for 2 hours, washed with water, dried with $MgSO_4$ and concentrated in vacuo. The crude products was purified by flash column chromatography to give the desired acrylic ester **S2**.

And the novel substrates **S2** were characterized in this ESI (Page 73-75).

1.3 General procedure for this reaction.

Example for the synthesis of 3e:

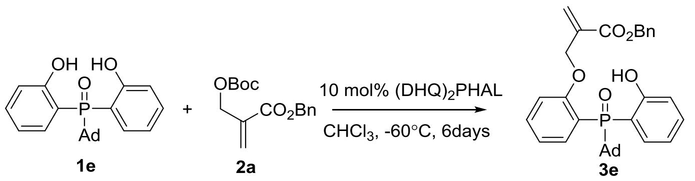

The compound **1e** (0.1 mmol, 36.8 mg), **2a** (0.2 mmol, 2.0 equiv, 58.4 mg) and catalyst **4f** $\mathbf{(DHQ)_2PHAL}$ (20 mol%, 15.6mg) were added to a 10 mL glass reactor, and then 1 mL $CHCl_3$ was added. The reaction was stirred at -40°C for 6 days. The mixture was chromatographed on a silica gel column eluted with PE:EA = 5:1 to afford the desired product **3e**.

1.3 General procedure for the derivatization experiments.

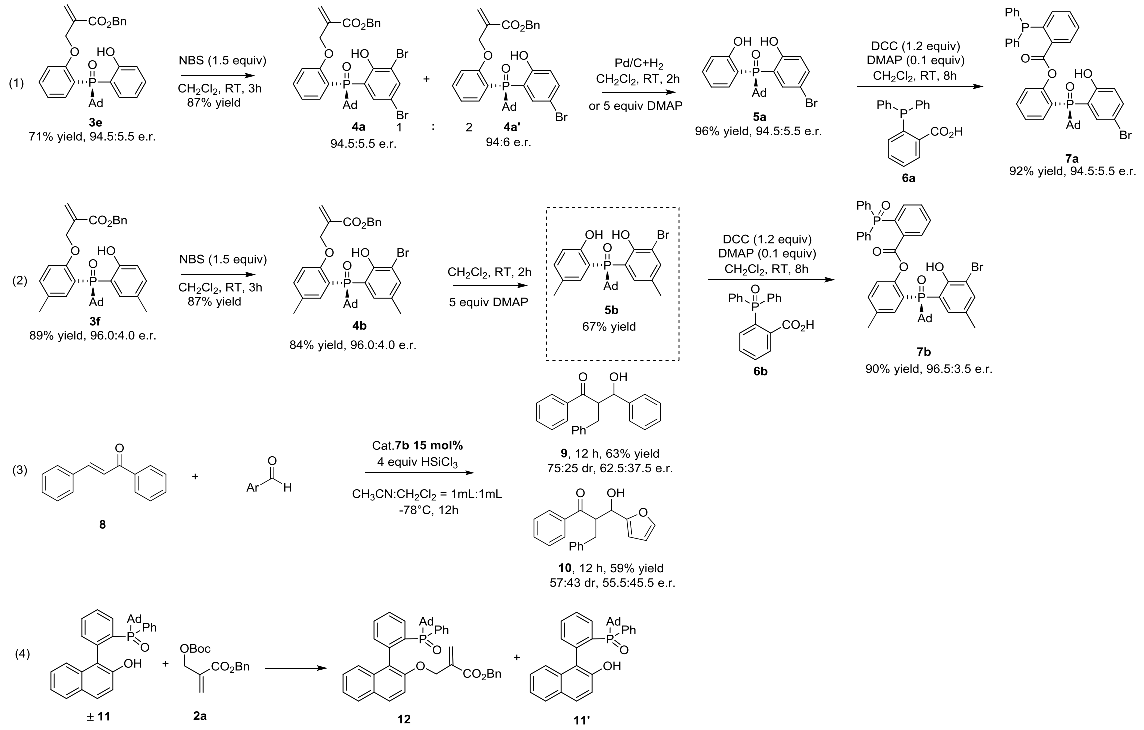

(1) The compound **3e** (0.1 mmol, 54.2 mg) was dissolved in 5mL CH_2Cl_2, and NBS (1.5 equiv, 0.15 mmol, 26.7 mg) was added. The reaction was stirred at room temperature for 3 h. The products **4a** and **4a'** can be separated by using PE: CH_2Cl_2 = 2:1 in silica gel plate. And then, compound **4a'** was dissolved in 5mL CH_2Cl_2, 10mg Pd/C was added. The reaction was stirred at room temperature for 2 h under H_2 atmosphere. The products **6** was separated by using CH_2Cl_2 in silica gel plate. The compound **5a**, **6a** (1.0 equiv), DCC (1.2 equiv) and DMAP (0.1 equiv) were dissolved in 1mL CH_2Cl_2. The reaction was stirred at room temperature for 8 h. The final product was separated by using PE: CH_2Cl_2 = 5:1 in silica gel plate.

(2) The compound **3f** (0.1 mmol, 57.0 mg) was dissolved in 2mL CH_2Cl_2, and NBS (1.5 equiv, 0.15 mmol, 26.7 mg) was added. The reaction was stirred at room temperature for 3 h. The products **4b** can be separated by using PE: CH_2Cl_2 = 2:1 in silica gel plate. And then, compound **4b** was dissolved in 2mL CH_2Cl_2, 61mg DMAP (5 equiv) was added. The reaction was stirred at room temperature for 2 h under. The products **5b** was separated by using CH_2Cl_2 in silica gel plate. The compound **5b**, **6b** (1.0 equiv), DCC (1.2 equiv) and DMAP (0.1 equiv) were dissolved in 1mL CH_2Cl_2. The reaction was stirred at room temperature for 8 h. The final product **7b** was separated by using EA in silica gel plate.

(3) The compound **8** (0.2 mmol, 41.6 mg), catalyst **7b** (15 mol%, 23.3mg) and aldehydes (1.2 equiv, 0.24 mmol, 25.4 mg) was dissolved in 1mL CH_2Cl_2:1ml CH_3CN at -78 °C. And then 4 equiv $HSiCl_3$ (108 mg, 80 μL) was added. The reaction was stirred for 12 h. The mixture was chromatographed on a silica gel column eluted with PE:EA = 4:1 to afford the desired products **9** and **10**.

(4) The compound **11** (0.1 mmol, 47.8 mg), catalyst **hydroquinine** (10 mol%, 3.26mg) and **2a** (0.055 equiv, 0.055 mmol, 14.2 mg) was dissolved in 1mL CH_2Cl_2. The reaction was stirred at room temperature for 12 h. The mixture was chromatographed on a silica gel column eluted with PE:EA = 4:1 to afford the desired products **12** and **S11'**.

1. P. Huszthy, Viktor. Farkas, T. Toth, G. Szekely, Miklos. Hollosi, *Tetrahedron*, 2008, **64**, 10107.
2. B. Dhawan, D. Redmore, *J. Org. Chem.*, 1986, **51**, 179.
3. H.-B. Yang, Y.-Z. Zhao, R. Sang, M. Shi, *J. Org. Chem.*, 2014, **79**, 3519.
4. T. P. Montgomery, A. Hassan, B. Y. Park, M. J. Krische, *J. Am. Chem. Soc.*, 2012, **134**, 11100; R. Zhou, J.-F. Wang, H.-B. Song, Z.-J. He, *Org. Lett.*, 2011, **13**, 580.

2. Linear free energy relation (LFER) researches

2.1 Linear FER between the log (e.r.) values of products **3** and the Charton constants[1]

Table 1 The log (e.r.) values of products and the Charton constants. (Conditions: -40 °C, 20 mol% catalyst, 6d, $CHCl_3$)

products		Charton constants	substituent	e.r.values	log (e.r.)
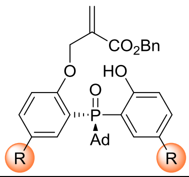	**3e**	0.00	H	94.4:5.6	1.2268
	**3f**	0.52	Me	96.2:3.8	1.4034
	**3g**	0.56	Et	96.6:3.4	1.4535
	**3h**	0.76	*i*-Pr	96.9:3.1	1.4950
	**3l**	0.27	F	88.5:11.5	0.8862
	**3m**	0.55	Cl	95.8:4.2	1.3581
	**3n**	0.65	Br	97.4:2.6	1.5736
	**3o**	0.78	I	98.4:1.6	1.7889

Using these data we got in Table 1, a very good linear FER (R^2 = 0.97) between the log (e.r.) values and the Charton constants of the alkyl substituted products **3e-h** was founded. And halogen substituted products **3l-o** have their own

linear correlation between the log (e.r.) values and the Charton constants with $R^2 = 0.99$.

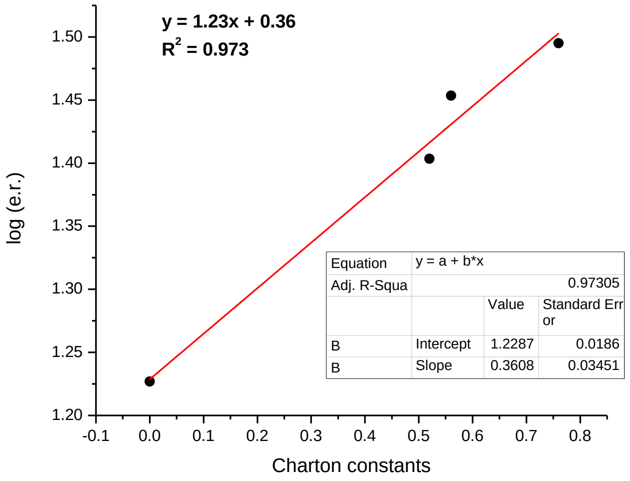

Fig. 1 LFER of the products (-40 °C) 3e-h between log (e.r.) values and the Charton constants.

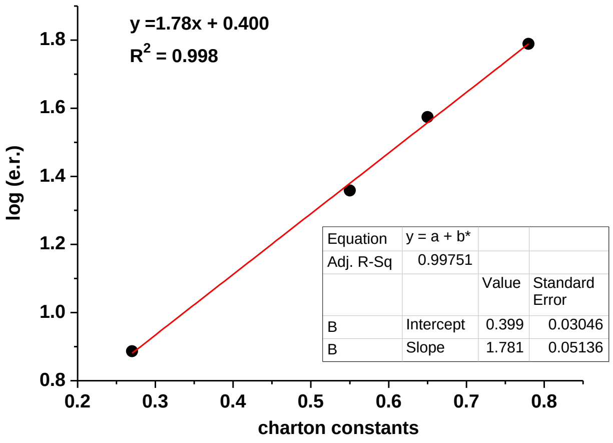

Fig. 2 LFER of the products (-40 °C) 3l-m,3o between log (e.r.) values and the Charton constants.

2.2 Linear FER between the log (e.r.) values of products **5** and the Charton constants

Table 2 The log (e.r.) values of products 5 and the Charton constants.(Conditions: RT, 10 mol% catalyst, 24h, $CHCl_3$)

products		Charton values	substrates	e.r. values	log (e.r.)
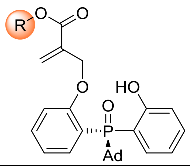	**5a**	0.52	Me	83.1：16.9	0.692
	**5b**	0.56	Et	83.9：16.1	0.717
	**5c**	0.70	Bn	85.2：14.8	0.760
	**5d**	0.87	Hex	84.6：15.4	0.740
	**5e**	1.24	*t*-Bu	87.8：12.2	0.857
	**5f**[2]	1.33	Ad	88.4：11.6	0.882

b. Using the data we got in Table 2, we also got a considerable linear FER between the log (e.r.) values of products **5** and

the Charton constants with $R^2 = 0.92$.

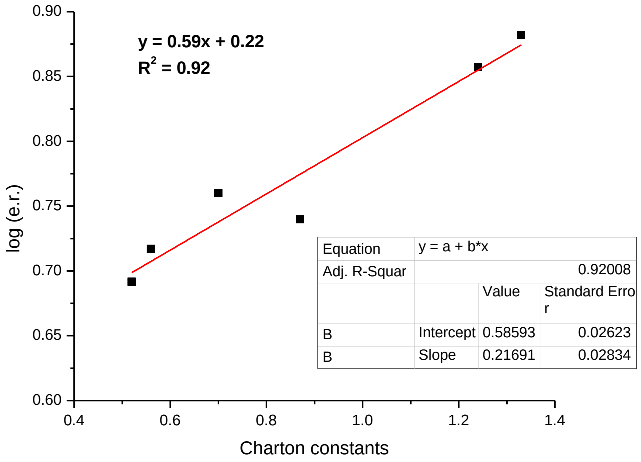

Fig. 3 LFER of the products (RT) 5a-5f between log (e.r.) values and the Charton constants.

Reference of Linear free energy relation (LFER) researches:

1. (*a*) M. Charton, *J. Am. Chem Soc.*, 1969, **91**, 615; (*b*) M. Charton, *J. Am. Chem. Soc.*, 1975, **97**, 1552; (*c*) M. Charton, *J. Am. Chem. Soc.*, 1975, **97**, 3691; (*d*) M. Charton, *J. Am. Chem. Soc.*, 1975, **97**, 3694.
2. J. J. Miller and M. S. Sigman, *Angew. Chem. Int. Ed*., 2008, **47**, 771.

2.3 Correlation studies related to the computation

All substrates mono negative ion studied here were geometrically optimized at M06-2X/6-311g(d,p) level (SDD for I atom) with Gaussian 09 software. [1-3] SMD solvating model was used for describe the solvating effect of chloroform. [4] IR vibration values were obtained by performing frequency analysis at the same level. Sterimol parameters were calculated for the geometry optimized structures using Molecular Modeling Pro software. [5] NPA charges were obtained using NBO package build-in Gaussian 09. [6] Charton values were taken from literature. [7] The stepwise regression was performed using Matlab (R2018a) software. [8] All parameters examined in this study were listed in Table S1-S2.

Table 3 Parameters examined in this study

Substrates	L	B_1	B_5
POMe	4.520	1.520	3.245
POEt	5.539	1.520	3.232
POiPr	5.517	1.520	4.122
POAd	7.078	1.520	5.882
PAd (H)	6.765	3.274	3.787
P*t*Bu	4.733	2.916	3.360
PTrityl	6.860	4.603	6.527

Table 4 Parameters examined in this study

Substrates	L	B_1	B_5	σ	σ-	σ_m	NPA(O^-)
H	2.254	1.17	1.17	0.00	0.00	0	-0.827
Me	3.069	1.7	2.192	-0.17	-0.17	-0.07	-0.832
Et	4.342	1.715	3.331	-0.15	-0.19	-0.07	-0.832

*i*Pr	4.346	2.079	3.345	-0.15	-0.16	-0.04	-0.831
F	2.819	1.47	1.47	0.06	-0.03	0.34	-0.832
Cl	3.481	1.73	1.73	0.23	0.19	0.37	-0.818
Br	3.853	1.95	1.95	0.23	0.25	0.39	-0.817
OMe	4.249	1.52	3.214	-0.27	-0.26	0.12	-0.844
*t*Bu	4.365	2.937	3.356	-0.20	-0.13	-0.1	-0.835
I	4.228	2.1	2.1	0.18	0.27	0.35	-0.815
chex	6.408	2.083	3.642	-0.15	-0.14	-0.05	-0.831
P*t*Bu	2.254	1.17	1.17	0.00	0.00	0	-0.826

(continued)

Substrates	NPA(O)	ν(C-O$^-$)	ν(O-H)	L(P)	B_1(P)	B_5(P)	charton values
H	-0.741	1559.48	3092.29	6.765	3.274	3.787	0.00
Me	-0.744	1561.1	3113.66	6.768	3.260	3.815	0.52
Et	-0.744	1560.00	3123.63	6.760	3.267	3.803	0.56
*i*Pr	-0.742	1559.39	3089.76	6.754	3.265	3.822	0.76
F	-0.740	1554.95	3131.63	6.768	3.261	3.819	0.27
Cl	-0.734	1554.77	3093.11	6.770	3.268	3.812	0.55
Br	-0.734	1553.53	3054.09	6.777	3.262	3.816	0.65
OMe	-0.748	1548.30	3162.61	6.786	3.258	3.822	
*t*Bu	-0.744	1578.77	3157.53	6.777	3.267	3.811	1.24
I	-0.733	1554.32	3081.29	6.759	3.268	3.805	0.73
chex	-0.743	1570.64	3095.32	6.768	3.262	3.809	0.87
P*t*Bu	-0.740	1560.53	3079.00	4.733	2.916	3.360	

Table 5 Experimental and Predicted ΔΔG(e.r.)‡

Substrates	Experimental ΔΔG(e.r.)‡	Predicted ΔΔG(e.r.)‡	Predicted ΔΔG(e.r.)‡
H	1.308	1.406	1.366
Me	1.496	1.508	1.455
Et	1.550	1.462	1.416
*i*Pr	1.594	1.549	1.502
F	0.945	0.895	0.848
Cl	1.448	1.480	1.435
Br	1.678	1.576	1.526
OMe	1.066	1.048	1.004
*t*Bu	1.157	1.116	1.063
I	1.907	1.874	1.827
chex	1.393	1.392	1.337
P*t*Bu	1.198		1.132

POMe

Standard orientation:

Center Number	Atomic Number	Atomic Type	Coordinates (Angstroms) X	Y	Z
1	15	0	0.016005	0.887719	0.375074
2	8	0	0.231037	2.284202	-0.166468
3	8	0	2.596139	1.786596	-1.067594
4	1	0	1.734997	2.203497	-0.803598
5	6	0	1.503051	-0.079070	0.037432
6	6	0	1.587650	-1.417794	0.440895
7	1	0	0.732465	-1.848564	0.953063
8	6	0	2.719653	-2.172022	0.173092
9	1	0	2.771078	-3.207588	0.486893
10	6	0	3.788634	-1.582887	-0.504145
11	1	0	4.681197	-2.160924	-0.718582
12	6	0	3.725069	-0.261092	-0.909934
13	1	0	4.548529	0.211507	-1.433007
14	6	0	2.585742	0.509929	-0.647154
15	6	0	-1.471006	0.160729	-0.272483
16	6	0	-1.974850	-1.084793	0.257382
17	6	0	-3.188789	-1.549505	-0.366716
18	1	0	-3.598937	-2.481723	0.009148
19	6	0	-3.805954	-0.862125	-1.384646
20	1	0	-4.718353	-1.264569	-1.816708
21	6	0	-3.285318	0.349255	-1.878764
22	1	0	-3.782440	0.882290	-2.679824
23	6	0	-2.124183	0.842251	-1.312012
24	1	0	-1.698166	1.777640	-1.662399
25	8	0	-1.418668	-1.715818	1.197454
26	8	0	-0.034318	0.837221	1.984568

27	6	0	-1.264025	1.081876	2.676538
28	1	0	-0.998878	1.302117	3.709894
29	1	0	-1.795006	1.938742	2.254510
30	1	0	-1.884128	0.185659	2.633902

POEt

Standard orientation:

Center Number	Atomic Number	Atomic Type	Coordinates (Angstroms) X	Y	Z
1	15	0	-0.031795	0.483390	0.675724
2	8	0	-0.245750	0.597794	2.169644
3	8	0	-2.680154	-0.258116	2.084892
4	1	0	-1.795398	0.099928	2.355790
5	6	0	-1.566435	-0.111254	-0.067556
6	6	0	-1.658454	-0.297604	-1.452757
7	1	0	-0.783956	-0.068768	-2.054515
8	6	0	-2.821719	-0.783073	-2.030072
9	1	0	-2.878831	-0.926322	-3.102240
10	6	0	-3.913761	-1.086249	-1.215146
11	1	0	-4.829806	-1.467843	-1.653274
12	6	0	-3.843919	-0.905433	0.155565
13	1	0	-4.685084	-1.133082	0.800243
14	6	0	-2.674157	-0.415502	0.749847
15	6	0	1.375776	-0.535434	0.298243
16	6	0	1.880015	-0.634568	-1.051906
17	6	0	3.019394	-1.506730	-1.196869
18	1	0	3.426231	-1.609398	-2.198021
19	6	0	3.576929	-2.177828	-0.134047

20	1	0	4.435724	-2.820886	-0.307424
21	6	0	3.063278	-2.051221	1.170604
22	1	0	3.514247	-2.581261	2.000295
23	6	0	1.967983	-1.229735	1.364337
24	1	0	1.545226	-1.102567	2.356302
25	8	0	1.383983	-0.005416	-2.026624
26	8	0	0.143864	1.910072	-0.050459
27	6	0	1.432634	2.550880	-0.096579
28	1	0	1.912040	2.486502	0.884820
29	1	0	2.041839	2.023276	-0.832879
30	6	0	1.210066	3.990728	-0.495481
31	1	0	0.591758	4.504756	0.242943
32	1	0	2.169574	4.507702	-0.570790
33	1	0	0.712028	4.040563	-1.465838

PO*i*Pr

Standard orientation:

Center Number	Atomic Number	Atomic Type	Coordinates (Angstroms) X	Y	Z
1	15	0	-0.070494	0.340346	0.699294
2	8	0	-0.269149	0.423560	2.197579
3	8	0	-2.745134	-0.309975	2.104712
4	1	0	-1.836570	-0.024539	2.382581
5	6	0	-1.669409	-0.034927	-0.053652
6	6	0	-1.808875	-0.058907	-1.446595
7	1	0	-0.929115	0.150496	-2.046872
8	6	0	-3.025496	-0.366561	-2.035994
9	1	0	-3.119884	-0.384153	-3.114954

10	6	0	-4.123975	-0.654154	-1.224103
11	1	0	-5.081953	-0.894946	-1.672327
12	6	0	-4.007601	-0.632444	0.155297
13	1	0	-4.853218	-0.847784	0.798445
14	6	0	-2.783794	-0.321375	0.760598
15	6	0	1.187223	-0.840679	0.273125
16	6	0	1.665664	-0.947288	-1.086975
17	6	0	2.661633	-1.971163	-1.286928
18	1	0	3.047255	-2.082265	-2.295482
19	6	0	3.109621	-2.775323	-0.265010
20	1	0	3.860061	-3.532149	-0.477878
21	6	0	2.622224	-2.641392	1.048798
22	1	0	2.985039	-3.279973	1.844407
23	6	0	1.666141	-1.672734	1.295812
24	1	0	1.268562	-1.537481	2.297138
25	8	0	1.265424	-0.201510	-2.021658
26	8	0	0.271566	1.748726	0.004462
27	6	0	1.615179	2.264524	-0.129523
28	1	0	2.297483	1.421917	-0.266064
29	6	0	1.977144	3.032487	1.129440
30	1	0	1.944972	2.377418	2.002989
31	1	0	2.984532	3.447412	1.042503
32	1	0	1.270678	3.851976	1.286196
33	6	0	1.613688	3.115750	-1.381367
34	1	0	0.893211	3.932377	-1.283017
35	1	0	2.605941	3.541666	-1.550022
36	1	0	1.342558	2.493544	-2.235345

POAd

Standard orientation:

Center Number	Atomic Number	Atomic Type	Coordinates (Angstroms) X	Y	Z
1	15	0	0.974181	-0.096715	0.615991
2	8	0	0.921581	-0.419169	2.094861
3	8	0	3.065895	-1.878426	2.030250
4	1	0	2.237860	-1.398442	2.287157
5	6	0	2.442726	-0.879662	-0.092390
6	6	0	2.755901	-0.705572	-1.445789
7	1	0	2.091253	-0.092533	-2.047925
8	6	0	3.890418	-1.283971	-1.994667
9	1	0	4.122805	-1.139537	-3.042792
10	6	0	4.728093	-2.049459	-1.182017
11	1	0	5.619210	-2.506375	-1.598868
12	6	0	4.435295	-2.233002	0.158759
13	1	0	5.075364	-2.826565	0.801450
14	6	0	3.291678	-1.653803	0.722468
15	6	0	-1.605116	-0.698936	-0.121909
16	6	0	-2.073717	0.705461	0.267461
17	1	0	-1.676400	0.974644	1.252111
18	1	0	-1.695315	1.427822	-0.460494
19	6	0	-3.613105	0.727434	0.299746
20	1	0	-3.942771	1.731774	0.580292
21	6	0	-4.120812	-0.292130	1.330393
22	1	0	-3.748517	-0.032225	2.327101
23	1	0	-5.215543	-0.270580	1.370051
24	6	0	-3.639361	-1.697607	0.941279
25	1	0	-3.993475	-2.426906	1.675944
26	6	0	-4.183994	-2.056443	-0.450081

27	1	0	-5.279567	-2.059944	-0.431106
28	1	0	-3.856577	-3.063084	-0.732145
29	6	0	-3.681015	-1.033980	-1.481896
30	1	0	-4.060462	-1.293455	-2.474521
31	6	0	-2.146143	-1.047837	-1.507050
32	1	0	-1.752774	-0.316156	-2.219155
33	1	0	-1.770720	-2.036077	-1.791379
34	6	0	-2.102171	-1.715724	0.908396
35	1	0	-1.686616	-1.465957	1.889624
36	1	0	-1.732440	-2.707710	0.628758
37	6	0	-4.162173	0.369685	-1.089659
38	1	0	-5.257264	0.402692	-1.078261
39	1	0	-3.815144	1.101751	-1.826451
40	6	0	0.976801	1.659394	0.327786
41	6	0	0.772035	2.199672	-0.997101
42	6	0	0.778454	3.640569	-1.054671
43	1	0	0.621048	4.086177	-2.032245
44	6	0	0.969571	4.427309	0.057757
45	1	0	0.964792	5.508556	-0.052290
46	6	0	1.169130	3.865072	1.331934
47	1	0	1.318472	4.492962	2.201480
48	6	0	1.166143	2.485504	1.444037
49	1	0	1.301831	2.016264	2.413838
50	8	0	0.585319	1.488225	-2.021964
51	8	0	-0.169495	-0.821659	-0.242740

PAd(H)

Standard orientation:

Center Atomic Atomic Coordinates (Angstroms)

Number	Number	Type	X	Y	Z
1	15	0	0.626063	0.037082	-0.764140
2	8	0	0.690681	0.286933	-2.273715
3	8	0	0.827188	2.840952	-1.974940
4	1	0	0.717127	1.910097	-2.315789
5	6	0	1.238273	1.556184	0.038712
6	6	0	1.695011	1.558359	1.361517
7	1	0	1.679001	0.622521	1.912731
8	6	0	2.128535	2.730846	1.964496
9	1	0	2.475919	2.718493	2.990549
10	6	0	2.106984	3.922127	1.238736
11	1	0	2.440186	4.845699	1.699963
12	6	0	1.669525	3.939278	-0.075655
13	1	0	1.658380	4.853980	-0.657356
14	6	0	1.237429	2.759931	-0.694036
15	6	0	-1.144507	-0.174850	-0.234202
16	6	0	-1.518607	-1.671161	-0.263769
17	1	0	-1.306070	-2.091049	-1.254172
18	1	0	-0.910027	-2.206848	0.468970
19	6	0	-3.008646	-1.840371	0.071906
20	1	0	-3.259061	-2.905686	0.047492
21	6	0	-3.860206	-1.083280	-0.956919
22	1	0	-3.680971	-1.487151	-1.959525
23	1	0	-4.925003	-1.217218	-0.733783
24	6	0	-3.501756	0.409723	-0.920880
25	1	0	-4.099902	0.950737	-1.660880
26	6	0	-3.781639	0.967248	0.481857
27	1	0	-4.844847	0.855167	0.724499
28	1	0	-3.547682	2.037431	0.512618

29	6	0	-2.921102	0.215434	1.506450
30	1	0	-3.105284	0.616606	2.507959
31	6	0	-1.434276	0.403896	1.166375
32	1	0	-0.799310	-0.097343	1.899378
33	1	0	-1.194350	1.472396	1.178334
34	6	0	-2.013406	0.585247	-1.263412
35	1	0	-1.811925	0.206663	-2.268661
36	1	0	-1.750038	1.650489	-1.259564
37	6	0	-3.274056	-1.278164	1.476839
38	1	0	-4.327325	-1.420932	1.744822
39	1	0	-2.668140	-1.818041	2.212447
40	6	0	1.659967	-1.368730	-0.335337
41	6	0	1.676613	-1.918724	0.993758
42	6	0	2.548377	-3.049367	1.162529
43	1	0	2.582803	-3.494264	2.152070
44	6	0	3.308583	-3.556140	0.131021
45	1	0	3.950953	-4.412350	0.319145
46	6	0	3.270180	-2.992062	-1.154290
47	1	0	3.869946	-3.398065	-1.959484
48	6	0	2.439654	-1.902907	-1.367768
49	1	0	2.374050	-1.444280	-2.349555
50	8	0	0.977764	-1.450183	1.941445

P*t*Bu

Standard orientation:

Center Number	Atomic Number	Atomic Type	Coordinates (Angstroms) X	Y	Z
1	15	0	0.014253	0.580858	0.490591

2	8	0	-0.226993	1.045833	1.929681
3	8	0	-2.692368	0.326314	1.898975
4	1	0	-1.801039	0.726247	2.104003
5	6	0	-1.360866	-0.539132	0.067586
6	6	0	-1.277978	-1.436790	-1.003012
7	1	0	-0.364671	-1.455018	-1.590938
8	6	0	-2.343046	-2.267797	-1.321492
9	1	0	-2.265560	-2.956864	-2.153673
10	6	0	-3.510777	-2.205760	-0.560390
11	1	0	-4.351077	-2.848182	-0.800683
12	6	0	-3.608683	-1.332642	0.510428
13	1	0	-4.503857	-1.282991	1.119950
14	6	0	-2.537039	-0.495515	0.843560
15	6	0	1.590650	-0.273557	0.386496
16	6	0	2.145343	-0.678537	-0.877125
17	6	0	3.420025	-1.337514	-0.789408
18	1	0	3.868658	-1.656529	-1.724999
19	6	0	4.054045	-1.559957	0.413319
20	1	0	5.016867	-2.063816	0.418768
21	6	0	3.484657	-1.148683	1.629456
22	1	0	3.992460	-1.324491	2.569734
23	6	0	2.256168	-0.507122	1.596091
24	1	0	1.788585	-0.167050	2.515053
25	8	0	1.562636	-0.469999	-1.982381
26	6	0	-0.103331	2.044618	-0.659234
27	6	0	-1.067099	3.032024	0.015528
28	1	0	-1.194100	3.901047	-0.638264
29	1	0	-2.054578	2.586380	0.173714
30	1	0	-0.690351	3.369319	0.981840
31	6	0	1.290050	2.668845	-0.786084

32	1	0	1.951750	2.011017	-1.353598
33	1	0	1.210695	3.624832	-1.314518
34	1	0	1.739326	2.861682	0.192699
35	6	0	-0.653578	1.686982	-2.043197
36	1	0	-1.685154	1.330960	-1.979529
37	1	0	-0.649789	2.591407	-2.661870
38	1	0	-0.039397	0.925209	-2.521894

PTrityl

Standard orientation:

Center Number	Atomic Number	Atomic Type	Coordinates (Angstroms) X	Y	Z
1	15	0	-0.046460	-0.633563	-0.889520
2	8	0	0.042719	-0.189387	-2.349953
3	8	0	2.243035	-1.313831	-2.850328
4	1	0	1.369358	-0.822565	-2.881774
5	6	0	1.388195	-1.734911	-0.606879
6	6	0	1.534760	-2.488401	0.567173
7	1	0	0.774772	-2.383691	1.333044
8	6	0	2.598403	-3.360971	0.737086
9	1	0	2.688936	-3.928545	1.655411
10	6	0	3.536452	-3.507914	-0.284500
11	1	0	4.373836	-4.186761	-0.164207
12	6	0	3.394179	-2.805607	-1.467597
13	1	0	4.095856	-2.924388	-2.285092
14	6	0	2.320011	-1.926809	-1.655207
15	6	0	-1.584910	-1.511057	-0.612752
16	6	0	-1.948888	-2.099042	0.655976

17	6	0	-3.269399	-2.681297	0.682113
18	1	0	-3.579302	-3.124316	1.623472
19	6	0	-4.105653	-2.678423	-0.409729
20	1	0	-5.090159	-3.130860	-0.323574
21	6	0	-3.711957	-2.106154	-1.633061
22	1	0	-4.373051	-2.111806	-2.490645
23	6	0	-2.456576	-1.528408	-1.711626
24	1	0	-2.128032	-1.060846	-2.634569
25	8	0	-1.201516	-2.105060	1.668122
26	6	0	0.011983	0.971833	0.174765
27	6	0	-1.153991	1.791376	-0.414040
28	6	0	-2.489801	1.404276	-0.229050
29	6	0	-0.914836	2.908385	-1.222550
30	6	0	-3.535274	2.117766	-0.803774
31	1	0	-2.726165	0.529435	0.360431
32	6	0	-1.960805	3.624046	-1.796946
33	1	0	0.099138	3.228811	-1.421816
34	6	0	-3.277952	3.236278	-1.588315
35	1	0	-4.553900	1.785365	-0.639034
36	1	0	-1.735758	4.484969	-2.416405
37	1	0	-4.093210	3.791153	-2.038718
38	6	0	-0.098418	0.667315	1.681267
39	6	0	1.005683	0.066768	2.299331
40	6	0	-1.171680	1.035568	2.489620
41	6	0	1.008563	-0.222963	3.653855
42	1	0	1.883392	-0.166970	1.708590
43	6	0	-1.169870	0.751886	3.854615
44	1	0	-2.021346	1.562430	2.081415
45	6	0	-0.091549	0.107610	4.441788
46	1	0	1.874942	-0.701827	4.096611

47	1	0	-2.023539	1.043792	4.456305
48	1	0	-0.096506	-0.119258	5.501891
49	6	0	1.333144	1.751622	-0.009523
50	6	0	1.551226	2.828321	0.863048
51	6	0	2.291684	1.506332	-0.989486
52	6	0	2.682401	3.622923	0.765155
53	1	0	0.816388	3.043783	1.630885
54	6	0	3.439801	2.296470	-1.081065
55	1	0	2.158931	0.720607	-1.716384
56	6	0	3.642613	3.355103	-0.209144
57	1	0	2.817388	4.448695	1.454446
58	1	0	4.171451	2.073999	-1.849627
59	1	0	4.534356	3.966943	-0.283885

Me

Standard orientation:

Center Number	Atomic Number	Atomic Type	Coordinates (Angstroms) X	Y	Z
1	15	0	0.370788	-0.033749	-0.791081
2	8	0	0.438188	-0.167049	-2.315589
3	8	0	-0.092069	2.327772	-2.686628
4	1	0	0.051841	1.345390	-2.775752
5	6	0	0.540892	1.742737	-0.418962
6	6	0	0.929024	2.193611	0.848436
7	1	0	1.119046	1.450362	1.619542
8	6	0	1.045962	3.548422	1.139467
9	6	0	0.761410	4.462772	0.118899
10	1	0	0.840167	5.527166	0.321070

11	6	0	0.385902	4.041976	-1.145701
12	1	0	0.176714	4.752594	-1.937458
13	6	0	0.276092	2.677726	-1.436342
14	6	0	-1.314810	-0.548643	-0.200139
15	6	0	-1.311973	-2.068777	0.061100
16	1	0	-0.957099	-2.602030	-0.828892
17	1	0	-0.623627	-2.288887	0.882300
18	6	0	-2.729014	-2.534948	0.426573
19	1	0	-2.714397	-3.615010	0.602761
20	6	0	-3.697042	-2.210736	-0.720802
21	1	0	-3.384287	-2.731935	-1.632507
22	1	0	-4.704753	-2.561645	-0.470232
23	6	0	-3.715514	-0.694512	-0.966141
24	1	0	-4.397612	-0.463603	-1.790596
25	6	0	-4.178749	0.023309	0.308897
26	1	0	-5.191333	-0.301300	0.575884
27	1	0	-4.213856	1.105021	0.137472
28	6	0	-3.201233	-0.293137	1.448345
29	1	0	-3.514814	0.226057	2.359488
30	6	0	-1.792799	0.191484	1.066122
31	1	0	-1.083795	0.012207	1.875643
32	1	0	-1.822736	1.268529	0.869057
33	6	0	-2.301514	-0.228725	-1.348024
34	1	0	-1.978737	-0.727180	-2.265365
35	1	0	-2.301002	0.850374	-1.547411
36	6	0	-3.180660	-1.808219	1.701736
37	1	0	-4.178387	-2.153047	1.997731
38	1	0	-2.492407	-2.037802	2.522511
39	6	0	1.701669	-0.977015	-0.037883
40	6	0	1.784748	-1.164539	1.382220

41	6	0	2.897913	-1.968098	1.809111
42	1	0	2.993619	-2.139891	2.876821
43	6	0	3.804025	-2.505258	0.923015
44	1	0	4.626069	-3.106833	1.306880
45	6	0	3.705505	-2.304085	-0.467833
46	6	0	2.641570	-1.537146	-0.916845
47	8	0	0.951068	-0.670016	2.200408
48	6	0	1.488883	4.022161	2.500412
49	1	0	1.363196	3.237082	3.247873
50	1	0	0.915644	4.895369	2.821713
51	1	0	2.545436	4.307668	2.493271
52	1	0	2.513930	-1.359900	-1.981451
53	6	0	4.714624	-2.909947	-1.411233
54	1	0	4.493799	-2.636087	-2.445142
55	1	0	5.731470	-2.571170	-1.188157
56	1	0	4.719136	-4.003377	-1.352932

Et

Standard orientation:

Center Number	Atomic Number	Atomic Type	Coordinates (Angstroms) X	Y	Z
1	15	0	-0.040600	-0.230209	0.808219
2	8	0	-0.117327	-0.583599	2.296465
3	8	0	0.403468	1.842445	3.015005
4	1	0	0.266974	0.856858	2.966360
5	6	0	-0.253100	1.577096	0.695436
6	6	0	-0.666051	2.196993	-0.488400
7	1	0	-0.857441	1.571237	-1.357446

8	6	0	-0.805199	3.578463	-0.581417
9	6	0	-0.520918	4.343847	0.554304
10	1	0	-0.618476	5.425136	0.507055
11	6	0	-0.120585	3.752703	1.741981
12	1	0	0.091821	4.345509	2.624637
13	6	0	0.012272	2.363405	1.832624
14	6	0	1.659878	-0.619627	0.163405
15	6	0	1.672825	-2.064574	-0.374802
16	1	0	1.317612	-2.755959	0.398705
17	1	0	0.991687	-2.132786	-1.227172
18	6	0	3.096216	-2.443790	-0.809868
19	1	0	3.089792	-3.470501	-1.188934
20	6	0	4.050302	-2.339447	0.388169
21	1	0	3.733173	-3.027451	1.179869
22	1	0	5.063706	-2.628734	0.086818
23	6	0	4.052863	-0.897252	0.917228
24	1	0	4.724623	-0.822774	1.778307
25	6	0	4.523129	0.052724	-0.193125
26	1	0	5.540516	-0.210709	-0.505460
27	1	0	4.549987	1.082161	0.181281
28	6	0	3.560470	-0.045770	-1.384362
29	1	0	3.881736	0.638021	-2.176463
30	6	0	2.143340	0.347543	-0.936612
31	1	0	1.446783	0.311802	-1.775434
32	1	0	2.157499	1.370287	-0.545008
33	6	0	2.631424	-0.516343	1.362426
34	1	0	2.298568	-1.177877	2.165954
35	1	0	2.623123	0.506073	1.760497
36	6	0	3.553511	-1.485551	-1.919254
37	1	0	4.556694	-1.762539	-2.263590

38	1	0	2.875616	-1.560372	-2.776534
39	6	0	-1.340775	-1.083898	-0.092648
40	6	0	-1.413985	-1.056218	-1.526849
41	6	0	-2.502285	-1.815330	-2.079118
42	1	0	-2.587501	-1.828428	-3.161422
43	6	0	-3.394790	-2.508414	-1.291347
44	1	0	-4.196398	-3.069443	-1.769088
45	6	0	-3.300560	-2.525147	0.113155
46	6	0	-2.262352	-1.801436	0.682524
47	8	0	-0.587260	-0.424926	-2.253419
48	6	0	-1.301297	4.221900	-1.853722
49	1	0	-0.989580	3.616675	-2.709132
50	1	0	-0.839947	5.207326	-1.970775
51	1	0	-2.142127	-1.789757	1.763040
52	6	0	-4.319065	-3.257544	0.953717
53	1	0	-3.882794	-3.491789	1.929469
54	1	0	-4.566305	-4.214829	0.481997
55	6	0	-2.826248	4.367186	-1.865806
56	1	0	-3.162904	4.984902	-1.028773
57	1	0	-3.172427	4.829996	-2.793663
58	1	0	-3.303886	3.388542	-1.772647
59	6	0	-5.607670	-2.453865	1.157582
60	1	0	-6.072241	-2.217928	0.196125
61	1	0	-6.334404	-3.007889	1.758890
62	1	0	-5.393642	-1.508901	1.663515

***i*Pr**

Standard orientation:

Center Atomic Atomic Coordinates (Angstroms)

Number	Number	Type	X	Y	Z
1	15	0	-0.130423	-0.354444	0.836734
2	8	0	-0.514505	-0.721486	2.273650
3	8	0	1.168385	0.922454	3.309184
4	1	0	0.514922	0.191251	3.126221
5	6	0	0.793603	1.214000	0.930585
6	6	0	0.978745	2.020714	-0.199454
7	1	0	0.570689	1.674533	-1.146881
8	6	0	1.671678	3.224005	-0.136518
9	6	0	2.183790	3.618468	1.106562
10	1	0	2.728678	4.554277	1.192247
11	6	0	2.005921	2.844366	2.239498
12	1	0	2.390625	3.159067	3.203150
13	6	0	1.307642	1.632394	2.171233
14	6	0	1.027922	-1.641605	0.163958
15	6	0	0.186933	-2.782259	-0.444039
16	1	0	-0.533475	-3.152299	0.295344
17	1	0	-0.376447	-2.394726	-1.297951
18	6	0	1.109632	-3.921419	-0.900853
19	1	0	0.499532	-4.724577	-1.325568
20	6	0	1.910005	-4.455604	0.295902
21	1	0	1.227150	-4.850159	1.056571
22	1	0	2.556759	-5.280664	-0.024689
23	6	0	2.761439	-3.323888	0.891575
24	1	0	3.324144	-3.700636	1.751653
25	6	0	3.732715	-2.801360	-0.175333
26	1	0	4.397779	-3.609434	-0.502127
27	1	0	4.360517	-2.007994	0.245394
28	6	0	2.930559	-2.257492	-1.364801

29	1	0	3.614628	-1.870256	-2.126554
30	6	0	2.022697	-1.111870	-0.888167
31	1	0	1.468805	-0.681978	-1.723151
32	1	0	2.637127	-0.322532	-0.441619
33	6	0	1.836419	-2.189815	1.363153
34	1	0	1.154830	-2.557447	2.134121
35	1	0	2.428566	-1.381182	1.810706
36	6	0	2.075091	-3.383932	-1.966149
37	1	0	2.720597	-4.192066	-2.329309
38	1	0	1.506984	-3.002943	-2.821552
39	6	0	-1.615636	-0.150721	-0.156641
40	6	0	-1.559916	0.030954	-1.580068
41	6	0	-2.844139	0.167176	-2.211340
42	1	0	-2.842965	0.298588	-3.289041
43	6	0	-4.024537	0.136548	-1.504310
44	1	0	-4.961091	0.250166	-2.046228
45	6	0	-4.056415	-0.036516	-0.105924
46	6	0	-2.835282	-0.180145	0.536415
47	8	0	-0.476570	0.069107	-2.238294
48	6	0	1.868134	4.080440	-1.372287
49	1	0	1.368554	3.568576	-2.200535
50	6	0	3.352438	4.221919	-1.724937
51	1	0	3.477514	4.800131	-2.644938
52	1	0	3.893572	4.739599	-0.926726
53	1	0	3.818256	3.243867	-1.868094
54	6	0	1.221632	5.460077	-1.207823
55	1	0	0.158947	5.370145	-0.971307
56	1	0	1.702051	6.021813	-0.400628
57	1	0	1.321256	6.044259	-2.127071
58	1	0	-2.808841	-0.325021	1.613419

59	6	0	-5.368295	-0.053207	0.655454
60	1	0	-5.128760	-0.247537	1.706550
61	6	0	-6.299759	-1.170177	0.170822
62	1	0	-5.815397	-2.146633	0.247054
63	1	0	-7.221064	-1.196808	0.761034
64	1	0	-6.580436	-1.015213	-0.875594
65	6	0	-6.081095	1.302107	0.577229
66	1	0	-7.004901	1.298242	1.164062
67	1	0	-5.439018	2.103134	0.951001
68	1	0	-6.343764	1.539576	-0.458743

F

Standard orientation:

Center Number	Atomic Number	Atomic Type	Coordinates (Angstroms) X	Y	Z
1	15	0	0.371820	-0.026266	-0.797140
2	8	0	0.435528	-0.112965	-2.322667
3	8	0	0.065884	2.422721	-2.617942
4	1	0	0.150983	1.438222	-2.740480
5	6	0	0.630912	1.727806	-0.363375
6	6	0	1.021725	2.106715	0.926127
7	1	0	1.163588	1.351747	1.695593
8	6	0	1.192751	3.443768	1.207961
9	6	0	0.993196	4.430983	0.255919
10	1	0	1.139000	5.471273	0.521651
11	6	0	0.615253	4.059136	-1.023622
12	1	0	0.461565	4.802088	-1.797252
13	6	0	0.433924	2.710836	-1.352984

14	6	0	-1.326738	-0.488577	-0.207684
15	6	0	-1.375208	-2.011643	0.032085
16	1	0	-1.040766	-2.544074	-0.866180
17	1	0	-0.694365	-2.268129	0.848944
18	6	0	-2.807652	-2.432607	0.393312
19	1	0	-2.829550	-3.514721	0.554715
20	6	0	-3.764057	-2.059708	-0.748859
21	1	0	-3.470344	-2.579897	-1.667320
22	1	0	-4.783191	-2.377965	-0.501335
23	6	0	-3.730259	-0.540577	-0.973374
24	1	0	-4.403954	-0.273992	-1.793759
25	6	0	-4.166314	0.175710	0.312235
26	1	0	-5.189248	-0.116906	0.576195
27	1	0	-4.163778	1.260206	0.155669
28	6	0	-3.200267	-0.190359	1.446629
29	1	0	-3.494375	0.327766	2.364602
30	6	0	-1.775323	0.249259	1.070714
31	1	0	-1.073730	0.031289	1.877423
32	1	0	-1.767769	1.329880	0.891942
33	6	0	-2.301756	-0.118410	-1.350487
34	1	0	-1.996577	-0.614366	-2.275027
35	1	0	-2.264259	0.962711	-1.535352
36	6	0	-3.233334	-1.708159	1.678894
37	1	0	-4.243043	-2.021113	1.969088
38	1	0	-2.555330	-1.974321	2.497004
39	6	0	1.666509	-1.046358	-0.081951
40	6	0	1.766308	-1.251182	1.335446
41	6	0	2.845464	-2.107311	1.745755
42	1	0	2.951134	-2.288466	2.809920
43	6	0	3.715429	-2.687318	0.847120

44	1	0	4.522821	-3.331403	1.180433
45	6	0	3.560574	-2.446326	-0.515935
46	6	0	2.554577	-1.640770	-0.989878
47	1	0	2.443521	-1.469338	-2.055160
48	8	0	0.965397	-0.721246	2.164759
49	9	0	4.428695	-3.032364	-1.392089
50	9	0	1.563705	3.814140	2.457457

CI

Standard orientation:

Center Number	Atomic Number	Atomic Type	Coordinates (Angstroms) X	Y	Z
1	15	0	-0.217383	-0.143035	0.814359
2	8	0	-0.441579	-0.466411	2.292153
3	8	0	0.861265	1.610003	3.077337
4	1	0	0.393312	0.732031	3.003934
5	6	0	0.228296	1.626083	0.739258
6	6	0	0.094440	2.363700	-0.440993
7	1	0	-0.276983	1.872337	-1.335826
8	6	0	0.464776	3.695273	-0.457833
9	6	0	0.972811	4.319981	0.676016
10	1	0	1.261048	5.363706	0.641109
11	6	0	1.097143	3.597010	1.848802
12	1	0	1.478091	4.061505	2.750573
13	6	0	0.721100	2.249105	1.902659
14	6	0	1.236021	-1.101357	0.165543
15	6	0	0.739538	-2.433605	-0.432418
16	1	0	0.139557	-2.976365	0.307588

17	1	0	0.103645	-2.224036	-1.296636
18	6	0	1.942407	-3.287620	-0.861462
19	1	0	1.574892	-4.228343	-1.283005
20	6	0	2.830921	-3.577470	0.356465
21	1	0	2.260463	-4.129704	1.111532
22	1	0	3.680215	-4.204166	0.061622
23	6	0	3.339663	-2.254060	0.947938
24	1	0	3.965046	-2.458579	1.822713
25	6	0	4.155806	-1.501796	-0.112521
26	1	0	5.019102	-2.104518	-0.417140
27	1	0	4.540032	-0.564886	0.306091
28	6	0	3.262003	-1.205473	-1.324700
29	1	0	3.833303	-0.657111	-2.080007
30	6	0	2.068000	-0.340054	-0.888779
31	1	0	1.434720	-0.096942	-1.744270
32	1	0	2.437251	0.597916	-0.460244
33	6	0	2.141824	-1.397111	1.384346
34	1	0	1.568315	-1.917264	2.155048
35	1	0	2.493926	-0.455116	1.823723
36	6	0	2.751612	-2.525244	-1.921263
37	1	0	3.596902	-3.136786	-2.257759
38	1	0	2.123291	-2.319997	-2.794884
39	6	0	-1.728255	-0.453749	-0.105802
40	6	0	-1.764674	-0.350969	-1.538532
41	6	0	-3.037803	-0.661038	-2.130749
42	1	0	-3.106346	-0.601215	-3.211690
43	6	0	-4.134357	-1.022538	-1.382264
44	1	0	-5.078032	-1.247966	-1.867414
45	6	0	-4.038082	-1.101360	0.011538
46	6	0	-2.846873	-0.822482	0.648762

47	1	0	-2.762573	-0.890939	1.728184
48	8	0	-0.755779	-0.017466	-2.224832
49	17	0	-5.455250	-1.572422	0.951905
50	17	0	0.295979	4.621339	-1.942490

Br

Standard orientation:

Center Number	Atomic Number	Atomic Type	Coordinates (Angstroms) X	Y	Z
1	15	0	0.152033	0.486108	0.819803
2	8	0	0.702679	0.834536	2.203873
3	8	0	-1.300682	-0.222251	3.413827
4	1	0	-0.486666	0.296563	3.157922
5	6	0	-1.082225	-0.833961	1.080666
6	6	0	-1.477022	-1.682458	0.041589
7	1	0	-1.044315	-1.544777	-0.945091
8	6	0	-2.426545	-2.659535	0.281587
9	6	0	-3.002017	-2.814865	1.539193
10	1	0	-3.747239	-3.581668	1.711789
11	6	0	-2.606546	-1.983622	2.572623
12	1	0	-3.028846	-2.091538	3.564810
13	6	0	-1.641063	-0.990743	2.364820
14	6	0	-0.746421	1.947360	0.104185
15	6	0	0.227767	2.769668	-0.763489
16	1	0	1.120882	3.029351	-0.182775
17	1	0	0.544085	2.165172	-1.617269
18	6	0	-0.470770	4.046014	-1.257460
19	1	0	0.231116	4.618239	-1.871783

20	6	0	-0.914133	4.889560	-0.054370
21	1	0	-0.041462	5.174529	0.543650
22	1	0	-1.392293	5.813549	-0.399087
23	6	0	-1.896497	4.080565	0.805815
24	1	0	-2.205503	4.676716	1.670070
25	6	0	-3.124074	3.705373	-0.034427
26	1	0	-3.630937	4.612307	-0.383993
27	1	0	-3.838202	3.143957	0.578223
28	6	0	-2.678193	2.853996	-1.231463
29	1	0	-3.549991	2.571819	-1.829681
30	6	0	-1.990678	1.572904	-0.727961
31	1	0	-1.690419	0.939903	-1.565258
32	1	0	-2.692834	1.007143	-0.105960
33	6	0	-1.203447	2.805024	1.308262
34	1	0	-0.341344	3.065081	1.926693
35	1	0	-1.891167	2.225731	1.937484
36	6	0	-1.697586	3.659309	-2.096824
37	1	0	-2.188641	4.561578	-2.479467
38	1	0	-1.385509	3.060879	-2.959380
39	6	0	1.489235	-0.091616	-0.230497
40	6	0	1.276054	-0.366763	-1.624256
41	6	0	2.453343	-0.773180	-2.342846
42	1	0	2.337429	-0.983167	-3.400767
43	6	0	3.683551	-0.898496	-1.738725
44	1	0	4.544991	-1.210465	-2.319360
45	6	0	3.827862	-0.625546	-0.372782
46	6	0	2.742283	-0.221651	0.378157
47	1	0	2.842896	0.004245	1.434043
48	8	0	0.143443	-0.253659	-2.175955
49	35	0	5.547319	-0.816309	0.446597

50	35	0	-2.963528	-3.813400	-1.141072

OMe

Standard orientation:

Center Number	Atomic Number	Atomic Type	Coordinates (Angstroms) X	Y	Z
1	15	0	-0.185375	-0.171550	0.830852
2	8	0	-0.376011	-0.470323	2.319146
3	8	0	1.109684	1.523612	3.036593
4	1	0	0.578452	0.684839	2.993521
5	6	0	0.371713	1.564051	0.718897
6	6	0	0.230450	2.296182	-0.457949
7	1	0	-0.200477	1.823957	-1.337313
8	6	0	0.663271	3.617884	-0.531938
9	6	0	1.247451	4.212458	0.586175
10	1	0	1.595868	5.236949	0.559114
11	6	0	1.383328	3.487051	1.765219
12	1	0	1.824291	3.944204	2.643761
13	6	0	0.948143	2.167177	1.856512
14	6	0	1.187647	-1.224096	0.148984
15	6	0	0.606353	-2.539106	-0.410877
16	1	0	-0.000656	-3.036802	0.354796
17	1	0	-0.041681	-2.308704	-1.261014
18	6	0	1.751759	-3.463711	-0.854613
19	1	0	1.325776	-4.391883	-1.248450
20	6	0	2.659318	-3.777174	0.343465
21	1	0	2.083843	-4.285844	1.124881
22	1	0	3.466015	-4.452814	0.036605

23	6	0	3.251782	-2.471323	0.894058
24	1	0	3.891088	-2.691593	1.754953
25	6	0	4.074563	-1.781607	-0.203181
26	1	0	4.898319	-2.431753	-0.520038
27	1	0	4.515319	-0.857505	0.187333
28	6	0	3.161926	-1.461973	-1.395287
29	1	0	3.738583	-0.957728	-2.177134
30	6	0	2.027600	-0.527794	-0.942694
31	1	0	1.379301	-0.263988	-1.780548
32	1	0	2.457636	0.396233	-0.541737
33	6	0	2.112231	-1.546770	1.345804
34	1	0	1.534712	-2.023656	2.141149
35	1	0	2.524309	-0.617653	1.758758
36	6	0	2.569376	-2.764737	-1.951360
37	1	0	3.373007	-3.424907	-2.298526
38	1	0	1.927731	-2.543739	-2.811278
39	6	0	-1.744278	-0.393010	-0.035468
40	6	0	-1.820660	-0.318314	-1.466347
41	6	0	-3.134210	-0.530459	-1.991150
42	1	0	-3.245683	-0.486536	-3.069846
43	6	0	-4.237713	-0.782589	-1.192420
44	1	0	-5.201361	-0.932335	-1.666262
45	6	0	-4.112936	-0.843569	0.201122
46	6	0	-2.861688	-0.648634	0.764674
47	1	0	-2.751638	-0.701953	1.843086
48	8	0	-0.806942	-0.086271	-2.200102
49	8	0	0.481561	4.244131	-1.731842
50	8	0	-5.152985	-1.092555	1.073967
51	6	0	-6.437785	-1.226166	0.513621
52	1	0	-6.499185	-2.084607	-0.165620

53	1	0	-7.120591	-1.388333	1.347281
54	1	0	-6.741845	-0.320709	-0.025044
55	6	0	0.862754	5.601469	-1.820614
56	1	0	1.937178	5.731005	-1.650564
57	1	0	0.622570	5.917645	-2.834517
58	1	0	0.304917	6.221278	-1.110016

***t*Bu**

Standard orientation:

Center Number	Atomic Number	Atomic Type	Coordinates (Angstroms) X	Y	Z
1	15	0	0.140210	0.447193	0.823630
2	8	0	0.694324	0.817515	2.202128
3	8	0	-1.371819	-0.196441	3.408433
4	1	0	-0.549322	0.305896	3.164988
5	6	0	-1.079714	-0.876878	1.098548
6	6	0	-1.433185	-1.762170	0.082138
7	1	0	-0.956745	-1.627569	-0.884573
8	6	0	-2.382464	-2.769199	0.266207
9	6	0	-2.981843	-2.859154	1.525451
10	1	0	-3.730783	-3.617253	1.722624
11	6	0	-2.635585	-1.997339	2.559915
12	1	0	-3.095003	-2.087035	3.538029
13	6	0	-1.677088	-1.002389	2.368329
14	6	0	-0.774353	1.899199	0.102413
15	6	0	0.183281	2.711712	-0.792529
16	1	0	1.084441	2.982344	-0.229363
17	1	0	0.488318	2.094181	-1.640965

18	6	0	-0.524758	3.978507	-1.296790
19	1	0	0.166717	4.543098	-1.930046
20	6	0	-0.953827	4.840021	-0.101418
21	1	0	-0.073469	5.136087	0.479919
22	1	0	-1.439013	5.757801	-0.453283
23	6	0	-1.921264	4.041504	0.784967
24	1	0	-2.221978	4.650349	1.643495
25	6	0	-3.158471	3.648394	-0.033355
26	1	0	-3.673361	4.547823	-0.391051
27	1	0	-3.862364	3.094583	0.597807
28	6	0	-2.726253	2.778742	-1.222665
29	1	0	-3.605850	2.486063	-1.804500
30	6	0	-2.027584	1.507680	-0.708080
31	1	0	-1.732900	0.860710	-1.537523
32	1	0	-2.719413	0.948342	-0.068699
33	6	0	-1.218232	2.775359	1.297206
34	1	0	-0.348552	3.045532	1.900662
35	1	0	-1.895899	2.204713	1.944627
36	6	0	-1.761308	3.574780	-2.113625
37	1	0	-2.261545	4.469425	-2.502786
38	1	0	-1.457921	2.964882	-2.971272
39	6	0	1.472570	-0.117386	-0.244171
40	6	0	1.255641	-0.411089	-1.634181
41	6	0	2.438240	-0.810461	-2.335539
42	1	0	2.325952	-1.043395	-3.390015
43	6	0	3.677014	-0.891431	-1.727979
44	1	0	4.521260	-1.197475	-2.337581
45	6	0	3.870828	-0.592677	-0.367682
46	6	0	2.736553	-0.207274	0.344483
47	8	0	0.118472	-0.315003	-2.192404

48	6	0	-2.704292	-3.709106	-0.897363
49	6	0	-3.814525	-4.701922	-0.543395
50	1	0	-4.021339	-5.342464	-1.405173
51	1	0	-3.528100	-5.348806	0.290683
52	1	0	-4.743084	-4.188762	-0.277129
53	6	0	-1.439324	-4.500524	-1.269453
54	1	0	-0.626646	-3.832890	-1.566689
55	1	0	-1.093279	-5.098472	-0.421608
56	1	0	-1.647005	-5.175746	-2.105693
57	6	0	-3.153671	-2.884839	-2.115006
58	1	0	-4.045941	-2.297361	-1.879252
59	1	0	-2.371016	-2.196256	-2.443196
60	1	0	-3.389983	-3.548479	-2.952800
61	1	0	2.815407	0.046645	1.397933
62	6	0	5.229064	-0.667263	0.336297
63	6	0	5.158673	-1.667238	1.502420
64	1	0	6.124360	-1.729617	2.015512
65	1	0	4.405987	-1.368061	2.236151
66	1	0	4.895822	-2.665388	1.140962
67	6	0	5.605153	0.716079	0.892587
68	1	0	6.571478	0.674157	1.406709
69	1	0	5.677057	1.451141	0.086023
70	1	0	4.859426	1.074042	1.606807
71	6	0	6.343435	-1.116687	-0.613808
72	1	0	6.465354	-0.420963	-1.448799
73	1	0	7.293064	-1.157541	-0.072106
74	1	0	6.148650	-2.112456	-1.022055

I

Standard orientation:

Center Number	Atomic Number	Atomic Type	Coordinates (Angstroms) X	Y	Z
1	15	0	0.181814	0.827326	0.812683
2	8	0	0.901629	1.193030	2.111955
3	8	0	-1.035046	0.383543	3.591406
4	1	0	-0.209796	0.804617	3.219742
5	6	0	-1.170835	-0.307582	1.273777
6	6	0	-1.801342	-1.112776	0.318252
7	1	0	-1.463977	-1.061282	-0.713001
8	6	0	-2.850972	-1.931427	0.695243
9	6	0	-3.289486	-1.974855	2.016379
10	1	0	-4.113798	-2.615451	2.305754
11	6	0	-2.659105	-1.191301	2.967791
12	1	0	-2.973513	-1.215714	4.004781
13	6	0	-1.595037	-0.351853	2.616246
14	6	0	-0.605230	2.339467	0.075961
15	6	0	0.408273	3.025571	-0.862057
16	1	0	1.343118	3.222622	-0.323924
17	1	0	0.634867	2.357360	-1.697881
18	6	0	-0.186604	4.341008	-1.388416
19	1	0	0.541956	4.819218	-2.050347
20	6	0	-0.509419	5.274192	-0.211831
21	1	0	0.405648	5.505180	0.344494
22	1	0	-0.914299	6.221480	-0.586294
23	6	0	-1.530013	4.598339	0.715804
24	1	0	-1.753883	5.256179	1.561558
25	6	0	-2.815886	4.303993	-0.069321
26	1	0	-3.246291	5.239433	-0.445016

27	1	0	-3.558880	3.840648	0.589412
28	6	0	-2.491938	3.363345	-1.236898
29	1	0	-3.406248	3.136739	-1.793879
30	6	0	-1.911497	2.048967	-0.691181
31	1	0	-1.707742	1.348662	-1.502105
32	1	0	-2.638639	1.586956	-0.014719
33	6	0	-0.932614	3.287381	1.253341
34	1	0	-0.025355	3.495991	1.825424
35	1	0	-1.642883	2.801906	1.934726
36	6	0	-1.472705	4.033312	-2.169660
37	1	0	-1.892249	4.959406	-2.579248
38	1	0	-1.247148	3.370790	-3.012337
39	6	0	1.333417	0.014190	-0.305545
40	6	0	0.972030	-0.329655	-1.654826
41	6	0	2.020033	-0.955150	-2.416019
42	1	0	1.787899	-1.224032	-3.441384
43	6	0	3.268415	-1.210952	-1.895329
44	1	0	4.023223	-1.686475	-2.512413
45	6	0	3.561554	-0.857208	-0.571897
46	6	0	2.605816	-0.245653	0.215237
47	1	0	2.822884	0.046053	1.236600
48	8	0	-0.172937	-0.101258	-2.139393
49	53	0	5.505779	-1.242687	0.229989
50	53	0	-3.828843	-3.123177	-0.779089

----

cHex

Standard orientation:

Center	Atomic	Atomic	Coordinates (Angstroms)		
Number	Number	Type	X	Y	Z

1	15	0	0.207052	0.951259	0.823834
2	8	0	0.922790	1.359303	2.115581
3	8	0	-1.035227	0.606808	3.607486
4	1	0	-0.206936	1.011717	3.227745
5	6	0	-1.146960	-0.166215	1.311131
6	6	0	-1.773513	-1.005473	0.381443
7	1	0	-1.421777	-0.981781	-0.647694
8	6	0	-2.837215	-1.828631	0.734954
9	6	0	-3.265820	-1.802992	2.069260
10	1	0	-4.095666	-2.430581	2.380588
11	6	0	-2.653112	-0.993580	3.009939
12	1	0	-2.979609	-0.983858	4.043932
13	6	0	-1.585374	-0.164139	2.647373
14	6	0	-0.588716	2.448620	0.058851
15	6	0	0.423369	3.131632	-0.882320
16	1	0	1.354001	3.341631	-0.341689
17	1	0	0.658206	2.453663	-1.707980
18	6	0	-0.177658	4.436667	-1.427444
19	1	0	0.550442	4.912921	-2.091630
20	6	0	-0.513798	5.381838	-0.264123
21	1	0	0.396600	5.626424	0.294214
22	1	0	-0.923657	6.321761	-0.651992
23	6	0	-1.533865	4.709497	0.666698
24	1	0	-1.766909	5.376075	1.503231
25	6	0	-2.813407	4.396936	-0.121588
26	1	0	-3.249003	5.324853	-0.510379
27	1	0	-3.556111	3.935585	0.538963
28	6	0	-2.476581	3.444407	-1.276104
29	1	0	-3.386669	3.205748	-1.835477

30	6	0	-1.888991	2.140746	-0.711920
31	1	0	-1.673448	1.431365	-1.512138
32	1	0	-2.615705	1.681039	-0.033391
33	6	0	-0.930298	3.409004	1.221579
34	1	0	-0.027856	3.630096	1.796520
35	1	0	-1.640314	2.926155	1.905194
36	6	0	-1.457553	4.110893	-2.211685
37	1	0	-1.881494	5.029169	-2.634701
38	1	0	-1.222787	3.439769	-3.045024
39	6	0	1.359962	0.118612	-0.274579
40	6	0	1.012971	-0.221330	-1.625870
41	6	0	2.066522	-0.865050	-2.362724
42	1	0	1.847362	-1.128562	-3.393109
43	6	0	3.296106	-1.143554	-1.809286
44	1	0	4.045723	-1.637057	-2.424477
45	6	0	3.611713	-0.811074	-0.476050
46	6	0	2.621904	-0.176499	0.261027
47	1	0	2.821808	0.111892	1.289810
48	8	0	-0.121794	0.022308	-2.136800
49	6	0	4.964509	-1.128746	0.123741
50	6	0	5.241458	-2.640854	0.174648
51	6	0	6.115945	-0.412749	-0.602863
52	1	0	4.956237	-0.763690	1.159543
53	6	0	6.595268	-2.949571	0.818794
54	1	0	5.228896	-3.039384	-0.848122
55	1	0	4.434429	-3.140035	0.719185
56	6	0	7.470737	-0.717876	0.040916
57	1	0	6.132008	-0.737788	-1.651150
58	1	0	5.928180	0.665043	-0.607978
59	6	0	7.731774	-2.224510	0.094583

60	1	0	6.774396	-4.028872	0.823035
61	1	0	6.574747	-2.626105	1.867096
62	1	0	8.272012	-0.211425	-0.505598
63	1	0	7.478777	-0.316818	1.062143
64	1	0	8.687922	-2.427914	0.586105
65	1	0	7.809176	-2.612209	-0.928981
66	6	0	-3.526246	-2.699939	-0.293649
67	6	0	-3.427928	-4.196022	0.045717
68	6	0	-4.998046	-2.295576	-0.484407
69	1	0	-3.014727	-2.544298	-1.251833
70	6	0	-4.121277	-5.059020	-1.011158
71	1	0	-3.897222	-4.374269	1.021612
72	1	0	-2.375781	-4.479220	0.141685
73	6	0	-5.691489	-3.160914	-1.538450
74	1	0	-5.523440	-2.402616	0.473350
75	1	0	-5.050964	-1.238111	-0.760898
76	6	0	-5.583654	-4.646660	-1.190509
77	1	0	-4.054233	-6.115532	-0.735307
78	1	0	-3.596541	-4.944667	-1.967717
79	1	0	-6.740761	-2.866484	-1.634302
80	1	0	-5.220279	-2.985164	-2.513126
81	1	0	-6.058425	-5.254596	-1.966118
82	1	0	-6.126949	-4.837371	-0.256311

----

3. Computation studies

Unless noted, all energetics are reported in kcal/mol, and the bond lengths are reported in angstroms (Å). Structures were generated using CYLview.[9] Due to the large catalysis system, to reduce the computation time, transition states towards product **3k** were explored and benzyl-substituted MBH carbonates were simplified to methyl-substituted

ones. All transition states were optimized using the ONIOM method implemented in Gaussian09 [1] at gas phase. M06-2X functional [2] with 6-31G(d) basis set was used for the high-layer and PM6 method for the low-layer. Quinuclidine ring moiety of the catalyst, which is far from reaction center, was treated as the low layer. The rest of the system was treated as the high layer. The frequency calculations were conducted at the same level of theory to confirm the nature of stationary points and obtain the thermal corrections. The high-level solution-phase energies of the transition states were calculated with SMD method [3] in chloroform. M06-2X functional [2] with 6-311+(d,p) basis set was used for the high-layer and PM6 method for the low-layer. Intermolecular non-covalent interactions (NCI) in transition states were analyzed by Multiwfn [10] using Independent Gradient Model (IGM). [11] The corresponding NCI pictures were generated using VMD. [12]

NCI analysis indicated that the interaction between *tert*-butyl and catalyst is favored. However, if the *tert*-butyl group linked to phosphorous atom is replaced by adamantyl group, the steric effect between catalyst and substrate will be more remarkable. The steric effect destabilizes **TS2**, which is crucial for excellent enantioselectivity.

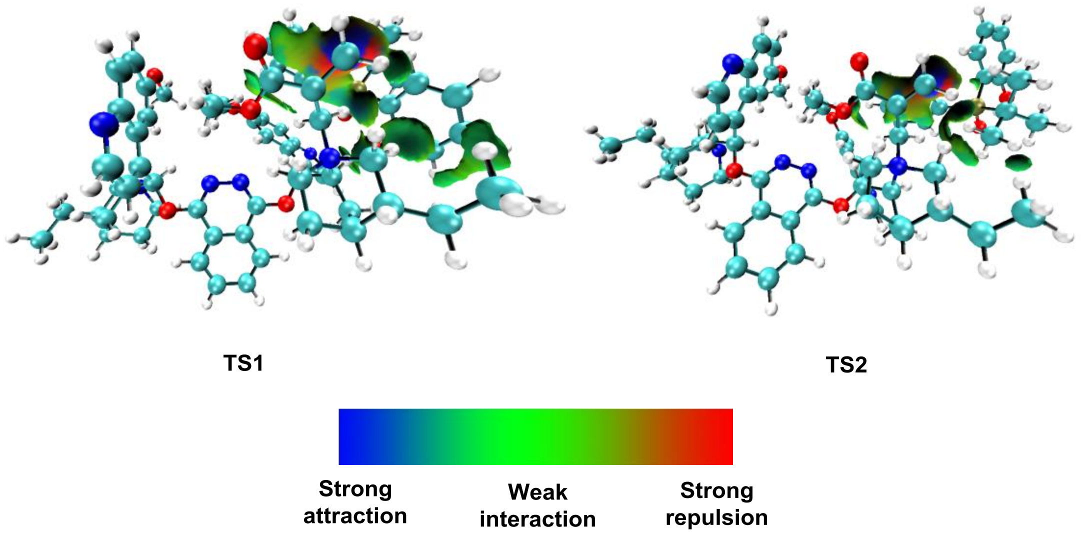

Fig. 4 IGM analysis of intermolecular interaction in transition states.

Calculated Cartesian coordinates and energies:

TS1

Imaginary Frequency: -282.9106

ONIOM(M06-2X/6-311+g(d,p):pm6)(smd)//ONIOM(M06-2X/6-31g(d):pm6) Free energy: -3615.421253

N 0 -2.53451700 0.43660800 -4.73116000 H

N 0 -2.47127800 1.59274000 1.46214300 H

N	0	1.27686800	1.19853500	-0.18916500	H
N	0	2.57218000	0.84515400	0.03408900	H
N	0	3.97589700	-1.36901000	4.19411000	H
N	0	6.77288500	-0.43994600	-1.56210800	L
O	0	0.78053000	-2.98120400	-1.95216900	H
O	0	-0.30590000	2.72900600	-0.84846400	H
O	0	4.79757000	1.36884600	0.04207400	H
O	0	3.75137800	-5.11209400	0.14968800	H
C	0	-0.01050600	-2.06478500	-2.56213700	H
C	0	-0.46372500	-2.42274800	-3.86078100	H
H	0	-0.13799200	-3.37356100	-4.27084800	H
C	0	-1.29308800	-1.58404800	-4.54803700	H
H	0	-1.66288700	-1.82960600	-5.53796900	H
C	0	-1.71528200	-0.34684200	-3.98460600	H
C	0	-1.25413400	0.00974900	-2.68465900	H
C	0	-0.38932900	-0.87579900	-1.98777700	H
H	0	-0.00668800	-0.61524600	-1.01040000	H
C	0	-1.71327800	1.25303100	-2.15762200	H
C	0	-2.54522200	2.02589500	-2.92938600	H
H	0	-2.94420200	2.96641400	-2.55912100	H
C	0	-2.92876000	1.57415900	-4.21187800	H
H	0	-3.59349300	2.18551300	-4.81879600	H
C	0	-1.31002200	1.69720500	-0.76740800	H
H	0	-0.85314400	0.85994600	-0.25331200	H
C	0	-2.51204300	2.19906700	0.06695300	H
H	0	-3.41589500	1.78204000	-0.38470200	H
C	0	-2.64605800	3.73088500	0.21105300	H
H	0	-1.98018900	4.22542000	-0.49886100	H
H	0	-3.67256100	4.02457000	-0.03372600	H
C	0	-2.29305500	4.14762200	1.64899300	H

H	0	-2.21040700	5.23735200	1.71470300	H
C	0	-0.97311600	3.48334500	2.06477900	H
H	0	-0.65873500	3.85881900	3.04318600	H
H	0	-0.18162700	3.72535900	1.35089800	H
C	0	-1.18297200	1.95612000	2.14949200	H
H	0	-0.37909400	1.38224400	1.68613100	H
H	0	-1.29064100	1.59351600	3.17485700	H
C	0	-3.61786500	2.15744700	2.26428200	H
H	0	-3.70539200	1.52192500	3.14709300	H
H	0	-4.51560500	2.03054300	1.64705000	H
C	0	-3.36943100	3.63484800	2.61609100	H
H	0	-2.95210200	3.68960800	3.63189300	H
C	0	-4.67415200	4.43131200	2.61614000	H
H	0	-5.16114300	4.34341900	1.63661400	H
H	0	-4.43883700	5.49237500	2.76464900	H
C	0	-5.63286300	3.95985800	3.70877200	H
H	0	-5.90024300	2.90587000	3.57596000	H
H	0	-6.56028600	4.53950200	3.69790400	H
H	0	-5.17841600	4.07172800	4.69903500	H
C	0	0.98164000	2.38170100	-0.62336700	H
C	0	1.95617800	3.41349600	-0.86937700	H
C	0	1.64144200	4.70216700	-1.33668500	H
H	0	0.60613500	4.95944400	-1.53188700	H
C	0	2.66119000	5.60563400	-1.55058100	H
H	0	2.43157300	6.60262100	-1.91275400	H
C	0	4.00368600	5.24537200	-1.31820500	H
H	0	4.78974000	5.96949000	-1.50597100	H
C	0	4.32543600	3.98364000	-0.86440100	H
H	0	5.35682100	3.67870800	-0.70015200	H
C	0	3.29266900	3.05905600	-0.62672500	H

C	0	3.51504400	1.71233600	-0.16591000	H
C	0	5.06044000	-0.03891300	0.20354600	H
H	0	4.44184700	-0.56959900	-0.53706900	H
C	0	4.69143900	-0.51080200	1.59909900	H
C	0	4.66862700	0.35468700	2.66276700	H
H	0	4.90708300	1.40386600	2.52311800	H
C	0	4.30758600	-0.12534800	3.94275100	H
H	0	4.29066900	0.56054600	4.78761400	H
C	0	3.98460100	-2.24172500	3.15103000	H
C	0	4.34670500	-1.87394400	1.82617900	H
C	0	4.30605200	-2.84842200	0.79294900	H
H	0	4.58646500	-2.57457600	-0.21832900	H
C	0	3.88388900	-4.12776700	1.07041300	H
C	0	3.52087100	-4.49882700	2.39458900	H
H	0	3.19407400	-5.51942600	2.56328600	H
C	0	3.57684600	-3.58157100	3.40366900	H
H	0	3.29621700	-3.83441500	4.42056100	H
C	0	6.55823600	-0.25675900	-0.08023300	L H 64 0.0000
H	0	6.83743200	-1.23449800	0.40312500	L
C	0	7.46665700	0.86553700	0.48304000	L
H	0	6.86456100	1.67787200	0.92900200	L
H	0	8.09933700	0.47251900	1.29770700	L
C	0	8.34201800	1.42271300	-0.66061400	L
H	0	9.01168300	2.21785100	-0.27138100	L
C	0	7.40441900	1.99664300	-1.74811500	L
H	0	6.76629500	2.79308600	-1.31648100	L
H	0	7.98591200	2.46569300	-2.55777100	L
C	0	6.52286100	0.84829100	-2.29363800	L
H	0	5.44688400	1.10111900	-2.19926700	L
H	0	6.71354600	0.67527000	-3.37179100	L

C	0	8.21022900	-0.83161400	-1.76895000	L
H	0	8.35430500	-1.04722500	-2.84679100	L
H	0	8.39103100	-1.78773500	-1.23804000	L
C	0	9.18743900	0.28491000	-1.29902500	L
H	0	9.70339200	0.70255800	-2.19620200	L
C	0	10.25871400	-0.27118600	-0.34702900	L
H	0	9.79529800	-0.57621000	0.61217700	L
H	0	10.68776300	-1.19746200	-0.78006200	L
C	0	11.38393800	0.72611300	-0.08251500	L
H	0	11.88879900	1.02334400	-1.00884600	L
H	0	12.14440900	0.29529000	0.57939700	L
H	0	11.01359800	1.63920700	0.39738500	L
C	0	1.08260700	-2.74891000	-0.58480200	H
H	0	1.77155000	-1.89964800	-0.46860400	H
H	0	0.16695600	-2.55816300	-0.01187800	H
H	0	1.55855000	-3.66225500	-0.22168300	H
C	0	4.07997000	-4.80446500	-1.19174000	H
H	0	3.43197500	-4.01297200	-1.58933400	H
H	0	5.12915900	-4.49602000	-1.27869200	H
H	0	3.91968900	-5.72122400	-1.75742500	H
C	0	-2.69834700	0.07056000	1.34374300	H
H	0	-1.99162600	-0.30123100	0.60269800	H
H	0	-3.70220600	0.00668600	0.92116500	H
C	0	-2.62328000	-0.72679400	2.59467000	H
C	0	-3.80394700	-1.14513800	3.17095100	H
H	0	-3.75144600	-1.84791500	3.99660600	H
H	0	-4.69419400	-0.52621100	3.12425000	H
C	0	-1.37529600	-1.40209300	2.93710000	H
O	0	-1.20175200	-2.20701700	3.82821200	H
O	0	-0.34123800	-0.97139800	2.14708300	H

C	0	0.94473800	-1.48697400	2.48052500	H
H	0	1.16002500	-1.33651200	3.54187500	H
H	0	1.00224300	-2.55978800	2.26636500	H
P	0	-5.26357900	-1.27517600	-1.12995600	H
O	0	-4.68271100	-0.65707300	-2.40342700	H
O	0	-5.34278900	1.71973800	-1.87489700	H
H	0	-4.99551700	0.86008000	-2.28717100	H
C	0	-5.90453300	0.12020800	-0.11871600	H
C	0	-6.53276000	-0.05349700	1.12637400	H
H	0	-6.46969000	-1.02973900	1.59571800	H
C	0	-7.17388700	0.99619300	1.77387200	H
H	0	-7.65140100	0.83015800	2.73448100	H
C	0	-7.21224500	2.25528200	1.17006800	H
H	0	-7.72586400	3.08042200	1.65719000	H
C	0	-6.59081300	2.46230800	-0.05040400	H
H	0	-6.61083400	3.42834700	-0.54658600	H
C	0	-5.93377300	1.40941200	-0.71186300	H
C	0	-4.03085600	-2.36278700	-0.37883900	H
C	0	-3.99609200	-2.76026500	0.98823600	H
C	0	-2.95030300	-3.63917000	1.36988100	H
H	0	-2.87242000	-3.89306300	2.42313900	H
C	0	-2.06699800	-4.16339600	0.44339300	H
H	0	-1.29406500	-4.85332000	0.77558000	H
C	0	-2.14643200	-3.80475700	-0.90654300	H
H	0	-1.44492000	-4.20944100	-1.62881700	H
C	0	-3.10915000	-2.88648900	-1.30163000	H
H	0	-3.15077300	-2.54961600	-2.33629500	H
O	0	-4.82118500	-2.28638900	1.87512100	H
C	0	-6.74437600	-2.30007700	-1.54996300	H
C	0	-7.77511300	-1.38527600	-2.22217700	H

H 0 -8.61791000 -1.98941500 -2.57828400 H

H 0 -8.16707400 -0.63838900 -1.52346500 H

H 0 -7.33657900 -0.86550700 -3.08039300 H

C 0 -6.26230900 -3.35690900 -2.55513900 H

H 0 -5.56488700 -4.06129100 -2.09147500 H

H 0 -7.12884100 -3.92213900 -2.91766800 H

H 0 -5.76840300 -2.88947600 -3.41286800 H

C 0 -7.34392600 -2.98414900 -0.31771500 H

H 0 -6.58494600 -3.50868900 0.27192700 H

H 0 -7.84889500 -2.26641800 0.33510200 H

H 0 -8.09236800 -3.71483200 -0.64780800 H

H 0 1.65663700 -0.93510300 1.86049600 H

TS 2

Imaginary Frequency: -163.1994

ONIOM(M06-2X/6-311+g(d,p):pm6)(smd)//ONIOM(M06-2X/6-31g(d):pm6) Free energy: -3615.417762

N 0 1.00894700 1.79360500 5.16758700 H

N 0 1.98634100 2.29400100 -1.05928500 H

N 0 -1.99972400 1.28999000 0.32423600 H

N 0 -3.20182000 0.69170600 0.09926500 H

N 0 -3.79251400 -1.97994000 -4.04550700 H

N 0 -7.18935200 -1.28067100 1.37646200 L

O 0 -0.56022100 -2.68538600 2.37021600 H

O 0 -0.72096900 3.16617900 0.64860400 H

O 0 -5.46060600 0.83234700 -0.22642200 H

O 0 -3.08336300 -5.25920900 0.33112800 H

C 0 -0.16303600 -1.53123100 2.95548900 H

C 0 0.13152200 -1.63428600 4.34362000 H

H 0 0.02605400 -2.60633500 4.81399500 H

C 0 0.52019500 -0.53056800 5.04350800 H

H	0	0.73978900	-0.57283400	6.10491100	H
C	0	0.64820200	0.73338100	4.40044200	H
C	0	0.38714200	0.82440200	3.00114700	H
C	0	-0.03090400	-0.33436700	2.29233300	H
H	0	-0.28668600	-0.26978500	1.24243700	H
C	0	0.56704000	2.10427800	2.40044400	H
C	0	0.92227000	3.16517600	3.20070300	H
H	0	1.05671300	4.15964200	2.78310700	H
C	0	1.12532500	2.96205900	4.58211000	H
H	0	1.40665900	3.80046500	5.21587400	H
C	0	0.40272900	2.31017600	0.90775800	H
H	0	0.20160900	1.35634200	0.42677000	H
C	0	1.66415300	2.94822600	0.27609700	H
H	0	2.51477800	2.68521300	0.91138200	H
C	0	1.58964200	4.47934300	0.05707300	H
H	0	0.72561200	4.88236100	0.58759000	H
H	0	2.48284700	4.95532800	0.47203500	H
C	0	1.47106200	4.78695200	-1.44512700	H
H	0	1.24546400	5.84812200	-1.59093800	H
C	0	0.37270100	3.91463900	-2.06289200	H
H	0	0.22372400	4.19332100	-3.11027500	H
H	0	-0.57528800	4.06365600	-1.54165700	H
C	0	0.80559800	2.43486900	-1.98905000	H
H	0	0.01938000	1.77099900	-1.62652600	H
H	0	1.14638400	2.04475300	-2.95029800	H
C	0	3.16694000	3.01232900	-1.66738800	H
H	0	3.53107800	2.37057200	-2.47240000	H
H	0	3.93429100	3.04887300	-0.88535700	H
C	0	2.78391600	4.42113500	-2.14955200	H
H	0	2.58380300	4.38068800	-3.22936000	H

C	0	3.92486400	5.41636400	-1.93304600	H
H	0	4.18671800	5.45687500	-0.86759500	H
H	0	3.56906200	6.41654100	-2.20786300	H
C	0	5.16606100	5.06988200	-2.75385800	H
H	0	5.56781500	4.08876900	-2.48044100	H
H	0	5.95930500	5.80466700	-2.59667300	H
H	0	4.93153200	5.04949200	-3.82323900	H
C	0	-1.92099500	2.57499200	0.43586400	H
C	0	-3.04260300	3.46877300	0.31560800	H
C	0	-2.95440000	4.86828200	0.42556500	H
H	0	-1.99244200	5.32837200	0.62464700	H
C	0	-4.09766700	5.62658900	0.28648300	H
H	0	-4.04260800	6.70713000	0.37086100	H
C	0	-5.34200400	5.01253400	0.04300900	H
H	0	-6.22983500	5.62813900	-0.05840700	H
C	0	-5.44212300	3.64102500	-0.06130800	H
H	0	-6.39335900	3.15352600	-0.24225800	H
C	0	-4.28283000	2.85696900	0.07384900	H
C	0	-4.27097800	1.41785100	-0.01514600	H
C	0	-5.45241900	-0.61037200	-0.26944500	H
H	0	-4.82333500	-0.95497000	0.56447400	H
C	0	-4.86980900	-1.09875800	-1.58610500	H
C	0	-4.95578000	-0.33780100	-2.72485000	H
H	0	-5.43190100	0.63711400	-2.70031700	H
C	0	-4.40375600	-0.82492800	-3.93195500	H
H	0	-4.47306300	-0.22267200	-4.83582200	H
C	0	-3.68986100	-2.74657400	-2.92705500	H
C	0	-4.21888400	-2.36317900	-1.66364600	H
C	0	-4.04668200	-3.21982300	-0.54292400	H
H	0	-4.45783000	-2.93687800	0.41997900	H

C	0	-3.34698000	-4.39642800	-0.67889500	H
C	0	-2.81384600	-4.78102600	-1.94053800	H
H	0	-2.26741000	-5.71650300	-1.99556800	H
C	0	-2.98861100	-3.98083000	-3.03176600	H
H	0	-2.58519100	-4.24523600	-4.00331200	H
C	0	-6.90128700	-1.09756800	-0.09268500	L H 64 0.0000
H	0	-6.94932000	-2.12339400	-0.55668600	L
C	0	-7.96697900	-0.19491200	-0.76179500	L
H	0	-7.52489300	0.75946200	-1.10047200	L
H	0	-8.36957300	-0.68303000	-1.66598300	L
C	0	-9.09623300	0.07884900	0.25658500	L
H	0	-9.90885000	0.65923000	-0.22805600	L
C	0	-8.49766300	0.87709000	1.43813000	L
H	0	-8.13558500	1.85938600	1.09277500	L
H	0	-9.27121400	1.08496900	2.19619400	L
C	0	-7.33756800	0.05506400	2.04779300	L
H	0	-6.37752800	0.60335700	1.95159000	L
H	0	-7.49745300	-0.12135100	3.13023400	L
C	0	-8.49047400	-2.02559000	1.49357000	L
H	0	-8.69888200	-2.18692600	2.57041000	L
H	0	-8.35298800	-3.03385300	1.05371200	L
C	0	-9.66325000	-1.25450600	0.82039200	L
H	0	-10.41578600	-1.00225300	1.60521100	L
C	0	-10.36021900	-2.11218000	-0.24826400	L
H	0	-9.67444200	-2.29680100	-1.09842900	L
H	0	-10.58533000	-3.11178300	0.17617000	L
C	0	-11.65411300	-1.48141200	-0.75665700	L
H	0	-12.36874900	-1.30925800	0.05639700	L
H	0	-12.14641300	-2.13049900	-1.49060500	L
H	0	-11.47276700	-0.51711200	-1.24466600	L

C	0	-0.90300300	-2.62070700	0.99561400	H
H	0	-1.76712000	-1.95907000	0.84016100	H
H	0	-0.06043800	-2.25554400	0.39602500	H
H	0	-1.15251200	-3.64052100	0.69839400	H
C	0	-3.58589400	-4.94133800	1.61480900	H
H	0	-3.15106600	-4.00715600	1.99258000	H
H	0	-4.67916500	-4.85365900	1.59744600	H
H	0	-3.29357000	-5.76505700	2.26421800	H
C	0	2.41840400	0.81878000	-0.82588000	H
H	0	1.75481800	0.40717000	-0.06610800	H
H	0	3.41394800	0.89629200	-0.37769000	H
C	0	2.42479100	-0.04283000	-2.04049000	H
C	0	3.56290100	-0.31023400	-2.74561300	H
H	0	3.50933000	-1.00949800	-3.57149200	H
H	0	4.43337800	0.33145700	-2.70779500	H
C	0	1.23682300	-0.85403000	-2.34262700	H
O	0	1.15005900	-1.69214900	-3.21059100	H
O	0	0.16700200	-0.53448900	-1.55102200	H
C	0	-1.05299000	-1.19110200	-1.91183400	H
H	0	-1.29178700	-0.99940500	-2.96158600	H
H	0	-0.96201100	-2.27319600	-1.77631800	H
P	0	7.02362200	-0.69214400	0.55736700	H
O	0	7.48643000	-0.10960100	1.88670800	H
C	0	5.26192300	-1.10281300	0.66176500	H
C	0	4.48362300	-1.62214500	-0.42184200	H
C	0	3.21267800	-2.17005600	-0.07858900	H
H	0	2.66129800	-2.67019500	-0.87113300	H
C	0	2.72432100	-2.11651200	1.21817100	H
H	0	1.77433700	-2.58951900	1.45494400	H
C	0	3.45119500	-1.49741200	2.23911300	H

H	0	3.04881900	-1.43775300	3.24629700	H
C	0	4.73065800	-1.03123800	1.95586100	H
H	0	5.36037300	-0.62420900	2.74266400	H
O	0	4.88357200	-1.59045600	-1.65031700	H
H	0	-1.82195500	-0.77934800	-1.25647500	H
C	0	7.43786700	0.52440400	-0.78782300	H
C	0	7.57863200	-0.10187200	-2.17790300	H
H	0	7.76383100	0.69659600	-2.90900100	H
H	0	8.43026000	-0.78842200	-2.20878800	H
H	0	6.67898700	-0.65429000	-2.45821500	H
C	0	6.33776800	1.58634200	-0.78910500	H
H	0	6.61402800	2.40571600	-1.46647400	H
H	0	5.40111700	1.14017600	-1.13650800	H
H	0	6.18083500	2.00303400	0.21404100	H
C	0	8.77609200	1.15534500	-0.37577300	H
H	0	9.55844600	0.39312200	-0.28315400	H
H	0	9.08950700	1.86939900	-1.14765900	H
H	0	8.69500400	1.67086600	0.58374700	H
C	0	7.97107900	-2.19417700	0.19722600	H
C	0	9.18138300	-2.41823300	0.88760400	H
C	0	7.54095900	-3.12109500	-0.76314400	H
C	0	9.94077500	-3.55624700	0.58064600	H
C	0	8.29859300	-4.24593500	-1.05558000	H
H	0	6.60615000	-2.93814200	-1.28894500	H
C	0	9.50321000	-4.45365100	-0.37950300	H
H	0	10.86866600	-3.70730700	1.12249800	H
H	0	7.95486600	-4.95499200	-1.80135300	H
H	0	10.10533500	-5.33048900	-0.60184100	H
O	0	9.66952800	-1.58723000	1.82732300	H
H	0	8.96829700	-0.91949200	2.05277300	H

Reference:

1 *Gaussian 09,* Revision D.01, M. J. Frisch, G. W. Trucks, H. B. Schlegel, G. E. Scuseria, M. A. Robb, J. R. Cheeseman, G. Scalmani, V. Barone, B. Mennucci, G. A. Petersson, H. Nakatsuji, M. Caricato, X. Li, H. P. Hratchian, A. F. Izmaylov, J. Bloino, G. Zheng, J. L. Sonnenberg, M. Hada, M. Ehara, K. Toyota, R. Fukuda, J. Hasegawa, M. Ishida, T. Nakajima, Y. Honda, O. Kitao, H. Nakai, T. Vreven, J. A. Montgomery, Jr., J. E. Peralta, F. Ogliaro, M. Bearpark, J. J. Heyd, E. Brothers, K. N. Kudin, V. N. Staroverov, R. Kobayashi, J. Normand, K. Raghavachari, A. Rendell, J. C. Burant, S. S. Iyengar, J. Tomasi, M. Cossi, N. Rega, J. M. Millam, M. Klene, J. E. Knox, J. B. Cross, V. Bakken, C. Adamo, J. Jaramillo, R. Gomperts, R. E. Stratmann, O. Yazyev, A. J. Austin, R. Cammi, C. Pomelli, J. W. Ochterski, R. L. Martin, K. Morokuma, V. G. Zakrzewski, G. A. Voth, P. Salvador, J. J. Dannenberg, S. Dapprich, A. D. Daniels, Ö. Farkas, J. B. Foresman, J. V. Ortiz, J. Cioslowski, and D. J. Fox, Gaussian, Inc., Wallingford CT, 2013.

2 (a) Y. Zhao and D. G. Truhlar, *Acc. Chem. Res.*, 2008, **41**, 157; (b) Y. Zhao and D. G. Truhlar, *Theor. Chem. Acc.*, 2008, **120**, 215.

3 T. Yanai, D. P. Tew and N. C. Handy, *Chem. Phys. Lett*., 2004, **393**, 51.

4 A. V. Marenich, C. J. Cramer and D. G. Truhlar, *J. Phys. Chem. B*, 2009, **113**, 6378.

5 Molecular Modeling Pro, ChemSW, Inc., Fairfield , CA.

6 NBO Version 3.1, E. D. Glendening, A. E. Reed, J. Carpenter, E. F. Weinhold.

7 (a) M. Charton, *J. Org. Chem.*, 1976, **41**, 2217; (b) M. Charton, *J. Am. Chem. Soc.*, 1975, **97**, 3691; (c) M. Charton, *J. Am. Chem. Soc.*, 1975, **97**, 1552; (d) M. Charton, *J. Am. Chem. Soc.*, 1969, **91**, 615.

8 MATLAB, version 9.4.0.813654 (R2018a), The MathWorks Inc., Natick, Massachusetts.

9 C. Y. Legault, CYLview, 1.0b; Université de Sherbrooke, 2009 (http://www.cylview.org).

10 T. Lu and F. Chen, *J. Comput. Chem.*, 2012, **33**, 580.

11 C. Lefebvre, G. Rubez, H. Khartabil, J. Boisson, J. Contreras-García and Eric Hénon, *Phys. Chem. Chem. Phys*., 2017, **19**, 17928.

12 W. Humphrey, A. Dalke and K. Schulten, *J. Molec. Graphics*, 1996, **14**, 33.

4. Kinetic resolution research

Some kinetic resolution researches were also detected. As (1) in Scheme 1 showed, the racemic 3d reacted with 20

mmol% $(DHQ)_2PHAL$ for 4 days, and finally we got 51.2:48.8 e.r with the contrary configuration. But when it reacted under -40°C, we acquired 3d in almost quantitative yield with 50:50 e.r. value (showed as (2)).

And next we added Boc protected substrate 2a to racemic 3d under room temperature as (3) showed, and finally we got 3d with 54.5:45.5 e.r. value with the contrary configuration. When the 2a was changed to 2b, the racemic 3d also produced a 53.0:47.0 e.r. value.

It indicated there is a decompose process accompanied by the generating process of the products with the existence of $(DHQ)_2PHAL$, and this phenomenon facilitated the kinetic resolution process. But when the reaction were happened under low temperature, these processes of decompose and kinetic resolution are absolutely not main controlling factors of the e.r. values.

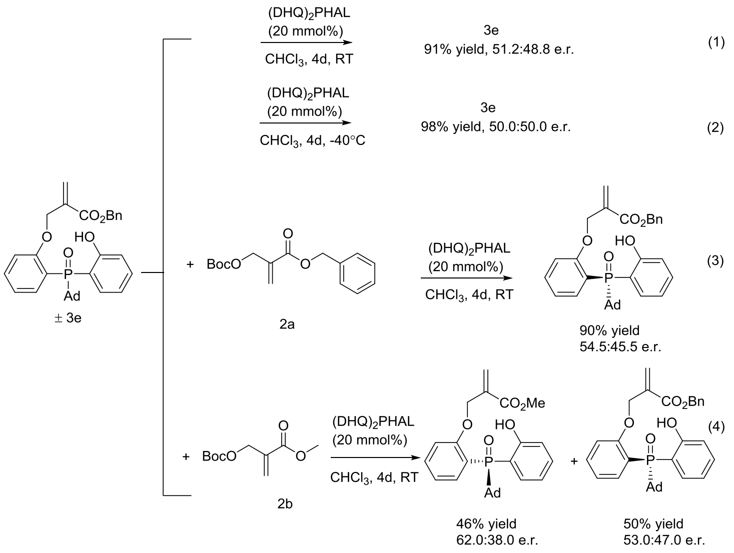

Scheme 1 Kinetic Resolution research

5. Crystal structure data of 3r

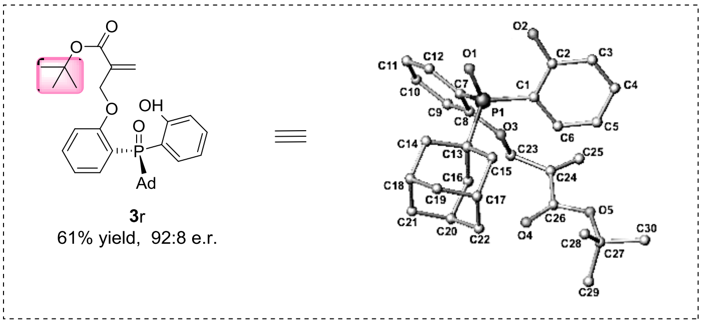

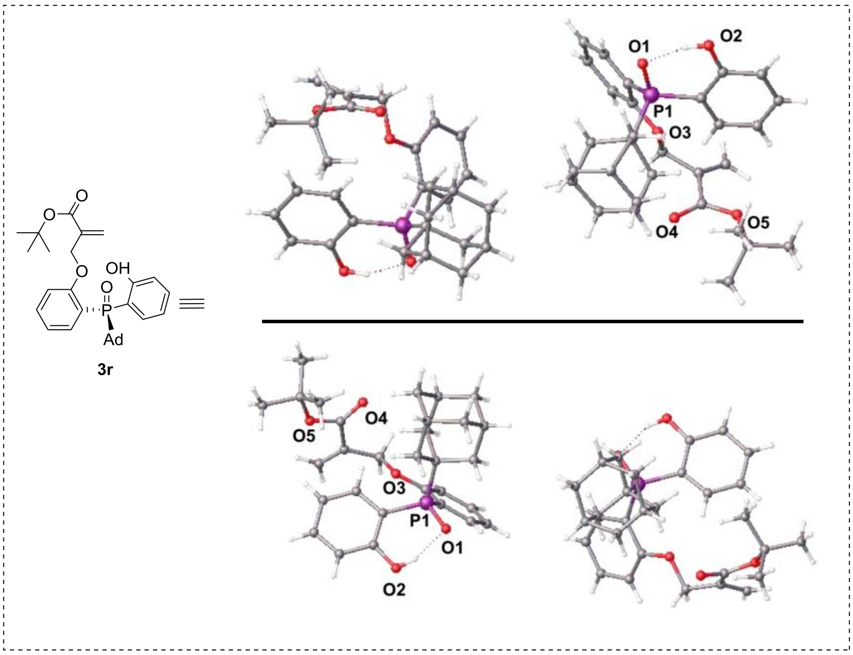

Table 3. Crystal data and structure refinement for 3z:1579041.

Identification code	3z:1579041
Empirical formula	C30 H37 O5 P

Formula weight	508.57
Temperature	133(2) K
Wavelength	0.71073 A
Crystal system, space group	Monoclinic, P2(1)
Unit cell dimensions	a = 12.027(5) A alpha = 90 deg.
	b = 15.901(7) A beta = 91.391(9) deg.
	c = 13.857(6) A gamma = 90 deg.
Volume	2649(2) A^3
Z, Calculated density	4, 1.275 Mg/m^3
Absorption coefficient	0.142 mm^-1
F(000)	1088
Crystal size	0.20 x 0.18 x 0.12 mm
Theta range for data collection	3.07 to 25.02 deg.
Limiting indices	-14<=h<=14, -16<=k<=18, -16<=l<=16
Reflections collected / unique	22390 / 8713 [R(int) = 0.1163]
Completeness to theta = 25.02	99.7 %
Absorption correction	Semi-empirical from equivalents
Max. and min. transmission	0.9832 and 0.9722
Refinement method	Full-matrix least-squares on F^2
Data / restraints / parameters	8713 / 1 / 657
Goodness-of-fit on F^2	1.036
Final R indices [I>2sigma(I)]	R1 = 0.1225, wR2 = 0.3363
R indices (all data)	R1 = 0.1449, wR2 = 0.3641
Absolute structure parameter	0.1(2)
Largest diff. peak and hole	1.760 and -0.520 e.A^-3

6.Characterization of products and novel substrates

6.1 Characterization of products

(R)-Benzyl2-((2-(((3s,5s,7s)-adamantan-1-yl)(2-hydroxyphenyl)phosphoryl)phenoxy)methyl)acrylate(3e)

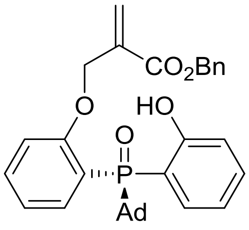

scale (0.1 mmol), product: weight of compound (542.2222), yield (45.5 mg, 0.084 mmol, 84%), colourless oil. **^{1}H NMR** (400 MHz, Chloroform-d) δ 12.05 (s, 1H), 8.16 (ddd, *J* = 12.2, 7.7, 1.8 Hz, 1H), 7.59 (ddd, *J* = 12.3, 7.8, 1.7 Hz, 1H), 7.50 (t, *J* = 7.8 Hz, 1H), 7.45 – 7.30 (m, 6H), 7.15 (t, *J* = 7.0 Hz, 1H), 6.90 (ddd, J = 13.5, 8.4, 4.7 Hz, 2H), 6.76 (td, *J* = 7.7, 2.6 Hz, 1H), 6.44 (s, 1H), 5.78 (s, 1H), 5.26 (d, J = 3.4 Hz, 2H), 4.85 (d, *J* = 3.4 Hz, 2H), 2.05 (m, 9H), 1.85 – 1.70 (m, 6H). **^{13}C NMR** (101 MHz, Chloroform-d) δ 165.05, 164.55 (d, *J* = 2.3Hz), 158.82 (d, *J* = 2.3Hz) 135.48, 135.43 (d, *J* = 1.31 Hz), 134.86, 133.98, 133.29 (d, *J* = 1.8 Hz), 131.97 (d, *J* = 10.6 Hz), 128.76, 128.65, 128.46, 128.24, 121.28 (d, *J* = 10.7 Hz), 118.7 (d, *J* = 86.6 Hz), 118.34 (d, *J* = 7.4 Hz), 117.74 (d, *J* = 11.9 Hz), 112.41 (d, *J* = 6.9 Hz), 110.25 (d, *J* = 93.1 Hz), 66.87, 66.82, 39.10

(d, J = 71.9 Hz), 36.46, 35.25, 27.73, 27.62. ^{31}**P NMR** (162 MHz, Chloroform-d) δ 49.53. **HRMS (ESI)** calculated for $[C_{33}H_{36}O_5P+H]^+$: 543.2295, found: 543.2293. $[\alpha]_D^{20}$= - 4.0 (c = 0.5, $CHCl_3$). **HPLC separation** (Chiralpak AD, *i*-PrOH / hexane = 1 / 4, 1.0 mL/min, 210 nm; tr (minor) = 14.5 min, t_r (major) = 18.6 min, 95 : 5 e.r.).

(R)-Benzyl2-((2-(((3s,5s,7s)-adamantan-1-yl)(2-hydroxy-5-methylphenyl)phosphoryl)-4-methylphenoxy)methyl)acrylate(3f)

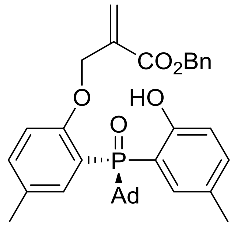

scale (0.1 mmol), product: weight of compound (570.2535), yield (51.9 mg, 0.091 mmol, 91%), colourless oil. ^{1}**H NMR** (400 MHz, Chloroform-d) δ 11.86 (s, 1H), 7.95 (dd, J = 12.5, 2.3 Hz, 1H), 7.36 (m, 6H), 7.24 (dd, J = 8.4, 2.2 Hz, 1H), 7.10 (d, J = 8.4 Hz, 1H), 6.77 (ddd, J = 8.3, 5.0, 3.2 Hz, 2H), 6.39 (s, 1H), 5.73 (s, 1H), 5.23 (s, 2H), 4.85 (s, 2H), 2.33 (s, 3H), 2.19 (s, 3H), 2.13 – 1.92 (m, 9H), 1.70 (m, 6H). ^{13}**C NMR** (101 MHz, Chloroform-d) δ 165.00, 162.25, 156.41 (d, J = 4.2 Hz), 135.88 (d, J = 5.3 Hz), 135.45, 135.07, 134.34, 131.58 (d, J = 10.6Hz), 130.89 (d, J = 10.3Hz), 128.65, 128.47, 128.25, 127.87, 126.66 (d, J = 12.7Hz), 118.58 (d, J = 87.7Hz), 118.11 (d, J = 8.0Hz), 112.45 (d, J = 7.4Hz), 109.96 (d, J = 92.8Hz), 66.90, 66.85, 38.96 (d, J = 70.7 Hz), 36.50, 35.34, 27.79, 27.69, 20.67, 20.53. ^{31}**P NMR** (162 MHz, Chloroform-d) δ 50.01. **HRMS (ESI)** calculated for $[C_{35}H_{40}O_5P+H]^+$: 571.2608, found: 571.2606. $[\alpha]_D^{20}$= - 10.4 (c = 0.5, $CHCl_3$). **HPLC separation** (Chiralpak AD, 4.6 x 250mm; *i*-PrOH / hexane = 1 / 4, 1.0 mL/min, 210 nm; tr (minor) = 8.9 min, tr (major) = 11.0 min, 96 : 4 e.r.).

(R)-Benzyl2-((2-(((3s,5s,7s)-adamantan-1-yl)(5-ethyl-2-hydroxyphenyl)phosphoryl)-4-ethylphenoxy)methyl)acrylate(3g)

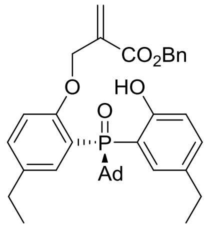

scale (0.1 mmol), product: weight of compound (598.2848), yield (44.9 mg, 0.075 mmol, 75%), colourless oil. ^{1}**H NMR** (400 MHz, Chloroform-d) δ 11.90 (s, 1H), 7.99 (dd, J = 12.5, 2.4 Hz, 1H), 7.49 – 7.35 (m, 6H), 7.30(dd, 1H), 7.18 (d, 1H), 6.82 (m,2H), 6.39 (s, 1H), 5.71 (s, 1H), 5.27 (s, 2H), 4.97 – 4.82 (m, 2H), 2.67 (q, J = 7.6 Hz, 2H), 2.52 (qd, J = 7.5, 2.1 Hz, 2H), 2.18 – 1.96 (m, 9H), 1.74 (m, 6H), 1.25 (t, J = 7.6 Hz, 3H), 1.16 (t, J = 7.6 Hz, 3H). ^{13}**C NMR** (101 MHz, Chloroform-d) δ 165.00，162.47 (d, J = 1.8 Hz), 156.63 (d, J = 3.1 Hz), 137.20 (d, J = 10.0Hz), 135.47, 134.95, 134.81 (d, J = 5.5 Hz), 133.21, 133.10, 130.57 (d, J = 10.2Hz), 128.66, 128.47, 128.22, 127.61, 118.58 (d, J = 87.3Hz), 118.19 (d, J = 7.9 Hz) , 112.54 (d, J = 7.0Hz) , 110.97 (d, J = 92.1Hz), 66.82, 38.98 (d, J = 71.7Hz), 36.53, 35.38, 28.06, 27.95, 27.80, 27.70, 15.9, 15.56. ^{31}**P NMR** (162 MHz, Chloroform-d) δ 50.02. **HRMS (ESI)** calculated for $[C_{37}H_{44}O_5P+H]^+$: 599.2921, found: 599.2926. $[\alpha]_D^{20}$= - 20.2 (c = 0.5, $CHCl_3$). **HPLC separation** (Chiralpak AD, 4.6 x 250mm; *i*-PrOH / hexane = 1 / 4, 1.0 mL/min, 210 nm; tr (minor) = 7.6 min, tr (major) = 8.9 min, 96 : 4 e.r.).

(R)-Benzyl2-((2-(((3s,5s,7s)-adamantan-1-yl)(2-hydroxy-5-isopropylphenyl)phosphoryl)-4-isopropylphenoxy)methyl)acrylate(3h)

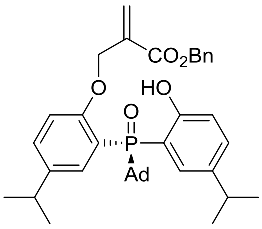

scale (0.1 mmol), product: weight of compound (626.3161), yield (44.5 mg, 0.071 mmol, 71%), colourless oil. **^{1}H NMR** (400 MHz, Chloroform-d) δ 11.888 (s, 1H), 7.99 (dd, *J* = 12.6, 2.4 Hz, 1H), 7.55 – 7.14 (m, 8H), 6.82 (ddd, *J* = 16.9, 8.6, 5.0 Hz, 2H), 6.36 (s, 1H), 5.66 (s, 1H), 5.27 (s, 2H), 4.89 (q, *J* = 15.6 Hz, 1H), 2.95 (p, *J* = 6.9 Hz, 1H), 2.79 (p, *J* = 6.9 Hz, 1H), 2.07 (m, 9 H), 1.81 – 1.70 (m, 6H), 1.26 (dd, *J* = 6.9, 2.3 Hz, 6H), 1.18 (d, *J* = 6.9 Hz, 6H). **^{13}C NMR** (101 MHz, Chloroform-d) δ 164.99, 162.56 (d, *J* = 2.2 Hz), 156.76 (d, *J* = 3.9 Hz), 141.74 (d, *J* = 9.7 Hz), 137.75 (d, *J* = 11.0 Hz), 135.50, 134.81, 133.49 (d, *J* = 5.7 Hz), 133.46 , 131.58, 129.28 (d, *J* = 10.0Hz), 128.66, 128.45, 128.20, 127.41, 118.45 (d, *J* = 87.1 Hz), 118.19 (d, *J* = 8.1 Hz), 112.54 (d, *J* = 7.4 Hz), 109.90 (d, *J* = 92.4 Hz), 66.78, 66.73, 38.97 (d, *J* = 71.7 Hz), 36.55, 35.43, 33.32, 33.27, 27.82, 27.71, 24.53, 24.08, 24.02, 23.87. **^{31}P NMR** (162 MHz, Chloroform-d) δ 50.02. **HRMS (ESI)** calculated for $[C_{39}H_{48}O_5P+H]^+$: 627.3234, found: 627.3239. **$[\alpha]_D^{20}$**= - 8.6 (c = 0.5, $CHCl_3$). **HPLC separation** (Chiralpak AD, 4.6 x 250mm; *i*-PrOH / hexane = 1 / 4, 1.0 mL/min, 210 nm; tr (minor) = 6.0 min, tr (major) = 6.8 min, 97 : 3 e.r.).

(R)- Benzyl 2-((2-((R)-((1s,3R,5R,7S)-adamantan-1-yl)(5-cyclohexyl-2-hydroxyphenyl)phosphoryl)-4-cyclohexylphenoxy)methyl)acrylate (3i)

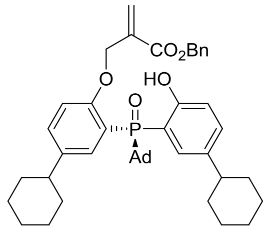

scale (0.1 mmol), product: weight of compound (706.3787), yield (45.2 mg, 0.064 mmol, 64%), colourless oil. **^{1}H NMR** (400 MHz, Chloroform-d) δ 11.88 (s, 1H), 7.94 (dd, *J* = 12.7, 2.3 Hz, 1H), 7.41 (dd, *J* = 12.6, 2.3 Hz, 1H), 7.34 – 7.38 (m, 5H), 7.27 (dd, *J* = 8.2， 2.4 Hz, 1H), 7.17 (d, *J* = 8.7 Hz, 1H), 6.76 – 6.82 (m, 2H), 6.35 (s, 1H), 5.66 (s, 1H), 5.27 (m, 2H), 4.94 – 4.80 (m, 2H), 2.53 – 2.48 (m, 1H), 2.37 – 2.35 (m, 1H), 2.02 (m, 9H), 1.84 – 1.68 (m, 16H), 1.37-1.15 (m, 10H).). **^{13}C NMR** (101 MHz, Chloroform-d) δ 164.94, 162.58(d, *J* = 2.0 Hz), 156.75 (d, *J* = 4.0 Hz), 141.00 (d, *J* = 9.0 Hz), 137.02(d, *J* = 11.0 Hz), 135.48, 134.81, 133.83 (d, *J* = 6.0 Hz), 131.92, 131.89, 131.86, 129.67 (d, *J* = 10.0 Hz), 128.61, 128.40, 128.15, 127.31, 118.40 (d, *J* = 87.0 Hz), 118.09 (d, *J* = 8.0 Hz), 112.48 (d, *J* = 7.0 Hz), 109.75 (d, *J* = 93.0 Hz), 66.77, 66.73, 43.56, 38.94 (d, *J* = 71.7 Hz), 36.52, 35.41, 35.07, 34.50, 34.45, 34.28, 27.79, 27.68, 26.82, 26.78, 26.05, 25.99. **^{31}P NMR** (162 MHz, Chloroform-d) δ 50.38. **HRMS (ESI)** calculated for $[C_{45}H_{55}O_5P+H]^+$: 707.3860, found: 707.3865. **$[\alpha]_D^{20}$**= - 30.4 (c = 0.5, $CHCl_3$). **HPLC separation** (Chiralpak AD, 4.6 x 250mm; *i*-PrOH / hexane = 1 / 4, 1.0 mL/min, 210 nm; tr (minor) = 9.5 min, tr (major) = 7.1 min, 95.5 : 4.5 e.r.).

(R)- Benzyl 2-((2-((R)-((1s,3R,5R,7S)-adamantan-1-yl)(5-(tert-butyl)-2-hydroxyphenyl)phosphoryl)-4-(tert-butyl)phenoxy)methyl)acrylate (3j)

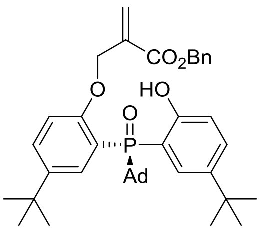

scale (0.1 mmol), product: weight of compound (654.3474), yield (22.2 mg, 0.034 mmol, 34%), colourless oil. **^{1}H NMR** (400 MHz, Chloroform-d) δ 11.77 (s, 1H), 8.07 (dd, *J* = 12.9, 2.6 Hz, 1H), 7.52 (dd, *J* = 12.8, 2.5 Hz, 1H), 7.43 (dd, *J* = 8.7, 2.5 Hz, 1H), 7.37 (m, 6H), 6.82 (dd, *J* = 8.8, 4.6 Hz, 1H), 6.76 (dd, *J* = 8.7, 5.5 Hz, 1H), 6.29 (s, 1H), 5.56 (s, 1H), 5.23 (s, 2H), 4.96 – 4.71 (m, 2H), 2.07– 2.03 (m, 9H), 1.70 – 1.69 (m, 6H), 1.31 (s, 9H), 1.22 (s, 9H)). **^{13}C NMR** (101 MHz, Chloroform-d δ 164.98, 162.20 (d, *J* = 2.0 Hz) , 156.77 (d, *J* = 3.8 Hz), 143.87 (d, *J* = 9.8 Hz), 140.02 (d, *J* = 11.0 Hz), 135.53, 134.57, 132.06 (d, *J* = 6.4 Hz), 130.77, 130.74, 130.70, 128.64 , 128.41 , 128.17 , 127.89 (d, *J* = 10.5 Hz), 127.22 , 118.41 , 117.99 (d, *J* = 85.9 Hz), 117.90 (d, *J* = 8.0 Hz), 112.33 (d, *J* = 7.0 Hz), 109.75 (d, *J* = 92.9 Hz), 66.70, 66.60, 38.88 (d, *J* = 71.5 Hz), 36.58, 35.51, 34.40, 33.98, 31.48, 31.43, 27.82, 27.71. **^{31}P NMR** (162 MHz, Chloroform-d) δ 49.80. **HRMS (ESI)** calculated for $[C_{39}H_{48}O_5P+H]^+$: 655.3547, found: 655.3550. **$[\alpha]_D^{20}$**= - 24.8 (c = 0.5, $CHCl_3$). **HPLC separation** (Chiralpak AD, 4.6 x 250mm; *i*-PrOH / hexane = 1 / 4, 1.0 mL/min, 210 nm; tr (minor) = 5.0 min, tr (major) = 6.9 min, 92.5 : 7.5 e.r.).

(R)- Benzyl 2-((2-((R)-((1s,3R,5R,7S)-adamantan-1-yl)(2-hydroxy-5-methoxyphenyl)phosphoryl)-4-methoxyphenoxy)methyl)acrylate (3k)

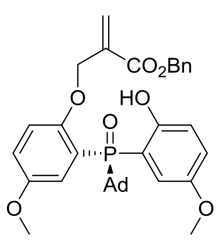

scale (0.1 mmol), product: weight of compound (602.2433), yield (34.9 mg, 0.058 mmol, 58%), colourless oil. **^{1}H NMR** (Chloroform-d δ) 11.47 (s, 1H), 7.68 (dd, *J* = 13.0, 3.2 Hz, 1H), 7.37 (m, 5H), 7.15 (dd, *J* = 13.2, 3.1 Hz, 1H), 6.98 (dd, *J* = 9.0, 3.2 Hz, 1H), 6.92 (dd, *J* = 9.0, 3.0 Hz, 1H), 6.83 (ddd, *J* = 10.8, 9.0, 5.4 Hz, 2H), 6.41 (s, 1H), 5.79 (s, 1H), 5.23 (s, 2H), 4.84 – 4.72 (m, 2H), 3.81 (s, 3H), 3.67 (s, 3H), 2.10 – 2.01 (s, 9H), 1.71 – 1.67 (s, 6H).). **^{13}C NMR** (101 MHz, Chloroform-d δ) 165.04, 158.37 (d, *J* = 2.0 Hz), 153.94 (d, *J* = 12.5 Hz), 152.73 (d, *J* = 3.0 Hz), 151.08 (d, *J* = 14.7 Hz), 135.50, 134.96, 128.64, 128.43, 128.28, 128.22, 119.60 (d, *J* = 84.5 Hz), 119.80 (d, *J* = 3.0 Hz), 119.55 (d, *J* = 6.4 Hz), 119.21 (d, *J* = 2.7 Hz), 118.76 (d, *J* = 9.0 Hz), 117.13 (d, *J* = 12.1 Hz), 114.27 (d, *J* = 8.6 Hz), 110.51 (d, *J* = 91.9 Hz), 67.55, 66.79, 55.90, 39.09 (d, *J* = 71.7 Hz), 36.45, 35.33, 27.74, 27.63. **^{31}P NMR** (162 MHz, Chloroform-d) δ 49.04. **HRMS (ESI)** calculated for $[C_{39}H_{48}O_5P+H]^+$: 603.2506, found: 603.2510. **$[\alpha]_D^{20}$**= - 6.0 (c = 0.5, $CHCl_3$). **HPLC separation** (Chiralpak AD, 4.6 x 250mm; *i*-PrOH / hexane = 1 / 4, 1.0 mL/min, 210 nm; tr (minor) = 13.5 min, tr (major) = 16.8 min, 91.0 : 9.0 e.r.).

(R)-Benzyl2-((2-(((3r)-adamantan-1-yl)(5-fluoro-2-hydroxyphenyl)phosphoryl)-4-fluorophenoxy)methyl)acrylate(3l)

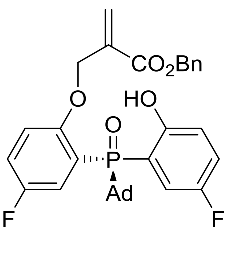

scale (0.1 mmol), product: weight of compound (578.2034), yield (37.6 mg, 0.065 mmol, 65%), colourless oil. **^{1}H NMR** (400 MHz, Chloroform-d) δ 11.72 (s, 1H), 7.92 – 7.82 (m, 1H), 7.36 (d, *J* = 2.8 Hz, 6H), 7.20 – 7.11 (m, 1H), 7.08 – 7.00 (m, 1H), 6.85 (ddd, *J* = m, 2H), 6.50 (s, 1H), 5.84 (s, 1H), 5.24 (s, 2H), 4.94 – 4.68 (m, 2H), 1.98 (m, 9H), 1.69 (t, *J* = 10.7 Hz, 6H). **^{13}C NMR** (101 MHz, Chloroform-d) δ 164.89, 160.84, 157.17 (d, *J* = 243.9 Hz), 154.50 (d, *J* = 238.8 Hz), 135.41, 134.61,

129.69, 128.64, 128.46, 128.22, 122.20 (dd, J = 25.5 Hz), 121.00 (J = 22.9 Hz), 120.54 (d, J = 23.2 Hz), 119.60 (dd, J = 8.4 Hz), 117.43 (dd, J = 24.1 Hz), 113.70 (dd, J = 8.0 Hz), 109.68 (dd, J = 92.7 Hz), 67.67, 66.96, 39.67 (d, J = 71.3 Hz), 36.32, 35.16, 27.63, 27.53. **^{31}P NMR** (162 MHz, Chloroform-d) δ 48.11. **^{19}F NMR** (376 MHz, Chloroform-d) δ -125.99, -129.90. **HRMS (ESI)** calculated for $[C_{33}H_{34}F_2O_5P+H]^+$: 579.2106, found: 579.2106. **$[\alpha]_D^{20}$** = 4.0 (c = 0.5, $CHCl_3$). **HPLC separation** (Chiralpak AD, 4.6 x 250mm; *i*-PrOH / hexane = 1 / 4, 1.0 mL/min, 210 nm; tr (minor) = 10.2 min, tr (major) = 18.4 min, 88.5 : 11.5 e.r.).

(R)-Benzyl2-((2-(((3s,5s,7s)-adamantan-1-yl)(5-chloro-2-hydroxyphenyl)phosphoryl)-4-chlorophenoxy)methyl)acrylate(3m)

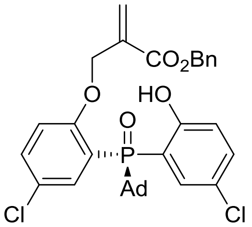

scale (0.1 mmol), product: weight of compound (610.1443), yield (44.5 mg, 0.073 mmol, 73%), colourless oil. **^{1}H NMR** (400 MHz, Chloroform-d) δ 12.00 (s, 1H), 8.10 (dd, J = 12.3, 2.7 Hz, 1H), 7.61 (dd, J = 12.3, 2.6 Hz, 1H), 7.41 (dd, J = 8.8, 2.7 Hz, 1H), 7.36 (m, 5H), 7.26 (dd, J = 8.8, 2.4 Hz, 1H), 6.84 (m, 2H), 6.49 (s, 1H), 5.81 (s, 1H), 5.24 (d, J = 1.8 Hz, 2H), 4.98 – 4.77 (m, 2H), 2.07 – 1.87 (m, 9H), 1.76 – 1.66 (m, 6H). **^{13}C NMR** (101 MHz, Chloroform-*d*) δ 164.84, 163.34 (d, J = 2.4 Hz), 156.90 (d, J = 4.2 Hz), 135.36, 135.25 (d, J = 6.1 Hz), 135.22, 134.23, 133.92 (d, J = 2.3 Hz), 133.71 (d, J = 2.4 Hz), 130.83 (d, J = 11.7 Hz), 129.48, 128.66, 128.49, 128.22, 127.16 (d, J = 2.7 Hz), 122.69 (d, J = 15.8 Hz), 120.31 (d, J = 84.0 Hz), 120.13 (d, J = 8.1 Hz), 113.90 (d, J = 7.4 Hz), 110.86 (d, J = 90.9 Hz), 67.33, 66.99, 39.41 (d, J = 71.7 Hz), 36.32, 35.19, 27.64, 27.53. **^{31}P NMR** (162 MHz, Chloroform-d) δ 48.48. **HRMS (ESI)** calculated for $[C_{33}H_{34}Cl_2O_5P+H]^+$: 611.1515, found: 611.1517. **$[\alpha]_D^{20}$**= - 4.8 (c = 0.5, $CHCl_3$). **HPLC separation** (Chiralpak AD, 4.6 x 250mm; *i*-PrOH / hexane = 1 / 4, 1.0 mL/min, 210 nm; tr (minor) = 9.5 min, tr (major) = 11.7 min, 96 : 4 e.r.).

(R)-Benzyl2-((2-(((3s,5s,7s)-adamantan-1-yl)(5-bromo-2-hydroxyphenyl)phosphoryl)-4-bromophenoxy)methyl)acrylate(3n)

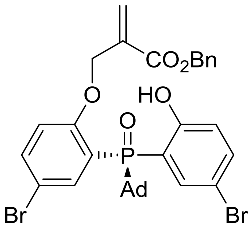

scale (0.1 mmol), product: weight of compound (698.0432), yield (49.6 mg, 0.071 mmol, 71%), colourless oil. **^{1}H NMR** (400 MHz, Chloroform-d) δ 12.03 (s, 1H), 8.23 (d, J = 12.3 Hz, 1H), 7.74 (d, J = 12.0 Hz, 1H), 7.55 (d, J = 8.8 Hz, 1H), 7.46 – 7.30 (m, 5H), 6.78 (ddd, J = 12.7, 8.8, 4.7 Hz, 2H), 6.47 (s, 1H), 5.78 (s, 1H), 5.24 (s, 2H), 5.00 – 4.76 (m, 2H), 1.98 (m, J = 26.8 Hz, 9H), 1.70 (t, J = 10.5 Hz, 6H). **^{13}C NMR** (101 MHz, Chloroform-d) δ 164.82, 163.80 (d, J = 1.1 Hz), 157.37 (d, J = 4.2 Hz), 138.07 (d, J = 5.7 Hz), 138.04, 136.91, 136.53, 135.37, 134.16, 133.72 (d, J = 11.3 Hz), 129.23, 128.67, 128.50, 128.23, 121.76 (d, J = 83.1 Hz) , 120.64 (d, J = 7.6 Hz), 114.37 (d, J = 7.1 Hz), 114.34 (d, J = 13.3 Hz), 110.54 (d, J = 89.6 Hz), 109.77 (d, J = 15.5 Hz), 67.24, 67.00, 39.50 (d, J = 70.7 Hz), 36.32, 35.22, 27.66, 27.55. **^{31}P NMR** (162 MHz, Chloroform-*d*) δ 48.30. **HRMS (ESI)** calculated for $[C_{33}H_{34}Br_2O_5P+H]^+$: 699.0505, found: 699.0503. **$[\alpha]_D^{20}$**= - 20.4 (c = 0.5,

$CHCl_3$). **HPLC separation** (Chiralpak AD, 4.6 x 250mm; *i*-PrOH / hexane = 1 / 4, 1.0 mL/min, 210 nm; tr (minor) = 9.9 min, tr (major) = 11.2 min, 97.5 : 2.5 e.r.).

(R)- Benzyl 2-((2-((R)-((1s,3R,5R,7S)-adamantan-1-yl)(2-hydroxy-5-iodophenyl)phosphoryl)-4-iodophenoxy)methyl)acrylate (3o)

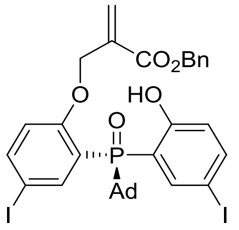

scale (0.1 mmol), product: weight of compound (794.0155), yield (52.4 mg, 0.066 mmol, 66%), colourless oil. **^{1}H NMR** (1H NMR (400 MHz, Chloroform-d) δ 12.08 (s, 1H), 8.37 (dd, *J* = 12.0, 2.3 Hz, 1H), 7.90 (dd, *J* = 11.9, 2.3 Hz, 1H), 7.73 (dd, *J* = 8.7, 2.3 Hz, 1H), 7.55 (dd, *J* = 8.8, 2.1 Hz, 1H), 7.37 (m, 5H), 6.66 (dd, *J* = 8.8, 5.0 Hz, 2H), 6.45 (s, 1H), 5.71 (s, 1H), 5.25 (s, 2H), 5.00 – 4.80 (m, 2H), 2.02 – 1.91 (m, 9H), 1.75 – 1.64 (m, 6H). **^{13}C NMR** (101 MHz, Chloroform-d) δ 164.80, 164.37 (d, *J* = 2.3 Hz), 157.99 (d, J = 4.3 Hz), 143.76 (d, *J* = 5.7 Hz), 142.85 (d, *J* = 2.0 Hz), 142.17(d, *J* = 2.0 Hz), 139.73 (d, *J* = 11.1 Hz), 135.33, 133.97, 128.79, 128.69, 128.53, 128.30, 121.02 (d, *J* = 82.8 Hz), 121.18 (d, *J* = 7.6 Hz), 114.81 (d, *J* = 7.0 Hz), 112.35 (d, *J* = 88.8 Hz), 84.10 (d, *J* = 11.7 Hz), 79.23 (d, *J* = 13.8 Hz), 67.00, 39.44 (d, *J* = 70.8 Hz), 36.31, 35.20, 27.63, 27.52. **^{31}P NMR** (162 MHz, Chloroform-d) δ 47.74. **HRMS (ESI)** calculated for $[C_{33}H_{34}Cl_2O_5P+H]^+$: 795.0228, found: 795.0232. **$[\alpha]_D$**^{20}= - 29.6 (c = 0.5, $CHCl_3$). **HPLC separation** (Chiralpak AD, 4.6 x 250mm; *i*-PrOH / hexane = 1 / 4, 1.0 mL/min, 210 nm; tr (minor) = 10.4 min, tr (major) = 11.1 min, 97 : 3 e.r.).

(R)-Benzyl 2-((2-(tert-butyl(2-hydroxyphenyl)phosphoryl)phenoxy)methyl)acrylate(3P)

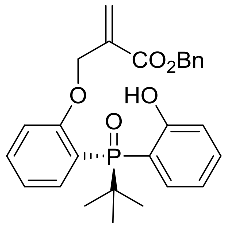

scale (0.1 mmol), product: weight of compound (464.1753), yield (37.6 mg, 0.081 mmol, 81%), colourless oil. **^{1}H NMR** (400 MHz, Chloroform-d) δ 11.89 (s, 1H), 8.08 (ddd, *J* = 12.1, 7.7, 1.7 Hz, 1H), 7.51 – 7.26 (m, 8H), 7.11 (tdd, *J* = 7.5, 2.0, 0.9 Hz, 1H), 6.90 (ddd, *J* = 8.5, 5.1, 0.9 Hz, 1H)， 6.85 (ddd, *J* = 8.4, 4.4, 1.1 Hz, 1H)， 6.72 (dddd, *J* = 8.0, 7.2, 2.8, 1.2 Hz, 1H)， 6.40 (d, *J* = 1.0 Hz, 1H), 5.76 (d, *J* = 1.0 Hz, 1H), 5.22 (s, 2H), 4.83 – 4.70 (m, 2H), 1.31 (d, J = 15.7 Hz, 9H). **^{13}C NMR** (101 MHz, Chloroform-d) δ 165.16， 164.42， 159.25 (d, *J* = 3.3 Hz)， 135.60, 135.02 (d, *J* = 5.7 Hz), 134.83, 134.25, 133.59 (d, *J* = 1.6 Hz), 131.90 (d, *J* = 10.2 Hz), 128.82, 128.74, 128.55, 128.37, 121.27 (d, *J* = 10.1 Hz), 119.12(d, *J* = 88.1 Hz), 118.41 (d, *J* = 7.0 Hz), 117.99 (d, *J* = 12.0 Hz), 112.83 (d, *J* = 6.7 Hz), 111.12 (d, *J* = 94.1 Hz), 66.93, 35.91 (d, *J* = 70.4 Hz), 25.39. **^{31}P NMR** (162 MHz, Chloroform-d) δ 53.82. **HRMS (ESI)** calculated for $[C_{27}H_{30}O_5P+H]^+$: 465.1825, found: 465.1820. **$[\alpha]_D^{20}$** = 4.0 (c = 0.5, $CHCl_3$). **HPLC separation** (Chiralpak AD, 4.6 x 250mm; *i*-PrOH / hexane = 1 / 4, 1.0 mL/min, 210 nm; tr (major) = 9.6, tr (minor) = 10.2 min, 93 : 7 e.r.).

(R)-Benzyl2-((2-((2-hydroxyphenyl)(trityl)phosphoryl)phenoxy)methyl)acrylate(3q)

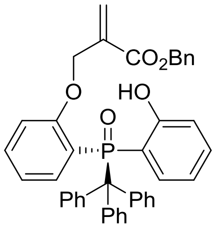

scale (0.1 mmol), product: weight of compound (650.2222), yield (52.7 mg, 0.081 mmol, 81%), white solid. **^{1}H NMR** (400 MHz, Chloroform-*d*) δ 11.55 (s, 1H), 7.54 – 7.04 (m, 23H), 6.84 (t, *J* = 6.9 Hz, 1H), 6.74 (m, 2H), 6.58 – 6.50 (m, 1H), 6.43 (d, *J* = 7.8 Hz, 1H), 6.20 (s, 1H), 5.56 (s, 1H), 5.18 (s, 2H), 4.43 (dd, *J* = 99.9, 14.7 Hz, 2H). **^{13}C NMR** (101 MHz, Chloroform-d) δ 164.99, 164.57 (d, *J* = 1.7 Hz), 160.89 (d, *J* = 1.3 Hz), 140.34 (d, *J* = 2.6 Hz), 135.65 (d, *J* = 7.7 Hz), 135.53, 134.00, 133.99, 133.43 (d, *J* = 2.3 Hz), 132.53 (d, *J* = 9.9 Hz), 131.85, 131.79, 128.57, 128.29, 128.09, 127.82, 127.76, 127.41, 127.40, 122.19 (d, *J* = 90.4 Hz), 120.09 (d, *J* = 11.6 Hz), 118.07 (d, *J* = 8.2 Hz), 117.39 (d, *J* = 12.1 Hz), 113.83 (d, *J* = 95.7 Hz), 113.62 (d, *J* = 6.4 Hz), 67.24 (d, *J* = 63.4 Hz), 66.52, 66.50. **^{31}P NMR** (162 MHz, Chloroform-*d*) δ 47.00. **HRMS (ESI)** calculated for $[C_{27}H_{30}O_5P+H]^+$: 651.2295, found: 651.2298. **$[\alpha]_D^{20}$** = - 46.4 (c = 0.5, $CHCl_3$). **HPLC separation** (Chiralpak AD, 4.6 x 250mm; *i*-PrOH / hexane = 1 / 4, 1.0 mL/min, 210 nm; tr (minor) = 8.6 min, tr (major) = 10.5 min, 88.5 : 11.5 e.r.).

(R)-tert-Butyl2-((2-((R)-((1s,3R,5R,7S)-adamantan-1-yl)(2-hydroxyphenyl)phosphoryl)phenoxy)methyl)acrylate(3r)

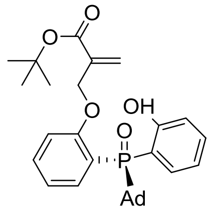

scale (0.1 mmol), product: weight of compound (508.2379), yield (31.0 mg, 0.061 mmol, 61%), semi-solid. **^{1}H NMR** (400 MHz, Chloroform-d) δ 12.07 (s, 1H), 8.21 – 8.07 (m, 1H), 7.66 – 7.56 (m, 1H), 7.47 (t, J = 7.9 Hz, 1H), 7.31 (t, J = 7.9 Hz, 1H), 7.12 (t, J = 7.6 Hz, 1H), 6.88 (ddd, J = 13.1, 7.4, 4.4 Hz, 2H), 6.77 (t, J = 7.8 Hz, 1H), 6.26 (s, 1H), 5.62 (s, 1H), 4.77 (s, 2H), 2.17 – 1.92 (m, 10H), 1.71 (m, 6H), 1.52 (s, 9H). **^{13}C NMR** (101 MHz, Chloroform-d) δ 164.55, 164.44, 158.89(d, *J* = 4.14 Hz), 136.34, 135.49(d, *J* = 5.5 Hz), 133.92, 133.40, 132.11 (d, *J* = 10.2 Hz), 127.09, 121.19 (d, *J* = 10.2 Hz), 118.70 (d, *J* = 89.2 Hz), 118.29 (d, *J* = 7.3 Hz), 117.78 (d, *J* = 12.1 Hz), 112.49 (d, *J* = 7.0 Hz), 110.20 (d, *J* = 93.2 Hz), 81.63, 67.05, 39.00 (d, *J* = 71.3 Hz), 36.47, 35.23, 28.09, 27.74, 27.63. **^{31}P NMR** (162 MHz, Chloroform-d) δ 49.81. **HRMS (ESI)** calculated for $[C_{30}H_{38}O_5P+H]^+$: 509.2451, found: 509.2454 **$[\alpha]_D^{20}$** = 4.8 (c = 0.5, $CHCl_3$). **HPLC separation** (Chiralpak AD, 4.6 x 250mm; *i*-PrOH / hexane = 1 / 4, 1.0 mL/min, 210 nm; tr (minor) = 10.9 min, tr (major) = 15.1 min), 95.5 : 4.5 e.r.).

(R)-Naphthalen-1-ylmethyl2-((2-(((3r)-adamantan-1-yl)(2-hydroxy-5-methylphenyl)phosphoryl)-4-methylphenoxy)methyl)acrylate(3s)

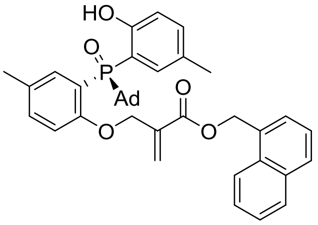

scale (0.1 mmol), product: weight of compound (620.2692), yield (57.7 mg, 0.093 mmol, 93%), colourless oil. **^{1}H NMR** (400 MHz, Chloroform-d) δ 11.82 (s, 1H), 8.06 – 7.80 (m, 4H), 7.58 – 7.32 (m, 5H), 7.18 (d, *J* = 8.4 Hz, 1H), 7.05 (d, *J* = 8.6 Hz, 1H), 6.72 (m, 2H), 6.33 (s, 1H), 5.67 (s, 3H), 4.80 (s, 2H), 2.30 (s, 3H), 2.13 (s, 3H), 2.09 – 1.85 (m, 9H), 1.66 (m, 6H). **^{13}C NMR** (101 MHz, Chloroform-d) δ 165.05, 162.21 (d, *J* = 1.6 Hz), 156.39 (d, *J* = 4.3 Hz), 135.87 (d, *J* = 4.8 Hz), 135.07, 134.36, 134.30, 133.76, 131.63 (d, *J* = 8.3 Hz), 131.49, 130.94, 130.84, 129.58, 128.81, 128.06, 127.72, 126.69, 126.66 (d, *J* = 11.8 Hz), 126.04, 125.25, 123.38, 118.56 (d, *J* = 84.7 Hz), 118.11 (d, *J* = 7.5 Hz),

112.45 (d, *J* = 7.3 Hz), 109.95 (d, *J* = 92.6 Hz), 66.90, 65.26, 38.95(d, *J*=71.8 Hz), 36.48, 35.33, 27.78, 27.6, 20.52. **^{31}P NMR** (162 MHz, Chloroform-d) δ 50.02. **HRMS (ESI)** calculated for $[C_{39}H_{42}O_5P+H]^+$: 621.2764, found: 621.2769. $[\alpha]_D^{20}$ = - 9.6 (c = 0.5, $CHCl_3$). **HPLC separation** (Chiralpak AD, 4.6 x 250mm; *i*-PrOH / hexane = 1 / 4, 1.0 mL/min, 210 nm; tr (minor) = 11.3 min, tr (major) = 15.9 min, 95.5 : 4.5 e.r.).

(R)-2-(trifluoromethyl)benzyl2-((2-(((3s,5s,7s)-adamantan-1-yl)(2-hydroxy-5-methylphenyl)phosphoryl)-4-methylphenoxy)methyl)acrylate(3t)

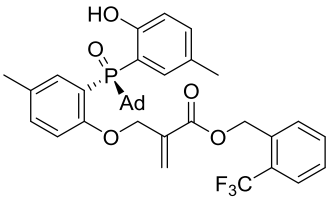

scale (0.1 mmol), product: weight of compound (638.2409), yield (63.2 mg, 0.099 mmol, 99%), colourless oil. **^{1}H NMR** (400 MHz, Chloroform-*d*) δ 11.85 (s, 1H), 7.94 (dd, *J* = 12.5, 2.2 Hz, 1H), 7.71 (d, *J* = 7.7 Hz, 1H), 7.59 – 7.49 (m, 2H), 7.46 (td, *J* = 7.0, 6.2, 2.3 Hz, 1H), 7.39 (dd, *J* = 12.6, 2.1 Hz, 1H), 7.25 (dd, *J* = 8.6, 2.5 Hz, 1H), 7.11 (dd, *J* = 8.4, 2.0 Hz, 1H), 6.77 (dt, *J* = 8.7, 4.6 Hz, 2H), 6.41 (s, 1H), 5.75 (s, 1H), 5.42 (s, 2H), 4.84 (d, *J* = 2.8 Hz, 2H), 2.34 (s, 3H), 2.18 (s, 3H), 2.11 – 1.93 (m, 9H), 1.70 (m, 6H). **^{13}C NMR** (101 MHz, Chloroform-d) δ 164.68, 162.23 (d, *J* = 1.5 Hz), 156.46 (d, *J* = 4.1 Hz), 135.82 (d, *J* = 5.5 Hz), 134.81, 134.34 (d, *J* = 2.6 Hz), 133.59, 132.15, 131.55 (d, *J* = 10.6 Hz), 130.90(d, *J* = 10.2 Hz), 130.31, 128.61, 128.58 (d, *J* = 30.9 Hz), 128.25, 126.67 (d, *J* = 11.9 Hz), 126.33 (d, *J* = 5.6 Hz), 124.12 (d, *J* = 275.1 Hz), 118.10 (d, *J* = 86.5 Hz), 118.14 (d, *J* = 7.5 Hz), 112.46 (d, *J* = 7.3 Hz), 110.97 (d, *J* = 92.5 Hz), 66.80, 63.37 (d, *J* = 2.8 Hz), 63.35, 38.97 (d, *J* = 71.7 Hz), 36.48, 35.33, 27.78, 27.67, 20.60, 20.53. **^{31}P NMR** (162 MHz, Chloroform-d) δ 49.86. **^{19}F NMR** (376 MHz, Chloroform-d) δ -64.61. **HRMS (ESI)** calculated for $[C_{36}H_{39}F_3O_5P+H]^+$: 639.2482, found: 639.2492. **$[\alpha]_D^{20}$** = - 18.4 (c = 0.5, $CHCl_3$). **HPLC separation** (Chiralpak AD, 4.6 x 250mm; *i*-PrOH / hexane = 1 / 4, 1.0 mL/min, 210 nm; tr (minor) = 6.9 min, tr (major) = 9.6 min, 96 : 4 e.r.).

(R)-2-methylbenzyl2-((2-(((3s,5s,7s)-adamantan-1-yl)(2-hydroxy-5-methylphenyl)phosphoryl)-4-methylphenoxy)methyl)acrylate(3u)

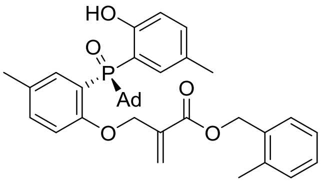

scale (0.1 mmol), product: weight of compound (584.2692), yield (44.9 mg, 0.077 mmol, 77%), colourless oil. **^{1}H NMR** (400 MHz, Chloroform-d) δ 11.85 (s, 1H), 7.94 (dd, *J* = 12.5, 2.3 Hz, 1H), 7.39 (dd, *J* = 12.5, 2.2 Hz, 1H), 7.33 (d, *J* = 7.9 Hz, 1H), 7.30 – 7.17 (m, 4H), 7.10 (s, 1H), 6.77 (m, 2H), 6.36 (d, *J* = 1.1 Hz, 1H), 5.71 (s, 1H), 5.25 (s, 2H), 4.83 (s, 1H), 2.36 (s, 3H), 2.33 (s, 3H), 2.18 (s, 3H), 2.11 – 1.92 (m, 9H), 1.71 (m, 6H). **^{13}C NMR** (101 MHz, Chloroform-d) δ 165.01, 162.23 (d, *J* = 2.2 Hz), 156.40 (d, *J* = 4.5 Hz), 137.02, 135.86 (d, *J* = 5.4 Hz), 135.04, 133.34 (d, *J* = 4.0 Hz), 134.33, 133.39, 131.56 (d, *J* = 10.8 Hz), 130.91 (d, *J* = 10.4 Hz), 130.48, 129.31, 128.77, 127.66, 126.64 (d, *J* = 11.9 Hz), 126.09, 118.57 (d, *J* = 84.8 Hz), 118.11 (d, *J* = 8.0 Hz), 112.48 (d, *J* = 6.8 Hz), 109.93 (d, *J* = 92.9 Hz), 66.88, 65.32, 38.96 (d, *J* = 71.7 Hz), 36.49, 35.33, 27.78, 27.67, 20.66, 20.54, 18.96. **^{31}P NMR** (162 MHz, Chloroform-d) δ 49.93. **HRMS**

(ESI) calculated for $[C_{36}H_{42}O_5P+H]^+$: 585.2764, found: 585.2769. **$[\alpha]_D^{20}$** = - 12.8 (c = 0.5, $CHCl_3$). **HPLC separation** (Chiralpak AD, 4.6 x 250mm; 25% *i*-PrOH / hexane = 1 / 4, 1.0 mL/min, 210 nm; tr (minor) = 7.9 min, tr (major) = 12.1 min, 95.5 : 4.5 e.r.).

(R)-2-methoxybenzyl2-((2-(((3s,5s,7s)-adamantan-1-yl)(2-hydroxy-5-methylphenyl)phosphoryl)-4-methylphenoxy)methyl)acrylate(3v)

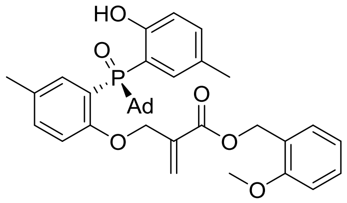

scale (0.1 mmol), product: weight of compound (600.2641), yield (17.6 mg, 0.046 mmol, 46%), colourless oil. **^{1}H NMR** (400 MHz, Chloroform-d) δ 11.88 (s, 1H), 7.95 (dd, *J* = 12.6, 2.2 Hz, 1H), 7.42 (dd, *J* = 12.7, 2.1 Hz, 1H), 7.32 (d, *J* = 7.8 Hz, 2H), 7.24 (dd, *J* = 8.6, 2.2 Hz, 1H), 7.14 – 7.06 (m, 1H), 6.95 (t, *J* = 7.5 Hz, 1H), 6.90 (d, *J* = 8.2 Hz, 1H), 6.77 (dt, *J* = 8.7, 4.6 Hz, 2H), 6.38 (s, 1H), 5.70 (s, 1H), 5.29 (s, 2H), 4.85 (s, 2H), 3.83 (s, 3H), 2.33 (s, 3H), 2.19 (s, 3H), 2.11 – 1.93 (m, 9H), 1.70 (m, 6H). **^{13}C NMR** (101 MHz, Chloroform-d) δ 165.17, 162.33 (d, *J* = 1.81 Hz), 157.40 (d, *J* = 3.9 Hz), 135.93 (d, *J* = 7.2 Hz), 135.26, 134.34 (d, *J* = 2.5 Hz), 134.30 (d, *J* = 2.1 Hz), 131.62 (d, *J* = 11.0 Hz), 130.86 (d, *J* = 10.5 Hz), 129.80, 129.64, 127.55, 126.67 (d, *J* = 11.8 Hz), 123.77, 120.43, 118.56 (d, *J* = 86.2 Hz), 118.10 (d, *J* = 7.5 Hz), 112.40 (d, *J* = 7.2 Hz), 110.51, 109.93 (d, *J* = 92.1 Hz), 66.96, 62.43, 55.42, 38.97 (d, *J* = 70.7 Hz), 36.51, 35.34, 27.80, 27.69, 20.64, 20.53. **^{31}P NMR** (162 MHz, Chloroform-d) δ 50.15. **HRMS (ESI)** calculated for $[C_{36}H_{42}O_6P+H]^+$: 601.2714, found: 601.2721. **$[\alpha]_D^{20}$** = - 12.0 (c = 0.5, $CHCl_3$). **HPLC separation** (Chiralpak AD, 4.6 x 250mm; *i*-PrOH / hexane = 1 / 4, 1.0 mL/min, 210 nm; tr (minor) = 9.6 min, tr (major) = 13.8 min, 96.5 : 3.5 e.r.).

(R)-2-chlorobenzyl2-((2-(((3s,5s,7s)-adamantan-1-yl)(2-hydroxy-5-methylphenyl)phosphoryl)-4-methylphenoxy)methyl)acrylate(3w)

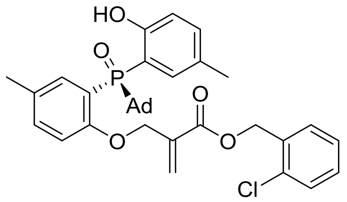

scale (0.1 mmol), product: weight of compound (604.2145), yield (59.8 mg, 0.099 mmol, 99%), colourless oil. **^{1}H NMR** (400 MHz, Chloroform-d) δ 11.84 (s, 1H), 7.94 (dd, *J* = 12.6, 2.3 Hz, 1H), 7.40 (qd, *J* = 7.6, 1.9 Hz, 3H), 7.35 – 7.20 (m, 3H), 7.11 (d, *J* = 8.3 Hz, 1H), 6.78 (dt, *J* = 8.5, 5.1 Hz, 2H), 6.41 (s, 1H), 5.74 (s, 1H), 5.33 (s, 2H), 4.84 (s, 2H), 2.34 (s, 3H), 2.19 (s, 3H), 2.13 – 1.91 (m, 9H), 1.70 (M, 6H). **^{13}C NMR** (101 MHz, Chloroform-d) δ 164.84, 162.19 (d, *J* = 2.3 Hz), 156.41 (d, *J* = 4.24 Hz), 135.84 (d, *J* = 5.4 Hz), 134.88, 134.37, 134.25, 133.91, 133.11, 131.56 (d, *J* = 10.4 Hz), 130.90 (d, *J* = 10.2 Hz), 130.07, 129.84, 129.72, 128.12, 126.96, 126.68 (d, *J* = 12.1 Hz), 118.51 (d, *J* = 86.7 Hz), 118.11 (d, *J* = 7.9 Hz), 112.46 (d, *J* = 7.3 Hz), 110.82 (d, *J* = 92.5 Hz), 66.83, 64.29, 38.95 (d, *J* = 71.7 Hz), 36.49, 36.47, 35.32, 35.30, 27.76, 27.66, 20.67, 20.55. **^{31}P NMR** (162 MHz, Chloroform-d) δ 49.91. **HRMS (ESI)** calculated for $[C_{35}H_{39}ClO_5P+H]^+$: 605.2218, found: 605.2220. **$[\alpha]_D^{20}$** = - 8.4 (c = 0.5, $CHCl_3$). **HPLC separation** (Chiralpak AD, 4.6 x 250mm; *i*-PrOH / hexane = 1 / 4, 1.0 mL/min, 210 nm; tr (minor) = 8.5 min, tr (major) = 12.4 min, 95.5 : 4.5 e.r.).

(R)-3-methylbenzyl2-((2-(((3s,5s,7s)-adamantan-1-yl)(2-hydroxy-5-methylphenyl)phosphoryl)-4-methylphenoxy)methyl)acrylate(3x)

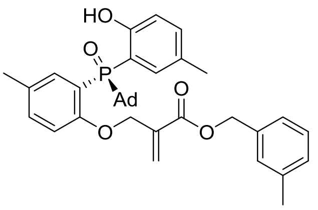

scale (0.1 mmol), product: weight of compound (584.2692), yield (43.8 mg, 0.075 mmol, 75%), colourless oil. **^{1}H NMR** (400 MHz, Chloroform-d) δ 11.85 (d, *J* = 1.9 Hz, 1H), 7.95 (d, *J* = 12.5 Hz, 1H), 7.40 (d, *J* = 12.6 Hz, 1H), 7.29 – 7.05 (m, 6H), 6.83 – 6.70 (m, 2H), 6.39 (s, 1H), 5.72 (s, 1H), 5.20 (s, 2H), 4.84 (s, 2H), 2.35 (s, 3H), 2.34 (s, 3H), 2.19 (d, *J* = 1.9 Hz, 3H), 2.13 – 1.94 (m, 9H), 1.70 (s, 6H). **^{13}C NMR** (101 MHz, Chloroform-d) δ 165.03, 162.19 (d, *J* = 1.7 Hz), 156.38 (d, *J* = 4.5 Hz), 138.37, 135.88 (d, *J* = 5.4 Hz), 135.32, 135.05, 134.32 (d, *J* = 6.5 Hz), 134.32, 131.56 (d, *J* = 10.6 Hz), 130.88 (d, *J* = 10.1 Hz), 129.22, 128.99, 128.54, 127.84, 126.66 (d, *J* = 11.8 Hz), 125.32, 118.53 (d, *J* = 86.3 Hz), 118.10 (d, *J* = 7.9 Hz), 112.41 (d, *J* = 7.3 Hz), 109.93 (d, *J* = 92.6 Hz), 66.90, 38.94 (d, *J* = 71.2 Hz), 36.48, 35.31, 27.77, 27.66, 21.36, 20.65, 20.52. **^{31}P** NMR (162 MHz, Chloroform-d) δ 50.05. **HRMS (ESI)** calculated for $[C_{36}H_{42}O_5P+H]^+$: 585.2764, found: 585.2772. **$[\alpha]_D^{20}$** = -8.4 (c = 0.5, $CHCl_3$). **HPLC separation** (Chiralpak AD, 4.6 x 250mm; *i*-PrOH / hexane = 1 / 4, 1.0 mL/min, 210 nm; tr (minor) = 8.0 min, tr (major) = 10.2 min, 95 : 5 e.r.).

(R)-3-methoxybenzyl2-((2-(((3s,5s,7s)-adamantan-1-yl)(2-hydroxy-5-methylphenyl)phosphoryl)-4-methylphenoxy)methyl)acrylate(3y)

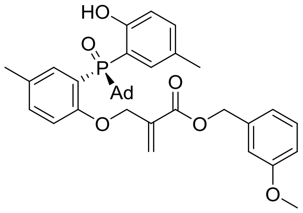

scale (0.1 mmol), product: weight of compound (600.2641), yield (46.8 mg, 0.078 mmol, 78%), colourless oil. **^{1}H NMR** (400 MHz, Chloroform-d) δ 11.83 (s, 1H), 7.94 (d, *J* = 12.5 Hz, 1H), 7.39 (d, *J* = 12.5 Hz, 1H), 7.30 (d, *J* = 7.6 Hz, 1H), 7.24 (d, *J* = 7.6 Hz, 1H), 7.11 (d, *J* = 8.6 Hz, 1H), 7.00 – 6.69 (m, 5H), 6.39 (s, 1H), 5.73 (s, 1H), 5.21 (d, J = 2.4 Hz, 2H), 4.84 (s, 2H), 3.80 (s, 3H), 2.33 (s, 3H), 2.19 (s, 3H), 2.12 – 1.93 (m, 9H), 1.71 (m, 6H). **^{13}C NMR** (101 MHz, Chloroform-d) δ 164.98, 162.18 (d, *J* = 2.2 Hz), 159.75, 156.38 (d, *J* = 4.1 Hz), 136.93, 135.86 (d, *J* = 5.6 Hz), 134.37 (d, *J* = 5.2 Hz), 134.34, 131.56 (d, *J* = 10.5 Hz), 131.90 (d, *J* = 10.2 Hz), 129.74, 127.93, 126.68 (d, *J* = 12.0 Hz), 120.37, 118.51 (d, *J* = 86.6 Hz), 118.11 (d, *J* = 7.2 Hz), 113.82, 113.73, 112.42 (d, *J* = 7.4 Hz), 109.94 (d, *J* = 92.3 Hz), 66.85, 66.70, 55.25, 38.94 (d, *J* = 71.5 Hz), 36.49, 36.47, 35.32, 35.30, 27.77, 27.66, 20.67, 20.54. **^{31}P NMR** (162 MHz, Chloroform-d) δ 49.98. **HRMS (ESI)** calculated for $[C_{36}H_{42}O_6P+H]^+$: 601.2714, found: 601.2720. $[\alpha]_D^{20}$ = - 7.2 (c = 0.5, $CHCl_3$). **HPLC separation** (Chiralpak AD, 4.6 x 250mm; *i*-PrOH / hexane = 1 / 4, 1.0 mL/min, 210 nm; tr (minor) = 11.7 min, tr (major) = 15.4 min, 95 : 5 e.r.).

(R)-3-chlorobenzyl2-((2-(((3s,5s,7s)-adamantan-1-yl)(2-hydroxy-5-methylphenyl)phosphoryl)-4-methylphenoxy)methyl)acrylate(3z)

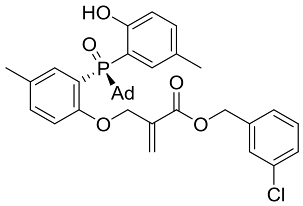

scale (0.1 mmol), product: weight of compound (604.2145), yield (53.2 mg, 0.088 mmol, 88%), colourless oil. **^{1}H NMR** (400 MHz, Chloroform-d) δ 11.83 (s, 1H), 7.94 (dd, *J* = 12.5, 2.3 Hz, 1H), 7.43 – 7.17 (m, 6H), 7.11 (d, *J* = 8.5 Hz, 1H), 6.78 (ddd, *J* = 8.3, 5.0, 3.1 Hz, 2H), 6.40 (s, 1H), 5.76 (s, 1H), 5.20 (s, 2H), 4.83 (d, *J* = 1.7 Hz, 2H), 2.34 (s, 3H), 2.19 (s, 3H), 2.12 – 1.92 (m, 9H), 1.78 – 1.65 (m, 6H). **^{13}C NMR** (101 MHz, Chloroform-d) δ 164.83, 162.24 (d, *J* = 2.5 Hz), 156.46 (d, *J* = 3.8 Hz), 137.43, 135.84 (d, *J* = 5.5 Hz), 134.87, 134.56, 134.34, 131.53 (d, *J* = 10.5 Hz), 130.93 (d, *J* = 10.1 Hz), 129.96, 128.63, 128.61, 128.21, 126.65 (d, *J* = 11.9Hz), 126.23, 118.61 (d, *J* = 88.3 Hz), 118.12 (d, *J* = 8.1 Hz), 112.48 (d, *J* = 7.4 Hz), 109.98 (d, *J* = 92.8 Hz), 66.85, 65.89, 38.96 (d, *J* = 71.8 Hz), 36.50, 35.34, 27.79, 27.68, 20.67, 20.55. **^{31}P NMR** (162 MHz, Chloroform-d) δ 49.85. **HRMS (ESI)** calculated for $[C_{35}H_{39}ClO_5P+H]^+$: 605.2218, found: 605.2222. $[\alpha]_D^{20}$ = - 10.4 (c = 0.5, $CHCl_3$). **HPLC separation** (Chiralpak AD, 4.6 x 250mm; *i*-PrOH / hexane = 1 / 4, 1.0 mL/min, 210 nm; tr (minor) = 9.8 min, tr (major) = 14.1 min, 96 : 4 e.r.).

(R)-4-fluorobenzyl2-((2-(((3s,5s,7s)-adamantan-1-yl)(2-hydroxy-5-methylphenyl)phosphoryl)-4-methylphenoxy)methyl)acrylate(3a')

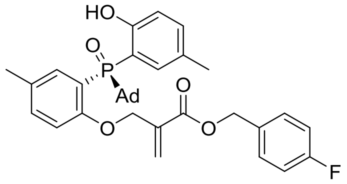

scale (0.1 mmol), product: weight of compound (588.2441), yield (50.0 mg, 0.085 mmol, 85%), colourless oil. **^{1}H NMR** (400 MHz, Chloroform-d) δ 11.83 (s, 1H), 7.94 (dd, *J* = 12.5, 2.3 Hz, 1H), 7.52 – 7.01 (m, 7H), 6.77 (ddd, *J* = 8.1, 5.0, 2.5 Hz, 2H), 6.38 (d, *J* = 1.1 Hz, 1H), 5.72 (s, 1H), 5.30 (d, *J* = 1.0 Hz, 2H), 4.83 (s, 2H), 2.33 (s, 3H), 2.18 (s, 3H), 2.12 – 1.90 (m, 9H), 1.78 – 1.65 (m, 6H). **^{13}C NMR** (101 MHz, Chloroform-d) δ 164.91, 162.19 (d, *J* = 2.4 Hz), 161.09 (d, *J* = 249.8 Hz), 156.41 (d, *J* = 4.1 Hz), 135.85 (d, *J* = 5.3 Hz), 134.89, 134.36, 134.33, 131.56 (d, *J* = 10.6 Hz), 130.89 (d, *J* = 10.2 Hz), 130.60 (d, *J* = 3.6 Hz), 130.54 (d, *J* = 8.2 Hz), 128.02, 126.67 (d, *J* = 11.9 Hz), 124.23 (d, *J* = 3.7 Hz), 122.61 (d, *J* = 14.4 Hz), 118.53 (d, *J* = 86.8 Hz), 118.10 (d, *J* = 7.9 Hz), 115.61 (d, *J* = 20.8 Hz), 112.45 (d, *J* = 7.4 Hz), 109.95 (d, *J* = 92.7 Hz), 66.83, 60.91 (d, *J* = 4.2 Hz), 38.9 (d, *J* = 71.2 Hz), 36.48, 35.32, 27.77, 27.67, 20.63, 20.53. **^{31}P NMR** (162 MHz, Chloroform-d) δ 49.95. **^{19}F NMR** (376 MHz, Chloroform-d) δ -122.45. **HRMS (ESI)** calculated for $[C_{35}H_{39}FO_5P+H]^+$: 589.2514, found: 589.2509. $[\alpha]_D^{20}$ = - 7.2 (c = 0.5, $CHCl_3$). **HPLC separation** (Chiralpak AD, 4.6 x 250mm; *i*-PrOH / hexane = 1 / 4, 1.0 mL/min, 210 nm; tr (minor) = 8.4 min, tr (major) = 11.2 min, 96 : 4 e.r.).

(R)-4-chlorobenzyl2-((2-(((3s,5s,7s)-adamantan-1-yl)(2-hydroxy-5-methylphenyl)phosphoryl)-4-methylphenoxy)methyl)acrylate(3b')

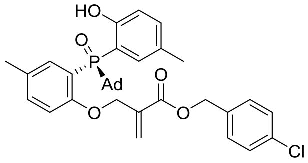

scale (0.1 mmol), product: weight of compound (604.2145), yield (59.8 mg, 0.075 mmol, 99%), colourless oil. **^{1}H NMR** (400 MHz, Chloroform-d) δ 11.81 (s, 1H), 7.94

(d, J = 12.5 Hz, 1H), 7.48 – 7.18 (m, 6H), 7.11 (d, J = 8.6 Hz, 1H), 6.77 (dt, J = 8.0, 3.5 Hz, 2H), 6.37 (s, 1H), 5.74 (s, 1H), 5.19 (s, 2H), 4.82 (s, 2H), 2.34 (s, 3H), 2.18 (s, 3H), 2.02 (d, J = 18.7 Hz, 9H), 1.70 (s, 6H). **^{13}C NMR** (101 MHz, Chloroform-d) δ 165.03, 162.33 (d, J = 1.7 Hz), 156.57 (d, J = 4.1 Hz), 135.94 (d, J = 5.6 Hz), 135.01, 134.55, 134.47 (d, J = 4.1 Hz), 134.47, 134.04, 131.65 (d, J = 10.5 Hz), 131.10 (d, J = 10.1 Hz), 129.82, 128.99, 128.23, 126.77 (d, J = 12.1 Hz), 118.67 (d, J = 86.8 Hz), 118.25 (d, J = 8.0 Hz), 112.57 (d, J = 7.3 Hz), 110.11 (d, J = 92.5 Hz), 66.95, 66.16, 39.11 (d, J = 71.4 Hz), 36.61, 35.45, 27.89, 27.79, 20.80, 20.68. **^{31}P NMR** (162 MHz, Chloroform-d) δ 49.82. **HRMS (ESI)** calculated for $[C_{35}H_{39}ClO_5P+H]^+$:605.2218, found: 605.2212. $[\alpha]_D^{20}$ = - 10.0 (c = 0.5, $CHCl_3$). **HPLC separation** (Chiralpak AD, 4.6 x 250mm; *i*-PrOH / hexane = 1 / 4, 1.0 mL/min, 210 nm; tr (minor) = 10.1 min, tr (major) = 12.8 min, 96 : 4 e.r.).

(R)-2-methylbenzyl2-((2-(((3r)-adamantan-1-yl)(5-bromo-2-hydroxyphenyl)phosphoryl)-bromophenoxy)methyl)acrylate(3c')

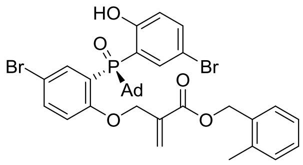

scale (0.1 mmol), product: weight of compound (712.0589), yield (37.0 mg, 0.052mmol, 52%), colourless oil. **^{1}H NMR** (400 MHz, Chloroform-d) δ 12.03 (s, 1H), 8.23 (dd, J = 12.3, 2.5 Hz, 1H), 7.74 (dd, J = 12.2, 2.4 Hz, 1H), 7.55 (dd, J = 8.8, 2.5 Hz, 1H), 7.39 (dd, J = 9.0, 2.3 Hz, 1H), 7.33 (d, J = 7.4 Hz, 1H), 7.30 – 7.15 (m, 3H), 6.78 (td, J = 8.8, 5.1 Hz, 2H), 6.45 (s, 1H), 5.76 (s, 1H), 5.26 (s, 2H), 5.07 – 4.73 (m, 2H), 2.36 (s, 3H), 2.11 – 1.85 (m, 9H), 1.81 – 1.60 (m, 6H). **^{13}C NMR** (101 MHz, Chloroform-d) δ 164.83, 163.77, 157.33 (d, J = 3.5 Hz), 138.05 (d, J = 5.6 Hz), 137.02, 136.92, 136.53, 134.09, 133.72(d, J = 11.0 Hz), 133.31, 130.49, 129.32, 129.10, 128.81, 126.11, 120.69 (d, J = 83.4 Hz), 120.64 (d, J = 8.4 Hz), 114.41, 114.31 (d, J = 5.5 Hz), 111.48 (d, J = 89.8 Hz), 109.77 (d, J = 14.9 Hz), 67.21, 65.47, 39.45 (d, J = 71.1 Hz), 36.30, 35.19, 27.63, 27.53, 18.98. **^{31}P NMR** (162 MHz, Chloroform-d) δ 48.30. **HRMS (ESI)** calculated for $[C_{34}H_{35}Br_2O_5P+H]^+$: 715.0641, found: 715.0638. $[\alpha]_D^{20}$ = - 18.1 (c = 0.5, $CHCl_3$). **HPLC separation** (Chiralpak AD, 4.6 x 250mm; *i*-PrOH / hexane = 1 / 4, 1.0 mL/min, 210 nm; tr (minor) = 8.9 min, tr (major) = 11.3 min, 96.5 : 3.5 e.r.).

(R)-3-methoxybenzyl2-((2-(((3r)-adamantan-1-yl)(2-hydroxy-5-methylphenyl)phosphoryl)-4-methylphenoxy)methyl)acrylate(3d')

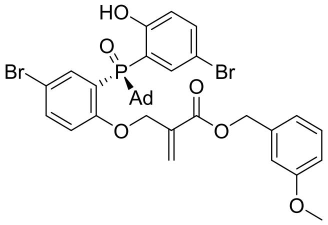

scale (0.1 mmol), product: weight of compound (728.0538), yield (29.8 mg, 0.041 mmol, 41%), colourless oil. **^{1}H NMR** (400 MHz, Chloroform-d) δ 12.04 (s, 1H), 8.23 (dd, J = 12.2, 2.6 Hz, 1H), 7.74 (dd, J = 12.2, 2.5 Hz, 1H), 7.56 (dd, J = 8.9, 2.6 Hz, 1H), 7.40 (ddd, J = 8.9, 2.5, 0.8 Hz, 1H), 7.29 (td, J = 7.7, 0.9 Hz, 1H), 6.95 (d, J = 7.6 Hz, 1H), 6.93 – 6.85 (m, 2H), 6.79 (ddd, J = 10.5, 8.8, 5.1 Hz, 2H), 6.48 (s, 1H), 5.78 (s, 1H), 5.21 (d, J = 2.2 Hz, 2H), 5.02 – 4.75 (m, 2H), 3.81 (s, 3H), 2.11 – 1.86 (m, 9H), 1.75-1.67 (m, 6H). ^{13}C NMR (101 MHz, Chloroform-d) δ 164.92, 163.84 (d, J = 2.2 Hz), 159.87, 157.41 (d, J = 4.1 Hz), 138.15 (d, J = 5.9 Hz), 137.05 (d,

J = 2.1 Hz), 136.93, 136.66 (d, *J* = 2.2 Hz), 134.12, 133.83(d, *J*=11.5 Hz), 129.87, 129.47, 120.75 (d, *J* = 8.0 Hz), 120.74 (d, *J* = 83.6 Hz), 120.49, 114.49 (d, *J* = 1.9 Hz), 114.39 (d, *J* = 3.4 Hz), 114.37 (d, *J* = 83.4 Hz), 114.01, 113.82, 111.58 (d, *J* = 90.0 Hz), 109.92 (d, *J* = 15.2 Hz), 67.28, 66.96, 55.39, 39.53 (d, *J* = 71.1 Hz), 36.41, 36.39, 35.29, 35.27, 27.72, 27.61. ^{31}P NMR (162 MHz, Chloroform-d) δ 48.34. **HRMS (ESI)** calculated for $[C_{34}H_{35}Br_2O_6P+H]^+$:731.0591, found: 731.0589. $[\alpha]_D^{20}$ = - 27.6 (c = 0.5, $CHCl_3$). **HPLC separation** (Chiralpak AD, 4.6 x 250mm; *i*-PrOH / hexane = 1 / 4, 1.0 mL/min, 210 nm; tr (minor) = 12.7 min, tr (major) = 13.6 min, 97.5 : 2.5 e.r.).

(R)-4-fluorobenzyl2-((4-bromo-2-((5-bromo-2-hydroxyphenyl)(methyl)phosphoryl)phenoxy)methyl)acrylate(3e')

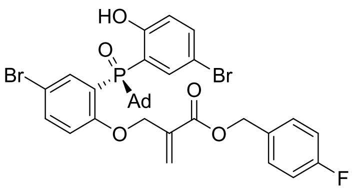

scale (0.1 mmol), product: weight of compound (716.0338), yield (50.1 mg, 0.070 mmol, 70%), colourless oil. **^{1}H NMR** (400 MHz, Chloroform-d) δ 12.03 (s, 1H), 8.23 (dd, *J* = 12.2, 2.6 Hz, 1H), 7.73 (dd, *J* = 12.2, 2.5 Hz, 1H), 7.55 (dd, *J* = 8.9, 2.5 Hz, 1H), 7.42 – 7.29 (m, 3H), 7.19 – 7.03 (m, 1H), 6.78 (ddd, *J* = 11.5, 8.8, 5.1 Hz, 2H), 6.47 (s, 1H), 5.78 (s, 1H), 5.30 (s, 2H), 5.04 – 4.75 (m, 2H), 1.98 (dq, *J* = 34.5, 7.3, 5.4 Hz, 10H), 1.79 – 1.55 (m, 8H). **^{13}C NMR** (101 MHz, Chloroform-d) δ 164.85, 163.89 (d, *J* = 1.4 Hz), 161.23 (d, *J* = 249.6 Hz), 157.46 (d, *J* = 4.24 Hz), 138.17 (d, *J* = 5.7 Hz), 137.04 (d, *J* = 0.8 Hz), 136.66 (d, *J* = 0.9 Hz), 134.10, 133.85 (d, *J* = 12.8 Hz), 130.84 (d, *J* = 4.34 Hz), 130.72 (d, *J* = 8.7 Hz), 129.61, 124.38 (d, *J* = 4.0 Hz), 122.65 (d, *J* = 14.2Hz), 120.81 (d, *J* = 83.1 Hz), 120.76 (d, *J* = 8.5 Hz), 115.77 (d, *J* = 21.4Hz), 114.47 (d, *J* = 7.6 Hz), 114.45 (d, *J* = 12.0 Hz), 111.64 (d, *J* = 89.8 Hz), 109.91 (d, *J* = 15.0 Hz), 67.29, 61.21 (d, *J* = 4.24 Hz), 39.57 (d, *J* = 71.1 Hz), 36.43, 35.32, 27.76, 27.65. **HRMS (ESI)** calculated for $[C_{34}H_{35}Br_2O_6P+H]^+$:717.0411, found: 717.0410. **$[\alpha]_D^{20}$** = - 28.2 (c = 0.5, $CHCl_3$). **HPLC separation** (Chiralpak AD, 4.6 x 250mm; *i*-PrOH / hexane = 1 / 4, 1.0 mL/min, 210 nm; tr (minor) = 12.7 min, tr (major) = 13.6 min, 97 : 3 e.r.).

(R)-Benzyl2-((2-(((3s,5s,7s)-adamantan-1-yl)(5-bromo-2-hydroxyphenyl)phosphoryl)phenoxy)methyl)acrylate

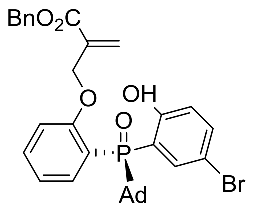

scale (0.1 mmol), product: weight of compound (620.1327), yield (36.0 mg, 0.058 mmol, 58%), colourless oil. **^{1}H NMR** (400 MHz, Chloroform-d) δ 12.17 (s, 1H), 8.13 (dd, J = 12.4, 7.7 Hz, 1H), 7.72 (d, J = 12.2 Hz, 1H), 7.49 (t, J = 7.9 Hz, 1H), 7.37 (s, 6H), 7.14 (t, J = 7.5 Hz, 1H), 6.91 (dd, J = 8.4, 5.5 Hz, 1H), 6.76 (dd, J = 8.9, 4.6 Hz, 1H), 6.46 (s, 1H), 5.81 (s, 1H), 5.25 (s, 2H), 4.99 – 4.77 (m, 2H), 2.00 (m, 9H), 1.71 (m, 6H). **^{13}C NMR** (101 MHz, Chloroform-*d*) δ 165.10, 163.84, 158.69(d, *J* = 3.7 Hz), 136.34, 135.72 (d, *J* = 5.8 Hz), 135.60, 134.48 (d, *J* = 7.6 Hz), 133.92 (d, *J* = 11.2 Hz), 128.94, 128.79, 128.58, 128.34, 121.66 (d, *J* = 10.8 Hz), 120.56 (d, *J* = 7.7 Hz), 118.17 (d, *J* = 87.7 Hz), 112.76 (d, *J* = 6.7 Hz), 112.46 (d, *J* = 89.5 Hz), 109.84 (d, *J* = 15.3 Hz), 67.01, 66.97, 39.39 (d, *J* = 71.2 Hz), 36.51, 35.34, 27.81, 27.70. **^{31}P NMR** (162 MHz, Chloroform-*d*) δ 49.46. **HRMS (ESI)** calculated for $[C_{33}H_{35}BrO_5P+H]^+$: 623.1380, found: 623.1374. **HPLC separation** (Chiralpak AD, 4.6 x 250mm; *i*-PrOH / hexane = 1 / 4, 1.0 mL/min, 210 nm; tr (major) = 14.8 min, tr (minor) = 16.8 min,

94 : 6 e.r.).

(R)-Benzyl2-((2-(((3s,5s,7s)-adamantan-1-yl)(3,5-dibromo-2-hydroxyphenyl)phosphoryl)phenoxy)methyl)acrylate

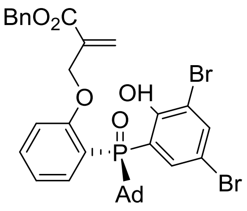

scale (0.1 mmol), product: weight of compound (698.0432), yield (20.2 mg, 0.075 mmol, 29%), colourless oil. **^{1}H NMR** (400 MHz, Chloroform-*d*) δ 13.08 (s, 1H), 8.12 (ddd, *J* = 12.4, 7.8, 1.7 Hz, 1H), 7.74 – 7.63 (m, 2H), 7.51 (t, *J* = 7.9 Hz, 1H), 7.42 – 7.31 (m, 5H), 7.19 – 7.10 (m, 1H), 6.93 (dd, *J* = 8.3, 5.4 Hz, 1H), 6.49 (s, 1H), 5.82 (s, 1H), 5.25 (d, *J* = 0.8 Hz, 2H), 4.91 (q, *J* = 14.2 Hz, 2H), 2.14 – 1.90 (m, 9H), 1.83 – 1.50 (m, 6H). **^{13}C NMR** (101 MHz, Chloroform-*d*) δ 164.90, 160.10 (d, *J* = 2.8 Hz), 158.55 (d, *J* = 4.2 Hz), 138.81, 135.59 (d, *J* = 5.7 Hz) , 135.41, 134.65, 134.32, 132.92 (d, *J* = 11.5 Hz), 128.99, 128.67, 128.48, 128.24, 121.65 (d, *J* = 10.6 Hz), 117.38 (d, *J* = 88.4 Hz), 113.36 (d, *J* = 86.1Hz), 112.32 (d, *J* = 10.5 Hz), 112.70 (d, *J* = 7.0 Hz), 109.42 (d, *J* = 16.2 Hz), 66.96, 66.93, 39.43 (d, *J* = 71.1 Hz), 36.30, 35.17, 27.63 , 27.52. **^{31}P NMR** (162 MHz, Chloroform-*d*) δ 50.23. **HRMS (ESI)** calculated for $[C_{33}H_{34}Br_2O_5P+H]^+$: 701.0485, found:701.0482. **HPLC separation** (Chiralpak IA, 4.6 x 250mm; *i*-PrOH / hexane = 2 / 3, 1.0 mL/min, 210 nm; tr (major) = 8.6 min, tr (minor) = 18.3 min, 94 : 6 e.r.).

(R)-((3s,5s,7s)-adamantan-1-yl)(5-bromo-2-hydroxyphenyl)(2-hydroxyphenyl)phosphine oxide

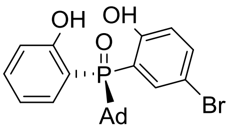

scale (0.058 mmol), product: weight of compound (446.0646), yield (24.8 mg, 0.056 mmol, 96%), white solid. **^{1}H NMR** (400 MHz, Chloroform-*d*) δ 11.24 (s, 1H), 11.07 (s, 1H), 7.67 – 7.36 (m, 4H), 6.97 (td, *J* = 7.7, 3.6 Hz, 2H), 6.84 (dd, *J* = 9.1, 4.7 Hz, 1H), 2.08-1.97 (m, 12H), 1.79 – 1.66 (m, 6H). **^{13}C NMR** (101 MHz, Chloroform-*d*) δ 164.38, 163.36, 137.11 (d, *J* = 1.7 Hz), 134.70 (d, *J* = 1.8 Hz), 132.57 (d, *J* = 10.0 Hz), 130.30 (d, *J* = 9.2 Hz), 121.15 (d, *J* = 7.9 Hz), 119.39 (d, *J* = 7.3 Hz), 119.24 (d, *J* = 11.6 Hz), 110.92 (d, *J* = 87.6 Hz), 108.83 (d, *J* = 14.5 Hz), 107.93 (d, *J* = 91.8 Hz), 39.66 (d, *J* = 70.0 Hz), 36.11, 34.16, 27.20, 27.10. **^{31}P NMR** (162 MHz, Chloroform-*d*) δ 56.38. **HRMS (ESI)** calculated for $[C_{22}H_{25}BrO_3P+H]^+$: 449.0699, found:449.0702. **HPLC separation** (Chiralpak IA, 4.6 x 250mm; *i*-PrOH / hexane = 1 / 19, 1.0 mL/min, 210 nm; tr (major) = 7.3 min, tr (minor) = 8.0 min, 94 : 6 e.r.).

(R)-2-((R)-((1s,3R,5R,7S)-adamantan-1-yl)(5-bromo-2-hydroxyphenyl)phosphoryl)phenyl 2-(diphenylphosphanyl)benzoate

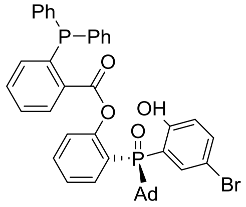

scale (0.1 mmol), product: weight of compound (734.1350), yield (68.2 mg, 0.093 mmol, 93%), white solid. **^{1}H NMR** (400 MHz, Chloroform-*d*) δ 11.39 (s, 1H), 8.15 (ddd, *J* = 10.7, 8.4, 3.1 Hz, 2H), 7.54 (dd, *J* = 8.7, 2.4 Hz, 1H), 7.52 – 7.42 (m, 2H), 7.42 – 7.17 (m, 9H), 7.20 – 7.11 (m, 1H), 7.00 – 6.90 (m, 1H), 6.75 (dd, *J* = 8.4, 4.4 Hz, 1H), 6.66 (dd, *J* = 8.7, 4.3 Hz, 1H), 6.58 (dt, *J* = 8.0, 3.9 Hz, 1H), 2.04 (m, 9H), 1.80 – 1.64 (m, 7H). ^{13}C NMR (101 MHz, Chloroform-*d*) δ 164.53, 152.01, 141.89 (d, *J* = 29.0 Hz), 137.73 (d,

J = 18.5 Hz), 136.31 – 135.84 (m), 134.32, 134.13 (d, *J* = 2.8 Hz), 134.03 (d, *J* = 2.0 Hz), 133.94, 132.91, 132.13 (d, *J* = 2.2 Hz), 131.19 (d, *J* = 10.0 Hz), 128.99 – 128.21 (m), 126.55 (d, *J* = 6.1 Hz), 125.08 (d, *J* = 81.9 Hz), 124.66, 119.13 – 117.68 (m), 109.23 (d, *J* = 94.3 Hz), 39.31 (d, *J* = 71.9 Hz), 36.44, 35.17 (d, *J* = 2.0 Hz), 27.60, 27.50. **^{31}P NMR** (162 MHz, Chloroform-*d*) 45.45, -4.47. **HRMS (ESI)** calculated for $[C_{41}H_{37}BrO_4P_2+H]^+$: 735.1423, found: 735.1428. **HPLC separation** (Chiralpak AD, 4.6 x 250mm; *i*-PrOH / hexane = 1 / 4, 1.0 mL/min, 210 nm; tr (major) = 8.3 min, tr (minor) = 12.0 min, 94.5 : 5.5 e.r.).

Benzyl 2-(((1-(2-(((3r)-adamantan-1-yl)(phenyl)phosphoryl)phenyl)naphthalen-2-yl)oxy)methyl)acrylate

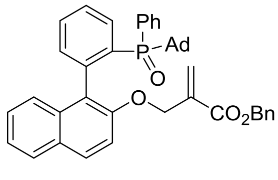

scale (0.1 mmol), product: weight of compound (652.2742), yield (26.1 mg, 0.040 mmol, 40%), semi-solid. **^{1}H NMR** (400 MHz, Chloroform-d) δ 8.24 – 8.15 (m, 1H), 7.75 (d, *J* = 8.9 Hz, 1H), 7.70 – 7.65 (m, 1H), 7.59 – 7.50 (m, 2H), 7.46 (dd, *J* = 10.1, 7.8 Hz, 2H), 7.41 – 7.30 (m, 5H), 7.27 – 7.17 (m, 4H), 7.14 –7.08 (m, 2H), 7.03 (dt, *J* = 7.7, 3.8 Hz, 2H), 5.98 (d, *J* = 2.0 Hz, 1H), 5.20 (s, 1H), 4.65 – 4.29 (m, 2H), 1.87 – 7.85 – 1.78 (m, 9H), 1.62 – 1.54 (m, 6H). **^{13}C NMR** (101 MHz, Chloroform-*d*) δ 165.08, 152.20, 142.27 (d, *J* = 5.8 Hz), 135.78, 135.45, 133.91 (d, *J* = 1.6 Hz), 133.82, 132.91 (d, *J* = 10.1 Hz), 131.94, 131.86, 131.70 (d, *J* = 87.9 Hz), 131.16 (d, *J* = 89.9 Hz), 130.36 (d, *J* = 2.7 Hz), 129.42, 128.68, 128.62, 128.35, 128.14, 127.58, 127.20 (d, *J* = 10.9 Hz), 126.25 (d, *J* = 10.9 Hz), 125.88, 125.64 (d, *J* = 10.1 Hz), 124.86 (d, *J* = 2.7 Hz), 123.20, 113.14, 66.37, 65.84, 38.39 (d, *J* = 71.4 Hz), 36.47, 36.45, 35.66, 35.64, 27.75, 27.65. **^{31}P NMR** (162 MHz, Chloroform-*d*) 33.48. **HRMS (ESI)** calculated for $[C_{43}H_{41}O_4P+Na]^+$: 675.2635, found: 675.2638. **HPLC separation** (Chiralpak IA, 4.6 x 250mm; *i*-PrOH / hexane = 1 / 2, 1.0 mL/min, 210 nm; tr (major) = 10.3 min, tr (minor) = 14.1 min, 64.5 : 35.5 e.r.).

benzyl 2-((2-((R)-((1s,3R,5R,7S)-adamantan-1-yl)(3-bromo-2-hydroxy-5-methylphenyl)phosphoryl)-4-methylphenoxy)methyl)acrylate

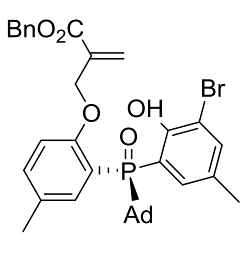

^{1}H NMR (400 MHz, Chloroform-d) δ 12.76 (s, 1H), 7.97 (dd, *J* = 12.7, 2.3 Hz, 1H), 7.48 – 7.32 (m, 7H), 7.32 – 7.24 (m, 1H), 6.81 (dd, *J* = 8.4, 5.6 Hz, 1H), 6.43 (d, *J* = 1.1 Hz, 1H), 5.76 (d, *J* = 1.1 Hz, 1H), 5.26 (d, *J* = 1.2 Hz, 2H), 5.01 – 4.74 (m, 2H), 2.35 (s, 3H), 2.20 (s, 3H), 2.15 – 1.94 (m, 9H), 1.80 – 1.66 (m, 6H). **^{13}C NMR** (101 MHz, Chloroform-*d*) δ164.93, 158.22 (d, *J* = 3.1 Hz), 156.36 (d, *J* = 4.3 Hz), 137.44 (d, *J* = 2.5 Hz), 135.90 (d, *J* = 5.2 Hz), 135.39, 135.02, 134.64 (d, *J* = 2.5 Hz), 131.12, 131.02, 130.99, 130.89, 128.67 128.51, 128.28, 128.04, 127.91, 117.89 (d, *J* = 87.0 Hz), 112.48 (d, *J* = 7.2 Hz), 111.84 (d, *J* = 10.5 Hz), 111.56 (d, *J* = 89.9 Hz), 66.96 (d, *J* = 12.3 Hz), 39.44, 36.41 (d, *J* = 1.8 Hz), 35.28, 27.73, 27.62, 20.52, 20.34. **^{31}P NMR** (162 MHz, Chloroform-*d*) δ 50.60. **HPLC separation** (Chiralpak AD, 4.6 x 250mm; *i*-PrOH / hexane = 1 / 4, 1.0 mL/min, 210 nm; tr (major) = 14.8 min, tr (minor) = 27.1 min, 96 : 4 e.r.).

(S)-((1s,3R,5S,7S)-adamantan-1-yl)(3-bromo-2-hydroxy-5-methylphenyl)(2-hydroxy-5-methylphenyl)phosphine oxide

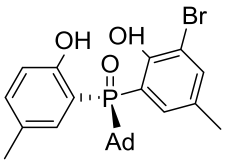

^{1}**H NMR** (400 MHz, Chloroform-d) δ 11.79 (s, 1H), 10.87 (s, 1H), 7.53 (d, *J* = 2.0 Hz, 1H), 7.26 – 7.05 (m, 3H), 6.85 (dd, *J* = 8.5, 4.7 Hz, 1H), 2.30 (d, *J* = 4.6 Hz, 6H), 2.02 (m, 9H), 1.84 – 1.63 (m, 6H).^{3}**C NMR** (101 MHz, Chloroform-*d*) δ 162.27, 158.10, 138.41, 135.56, 130.09 (d, *J* = 8.8 Hz), 129.79 (d, *J* = 8.8 Hz), 128.03 (d, *J* = 11.1 Hz), 119.20 (d, *J* = 7.5 Hz), 112.73, 110.05 (d, *J* = 87.1 Hz), 107.69 (d, *J* = 90.3 Hz), 39.66 (d, *J* = 69.6 Hz), 36.18, 34.34, 27.30, 27.19, 20.84, 20.54.^{31}**P NMR** (162 MHz, Chloroform-*d*) δ 57.0.

2-((R)-((1s,3R,5R,7S)-adamantan-1-yl)(3-bromo-2-hydroxy-5-methylphenyl)phosphoryl)-4-methylphenyl 2-(diphenylphosphoryl)benzoate

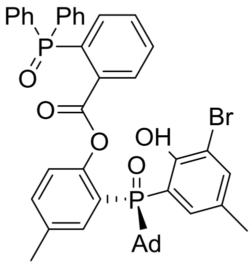

^{1}**H NMR** (400 MHz, Chloroform-d) δ 12.13 (s, 1H), 8.08 (dd, *J* = 7.9, 3.6 Hz, 1H), 7.77 – 7.64 (m, 2H), 7.57 (m, 6H), 7.47 (t, *J* = 7.5 Hz, 2H), 7.43 – 7.28 (m, 6H), 7.17 (dd, *J* = 8.5, 4.2 Hz, 1H), 6.61 (d, *J* = 12.0 Hz, 1H), 2.37 (s, 3H), 2.01 (m, 12H), 1.69 (m, 6H). ^{13}**C NMR** (101 MHz, Chloroform-*d*) δ165.37 (d, *J* = 3.3 Hz), 158.09 (d, *J* = 2.8 Hz), 151.68 , 137.64 (d, *J* = 2.3 Hz), 135.20 (d, *J* = 5.7 Hz), 134.86 (d, *J* = 10.5 Hz), 134.56 (d, *J* = 2.3 Hz), 134.18 (d, *J* = 10.0 Hz), 133.39 (d, *J* = 2.0 Hz), 132.48, 132.37, 132.30, 132.18 (d, *J* = 2.4 Hz), 131.94 (d, *J* = 4.0 Hz), 131.84 (d, *J* = 4.0 Hz), 131.51 (d, *J* = 2.7 Hz), 131.03 (d, *J* = 8.2 Hz), 130.88 (d, *J* = 11.7 Hz), 130.41 (d, *J* = 9.4 Hz), 128.35 (d, *J* = 3.8 Hz), 128.23 (d, *J* = 3.9 Hz), 125.56 (d, *J* = 6.1 Hz), 121.17 (d, *J* = 87.7 Hz), 111.92 (d, *J* = 10.5 Hz), 111.15 (d, *J* = 90.1 Hz), 39.09 (d, *J* = 71.8 Hz), 36.31 (d, *J* = 1.7 Hz), 35.00 (d, *J* = 2.1 Hz), 27.46, 27.35, 21.23, 20.22. ^{31}**P NMR** (162 MHz, Chloroform-*d*) δ 46.4, 31.7. **HRMS (ESI)** calculated for $[C_{43}H_{41}BrO_5P_2+Na]^+$: 801.1505, found: 801.1508. **HPLC separation** (Chiralpak AD, 4.6 x 250mm; *i*-PrOH / hexane = 1 / 2, 1.0 mL/min, 210 nm; tr (major) = 9.3 min, tr (minor) = 70.2 min, 96.5 : 3.5 e.r.).

6.2 Characterization of novel substrates.

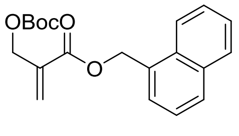

^{1}H NMR (400 MHz, Chloroform-d) δ 8.12 – 7.97 (m, 1H), 7.94 – 7.84 (m, 2H), 7.61 – 7.37 (m, 4H), 6.40 (s, 1H), 5.88 (s, 1H), 5.67 (s, 2H), 4.81 (s, 2H), 1.46 (s, 9H). ^{13}C NMR (101 MHz, Chloroform-d) δ 165.04, 153.12 , 135.11, 133.79, 131.71, 131.16, 129.48, 128.83, 128.15, 127.56, 126.74, 126.06, 125.34, 123.57, 82.59, 65.22, 64.82, 27.82.

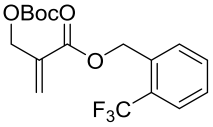

^{1}H NMR (400 MHz, Chloroform-d) δ 7.69 (d, *J* = 7.8 Hz, 1H), 7.57 (d, *J* = 4.3 Hz, 2H), 7.45 (dq, *J* = 8.4, 4.3 Hz, 1H), 6.45 (s, 1H), 5.94 (s, 1H), 5.42 (s, 2H), 4.83 (s, 2H), 1.49 (s, 9H). ^{13}C NMR (101 MHz, Chloroform-d) δ 164.72, 153.15, 134.92, 133.94, 132.24, 130.17, 128.55(d, *J* = 31.3 Hz), 128.53, 128.51, 126.33 (d, *J* = 5.6 Hz), 124.23 (d, *J* = 273.7 Hz), 82.71, 64.79, 63.27 (d, *J* = 2.7 Hz), 27.86.

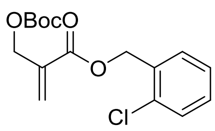

^{1}H NMR (400 MHz, Chloroform-d) δ 7.50 – 7.34 (m, 2H), 7.32 – 7.18 (m, 2H), 6.45 (s, 1H), 5.94 (s, 1H), 5.33 (s, 2H), 4.84 (s, 2H), 1.49 (s, 9H). ^{13}C NMR (101 MHz, Chloroform-d) δ 164.78, 153.11, 134.98, 133.76, 133.39, 129.88, 129.68, 128.30, 126.99, 82.62, 64.78, 64.17, 27.82.

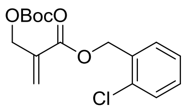

^{1}H NMR (400 MHz, Chloroform-d) δ 7.37 – 7.13 (m, 4H), 6.41 (s, 1H), 5.90 (s, 1H), 5.23 (s, 2H), 4.81 (s, 2H), 2.36 (s, 3H), 1.48 (s, 9H). ^{13}C NMR (101 MHz, Chloroform-d) δ 165.02, 153.15, 137.14, 135.16, 133.64, 130.50, 129.36, 128.73, 128.00, 126.14, 82.63, 65.35, 64.84, 27.85, 19.07.

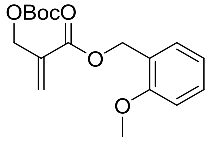

^{1}H NMR (400 MHz, Chloroform-d) δ 7.39 – 7.27 (m, 2H), 6.95 (td, *J* = 7.5, 1.1 Hz, 1H), 6.89 (dd, *J* = 8.1, 1.0 Hz, 1H), 6.41 (d, *J* = 1.1 Hz, 1H), 5.89 (d, *J* = 1.3 Hz, 1H), 5.27 (s, 2H), 4.83 (t, *J* = 1.3 Hz, 2H), 3.84 (s, 3H), 1.48 (s, 9H). ^{13}C NMR (101 MHz, Chloroform-d) δ 165.13, 157.56, 153.18, 135.31, 129.66, 129.52, 127.60, 124.07, 120.50, 110.51, 82.58, 64.90, 62.36, 55.51, 27.85.

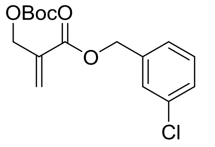

^{1}H NMR (400 MHz, Chloroform-d) δ 7.37 – 7.23 (m, 4H), 6.44 (d, *J* = 1.0 Hz, 1H), 5.94 (d, *J* = 1.2 Hz, 1H), 5.19 (s, 2H), 4.82 (s, 1H), 1.49 (s, 9H). ^{13}C NMR (101 MHz, Chloroform-d) δ 164.85, 153.14, 137.70, 134.97, 134.59, 130.02, 128.59, 128.52, 128.24, 126.25 , 82.72 , 65.91 , 64.79, 27.86.

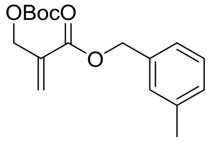

^{1}H NMR (400 MHz, Chloroform-d) δ 7.29 – 7.23 (m, 1H), 7.19 – 7.11 (m, 3H), 6.42 (d, *J* = 1.1 Hz, 1H), 5.90 (d, *J* = 1.2 Hz, 1H), 5.18 (s, 2H), 4.82 (s, 1H), 2.36 (s, 3H), 1.49 (s, 9H). ^{13}C NMR (101 MHz, Chloroform-d) δ 165.04, 153.17, 138.39, 135.62, 135.18, 129.18, 129.03, 128.61, 127.97, 125.36, 82.64, 66.88, 64.85, 27.86, 21.50.

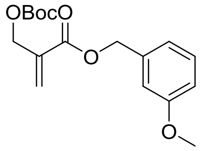

^{1}H NMR (400 MHz, Chloroform-d) δ 7.29 (d, *J* = 7.9 Hz, 1H), 7.00 – 6.93 (m, 1H), 6.93 – 6.82 (m, 2H), 6.43 (d, *J* = 1.1 Hz, 1H), 5.92 (d, *J* = 1.2 Hz, 1H), 5.20 (s, 2H), 4.83 (t, *J* = 1.2 Hz, 2H), 3.82 (s, 3H), 1.49 (s, 9H). ^{13}C NMR (101 MHz, Chloroform-d) δ 164.98, 159.84, 153.16, 137.23, 135.14 , 129.76, 128.15, 120.38, 113.88, 113.65, 82.65, 66.67, 64.85, 55.36, 27.85.

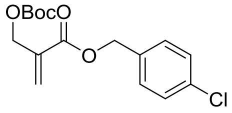

^{1}H NMR (400 MHz, Chloroform-d) δ 7.45 – 7.23 (m, 4H), 6.42 (s, 1H), 5.92 (s, 1H), 5.18 (s, 2H), 4.81 (d, *J* = 1.3 Hz, 2H), 1.49 (s, 9H). ^{13}C NMR (101 MHz, Chloroform-d) δ 164.88, 153.11, 135.00, 134.31, 134.19, 129.66, 128.88, 128.38, 82.69, 65.98, 64.78, 27.83.

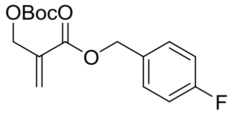

^{1}H NMR (400 MHz, Chloroform-d) δ 7.40 (td, *J* = 7.5, 1.8 Hz, 1H), 7.33 (tdd, *J* = 7.5, 5.3, 1.8 Hz, 1H), 7.14 (td, *J* = 7.5, 1.2 Hz, 1H), 7.08 (ddd, *J* = 9.7, 8.2, 1.1 Hz, 1H), 6.42 (d, *J* = 1.0 Hz, 1H), 5.91 (d, *J* = 1.2 Hz, 1H), 5.29 (s, 2H), 4.81 (t, *J* = 1.3 Hz, 2H), 1.48 (s, 9H). ^{13}C NMR (101 MHz, Chloroform-d) δ 164.88, 161.09 (d, *J* = 248.7 Hz), 153.13, 135.01, 130.64 (d, *J* = 3.7 Hz), 130.41 (d, *J* = 8.2 Hz), 128.19, 124.28 (d, *J* = 3.7 Hz), 122.89 (d, *J* = 14.4 Hz), 115.62 (d, *J* = 21.1 Hz), 82.64, 64.78, 60.81 (d, *J* = 4.3 Hz), 27.83.

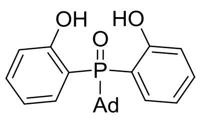

^{1}H NMR (400 MHz, Chloroform-d) δ 11.20 (s, 2H), 7.56 – 7.37 (m, 4H), 7.10 – 6.67 (m, 4H), 2.03 (ddd, *J* = 20.0, 4.9, 2.6 Hz, 9H), 1.86 – 1.62 (m, 6H). ^{13}C NMR (101 MHz, Chloroform-d) δ 164.43 (d, *J* = 2.0 Hz), 134.47 (d, *J* = 2.2 Hz), 130.73 (d, *J* = 9.1 Hz), 119.27 (d, *J* = 7.1 Hz), 119.07 (d, *J* =

11.3 Hz), 108.79 (d, *J* = 90.6 Hz), 39.62 (d, *J* = 70.0 Hz), 36.30 (d, *J* = 1.7 Hz), 34.32 (d, *J* = 1.7 Hz), 27.37, 27.27. ^{31}P NMR (162 MHz, Chloroform-d) δ 57.22.

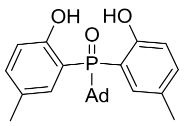

^{1}H NMR (400 MHz, Chloroform-d) δ 11.04 (s, 2H), 7.29 – 7.10 (m, 4H), 6.85 (dd, *J* = 8.7, 4.6 Hz, 2H), 2.31 (s, 6H), 2.02 (m, 9H), 1.79 – 1.68 (m, 6H). ^{13}C NMR (101 MHz, Chloroform-d) δ 162.27 (d, *J* = 1.9 Hz), 130.39 (d, *J* = 9.1 Hz), 128.01 (d, *J* = 11.2 Hz), 119.03 (d, *J* = 7.8 Hz), 108.40 (d, *J* = 90.2 Hz), 39.56 (d, *J* = 70.0 Hz), 36.32 (d, *J* = 1.7 Hz), 34.37 (d, *J* = 1.8 Hz), 27.39, 27.29, 20.97. ^{31}P NMR (162 MHz, Chloroform-d) δ 56.85.

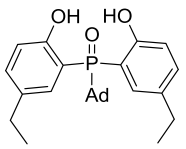

^{1}H NMR (400 MHz, Chloroform-d) δ 11.03 (s, 2H), 7.25 (d, *J* = 9.4 Hz, 4H), 6.87 (dd, *J* = 8.3, 4.6 Hz, 2H), 2.61 (q, *J* = 7.6 Hz, 4H), 2.23 – 1.83 (m, 9H), 1.72 (q, *J* = 12.8 Hz, 6H), 1.22 (t, *J* = 7.6 Hz, 6H). ^{13}C NMR (101 MHz, Chloroform-d) δ 162.53, 134.38 (d, *J* = 10.7 Hz), 134.27, 129.28 (d, *J* = 8.9 Hz), 119.10 (d, *J* = 7.6 Hz), 108.52 (d, *J* = 90.5 Hz), 39.63 (d, *J* = 69.8 Hz), 36.41, 34.49, 28.17, 27.48, 27.38, 15.79. ^{31}P NMR (162 MHz, Chloroform-d) δ 56.95.

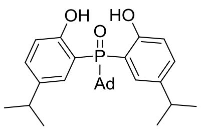

^{1}H NMR (400 MHz, Chloroform-d) δ 11.05 (s, 2H), 7.42 – 7.17 (m, 4H), 6.90 (dd, *J* = 8.9, 4.6 Hz, 2H), 2.89 (p, *J* = 6.9 Hz, 2H), 2.05 (m, 9H), 1.88 – 1.66 (m, 6H), 1.26 (dd, *J* = 6.9, 2.5 Hz, 12H). ^{13}C NMR (101 MHz, Chloroform-d) δ 162.54, 138.99 (d, *J* = 10.9 Hz), 133.03, 127.79 (d, *J* = 8.8 Hz), 118.98 (d, *J* = 7.6 Hz), 108.45 (d, *J* = 90.4 Hz), 39.58 (d, *J* = 70.0 Hz), 36.42, 34.53, 33.33, 27.50, 27.39, 24.31, 24.22. ^{31}P NMR (162 MHz, Chloroform-d) δ 57.06.

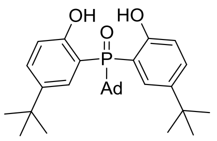

^{1}H NMR (400 MHz, Chloroform-d) δ 11.03 (s, 2H), 7.57 – 7.34 (m, 4H), 6.89 (dd, *J* = 9.1, 4.7 Hz, 2H), 2.29 – 1.87 (m, 9H), 1.72 (m, 6H), 1.30 (s, 18H). ^{13}C NMR (101 MHz, Chloroform-d) δ 162.15, 141.35 (d, *J* = 10.0 Hz), 131.82, 126.82 (d, *J* = 9.8 Hz), 118.71 (d, *J* = 7.7 Hz), 108.04 (d, *J* = 90.6 Hz), 39.51 (d, *J* = 69.7 Hz), 36.42, 34.63, 34.24, 31.58, 27.51, 27.41. ^{31}P NMR (162 MHz, Chloroform-d) δ 57.43.

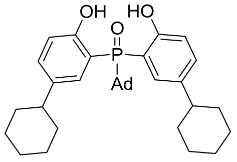

^{1}H NMR (400 MHz, Chloroform-d) δ 11.01 (s, 2H), 7.39 – 7.02 (m, 4H), 6.86 (dd, *J* = 8.5, 4.6 Hz, 2H), 2.45 (tt, *J* = 11.6, 3.2 Hz, 2H), 2.03 (m, 9H), 1.93 – 1.81 (m, 8H), 1.81 – 1.62 (m, 8H), 1.51 – 1.13 (m, 10H). ^{13}C NMR (101 MHz, Chloroform-d) δ 162.50, 138.23 (d, *J* = 10.1 Hz), 133.43, 128.16 (d, *J* = 9.1 Hz), 118.83 (d, *J* = 7.6 Hz), 108.44 (d, *J* = 90.4 Hz), 43.50, 39.54 (d, *J* = 69.9 Hz), 36.40, 35.03, 34.87, 34.46, 27.46, 27.35, 26.95, 26.93, 26.23. ^{31}P NMR (162 MHz, Chloroform-d) δ 57.06.

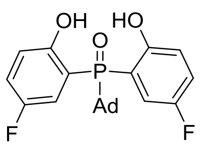

^{1}H NMR (400 MHz, Chloroform-d) δ 10.90 (s, 2H), 7.19 (td, *J* = 8.5, 3.0 Hz, 2H), 7.10 (ddd, *J* = 11.4, 8.3, 3.1 Hz, 2H), 6.95 (dt, *J* = 9.4, 4.8 Hz, 2H), 2.06 (m, 9H), 1.85 – 1.68 (m, 6H). ^{13}C NMR (101 MHz, Chloroform-d) δ 160.66, 155.21 (dd, *J* = 240.2, 15.8 Hz), 122.15 (dd, *J* = 23.0, 2.3 Hz), 121.61 – 120.18, 115.94 (dd, *J* = 23.9, 10.1 Hz), 108.35 (dd, *J* = 90.1, 5.2 Hz), 39.81 (d, *J* = 70.2 Hz), 36.19 (d, *J* = 1.8 Hz), 34.30 (d, *J* = 1.9 Hz), 27.29, 27.18. ^{31}P NMR (162 MHz, Chloroform-d) δ 55.21.

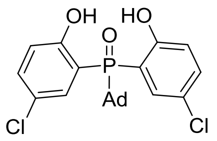

^{1}H NMR (400 MHz, Chloroform-d) δ 11.08 (s, 2H), 7.39 (dd, *J* = 8.9, 2.5 Hz, 2H), 7.32 (dd, *J* = 10.9, 2.5 Hz, 2H), 6.91 (dd, *J* = 8.9, 4.8 Hz, 2H), 2.04 (m, 9H), 1.88 – 1.64 (m, 6H). ^{13}C NMR (101 MHz, Chloroform-d) δ 163.08 (d, *J* = 1.8 Hz), 134.82 (d, *J* = 2.2 Hz), 129.49 (d, *J* = 10.0 Hz), 124.22 (d, *J* = 14.7 Hz), 121.06 (d, *J* = 8.0 Hz), 109.59 (d, *J* = 88.7 Hz), 39.88 (d, *J* = 69.7 Hz),

36.16 (d, J = 1.8 Hz), 34.24 (d, J = 2.0 Hz), 27.26, 27.15. ^{31}P NMR (162 MHz, Chloroform-d) δ 55.66.

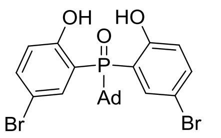

^{1}H NMR (400 MHz, Chloroform-d) δ 11.09 (s, 2H), 7.59 – 7.37 (m, 4H), 6.86 (dt, J = 8.0, 3.4 Hz, 2H), 2.39 – 1.88 (m, 9H), 1.74 (m, 6H). ^{13}C NMR (101 MHz, Chloroform-d) δ 163.51, 137.63 (d, J = 2.1 Hz), 132.40 (d, J = 10.0 Hz), 121.50 (d, J = 7.7 Hz), 111.20 (d, J = 14.1 Hz), 110.30 (d, J = 87.9 Hz), 39.93 (d, J = 69.6 Hz), 36.16, 34.23 (d, J = 1.9 Hz), 27.26, 27.15. ^{31}P NMR (162 MHz, Chloroform-d) δ 55.38.

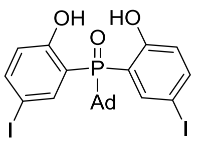

^{1}H NMR (400 MHz, Chloroform-d) δ 11.10 (s, 2H), 7.68 (dd, J = 9.0, 2.0 Hz, 2H), 7.60 (dd, J = 10.6, 2.1 Hz, 2H), 6.74 (dd, J = 8.9, 4.6 Hz, 2H), 2.19 – 1.85 (m, 9H), 1.75 (m, 6H). ^{13}C NMR (101 MHz, Chloroform-d) δ 164.15, 143.29, 138.49 (d, J = 9.8 Hz), 121.97 (d, J = 7.6 Hz), 111.29 (d, J = 87.2 Hz), 80.44 (d, J = 12.9 Hz), 39.99 (d, J = 69.6 Hz), 36.21, 34.28, 27.31, 27.20. ^{31}P NMR (162 MHz, Chloroform-d) δ 54.51.

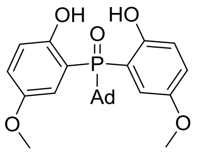

^{1}H NMR (400 MHz, Chloroform-d) δ 10.72 (s, 2H), 7.03 (dd, J = 9.1, 3.0 Hz, 2H), 6.96 (dd, J = 11.7, 3.0 Hz, 2H), 6.90 (dd, J = 9.0, 5.1 Hz, 2H), 3.77 (s, 6H), 2.04 (m, 9H), 1.81 – 1.64 (m, 6H). ^{31}P NMR (162 MHz, Chloroform-d) δ 56.01. ^{13}C NMR (101 MHz, Chloroform-d) δ 158.40, 151.83 (d, J = 14.5 Hz), 120.39, 119.95 (d, J = 8.8 Hz), 115.47 (d, J = 10.6 Hz), 108.72 (d, J = 89.8 Hz), 56.04, 39.71 (d, J = 69.8 Hz), 36.30, 34.48, 27.41, 27.30. ^{31}P NMR (162 MHz, Chloroform-d) δ 56.01.

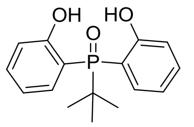

^{1}H NMR (400 MHz, Chloroform-d) δ 11.17 (s, 2H), 7.69 – 7.38 (m, 4H), 6.95 (dt, J = 16.3, 6.5 Hz, 4H), 1.35 (dd, J = 16.0, 2.4 Hz, 9H). ^{13}C NMR (101 MHz, Chloroform-d) δ 164.17 (d, J = 2.0 Hz), 134.59 (d, J = 2.5 Hz), 130.76 (d, J = 9.2 Hz), 119.28 (d, J = 9.1 Hz), 119.19 (d, J = 13.1 Hz), 109.51 (d, J = 90.7 Hz), 36.69 (d, J = 68.6 Hz), 24.23. ^{31}P NMR (162 MHz, Chloroform-d) δ 62.46 .

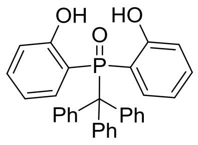

^{1}H NMR (400 MHz, Chloroform-d) δ 11.05 (s, 2H), 7.55 – 7.22 (m, 17H), 6.81 (dd, J = 8.4, 5.0 Hz, 2H), 6.59 (td, J = 7.7, 3.2 Hz, 2H), 6.36 (dd, J = 10.5, 8.3 Hz, 2H). ^{13}C NMR (101 MHz, Chloroform-d) δ 163.55 (d, J = 2.4 Hz), 139.70, 134.69 (d, J = 2.5 Hz), 132.94 (d, J = 8.5 Hz), 131.68 (d, J = 5.9 Hz), 128.20 (d, J = 1.7 Hz), 128.03 (d, J = 2.2 Hz), 119.20 (d, J = 7.8 Hz), 118.56 (d, J = 11.3 Hz), 112.06 (d, J = 91.4 Hz), 67.12 (d, J = 62.4 Hz). ^{31}P NMR (162 MHz, Chloroform-d) δ 53.38.

7. NMR and HPLC spectra

NMR spectra

^{1}H NMR/^{13}C NMR/^{31}P NMR of product 3e

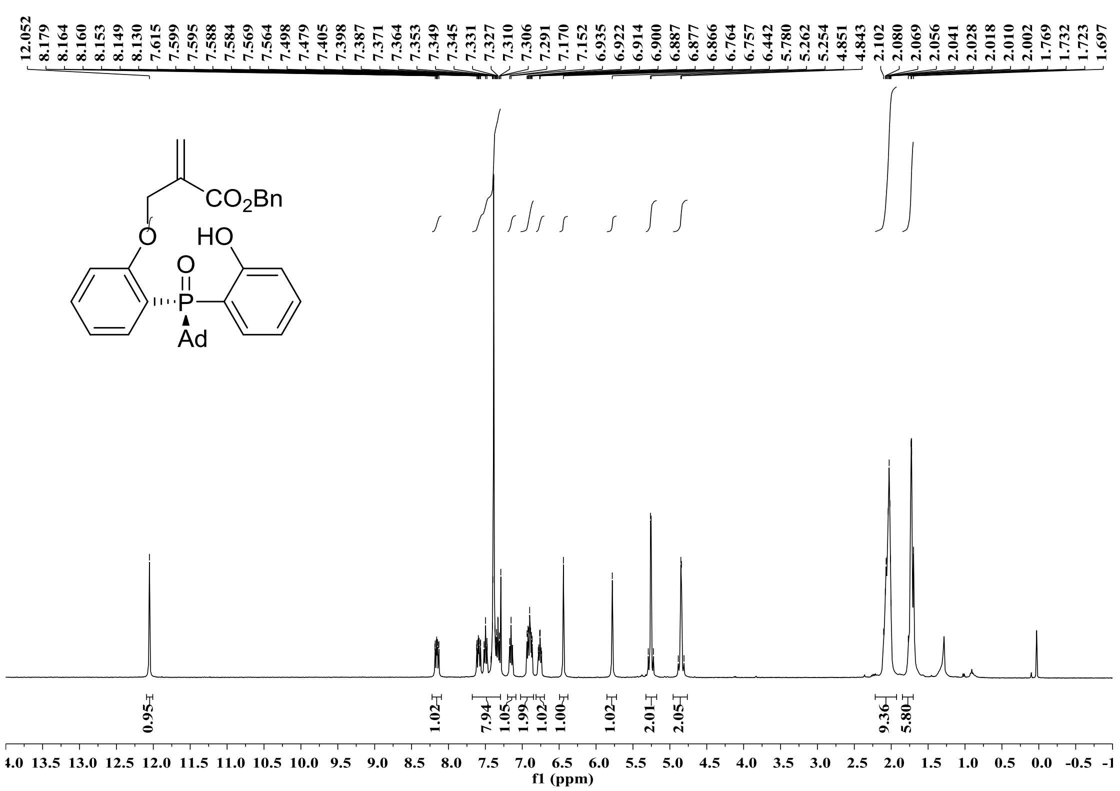
12.052 8.179 8.164 8.160 8.153 8.149 8.130 7.615 7.599 7.595 7.588 7.584 7.569 7.564 7.498 7.479 7.405 7.398 7.387 7.371 7.364 7.353 7.349 7.345 7.331 7.327 7.310 7.306 7.291 7.170 7.152 6.935 6.922 6.914 6.900 6.887 6.877 6.866 6.764 6.757 6.442 5.780 5.262 5.254 4.851 4.843 2.102 2.080 2.069 2.056 2.041 2.028 2.018 2.010 2.002 1.769 1.732 1.723 1.697
CO2Bn
O
HO
O
P
Ad
0.95 1.02 7.94 1.05 1.99 1.02 1.00 1.02 2.01 2.05 9.36 5.80
f1 (ppm)

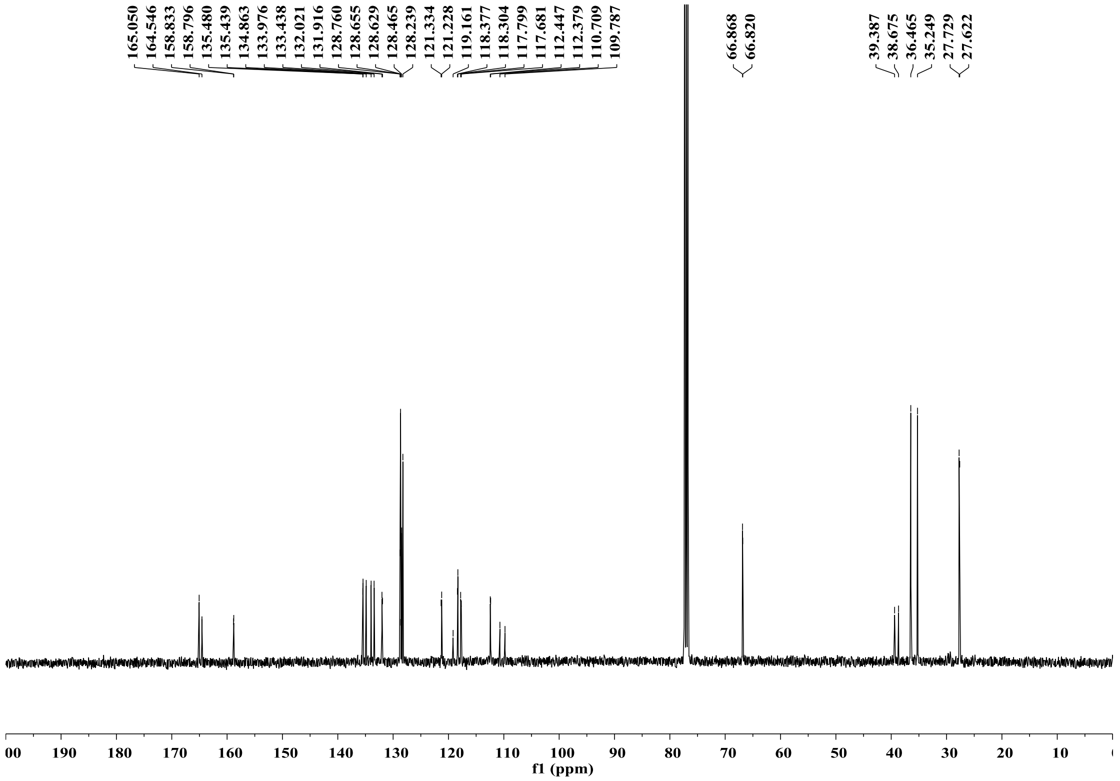
165.050 164.546 158.833 158.796 135.480 135.439 134.863 133.976 133.438 132.021 131.916 128.760 128.655 128.629 128.465 128.239 121.334 121.228 119.161 118.377 118.304 117.799 117.681 112.447 112.379 110.709 109.787
66.868 66.820
39.387 38.675 36.465 35.249 27.729 27.622
f1 (ppm)

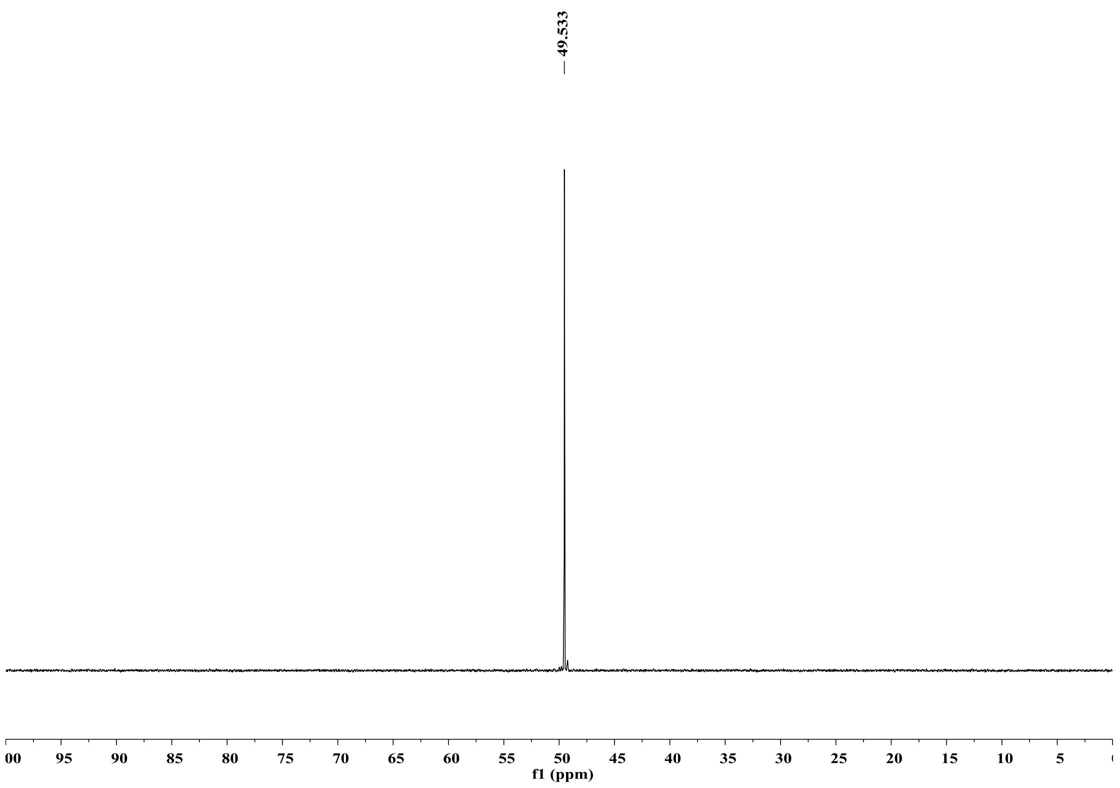

^{1}H NMR/^{13}C NMR/^{31}P NMR of product 3f

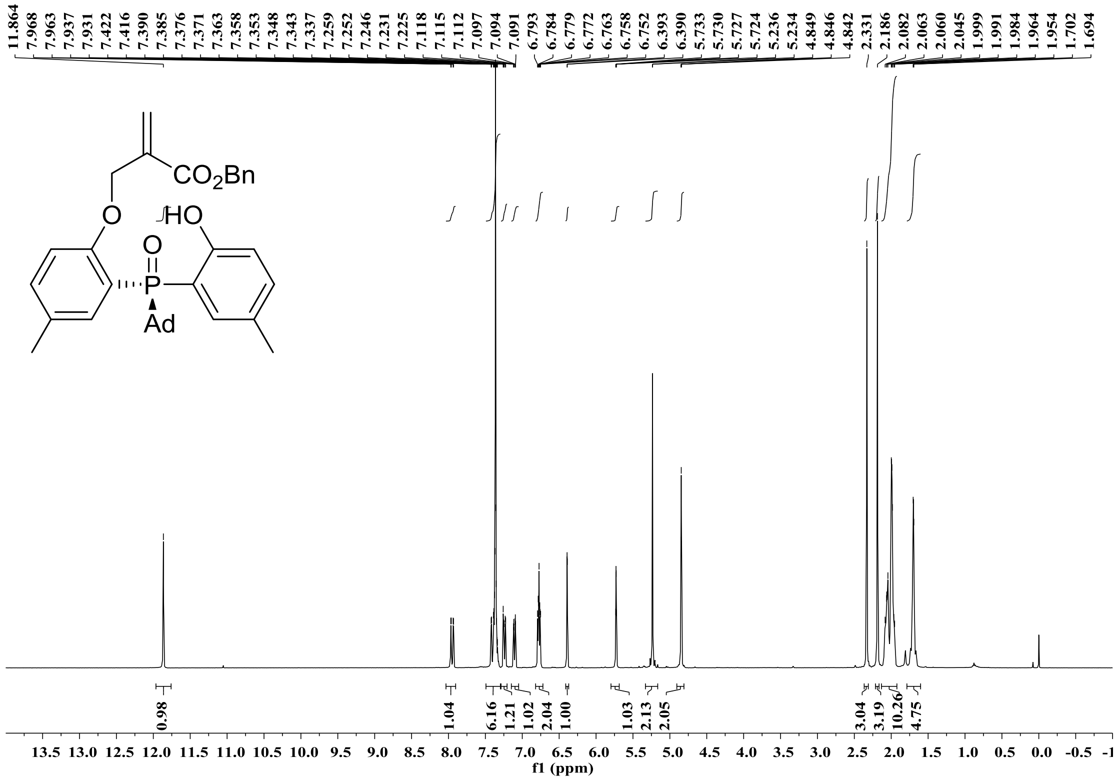

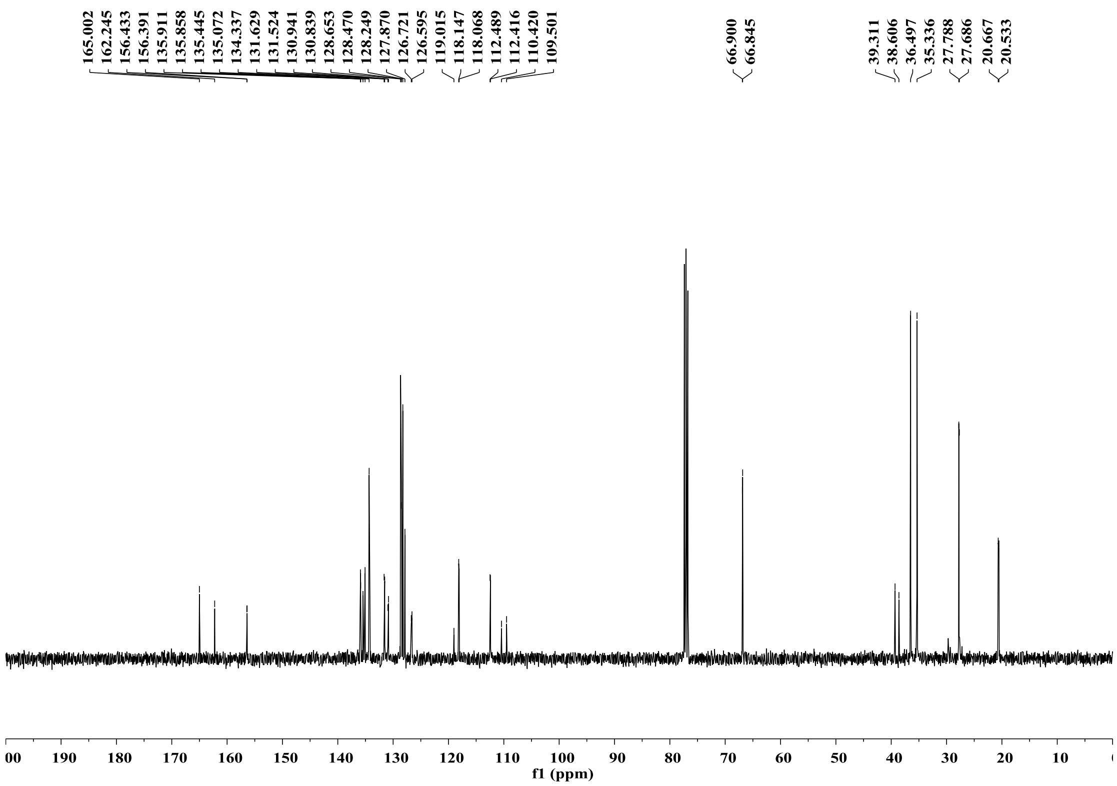

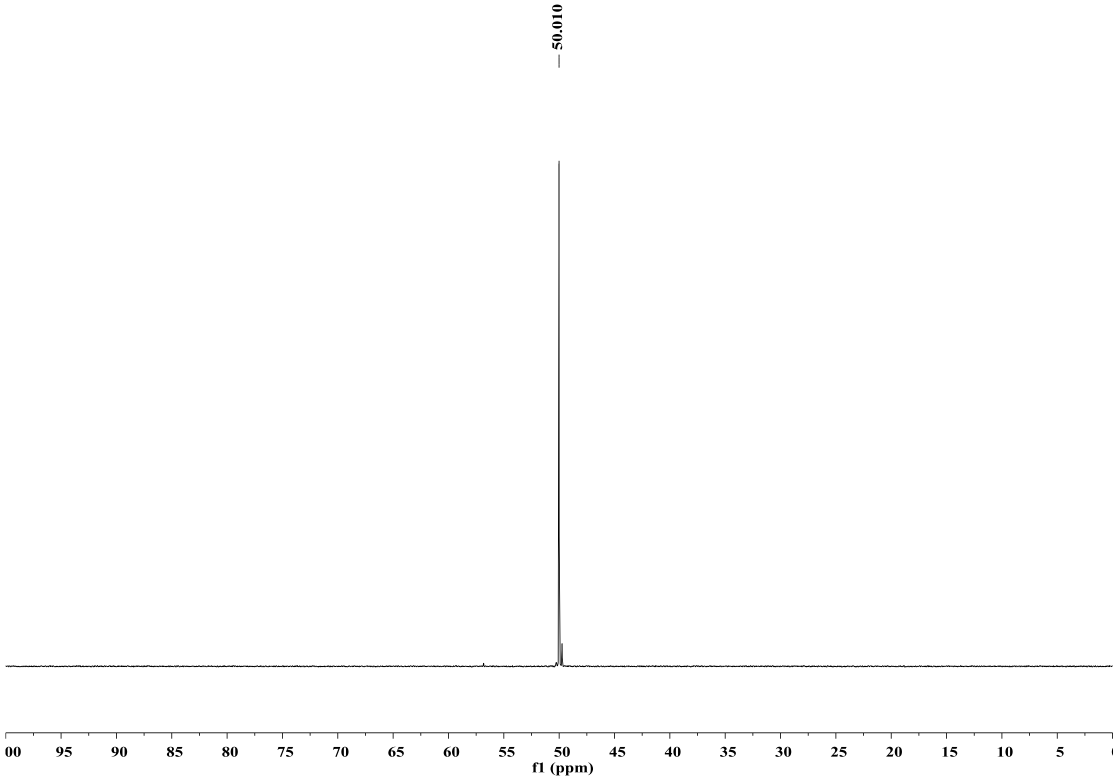

^{1}H NMR/^{13}C NMR/^{31}P NMR of product 3g

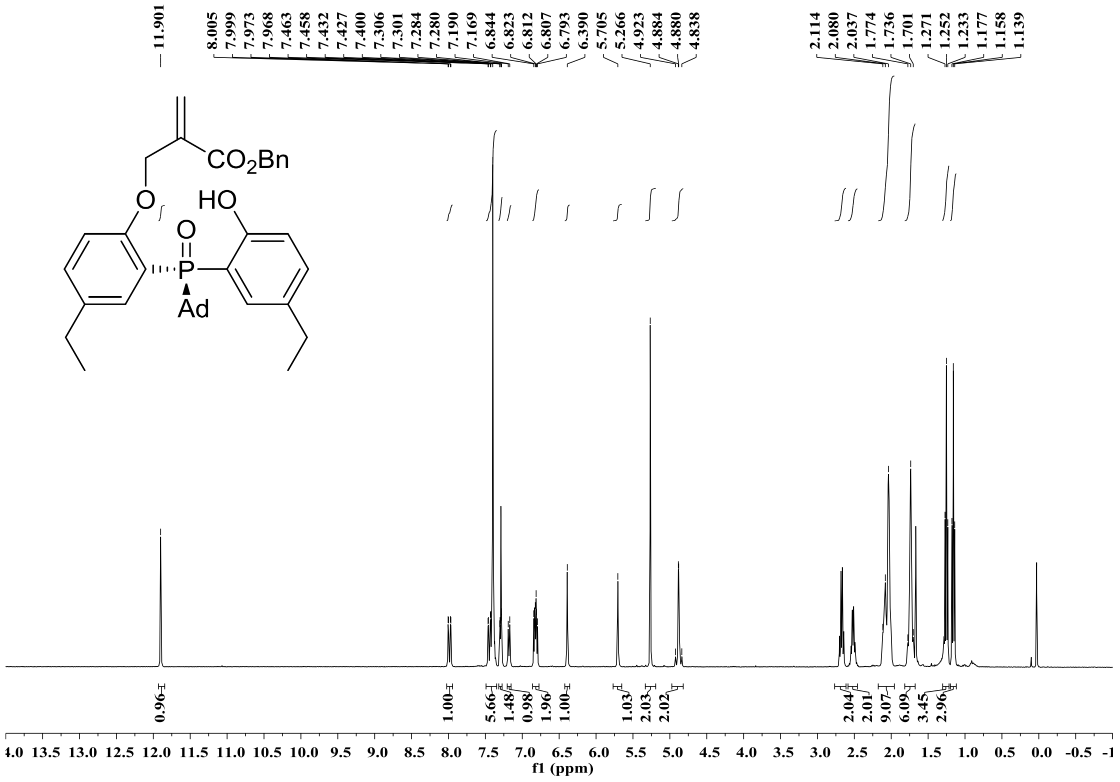
CO2Bn
O
HO
O
P
Ad
f1 (ppm)

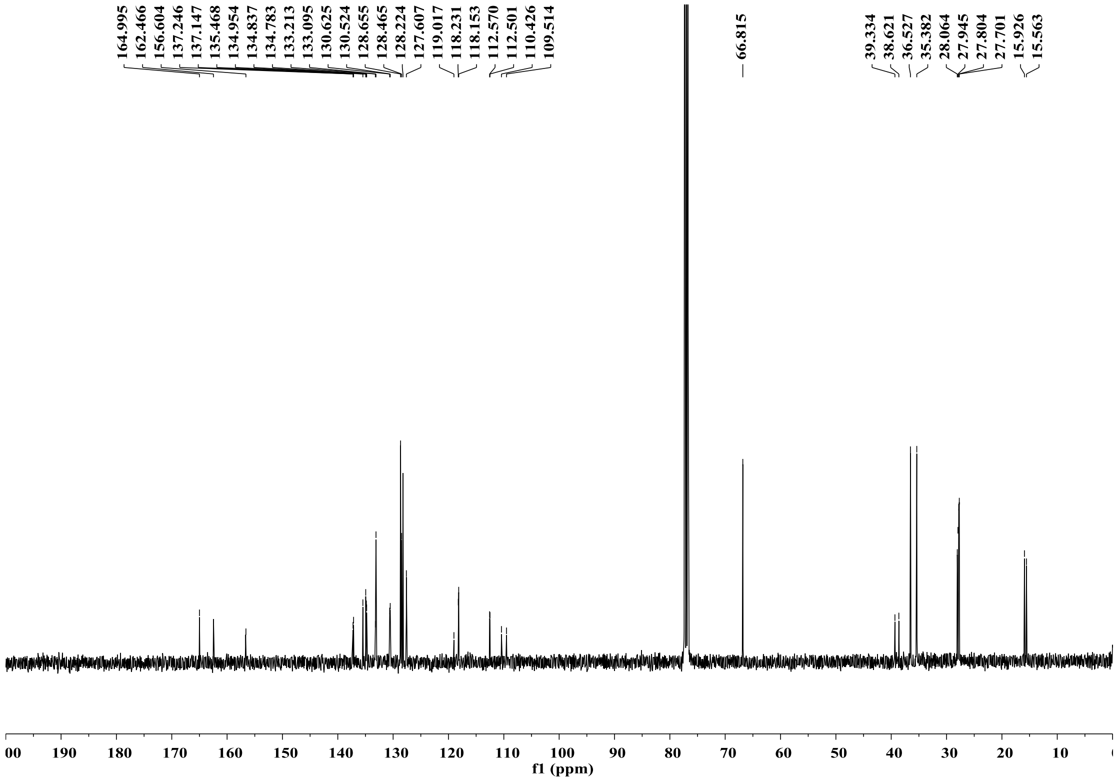
f1 (ppm)

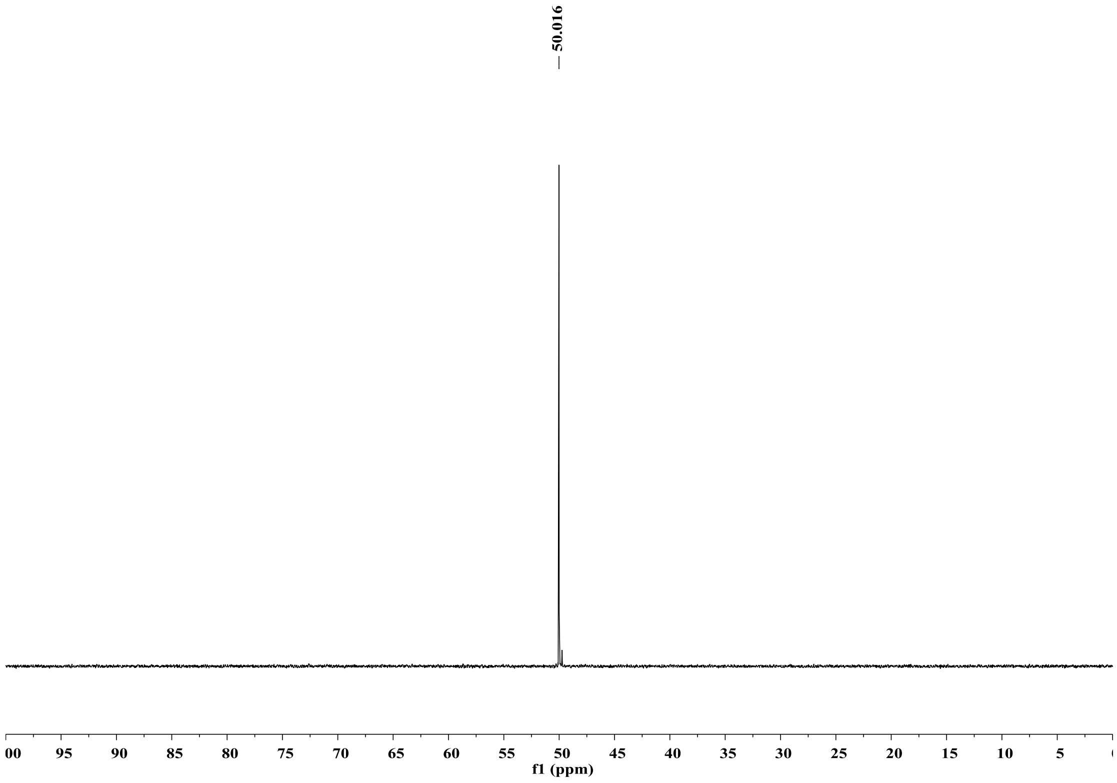

^{1}H NMR/^{13}C NMR/^{31}P NMR of product 3h

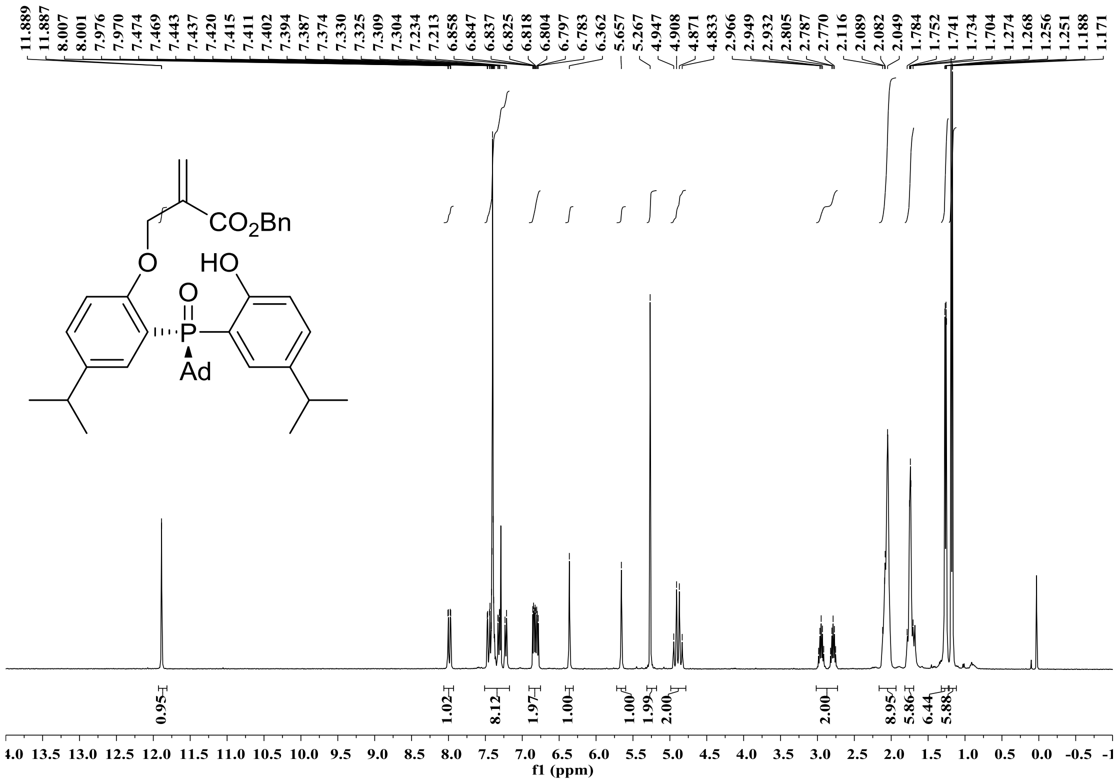

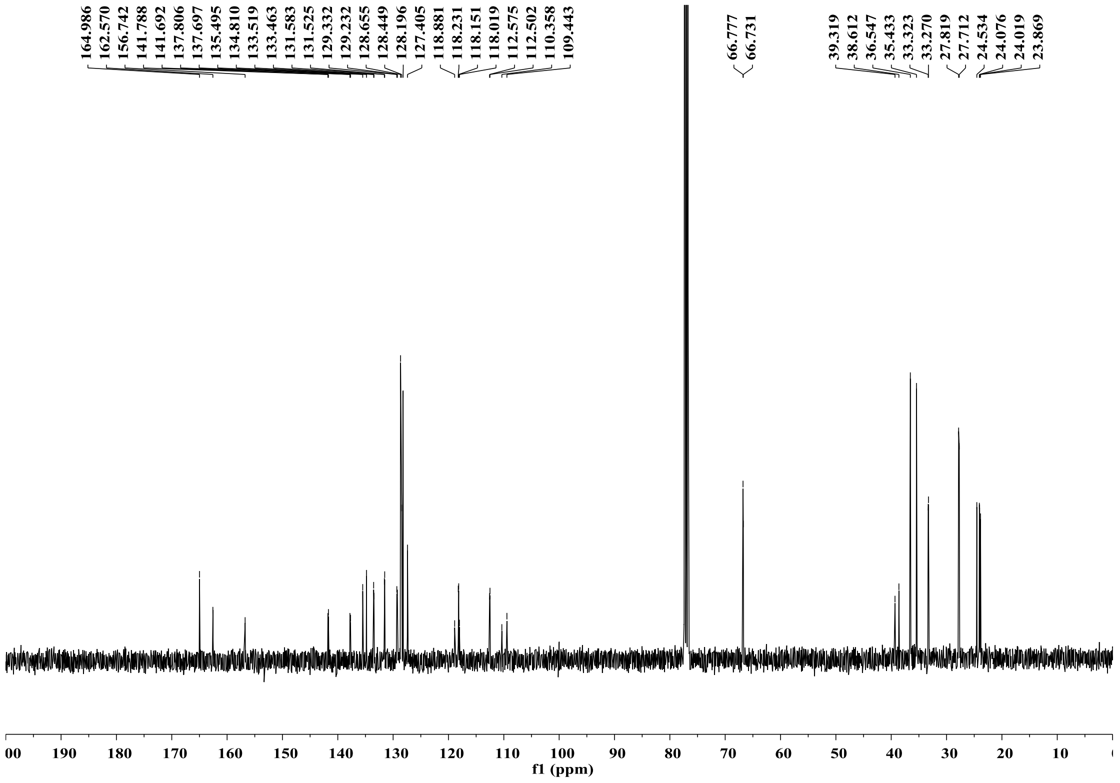
164.986
162.570
156.742
141.788
141.692
137.806
137.697
135.495
134.810
133.519
133.463
131.583
131.525
129.332
129.232
128.655
128.449
128.196
127.405
118.881
118.231
118.151
118.019
112.575
112.502
110.358
109.443
66.777
66.731
39.319
38.612
36.547
35.433
33.323
33.270
27.819
27.712
24.534
24.076
24.019
23.869
f1 (ppm)

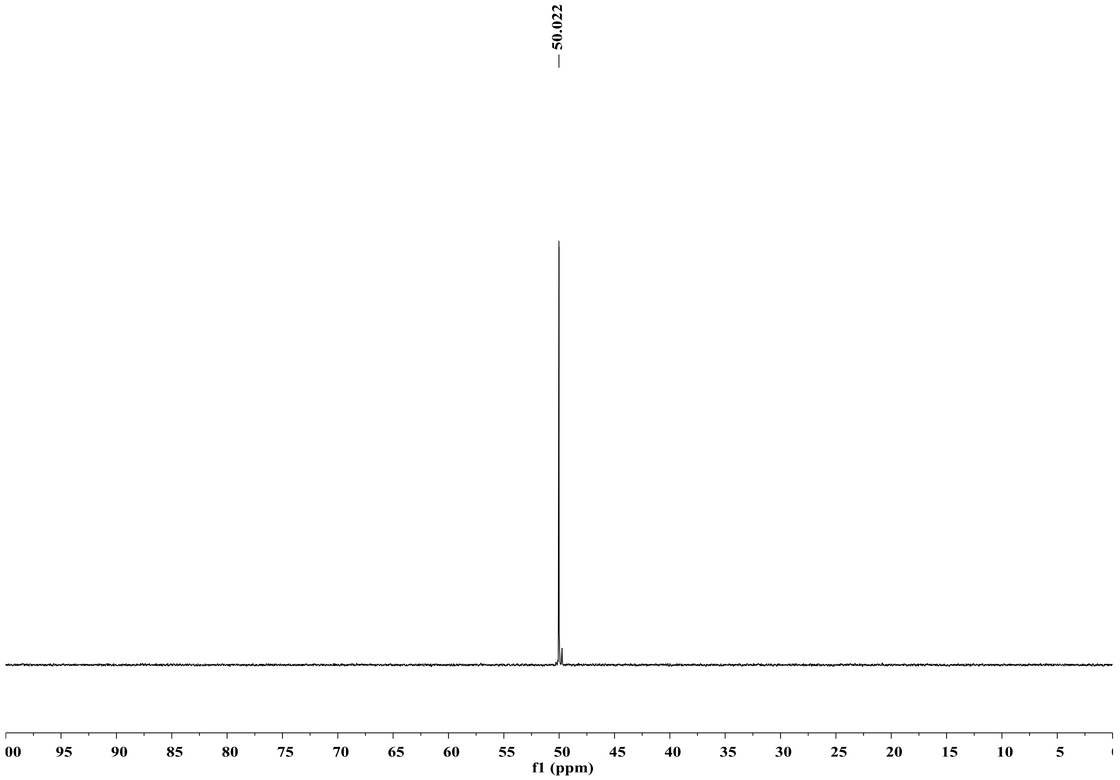
50.022
f1 (ppm)

^{1}H NMR/^{13}C NMR/^{31}P NMR of product 3i

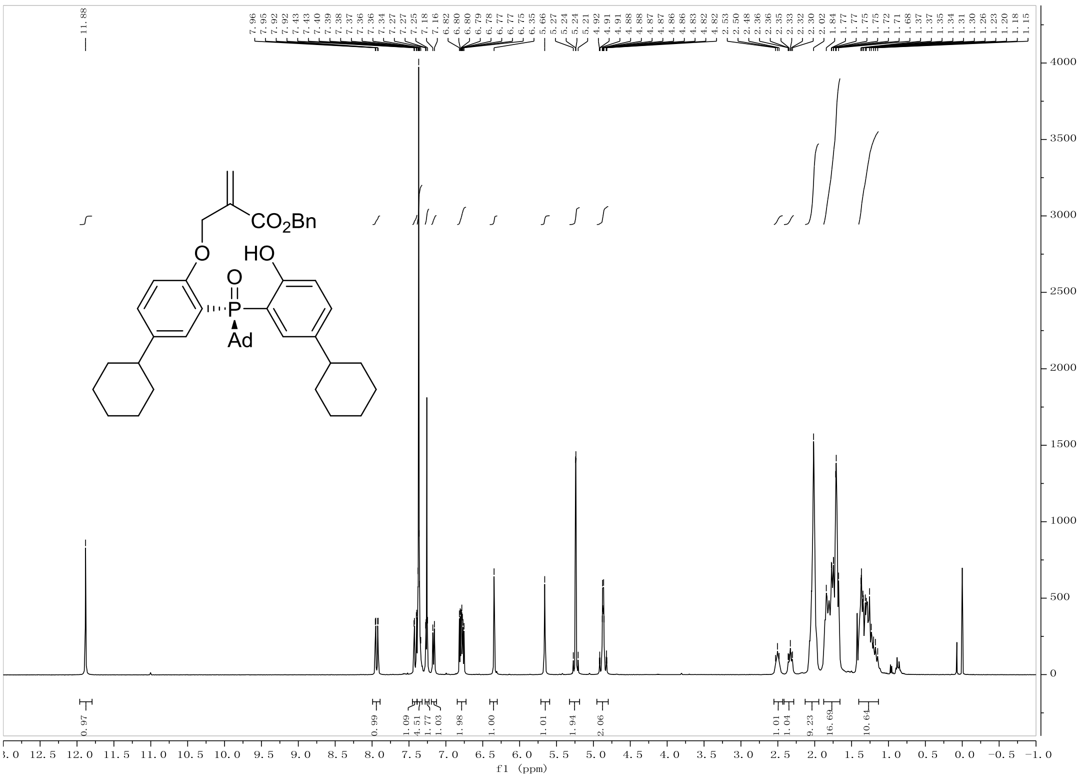

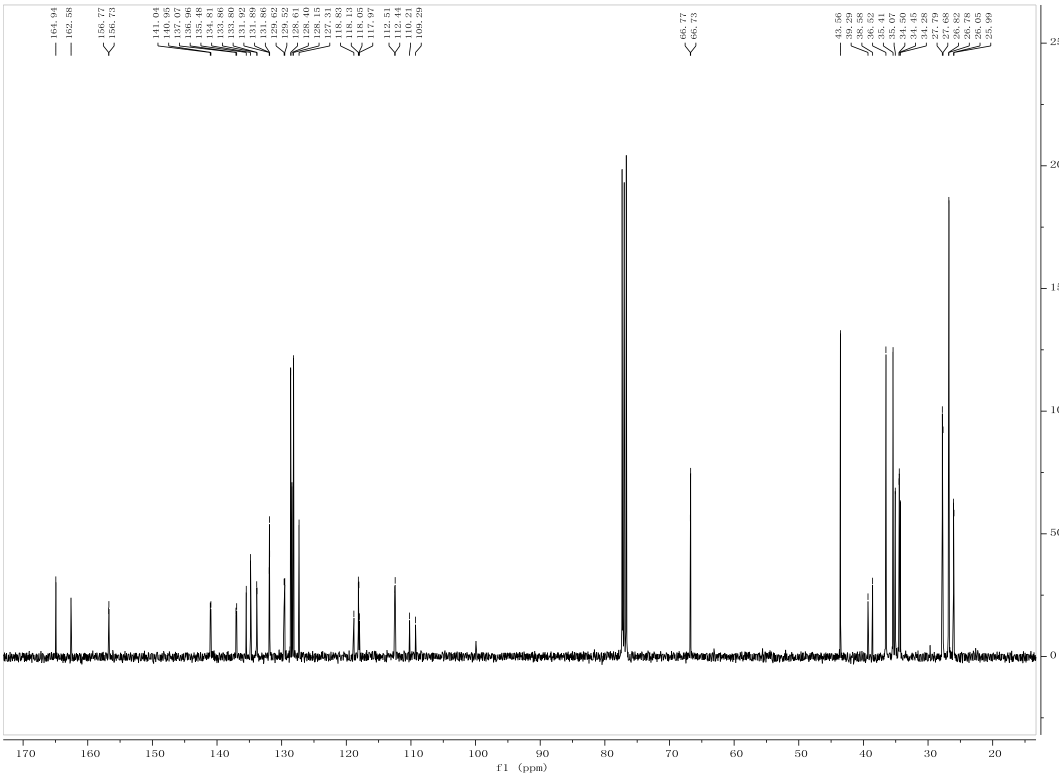

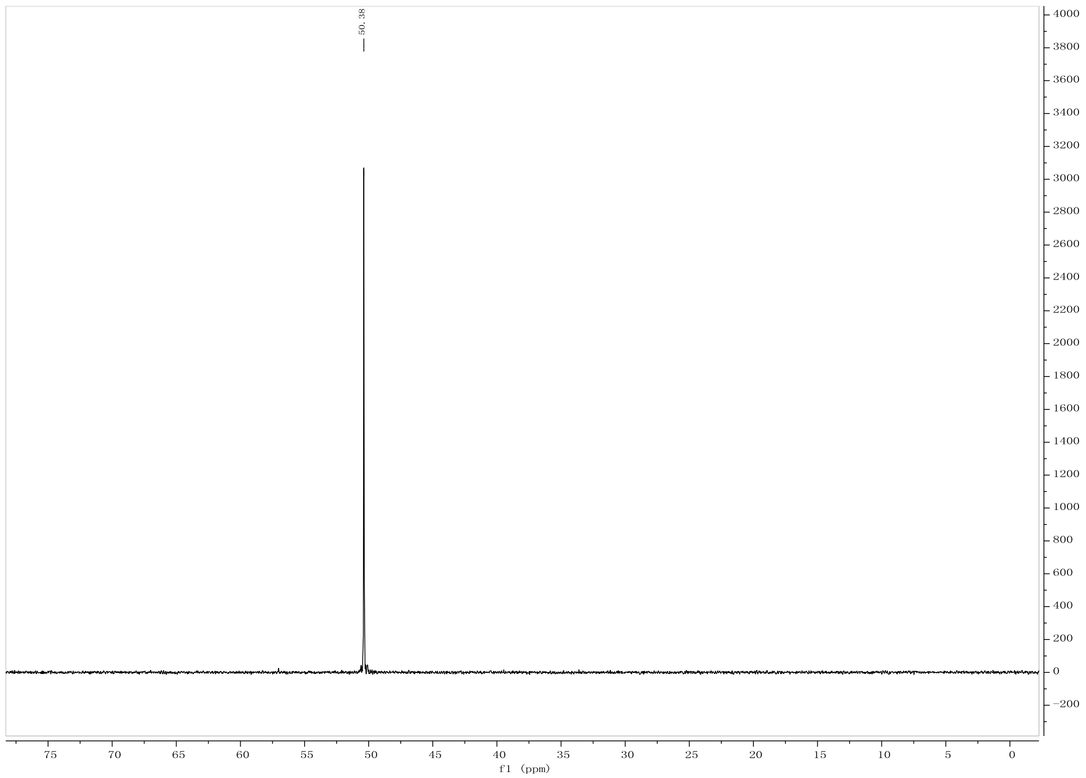

^{1}H NMR/^{13}C NMR/^{31}P NMR of product 3j

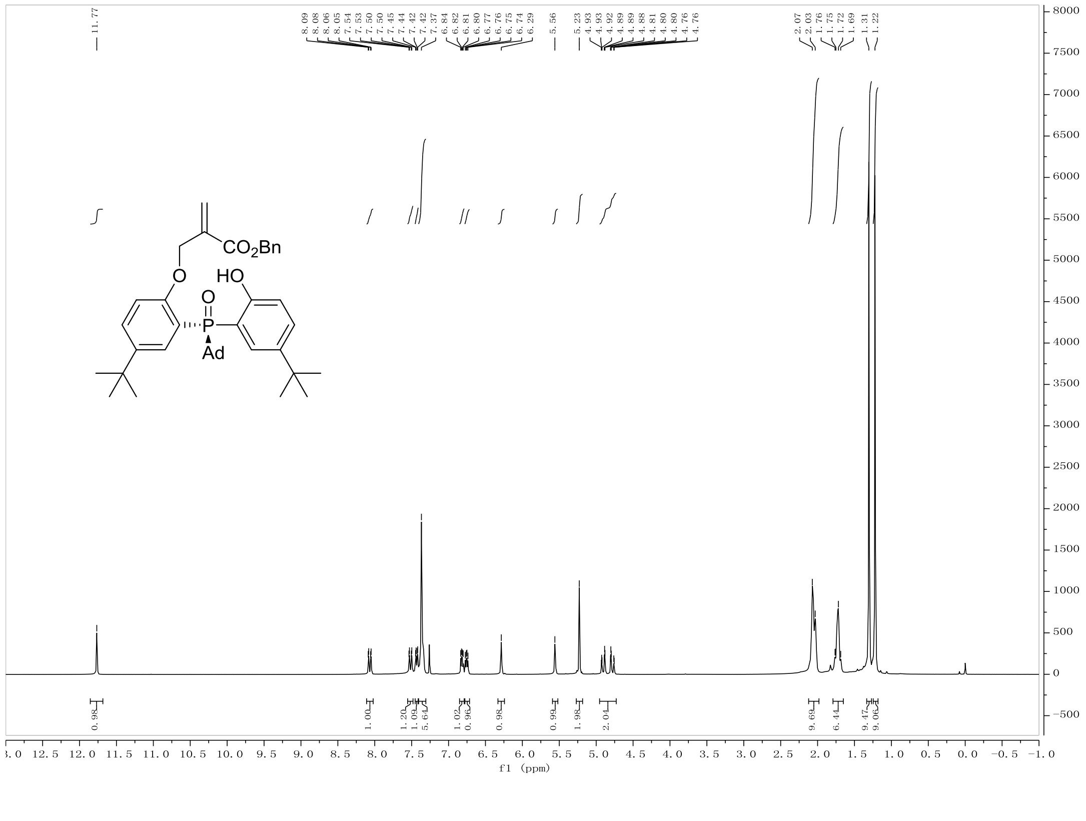

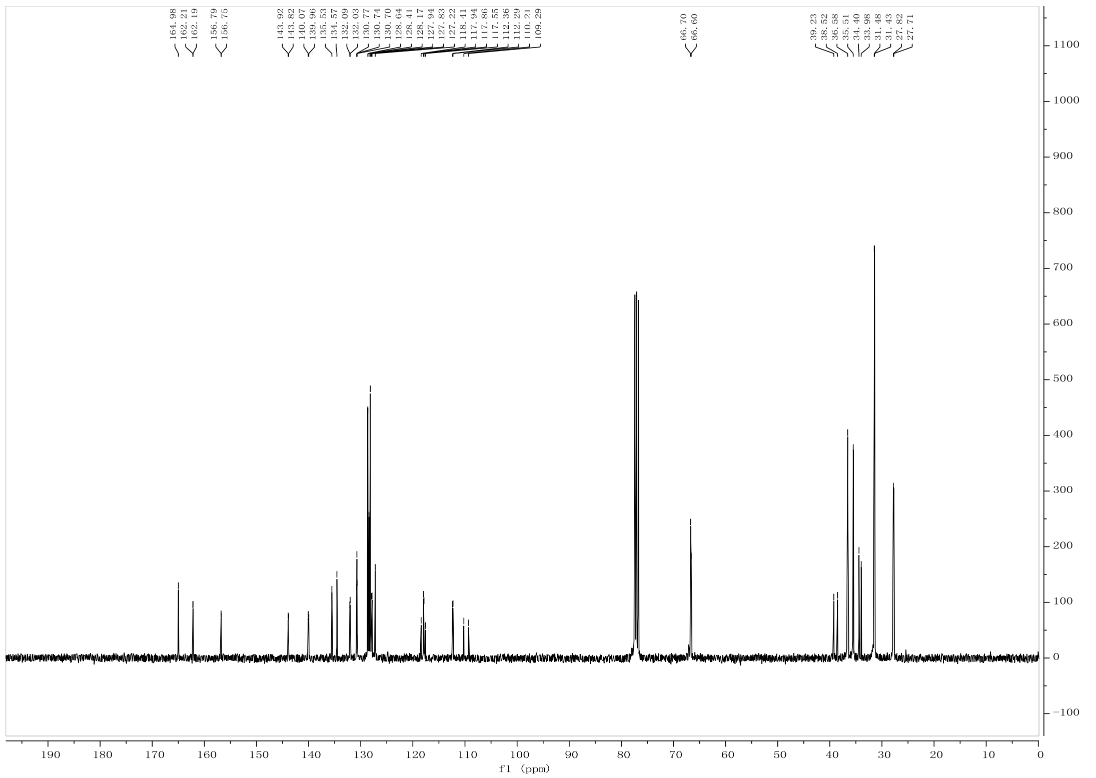

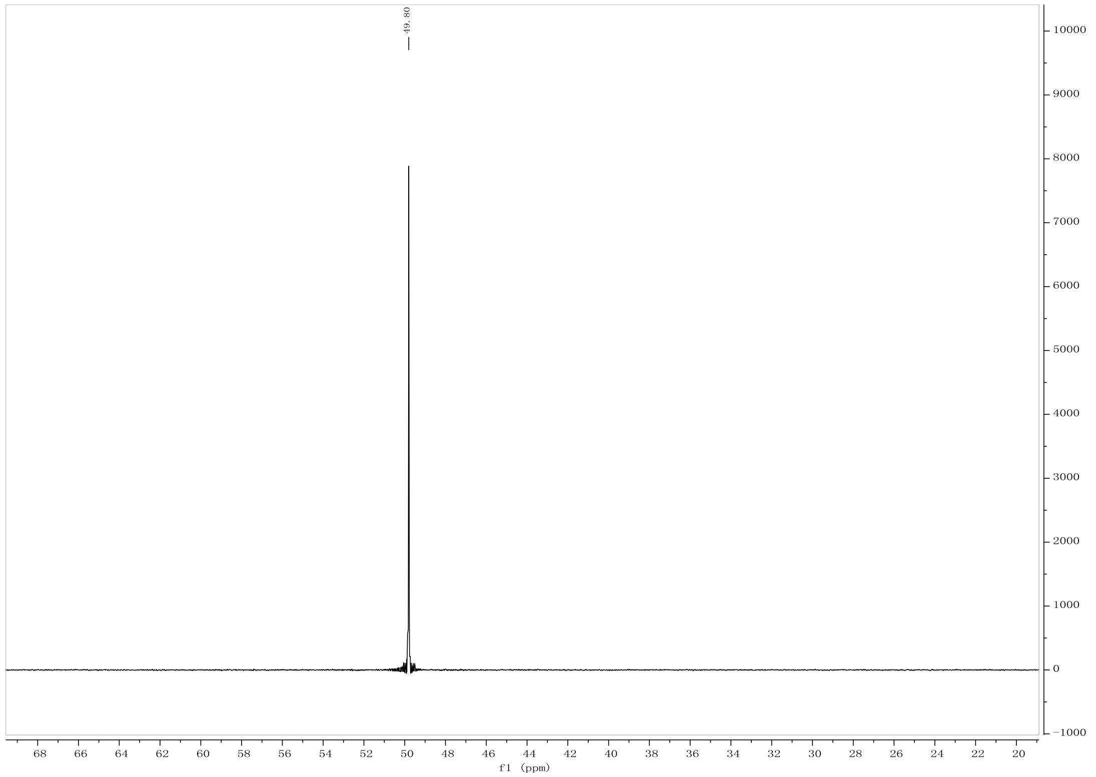

^{1}H NMR/^{13}C NMR/^{31}P NMR of product 3k

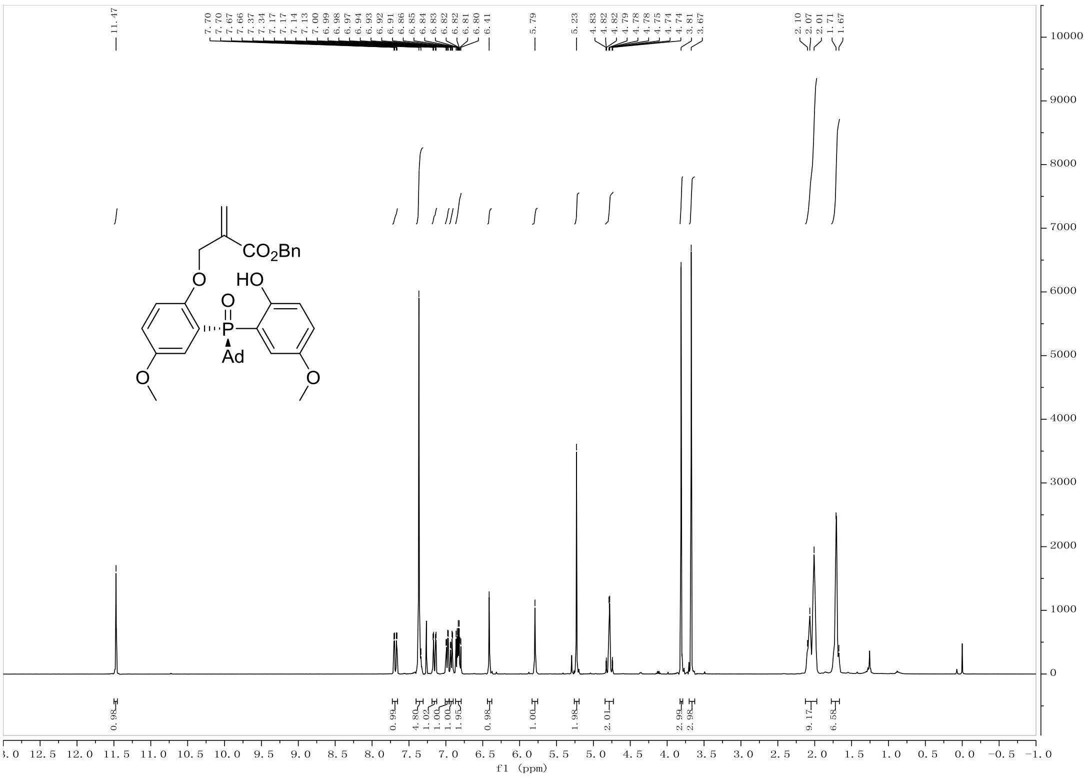
11.47
7.70 7.70 7.67 7.66 7.37 7.34 7.17 7.17 7.14 7.13 7.00 6.99 6.98 6.97 6.94 6.93 6.92 6.91 6.86 6.85 6.84 6.83 6.82 6.82 6.81 6.80
6.41
5.79
5.23
4.83 4.82 4.82 4.79 4.78 4.78 4.75 4.74 4.74
3.81 3.67
2.10 2.07 2.01 1.71 1.67
CO2Bn
O
HO
O
P
Ad
O
O
0.98
0.99 4.80 1.02 1.00 1.00 1.00 1.95
0.98
1.00
1.98
2.01
2.99 2.98
9.17 6.58
f1 (ppm)

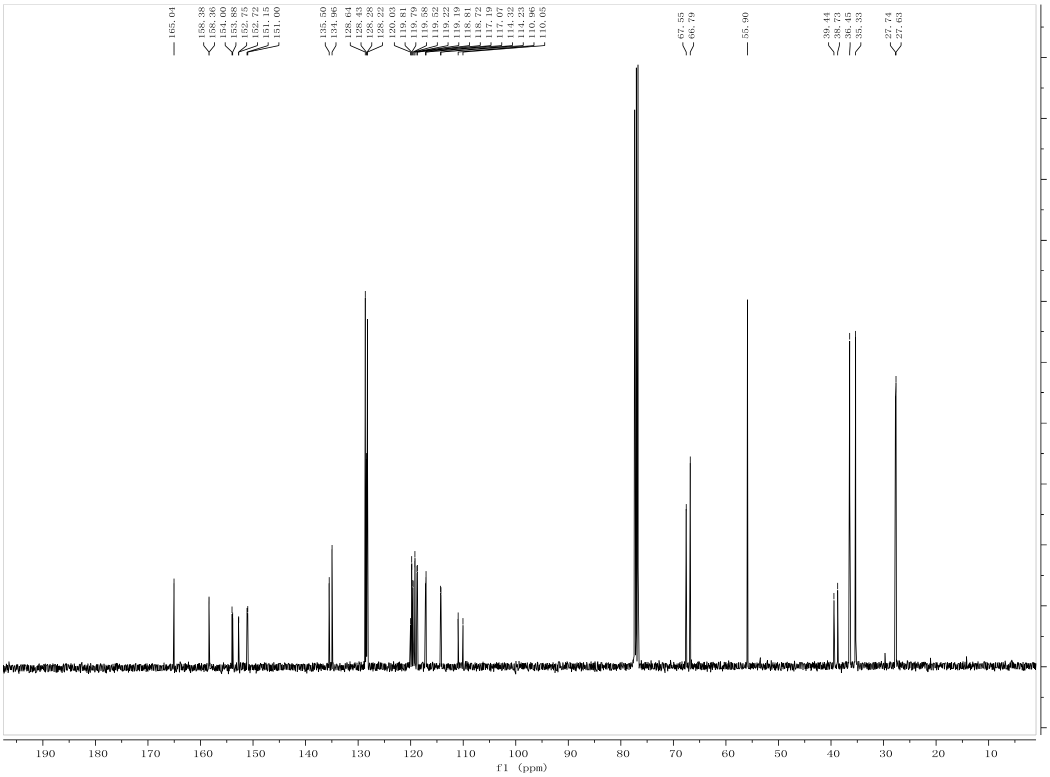
165.04
158.38 158.36 154.00 153.88 152.75 152.72 151.15 151.00
135.50 134.96 128.64 128.43 128.28 128.22 120.03 119.81 119.79 119.58 119.52 119.22 119.19 118.81 118.72 117.19 117.07 114.32 114.23 110.96 110.05
67.55 66.79
55.90
39.44 38.73 36.45 35.33
27.74 27.63
f1 (ppm)

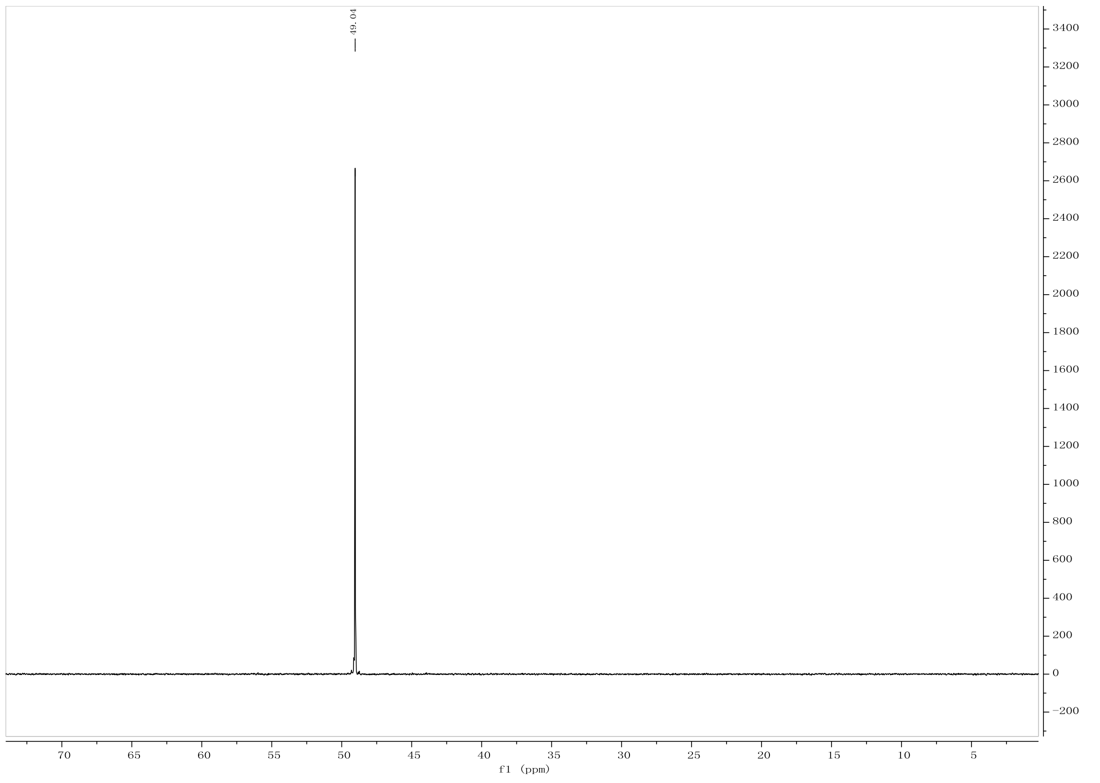

^{1}H NMR/^{13}C NMR/^{31}P NMR/^{19}F NMR of product 3l

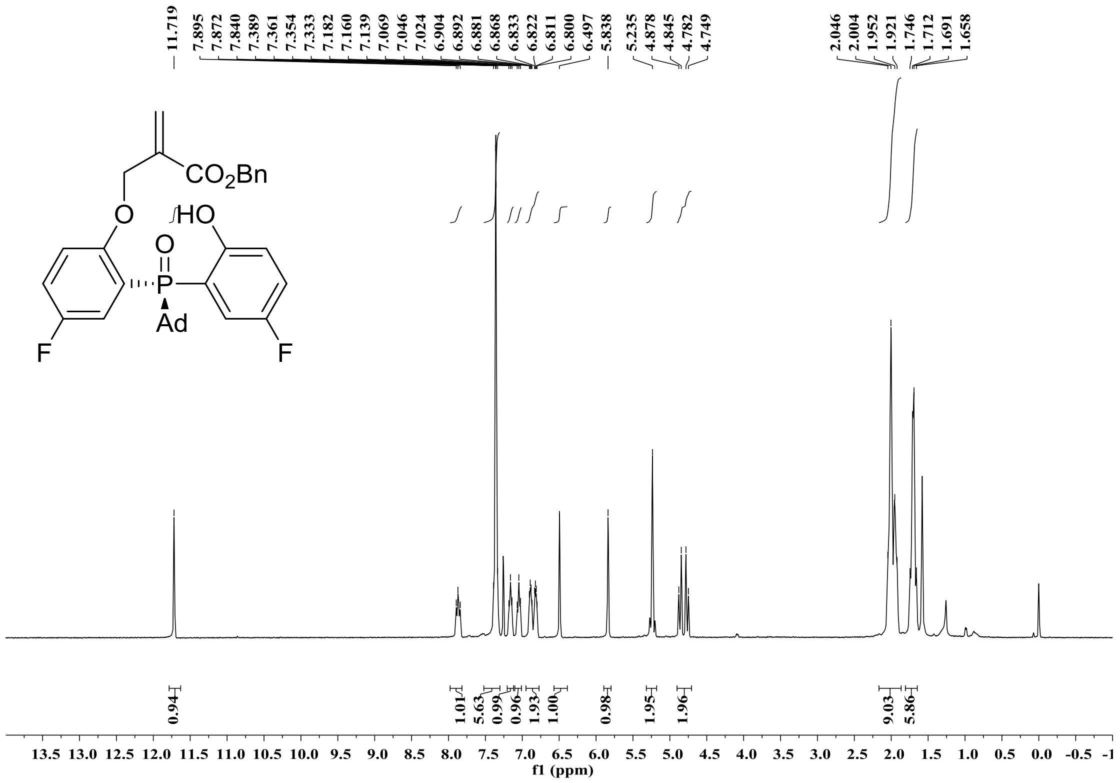

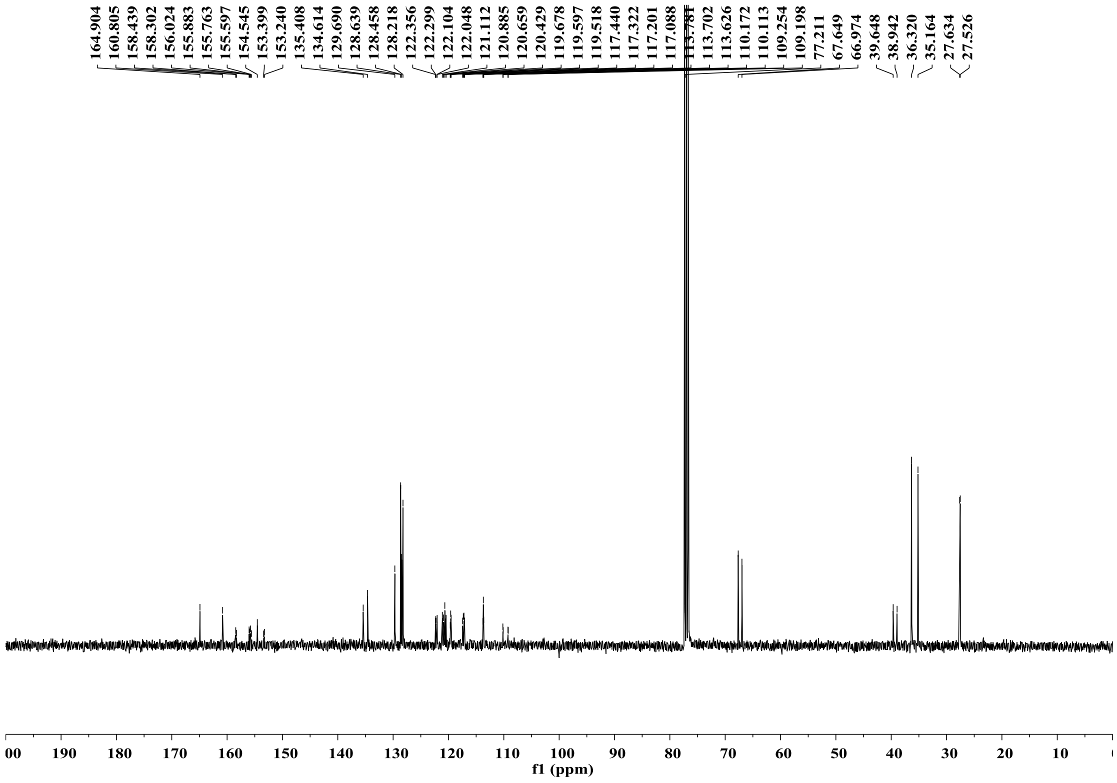
164.904
160.805
158.439
158.302
156.024
155.883
155.763
155.597
154.545
153.399
153.240
135.408
134.614
129.690
128.639
128.458
128.218
122.356
122.299
122.104
122.048
121.112
120.885
120.659
120.429
119.678
119.597
119.518
117.440
117.322
117.201
117.088
113.781
113.702
113.626
110.172
110.113
109.254
109.198
77.211
67.649
66.974
39.648
38.942
36.320
35.164
27.634
27.526
f1 (ppm)

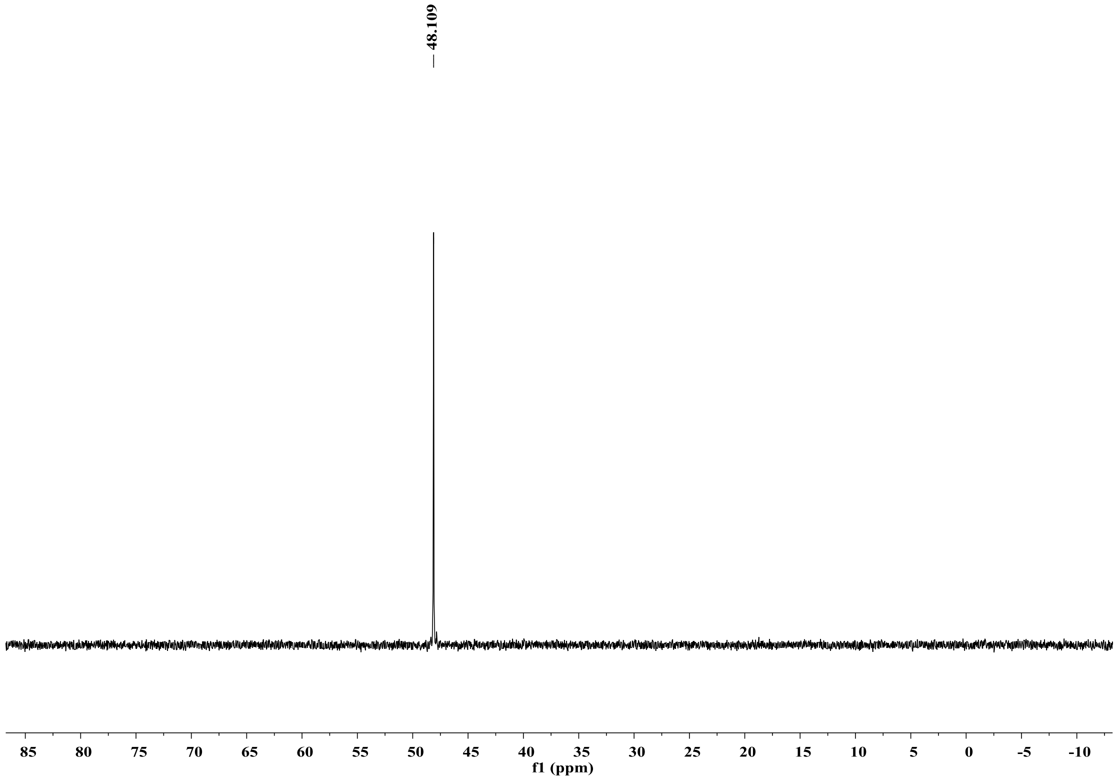
48.109
f1 (ppm)

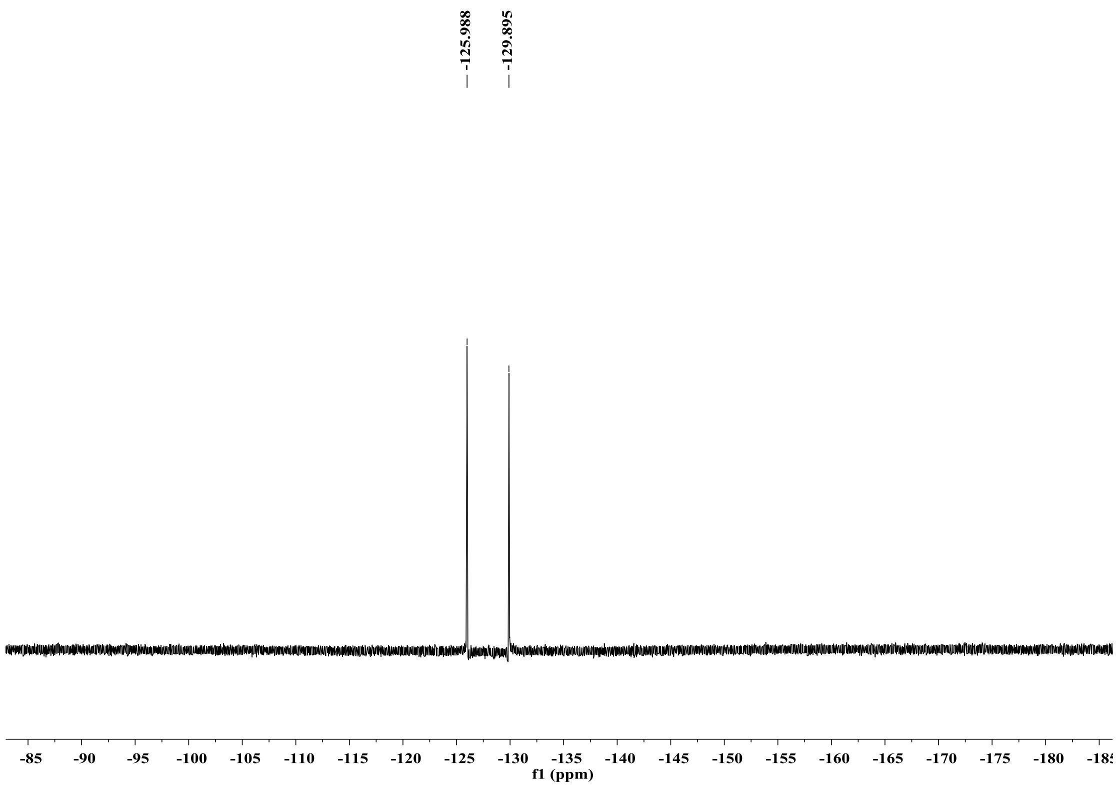

^{1}H NMR/^{13}C NMR/^{31}P NMR of product 3m

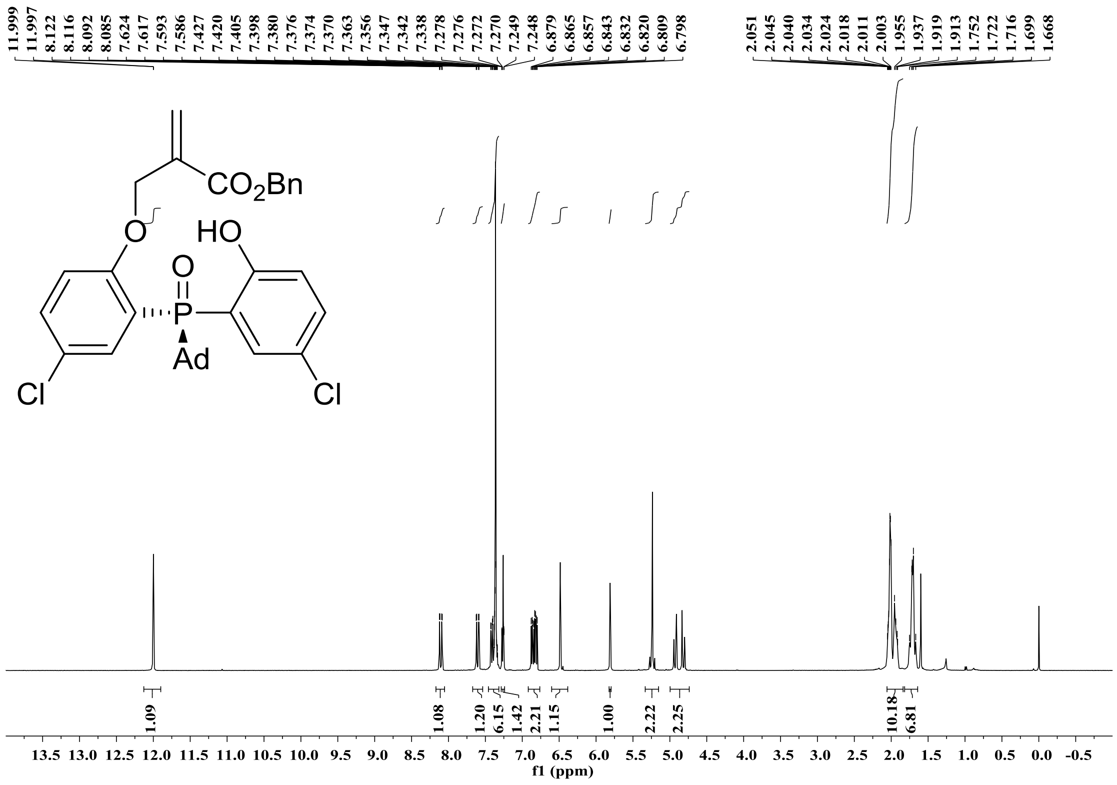

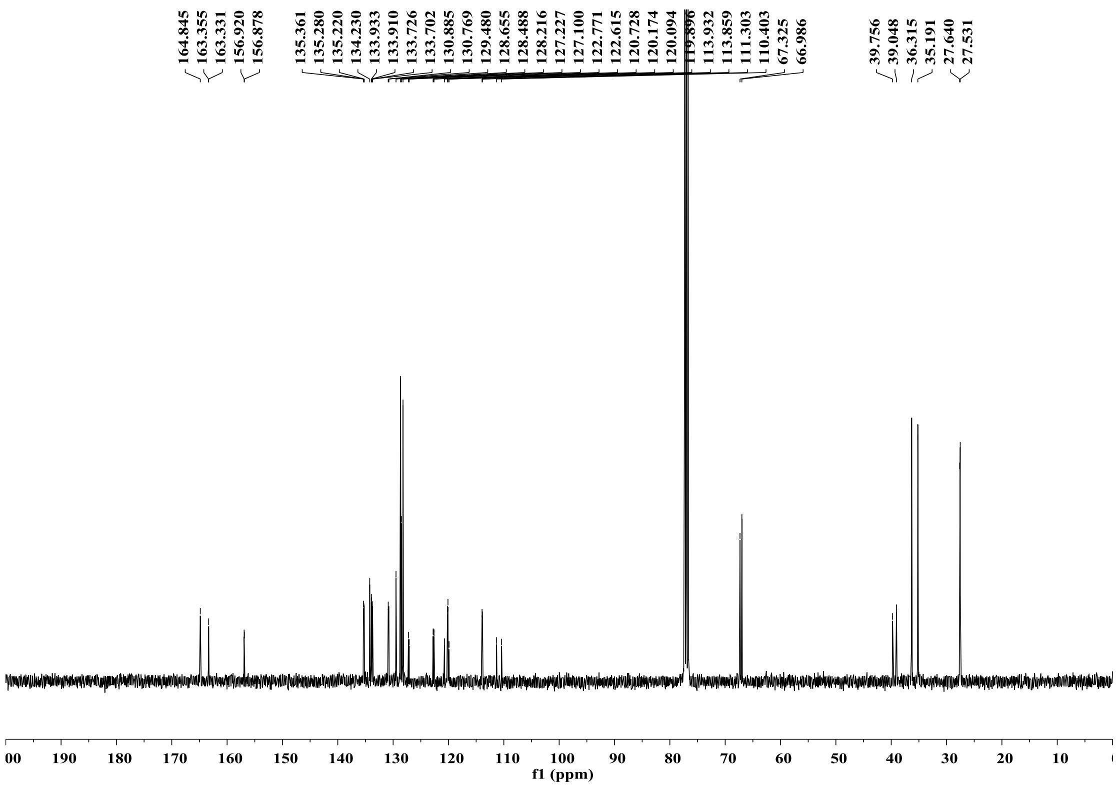

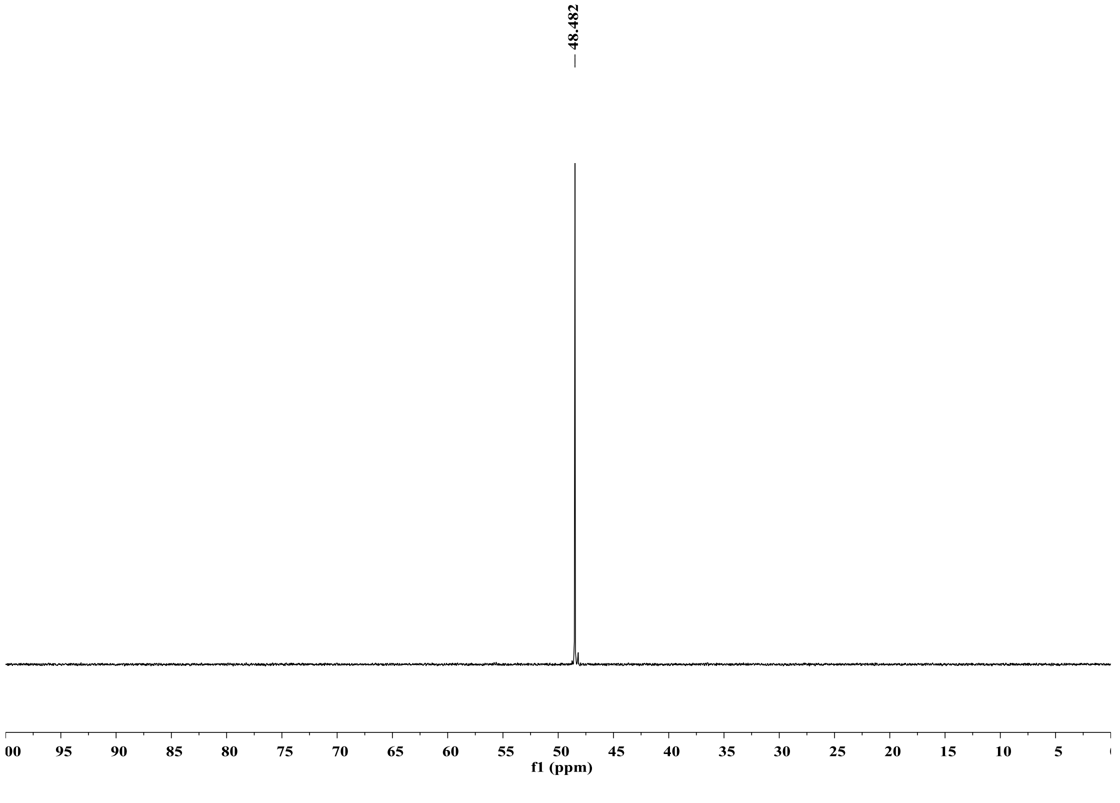

^{1}H NMR/^{13}C NMR/^{31}P NMR of product 3n

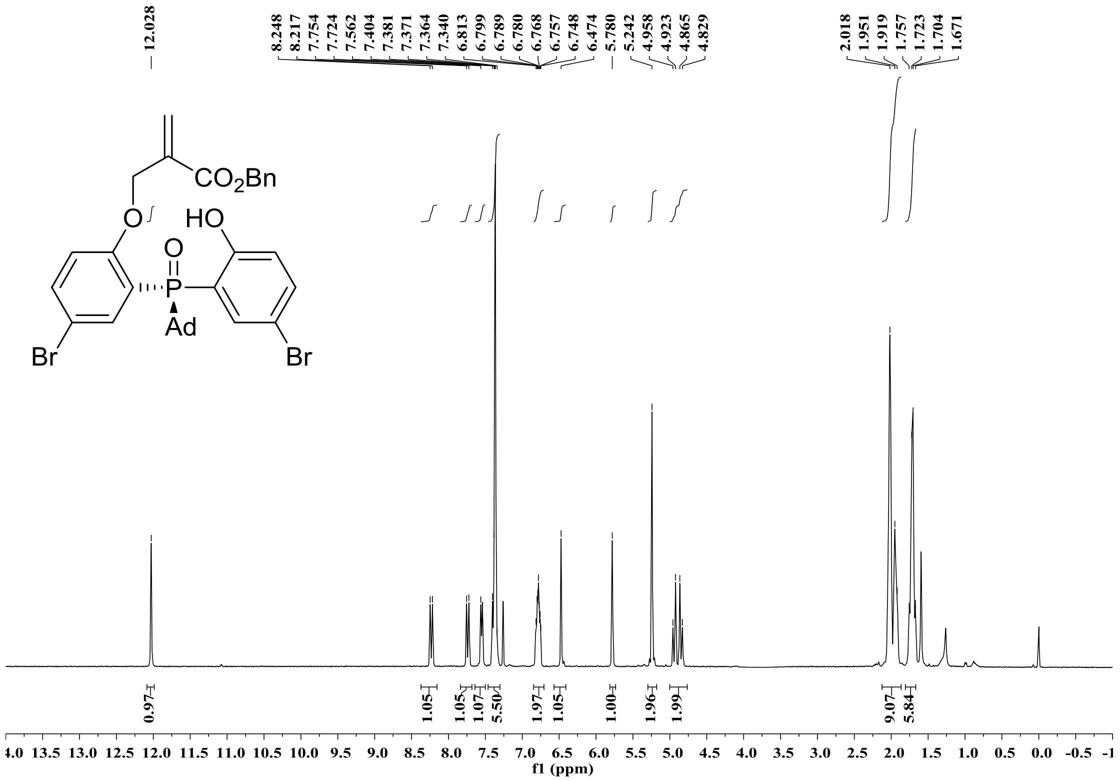
12.028
8.248
8.217
7.754
7.724
7.562
7.404
7.381
7.371
7.364
7.340
6.813
6.799
6.789
6.780
6.768
6.757
6.748
6.474
5.780
5.242
4.958
4.923
4.865
4.829
2.018
1.951
1.919
1.757
1.723
1.704
1.671
CO2Bn
O
HO
O
P
Ad
Br
Br
0.97
1.05
1.05
1.07
5.50
1.97
1.05
1.00
1.96
1.99
9.07
5.84
f1 (ppm)

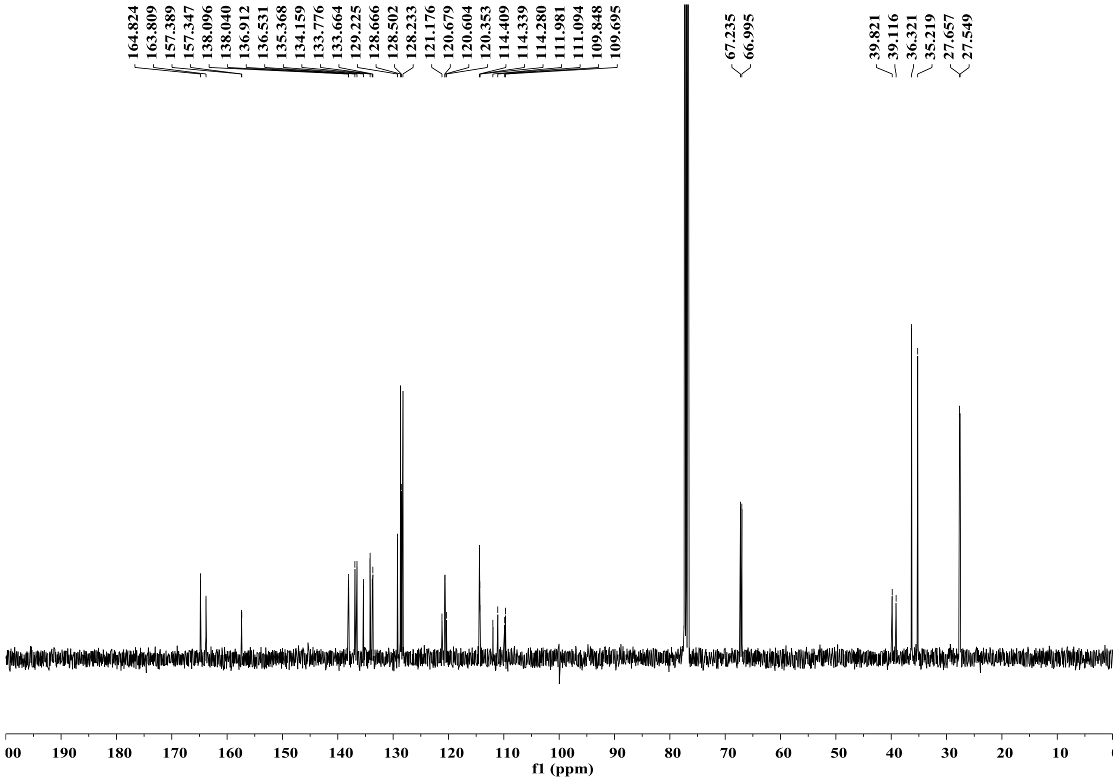
164.824
163.809
157.389
157.347
138.096
138.040
136.912
136.531
135.368
134.159
133.776
133.664
129.225
128.666
128.502
128.233
121.176
120.679
120.604
120.353
114.409
114.339
114.280
111.981
111.094
109.848
109.695
67.235
66.995
39.821
39.116
36.321
35.219
27.657
27.549
f1 (ppm)

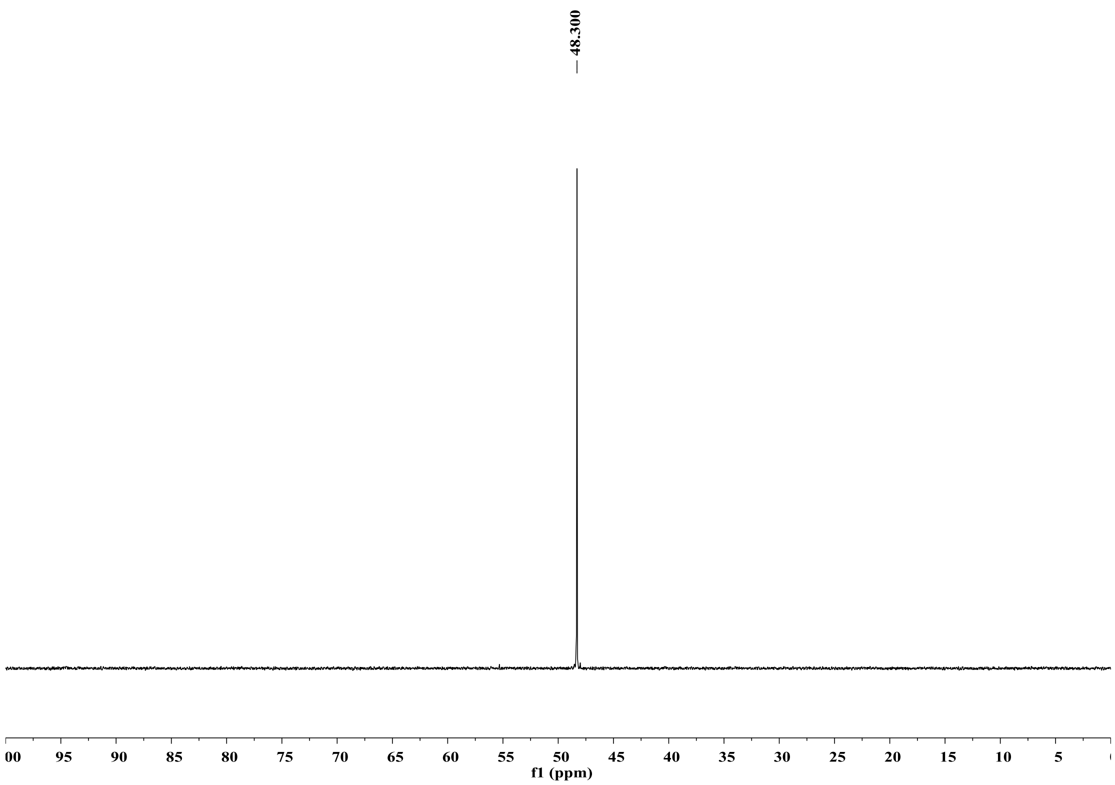

^{1}H NMR/^{13}C NMR/^{31}P NMR of product 3o

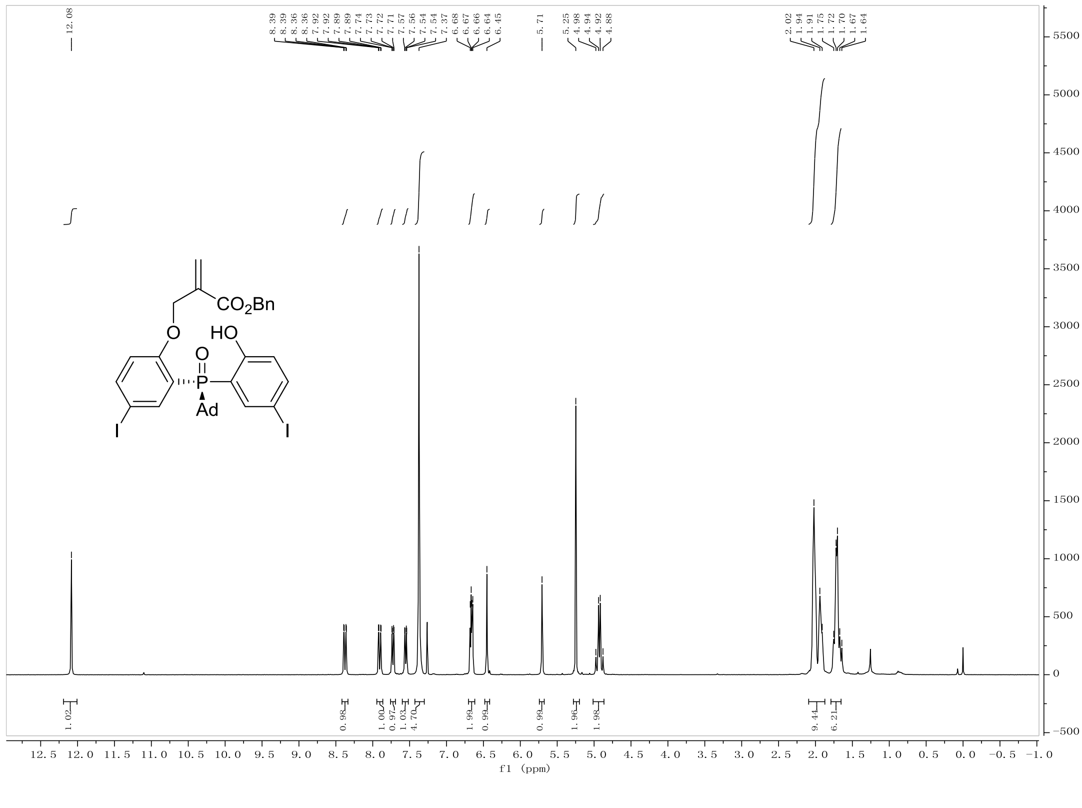

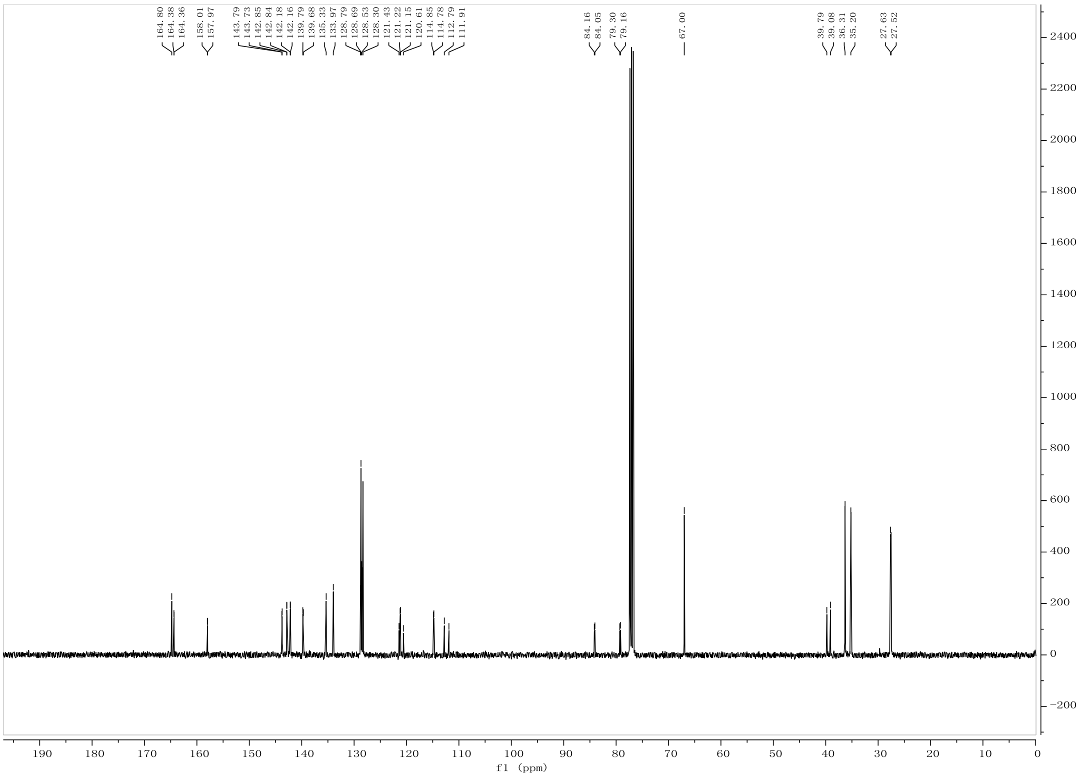

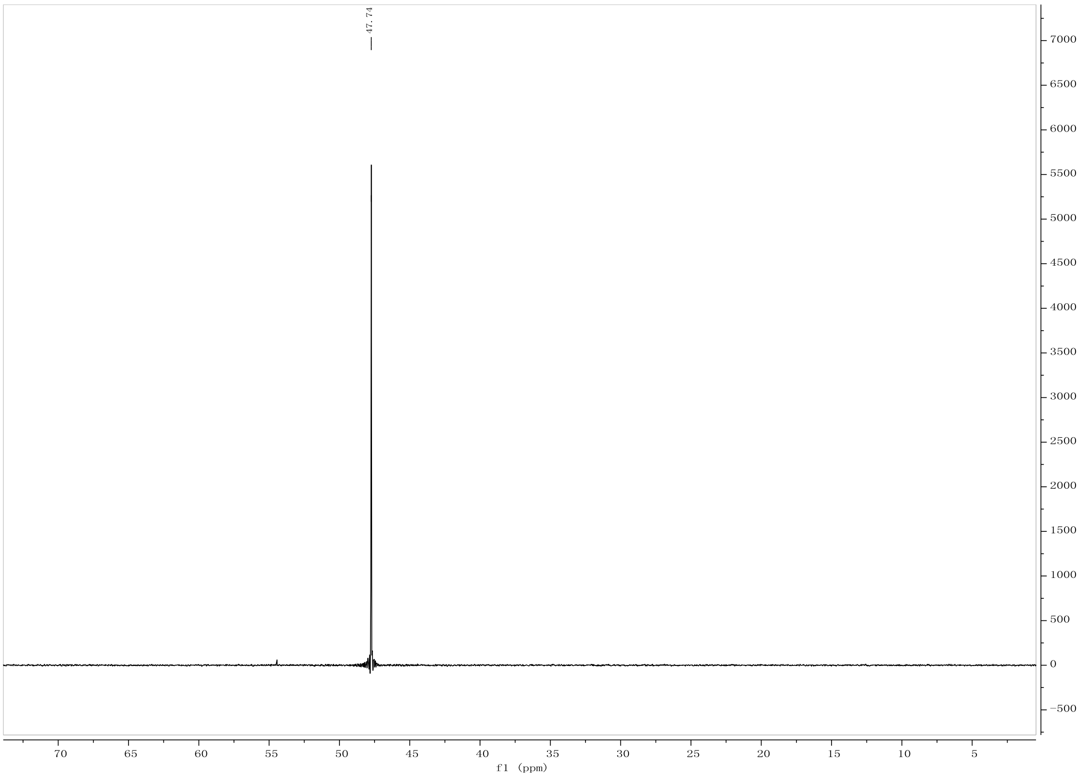

^{1}H NMR/^{13}C NMR/^{31}P NMR of product 3p

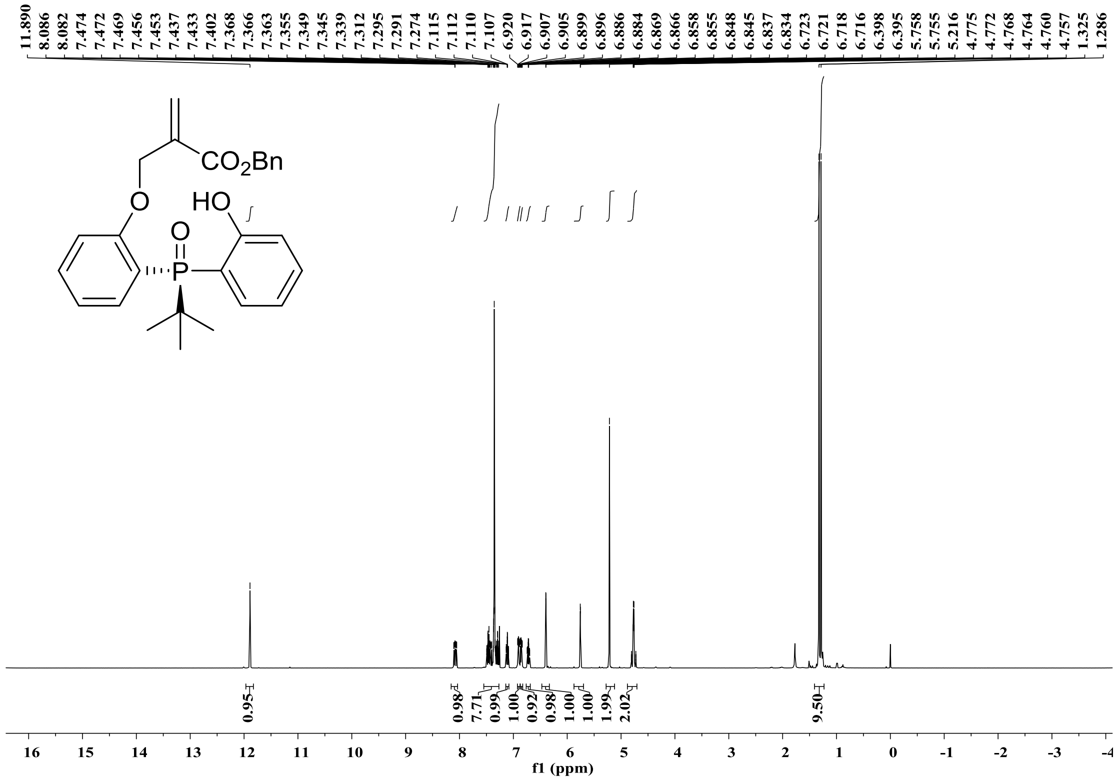

11.890
8.086
8.082
7.474
7.472
7.469
7.456
7.453
7.437
7.433
7.402
7.368
7.366
7.363
7.355
7.349
7.345
7.339
7.312
7.295
7.291
7.274
7.115
7.112
7.110
7.107
6.920
6.917
6.907
6.905
6.899
6.896
6.886
6.884
6.869
6.866
6.858
6.855
6.848
6.845
6.837
6.834
6.723
6.721
6.718
6.716
6.398
6.395
5.758
5.755
5.216
4.775
4.772
4.768
4.764
4.760
4.757
1.325
1.286
CO2Bn
O
HO
O
P
0.95
0.98
7.71
0.99
1.00
0.92
0.98
1.00
1.00
1.99
2.02
9.50
16 15 14 13 12 11 10 9 8 7 6 5 4 3 2 1 0 -1 -2 -3 -4
f1 (ppm)

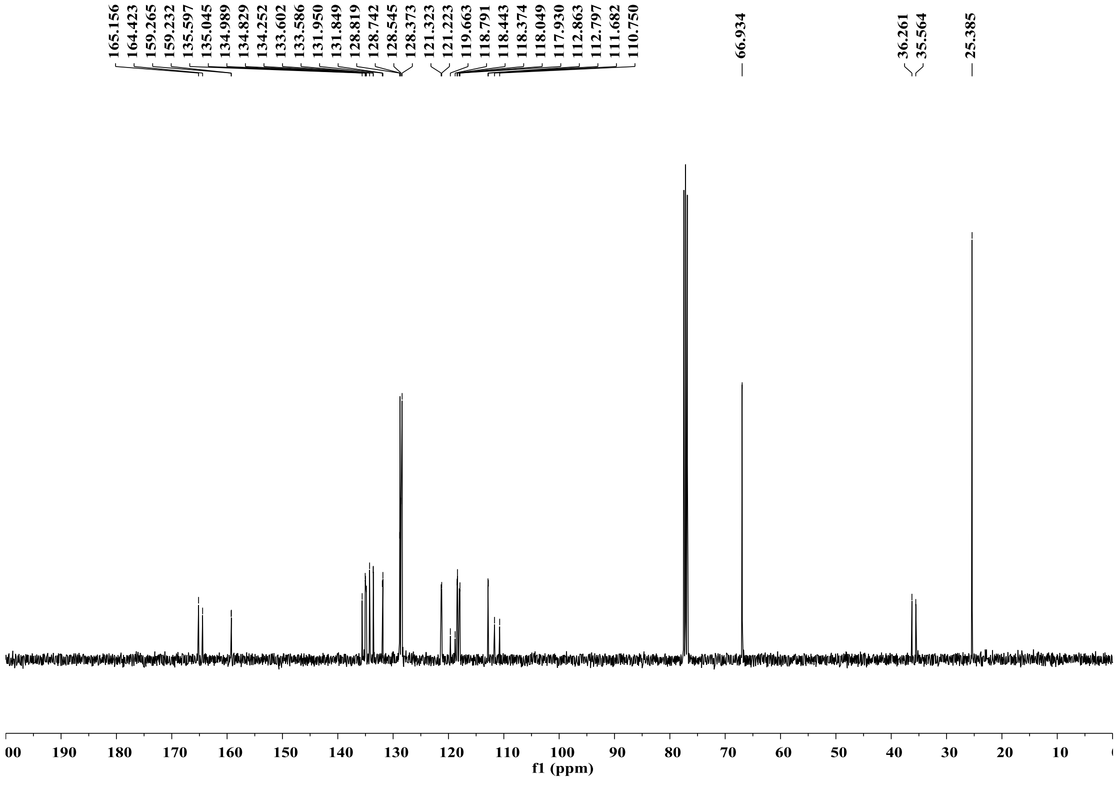

165.156
164.423
159.265
159.232
135.597
135.045
134.989
134.829
134.252
133.602
133.586
131.950
131.849
128.819
128.742
128.545
128.373
121.323
121.223
119.663
118.791
118.443
118.374
118.049
117.930
112.863
112.797
111.682
110.750
66.934
36.261
35.564
25.385
00 190 180 170 160 150 140 130 120 110 100 90 80 70 60 50 40 30 20 10
f1 (ppm)

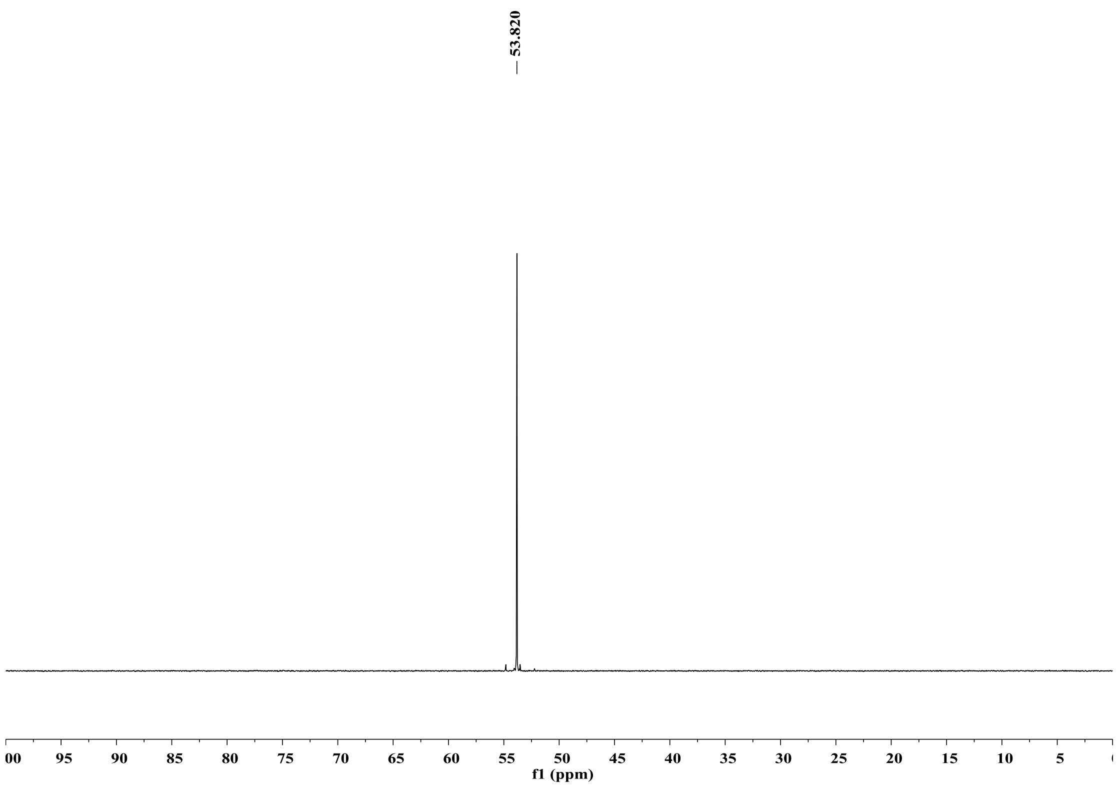

^{1}H NMR/^{13}C NMR/^{31}P NMR of product 3q

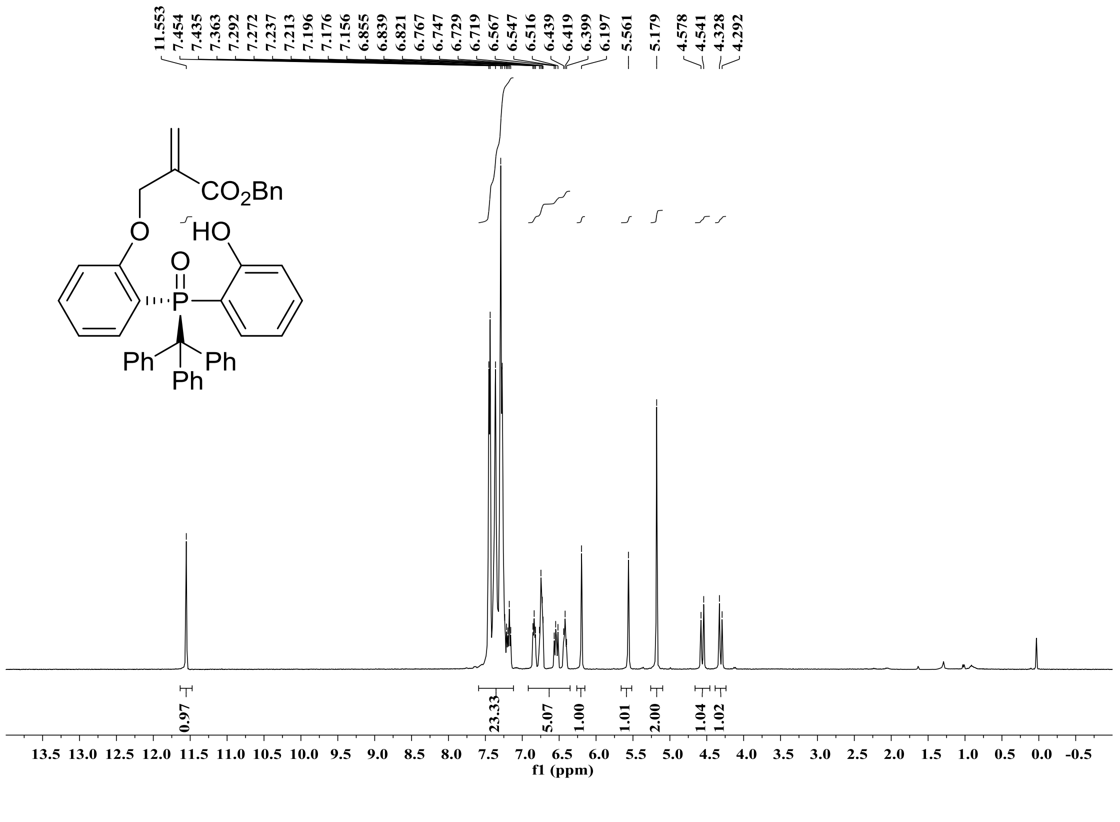

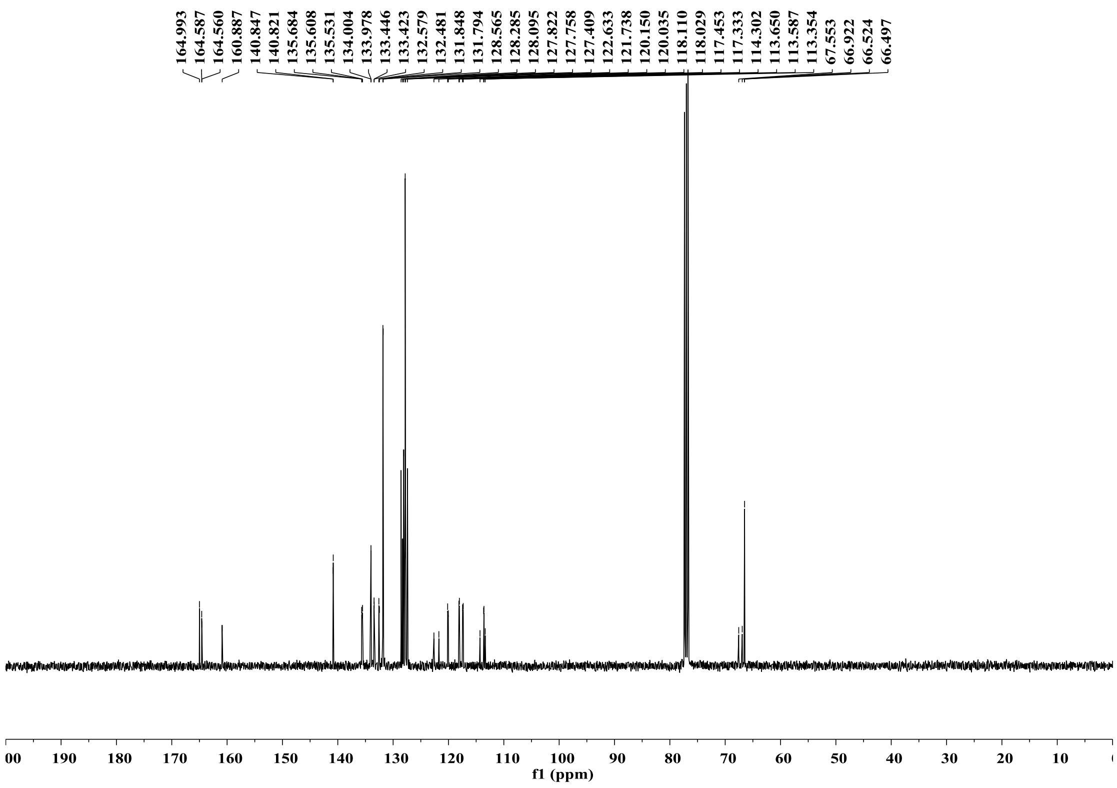

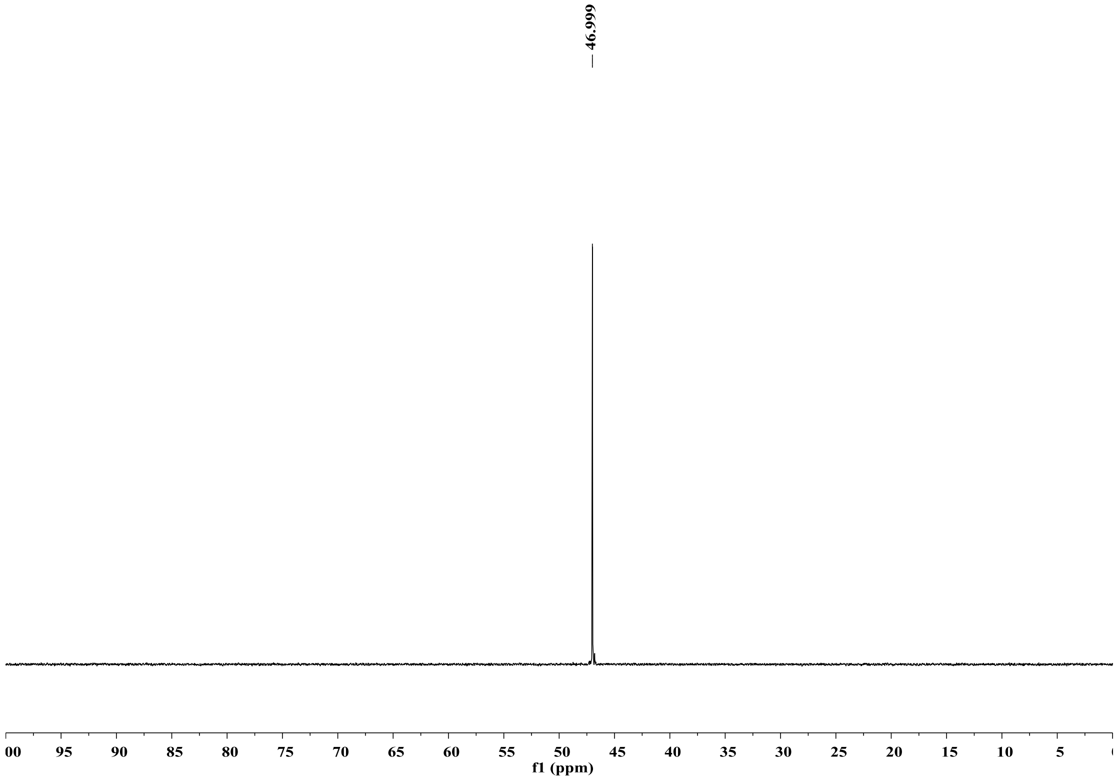

^{1}H NMR/^{13}C NMR/^{31}P NMR of product 3r

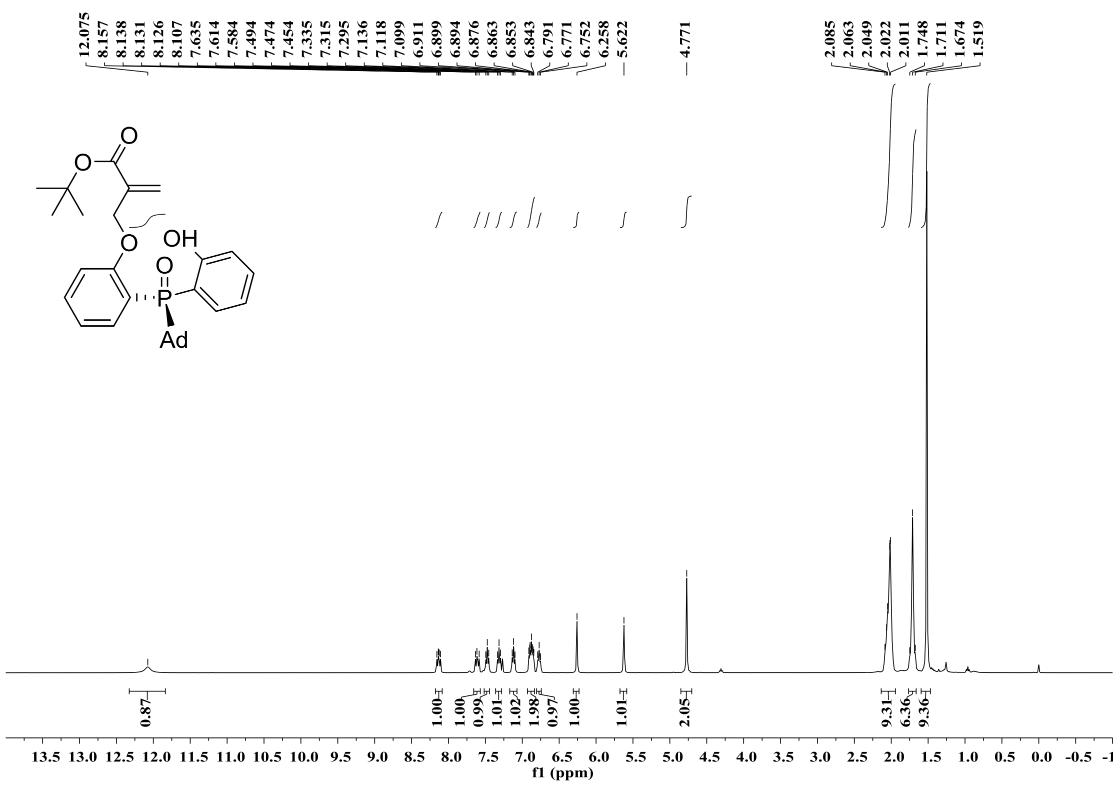
12.075
8.157
8.138
8.131
8.126
8.107
7.635
7.614
7.584
7.494
7.474
7.454
7.335
7.315
7.295
7.136
7.118
7.099
6.911
6.899
6.894
6.876
6.863
6.853
6.843
6.791
6.771
6.752
6.258
5.622
4.771
2.085
2.063
2.049
2.022
2.011
1.748
1.711
1.674
1.519
OH
O
P
Ad
0.87
1.00
1.00
0.99
1.01
1.02
1.98
0.97
1.00
1.01
2.05
9.31
6.36
9.36
f1 (ppm)

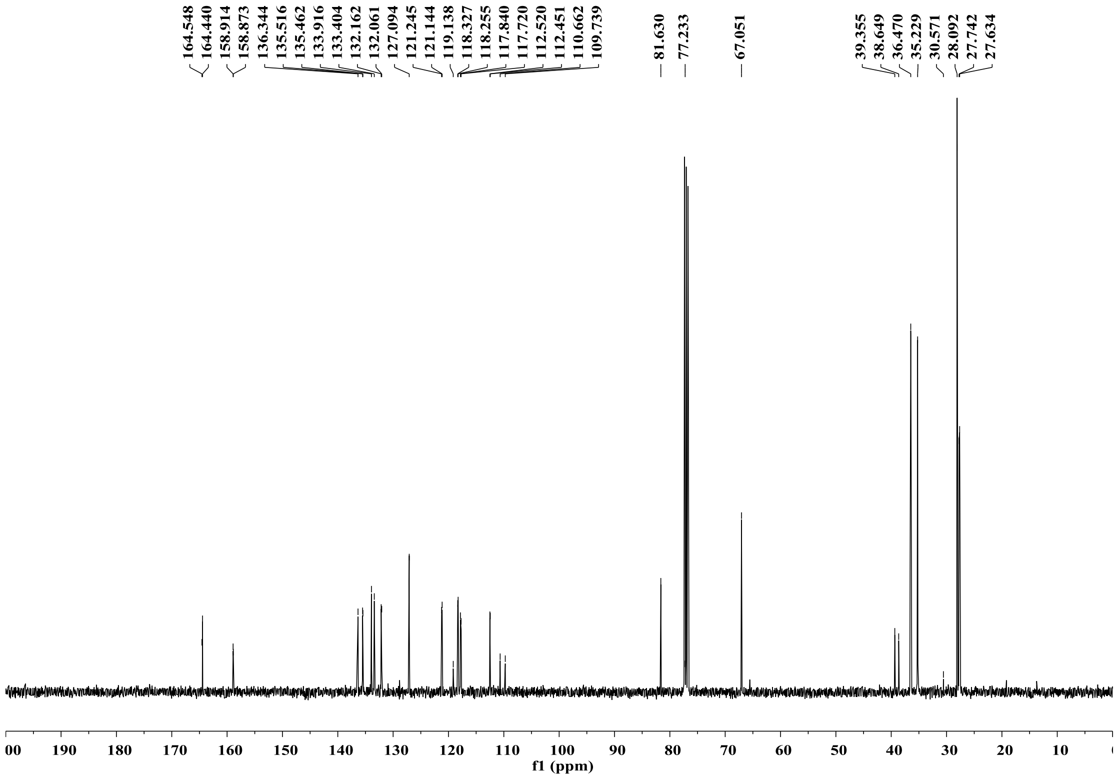
164.548
164.440
158.914
158.873
136.344
135.516
135.462
133.916
133.404
132.162
132.061
127.094
121.245
121.144
119.138
118.327
118.255
117.840
117.720
112.520
112.451
110.662
109.739
81.630
77.233
67.051
39.355
38.649
36.470
35.229
30.571
28.092
27.742
27.634
f1 (ppm)

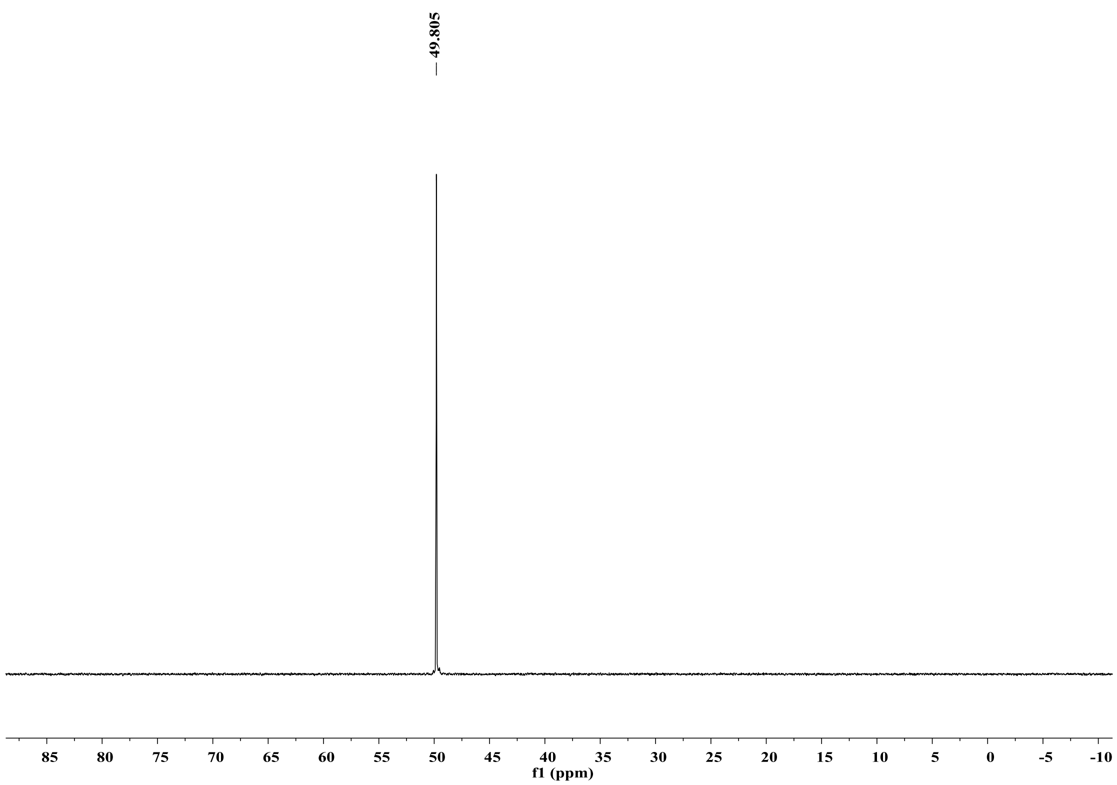

^{1}H NMR/^{13}C NMR/^{31}P NMR of product 3s

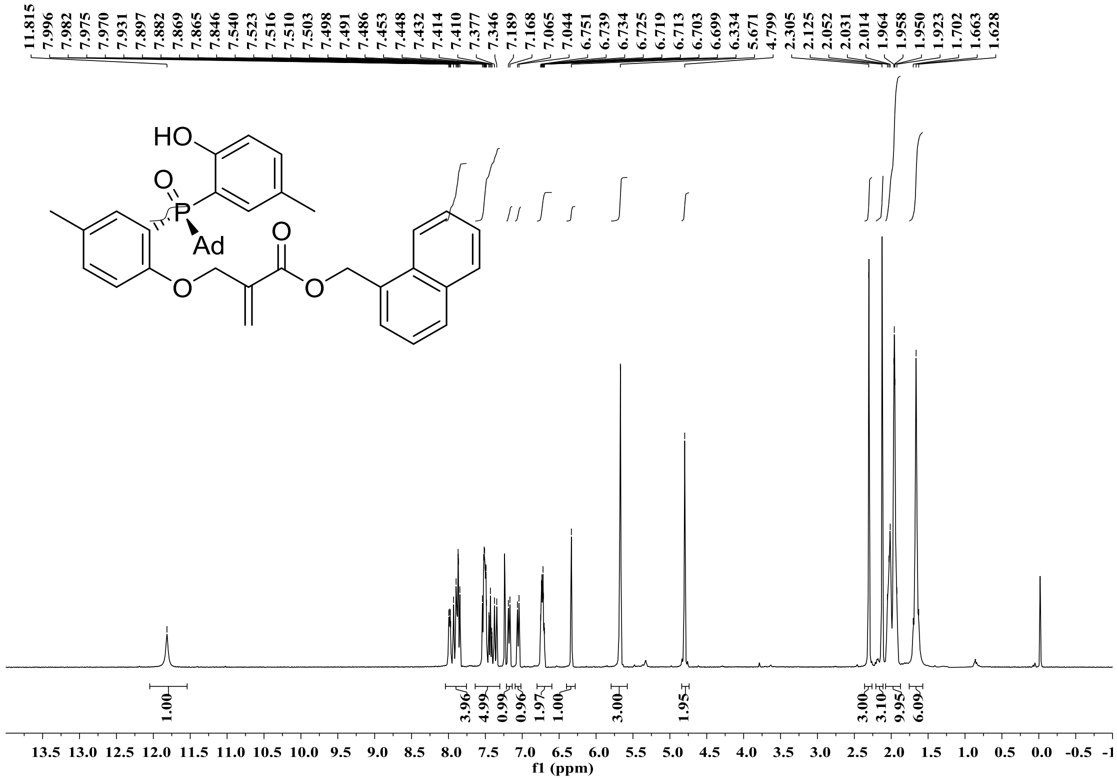

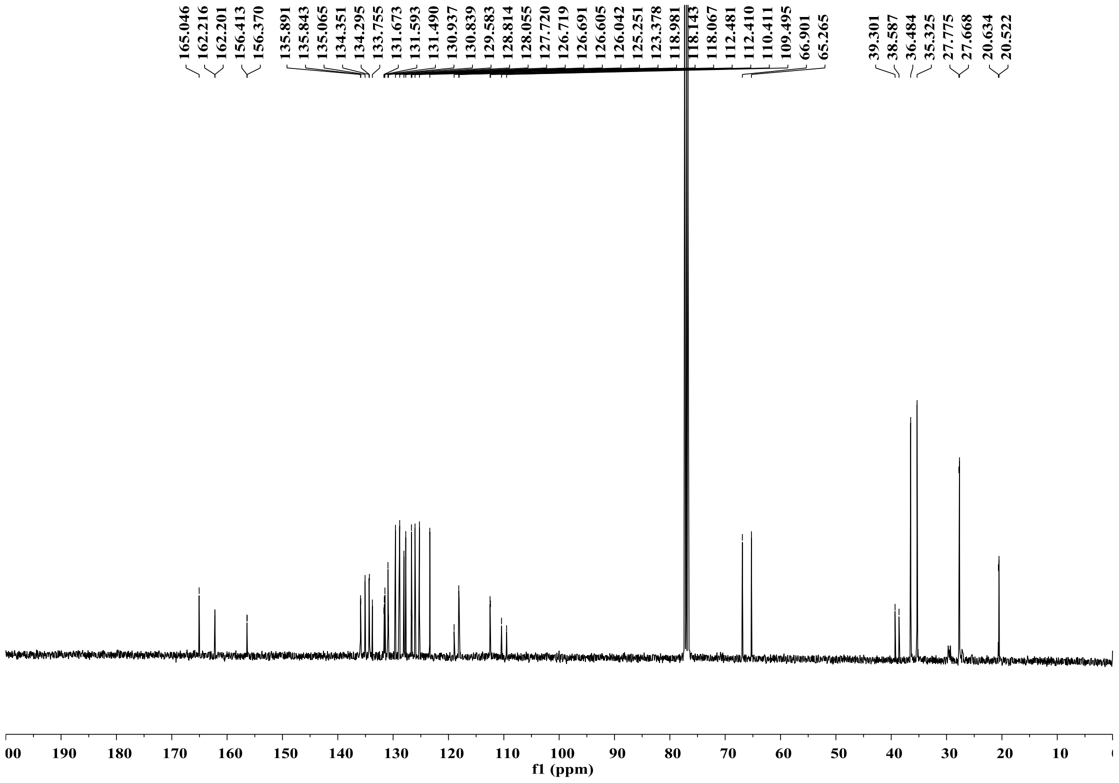

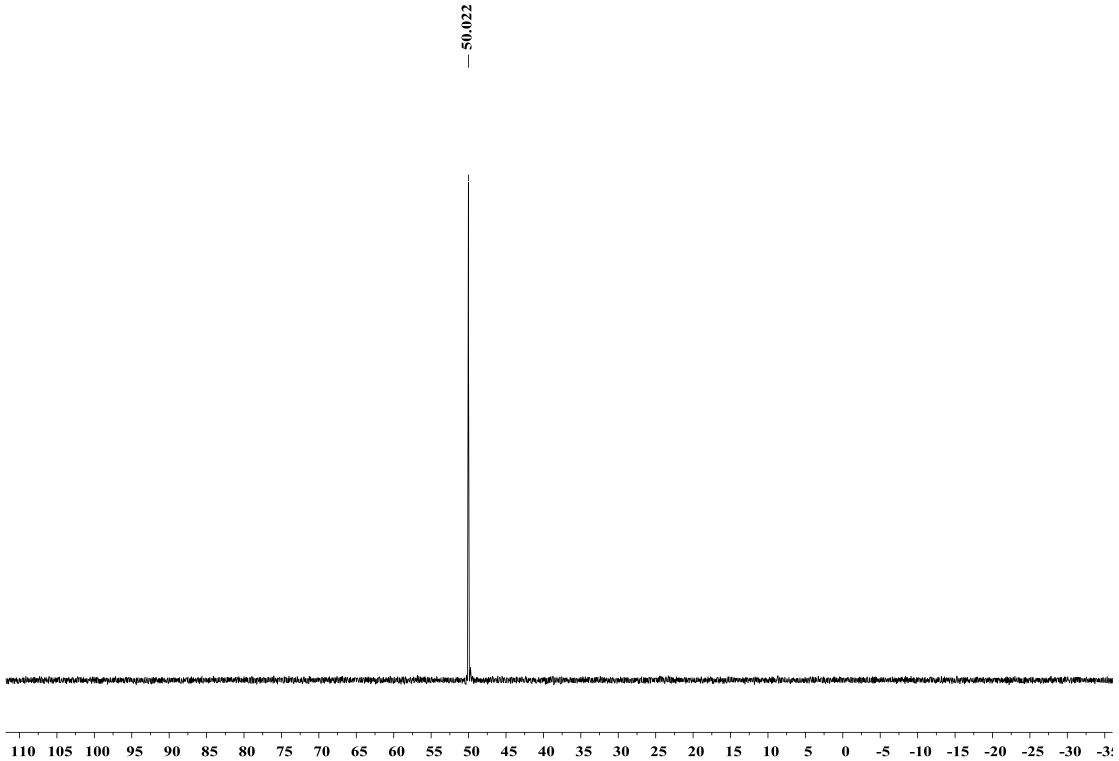

^{1}H NMR/^{13}C NMR/^{31}P NMR/^{19}F NMR of product 3t

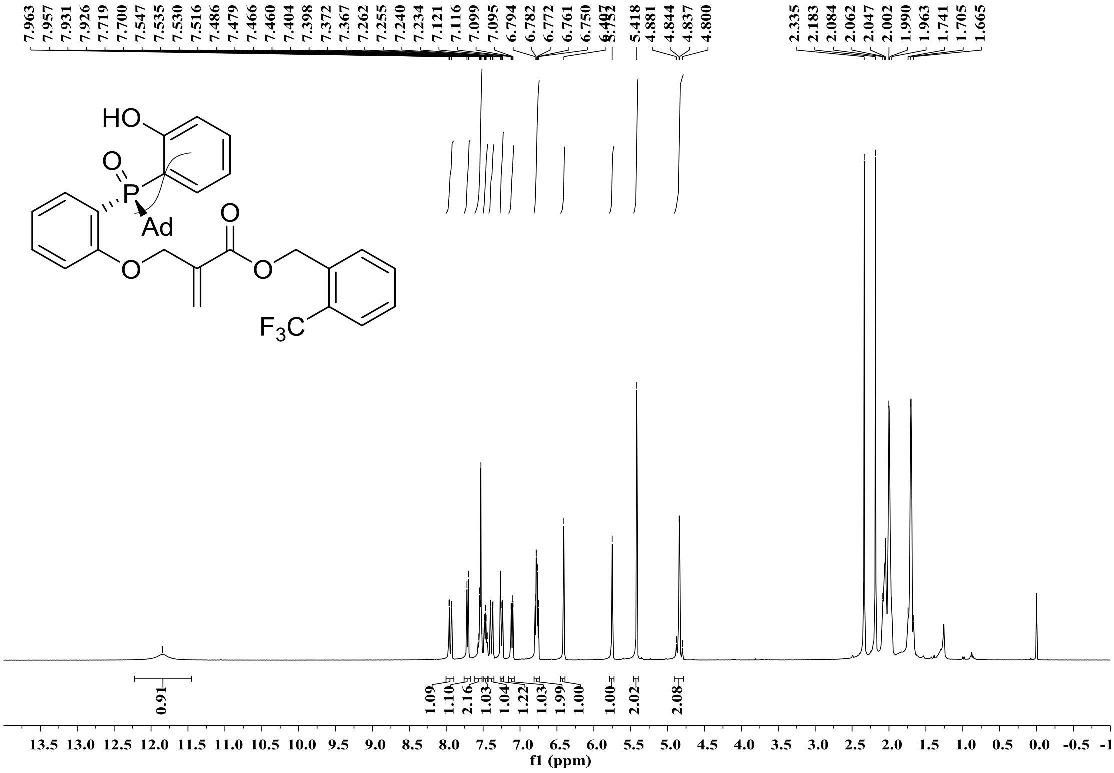
7.963 7.957 7.931 7.926 7.719 7.700 7.547 7.535 7.530 7.516 7.486 7.479 7.466 7.460 7.404 7.398 7.372 7.367 7.262 7.255 7.240 7.234 7.121 7.116 7.099 7.095 6.794 6.782 6.772 6.761 6.750 5.752 5.418 4.881 4.844 4.837 4.800 2.335 2.183 2.084 2.062 2.047 2.002 1.990 1.963 1.741 1.705 1.665
HO
O
P
Ad
O
O
O
F3C
0.91
1.09 1.10 2.16 1.03 1.04 1.22 1.03 1.99 1.00 1.00 2.02 2.08
13.5 13.0 12.5 12.0 11.5 11.0 10.5 10.0 9.5 9.0 8.5 8.0 7.5 7.0 6.5 6.0 5.5 5.0 4.5 4.0 3.5 3.0 2.5 2.0 1.5 1.0 0.5 0.0 -0.5 -1
f1 (ppm)

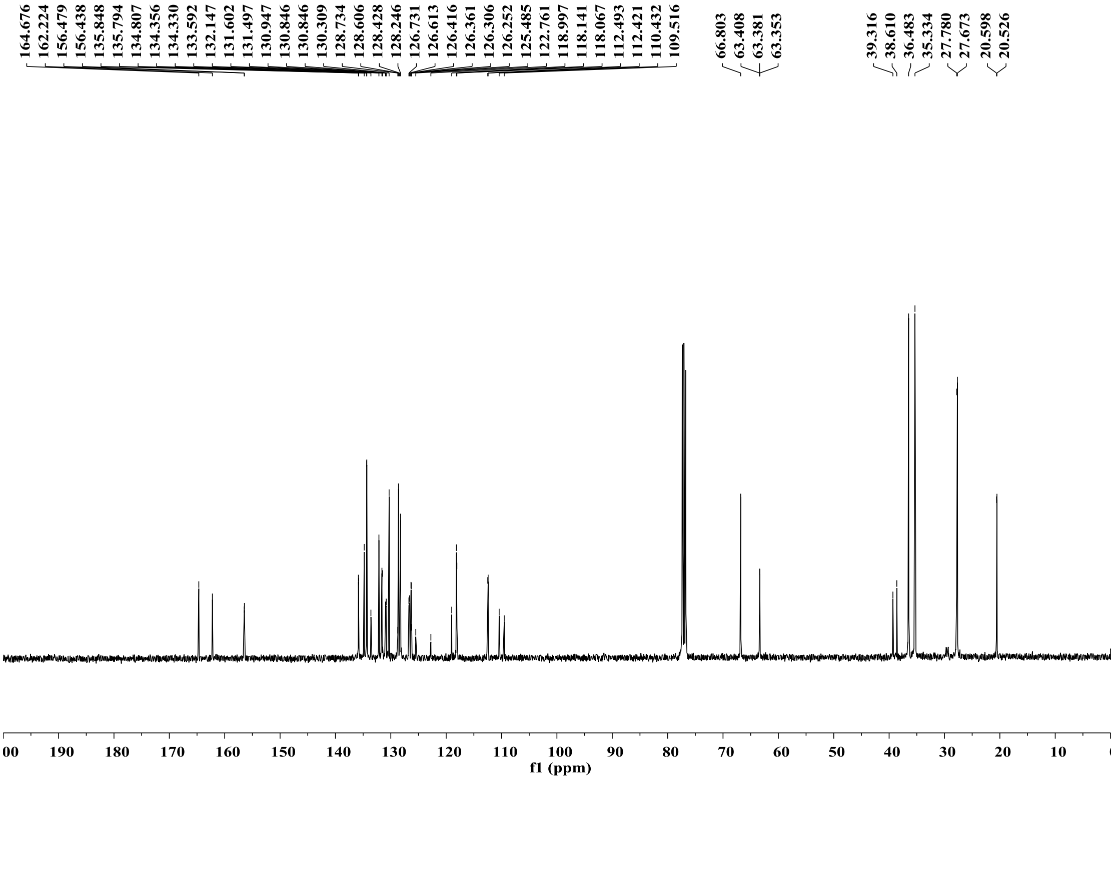
164.676 162.224 156.479 156.438 135.848 135.794 134.807 134.356 134.330 133.592 132.147 131.602 131.497 130.947 130.846 130.846 130.309 128.734 128.606 128.428 128.246 126.731 126.613 126.416 126.361 126.306 126.252 125.485 122.761 118.997 118.141 118.067 112.493 112.421 110.432 109.516
66.803 63.408 63.381 63.353
39.316 38.610 36.483 35.334 27.780 27.673 20.598 20.526
00 190 180 170 160 150 140 130 120 110 100 90 80 70 60 50 40 30 20 10
f1 (ppm)

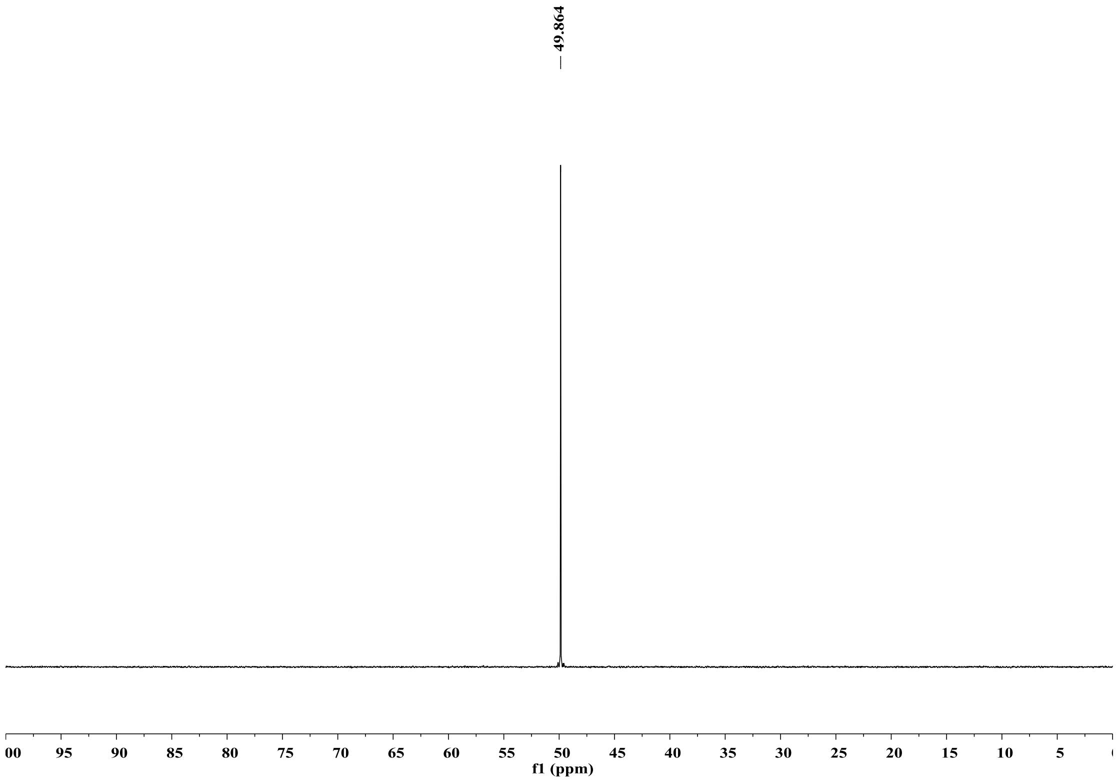

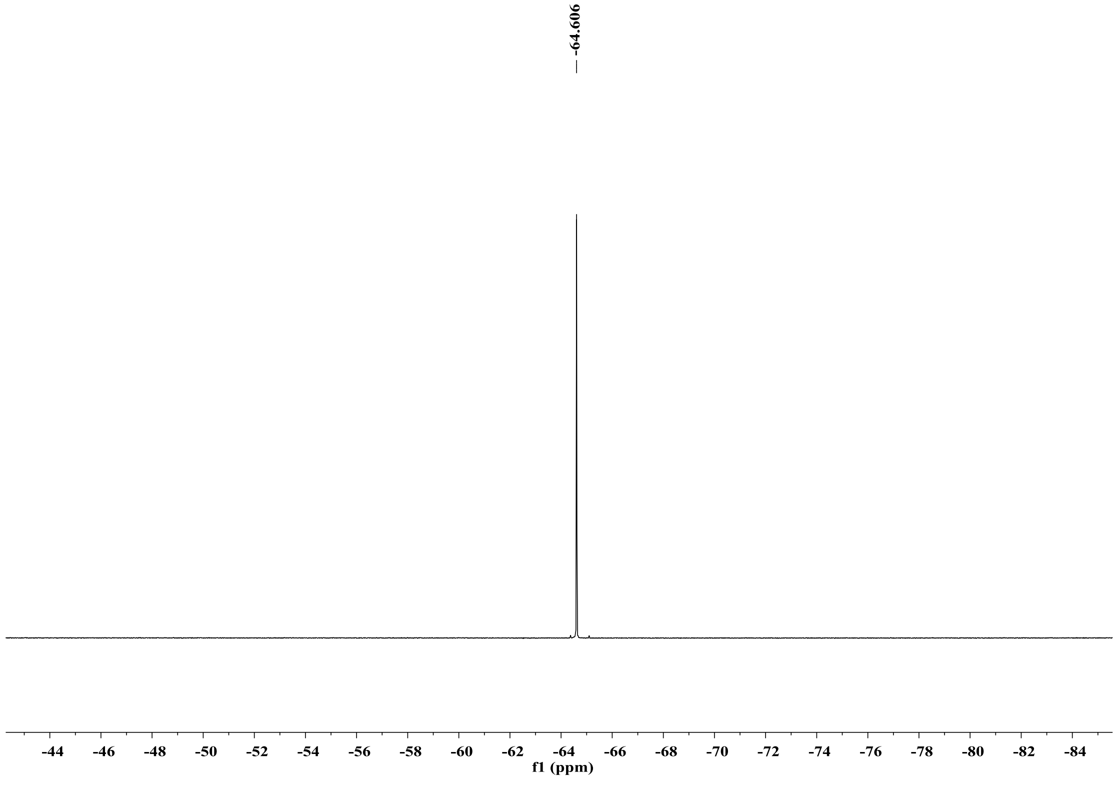

^{1}H NMR/^{13}C NMR/^{31}P NMR of product 3u

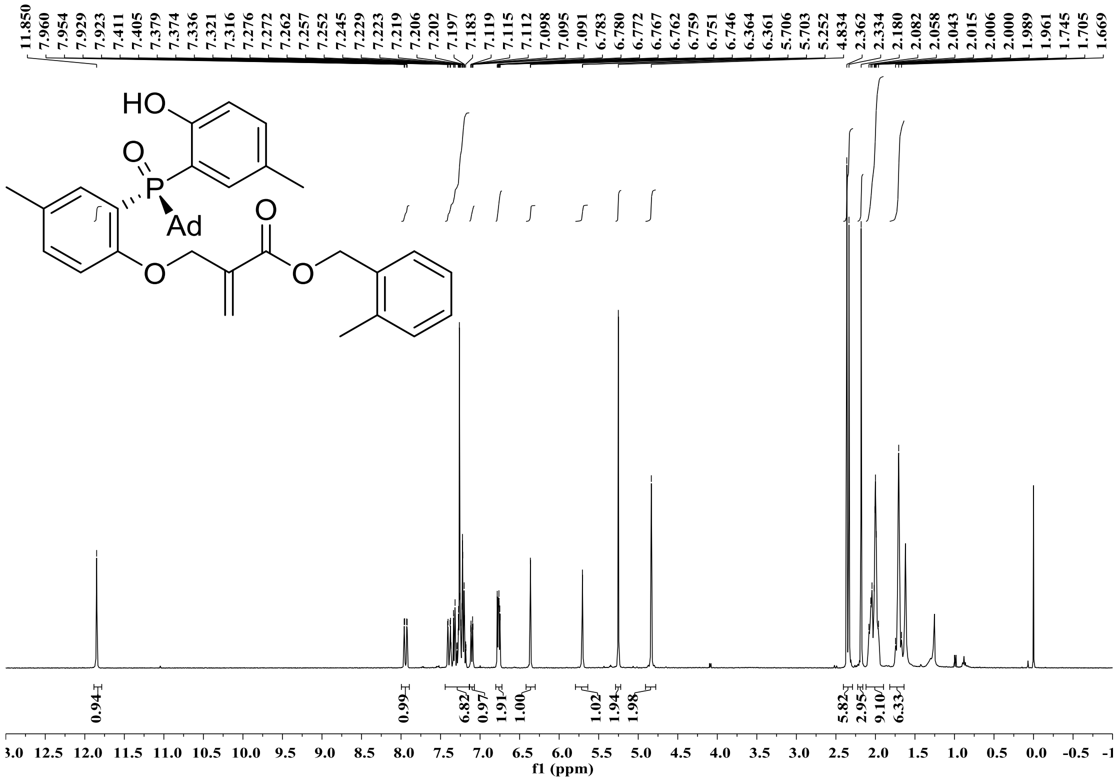
11.850 7.960 7.954 7.929 7.923 7.411 7.405 7.379 7.374 7.336 7.321 7.316 7.276 7.272 7.262 7.257 7.252 7.245 7.229 7.223 7.219 7.206 7.202 7.197 7.183 7.119 7.115 7.112 7.098 7.095 7.091 6.783 6.780 6.772 6.767 6.762 6.759 6.751 6.746 6.364 6.361 5.706 5.703 5.252 4.834 2.362 2.334 2.180 2.082 2.058 2.043 2.015 2.006 2.000 1.989 1.961 1.745 1.705 1.669
HO
O
P
Ad
O
O
O
0.94
0.99
6.82
0.97
1.91
1.00
1.02
1.94
1.98
5.82
2.95
9.10
6.33
12.5 12.0 11.5 11.0 10.5 10.0 9.5 9.0 8.5 8.0 7.5 7.0 6.5 6.0 5.5 5.0 4.5 4.0 3.5 3.0 2.5 2.0 1.5 1.0 0.5 0.0 -0.5
f1 (ppm)

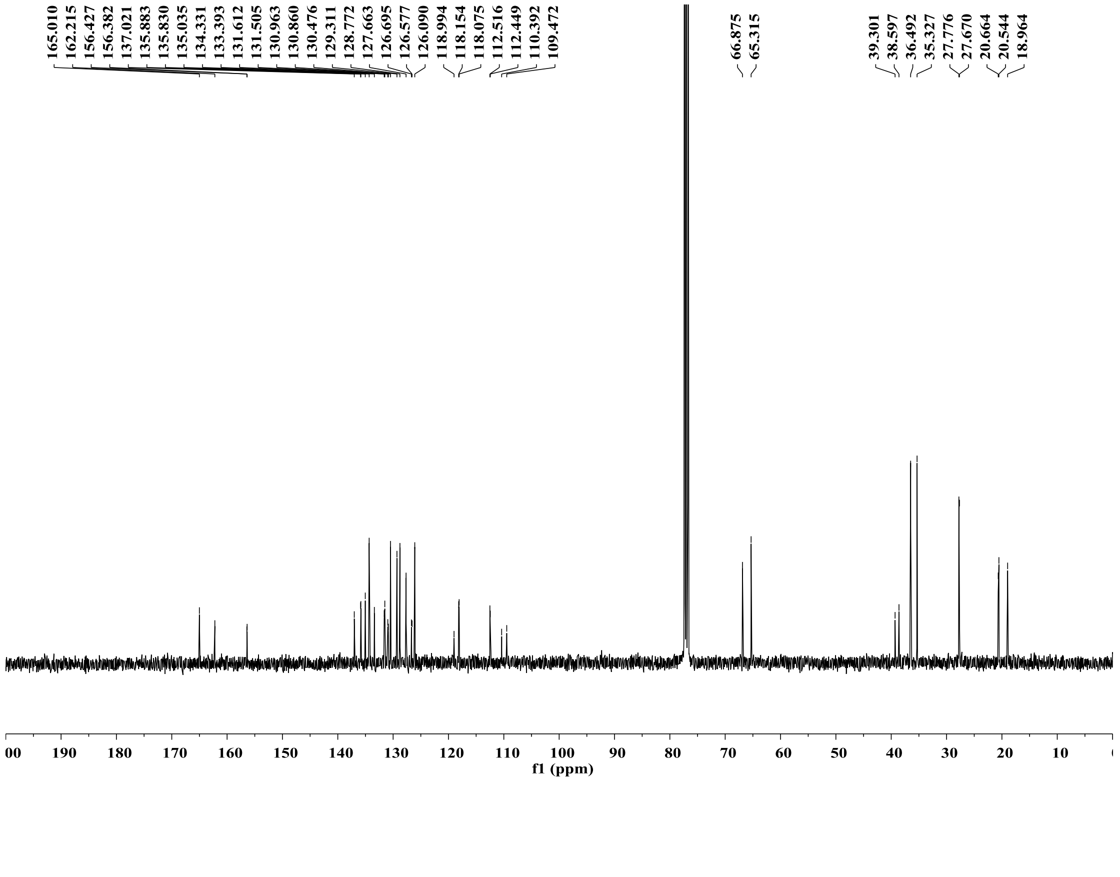
165.010 162.215 156.427 156.382 137.021 135.883 135.830 135.035 134.331 133.393 131.612 131.505 130.963 130.860 130.476 129.311 128.772 127.663 126.695 126.577 126.090 118.994 118.154 118.075 112.516 112.449 110.392 109.472
66.875 65.315
39.301 38.597 36.492 35.327 27.776 27.670 20.664 20.544 18.964
190 180 170 160 150 140 130 120 110 100 90 80 70 60 50 40 30 20 10
f1 (ppm)

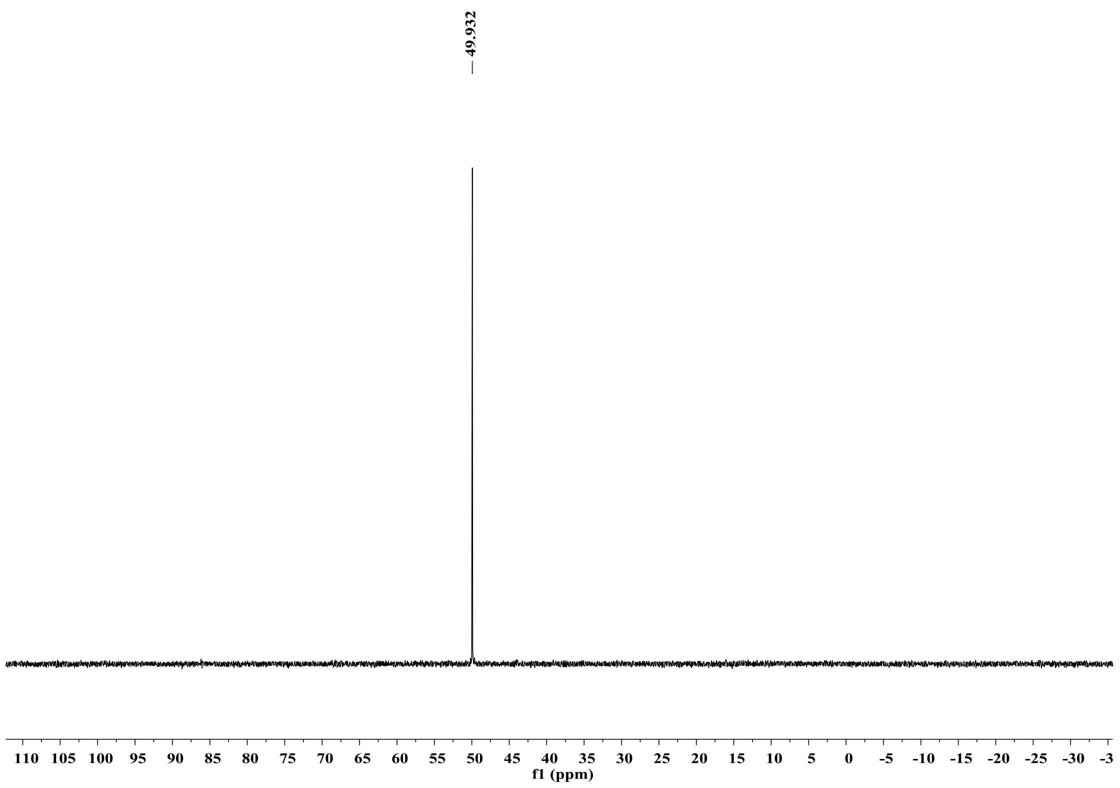

^{1}H NMR/^{13}C NMR/^{31}P NMR of product 3v

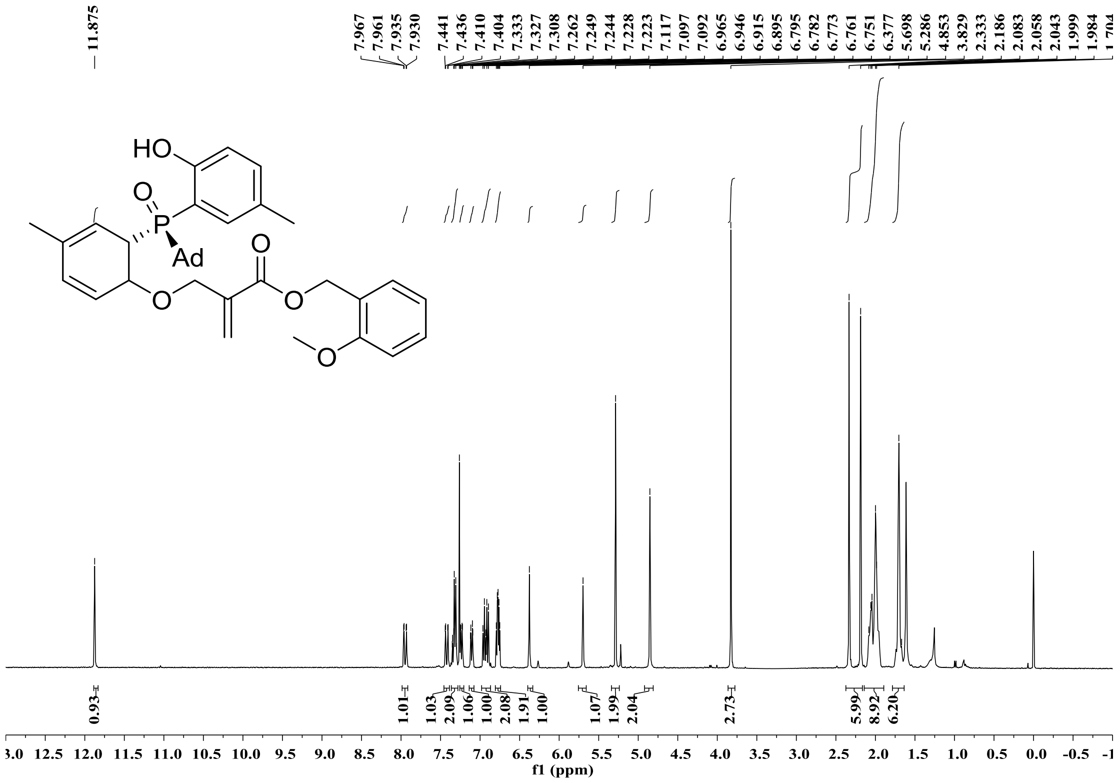

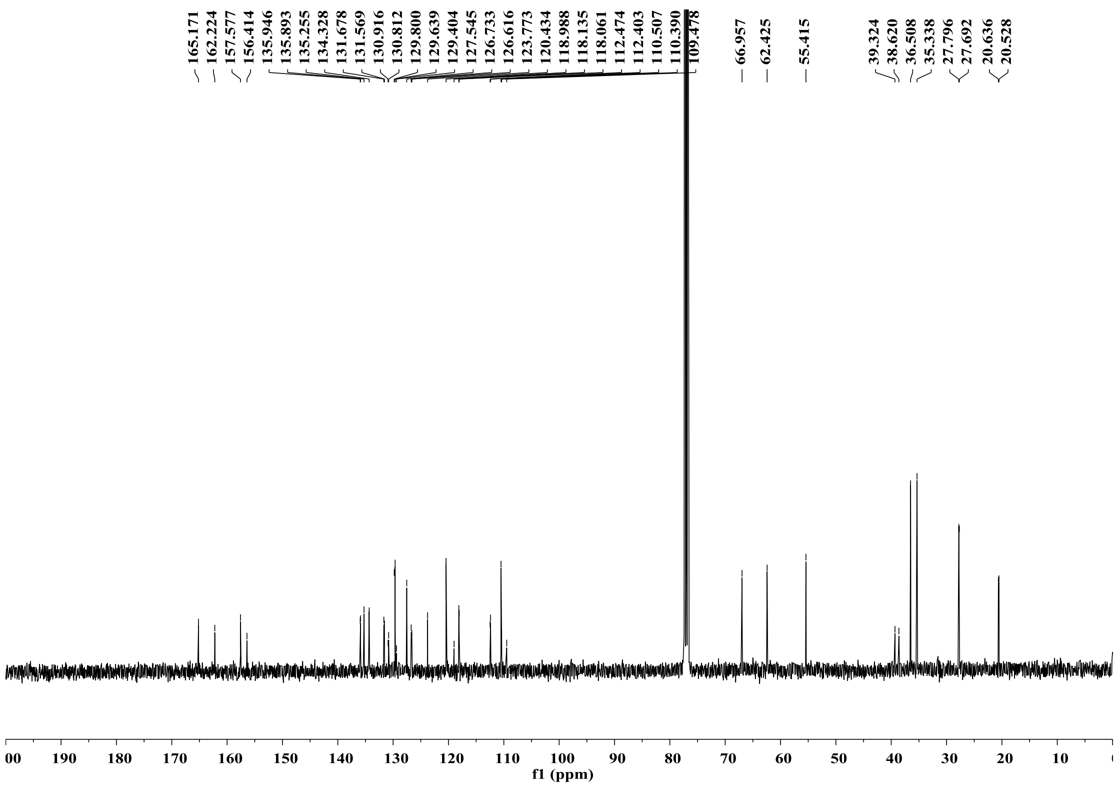

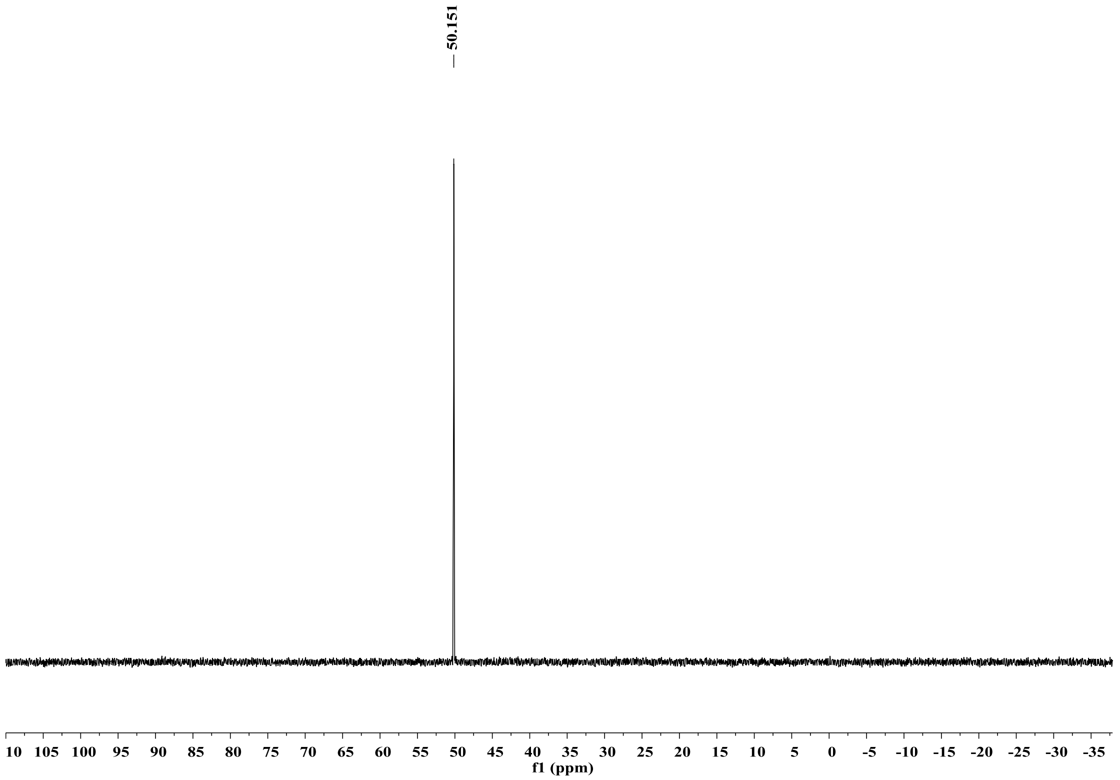

^{1}H NMR/^{13}C NMR/^{31}P NMR of product 3w

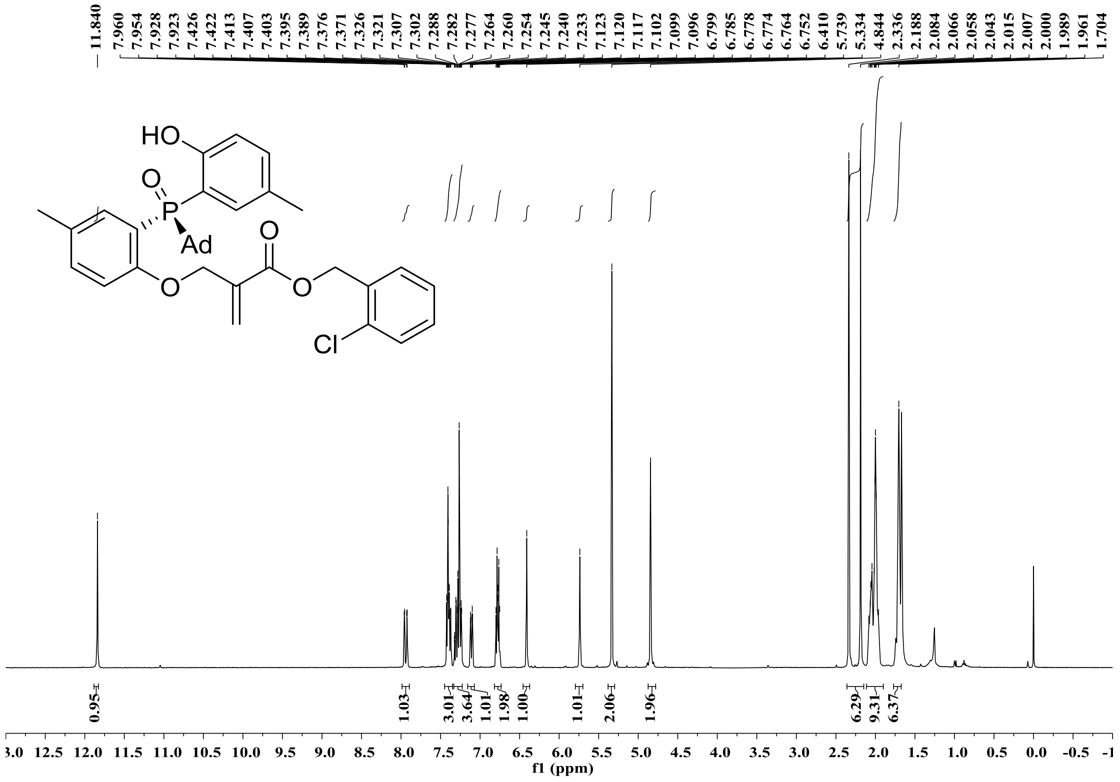

11.840
7.960
7.954
7.928
7.923
7.426
7.422
7.413
7.407
7.403
7.395
7.389
7.376
7.371
7.326
7.321
7.307
7.302
7.288
7.282
7.277
7.264
7.260
7.254
7.245
7.240
7.233
7.123
7.120
7.117
7.102
7.099
7.096
6.799
6.785
6.778
6.774
6.764
6.752
6.410
5.739
5.334
4.844
2.336
2.188
2.084
2.066
2.058
2.043
2.015
2.007
2.000
1.989
1.961
1.704
HO
O
P
Ad
O
O
O
Cl
0.95
1.03
3.01
3.64
1.01
1.98
1.00
1.01
2.06
1.96
6.29
9.31
6.37
f1 (ppm)

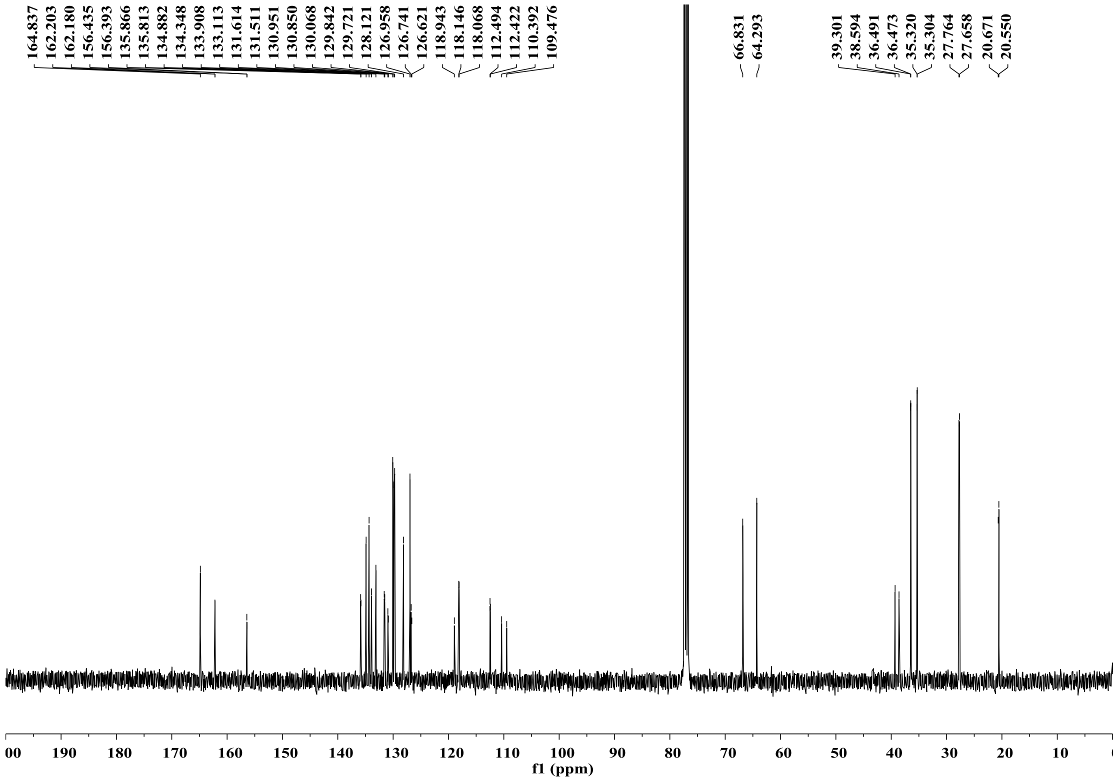

164.837
162.203
162.180
156.435
156.393
135.866
135.813
134.882
134.348
133.908
133.113
131.614
131.511
130.951
130.850
130.068
129.842
129.721
128.121
126.958
126.741
126.621
118.943
118.146
118.068
112.494
112.422
110.392
109.476
66.831
64.293
39.301
38.594
36.491
36.473
35.320
35.304
27.764
27.658
20.671
20.550
f1 (ppm)

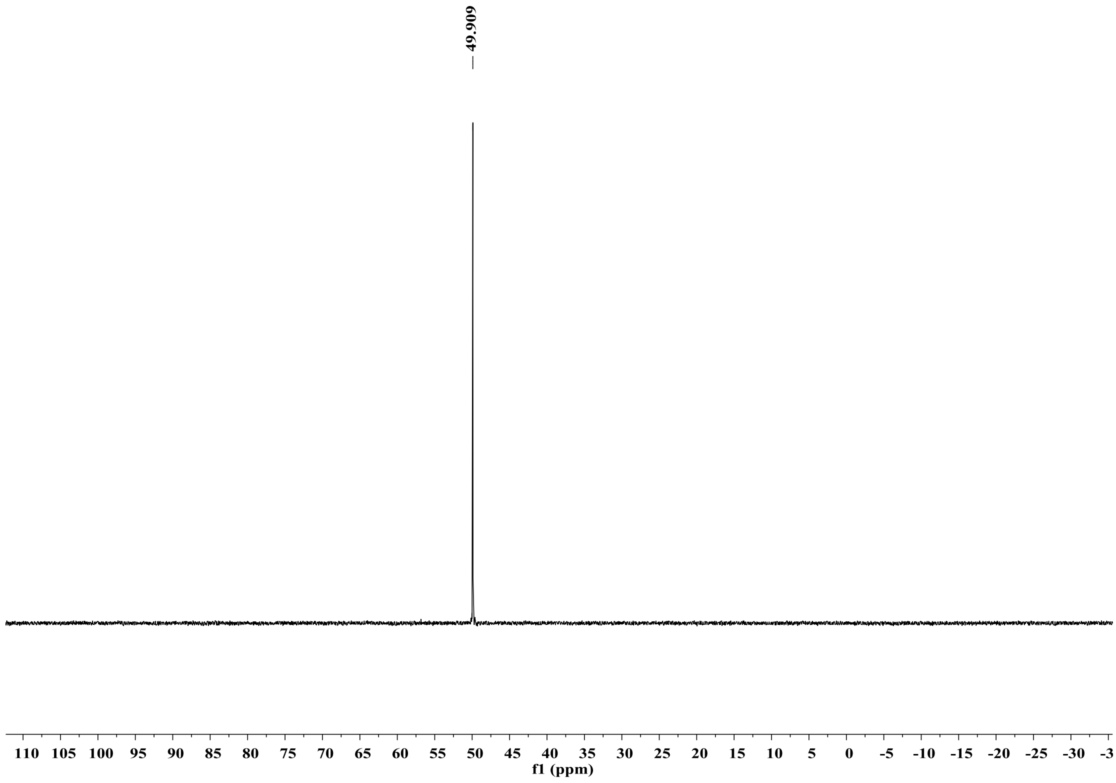

^{1}H NMR/^{13}C NMR/^{31}P NMR of product 3x

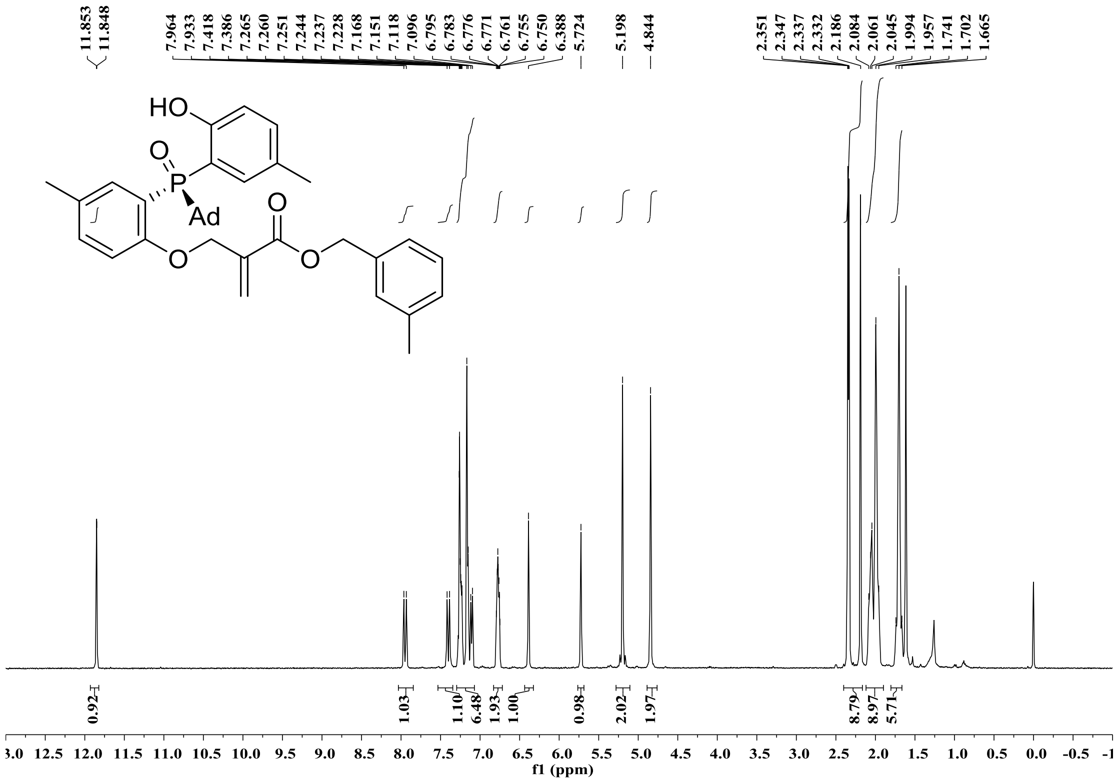

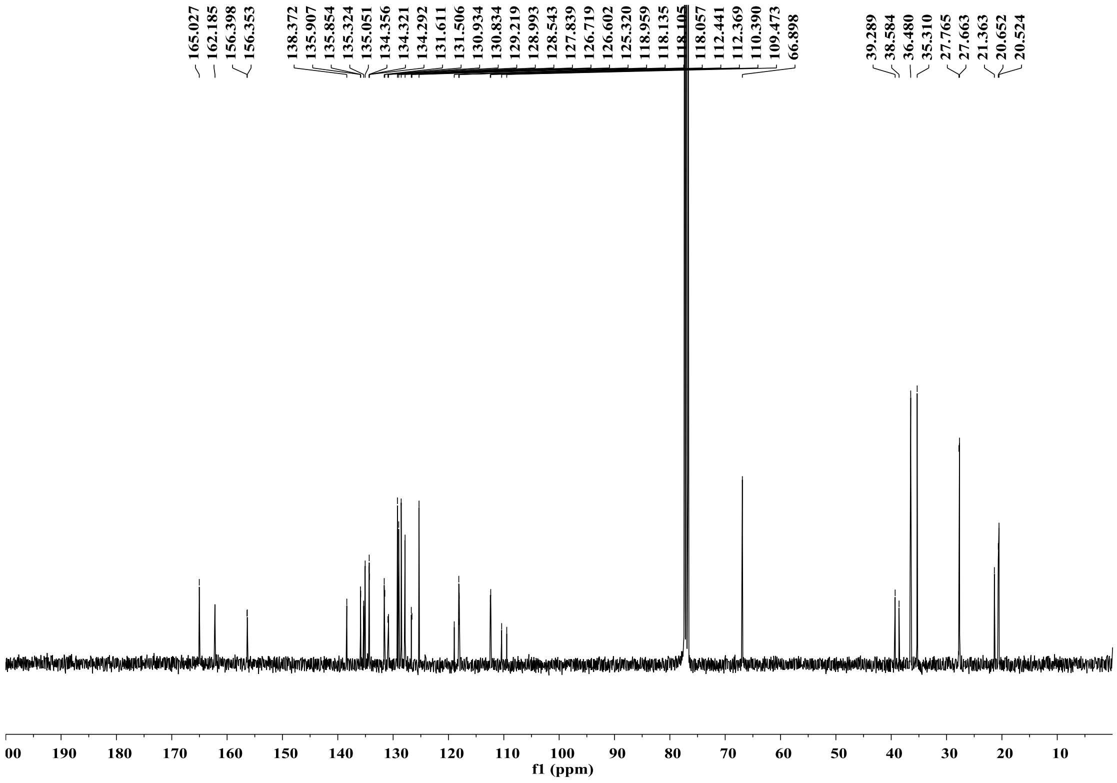

^{1}H NMR/^{13}C NMR/^{31}P NMR of product 3d

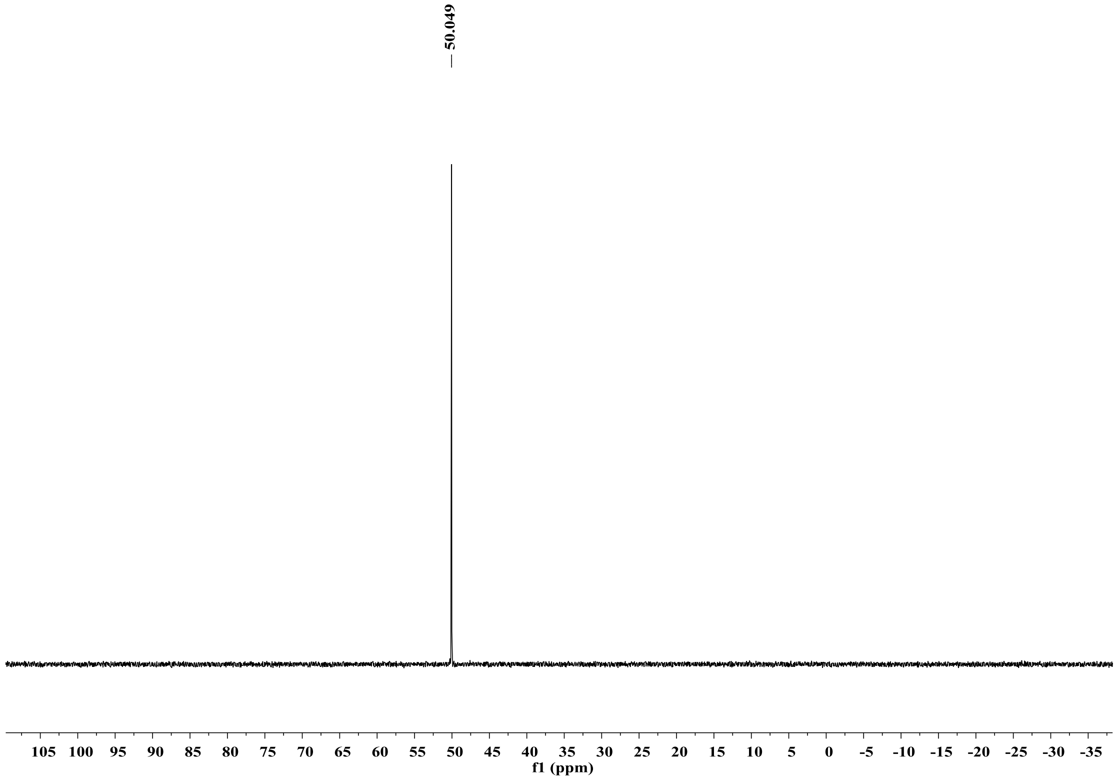

^{1}H NMR/^{13}C NMR/^{31}P NMR of product 3y

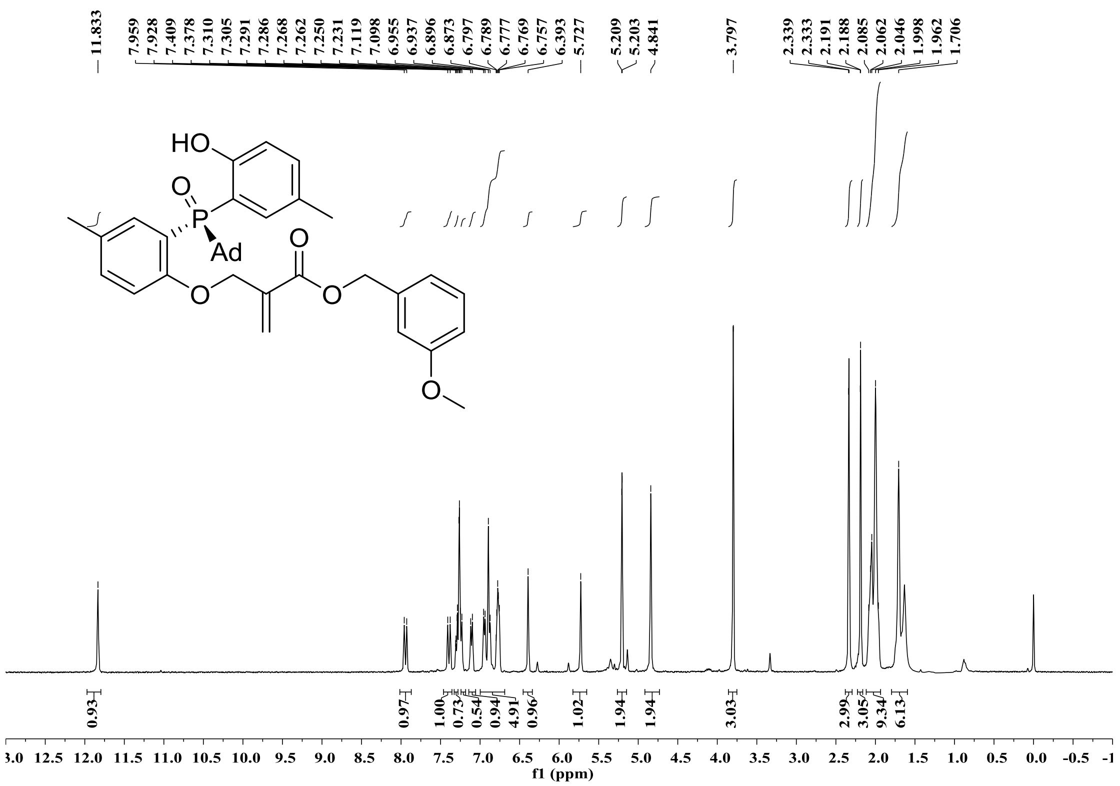
11.833
7.959
7.928
7.409
7.378
7.310
7.305
7.291
7.286
7.268
7.262
7.250
7.231
7.119
7.098
6.955
6.937
6.896
6.873
6.797
6.789
6.777
6.769
6.757
6.393
5.727
5.209
5.203
4.841
3.797
2.339
2.333
2.191
2.188
2.085
2.062
2.046
1.998
1.962
1.706
HO
O
P
Ad
O
O
O
O
0.93
0.97
1.00
0.73
0.54
0.94
4.91
0.96
1.02
1.94
1.94
3.03
2.99
3.05
9.34
6.13
f1 (ppm)

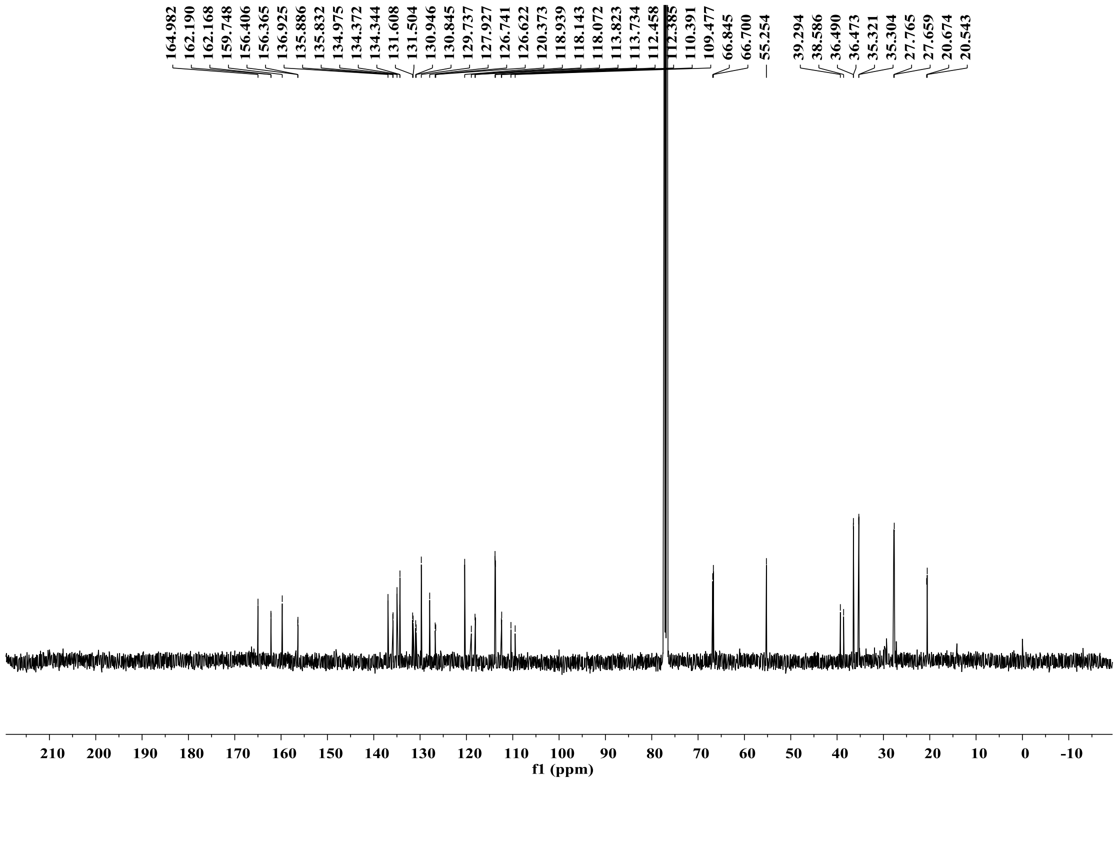
164.982
162.190
162.168
159.748
156.406
156.365
136.925
135.886
135.832
134.975
134.372
134.344
131.608
131.504
130.946
130.845
129.737
127.927
126.741
126.622
120.373
118.939
118.143
118.072
113.823
113.734
112.458
112.385
110.391
109.477
66.845
66.700
55.254
39.294
38.586
36.490
36.473
35.321
35.304
27.765
27.659
20.674
20.543
f1 (ppm)

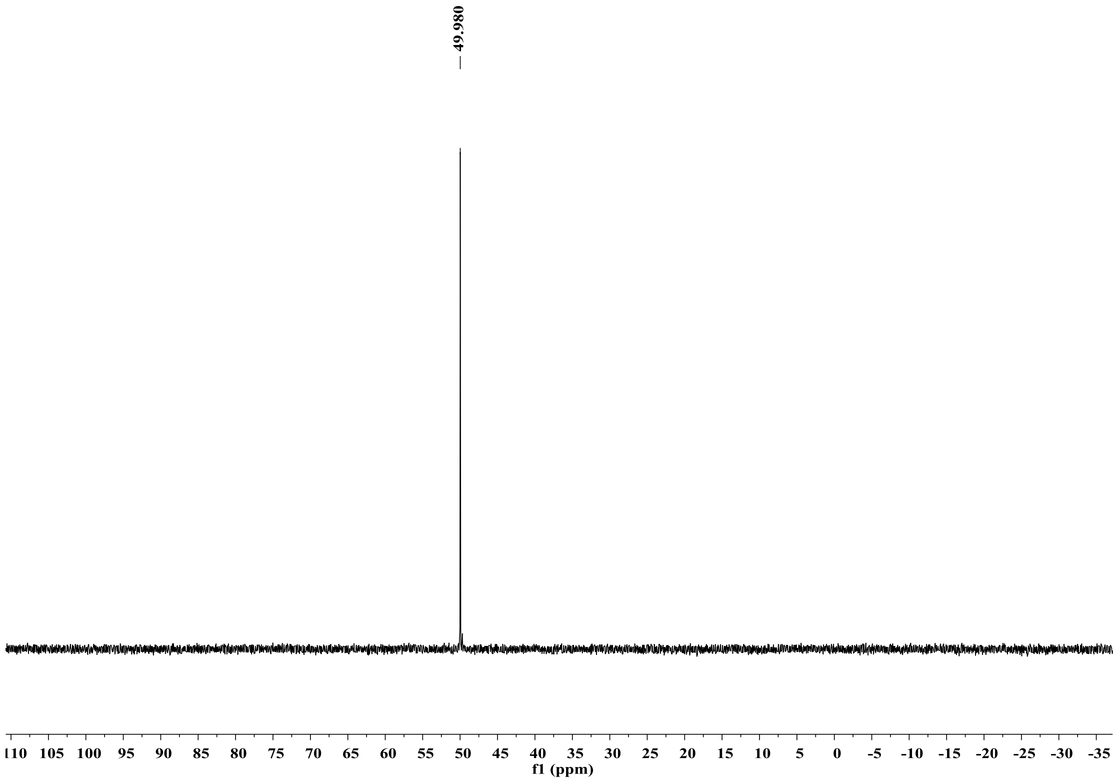

^{1}H NMR/^{13}C NMR/^{31}P NMR of product 3z

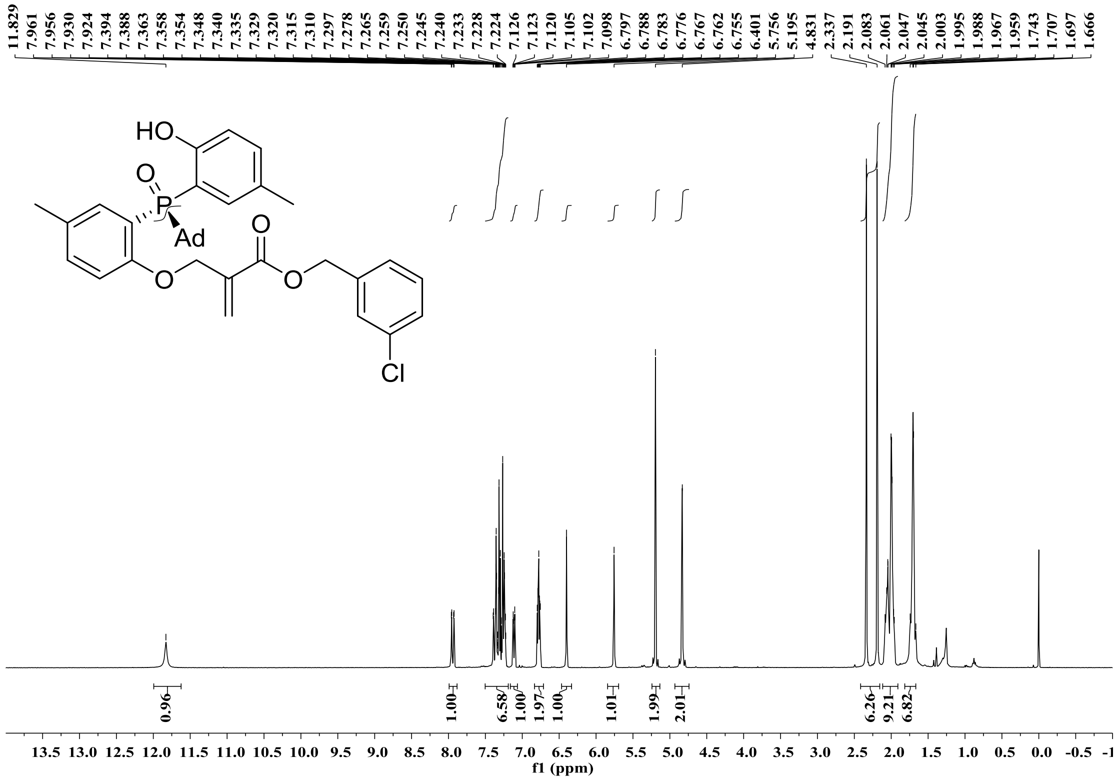

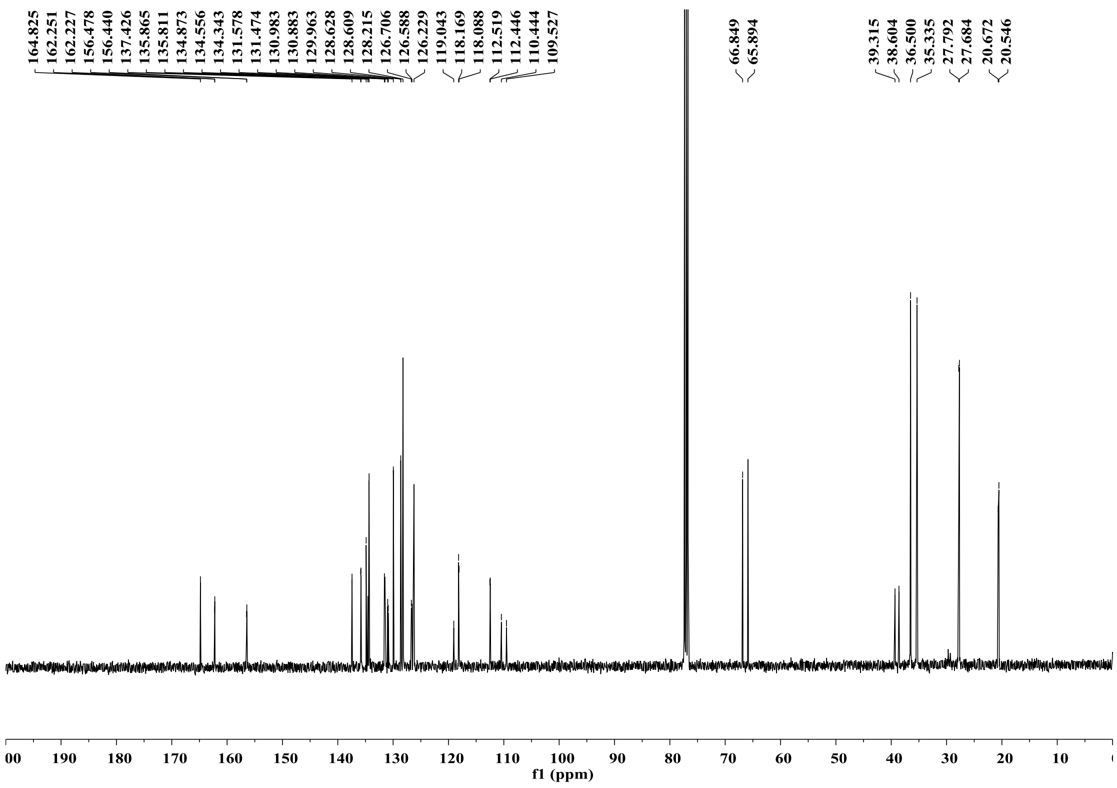

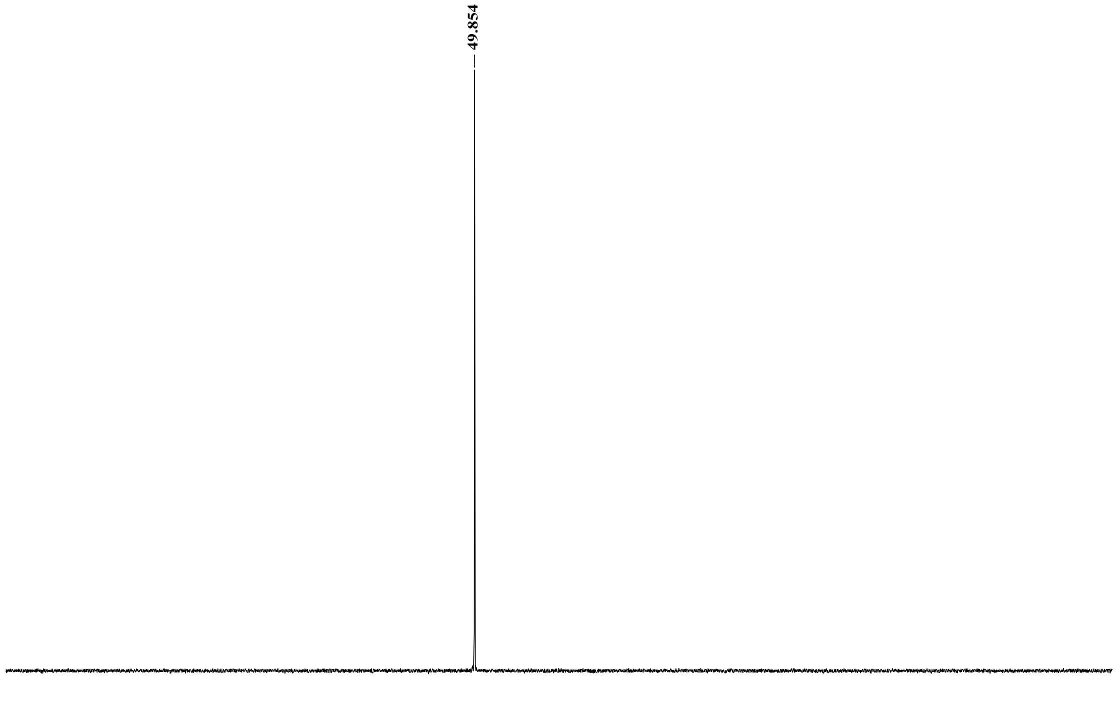

^1H NMR/^{13}C NMR/^{31}P NMR/^{19}F NMR of product 3a’

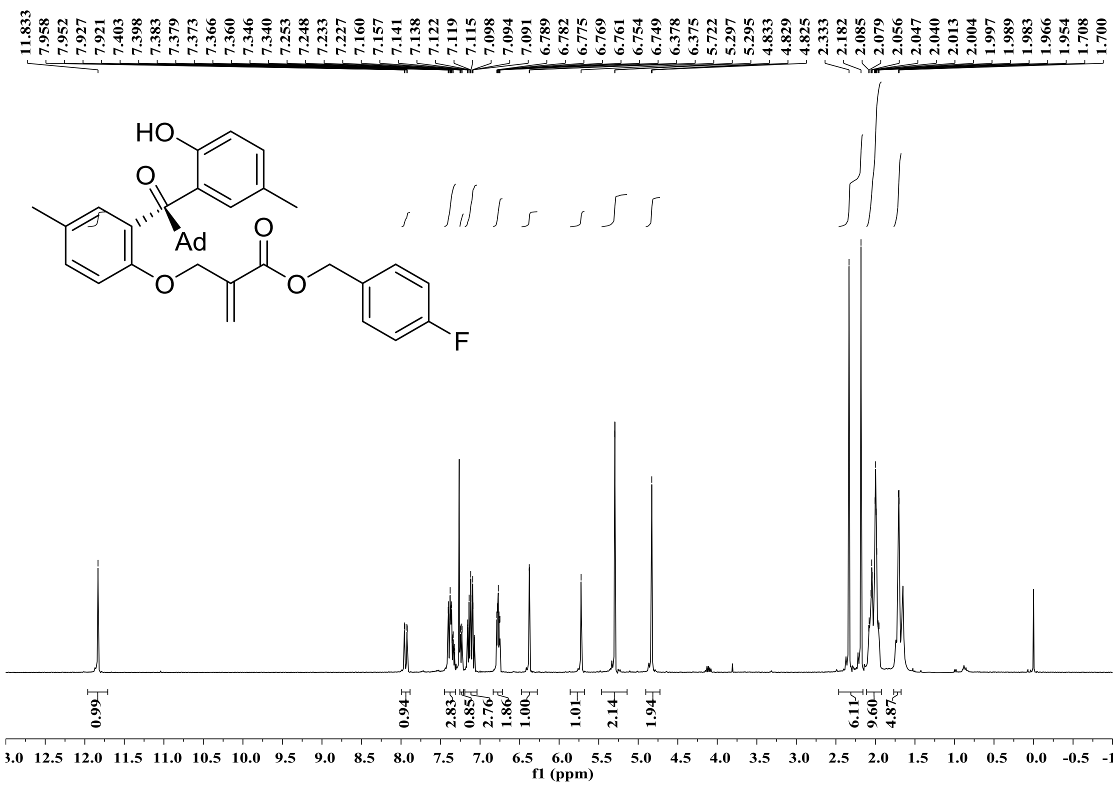
11.833 7.958 7.952 7.927 7.921 7.403 7.398 7.383 7.379 7.373 7.366 7.360 7.346 7.340 7.253 7.248 7.233 7.227 7.160 7.157 7.141 7.138 7.122 7.119 7.115 7.098 7.094 7.091 6.789 6.782 6.775 6.769 6.761 6.754 6.749 6.378 6.375 5.722 5.297 5.295 4.833 4.829 4.825 2.333 2.182 2.085 2.079 2.056 2.047 2.040 2.013 2.004 1.997 1.989 1.983 1.966 1.954 1.708 1.700
HO
O
Ad
O
O
O
F
0.99
0.94
2.83
0.85
2.76
1.86
1.00
1.01
2.14
1.94
6.11
9.60
4.87
f1 (ppm)

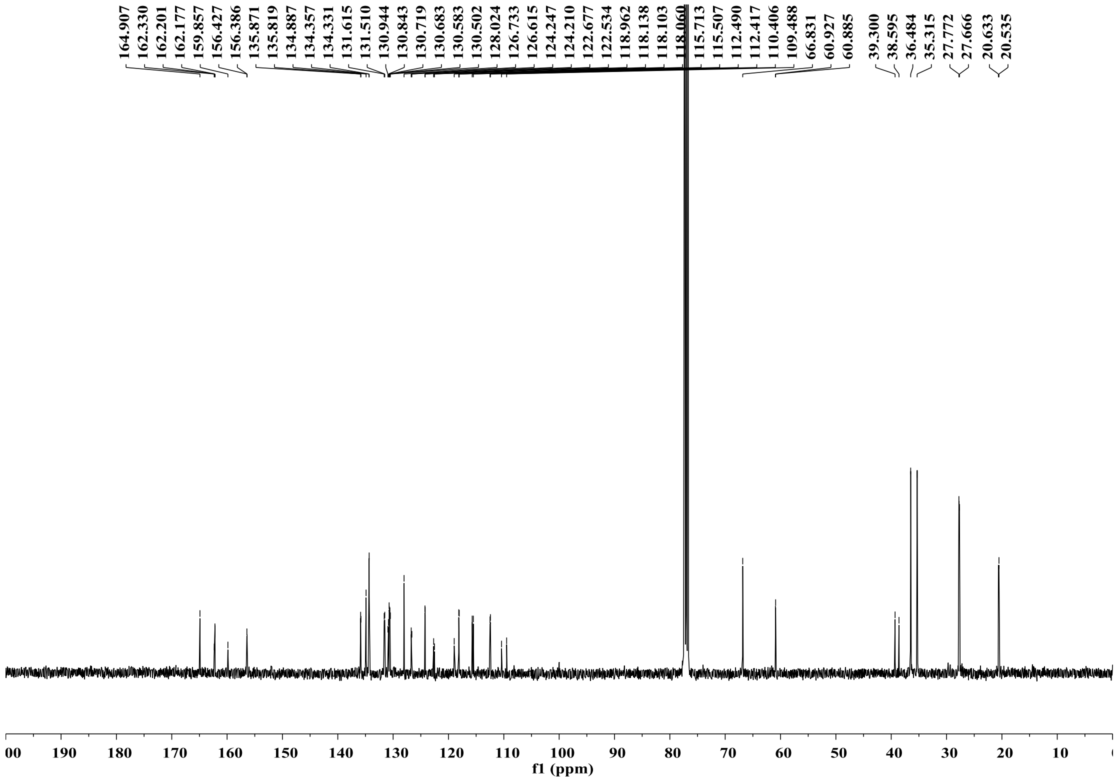
164.907 162.330 162.201 162.177 159.857 156.427 156.386 135.871 135.819 134.887 134.357 134.331 131.615 131.510 130.944 130.843 130.719 130.683 130.583 130.502 128.024 126.733 126.615 124.247 124.210 122.677 122.534 118.962 118.138 118.103 118.060 115.713 115.507 112.490 112.417 110.406 109.488 66.831 60.927 60.885 39.300 38.595 36.484 35.315 27.772 27.666 20.633 20.535
f1 (ppm)

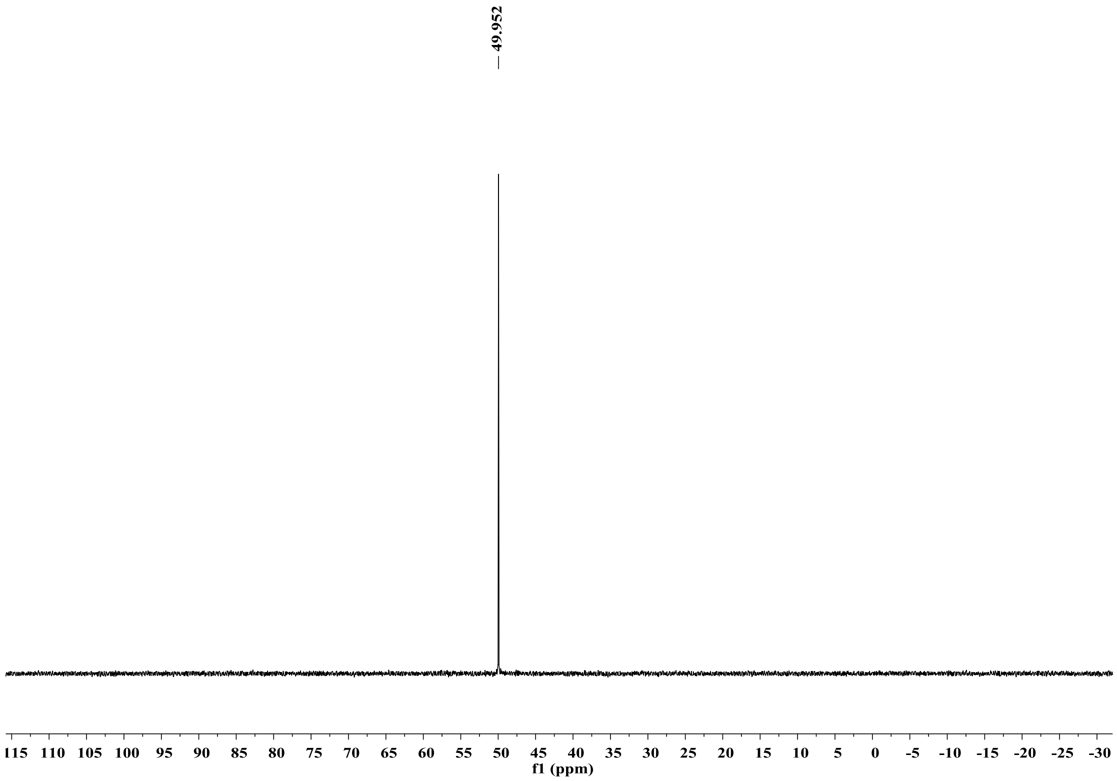

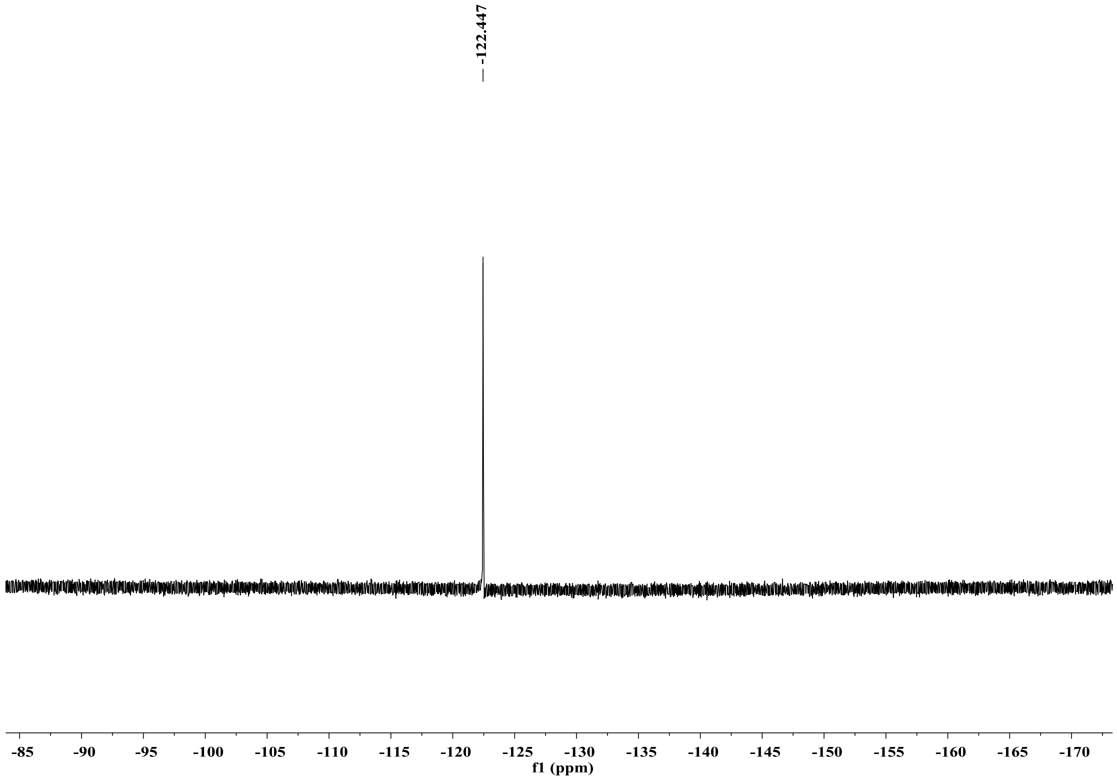

^{1}H NMR/^{13}C NMR/^{31}P NMR of product 3b’

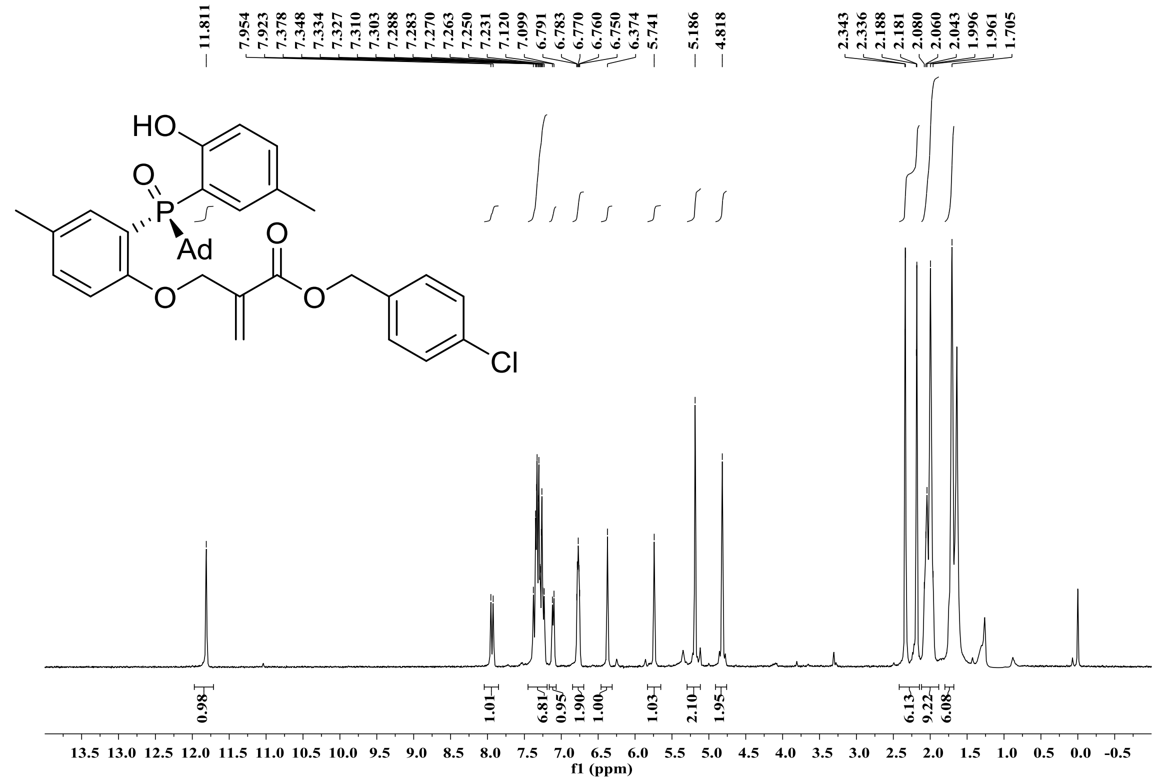
11.811
7.954
7.923
7.378
7.348
7.334
7.327
7.310
7.303
7.288
7.283
7.270
7.263
7.250
7.231
7.120
7.099
6.791
6.783
6.770
6.760
6.750
6.374
5.741
5.186
4.818
2.343
2.336
2.188
2.181
2.080
2.060
2.043
1.996
1.961
1.705
HO
O
P
Ad
O
O
O
Cl
0.98
1.01
6.81
0.95
1.90
1.00
1.03
2.10
1.95
6.13
9.22
6.08
13.5 13.0 12.5 12.0 11.5 11.0 10.5 10.0 9.5 9.0 8.5 8.0 7.5 7.0 6.5 6.0 5.5 5.0 4.5 4.0 3.5 3.0 2.5 2.0 1.5 1.0 0.5 0.0 -0.5
f1 (ppm)

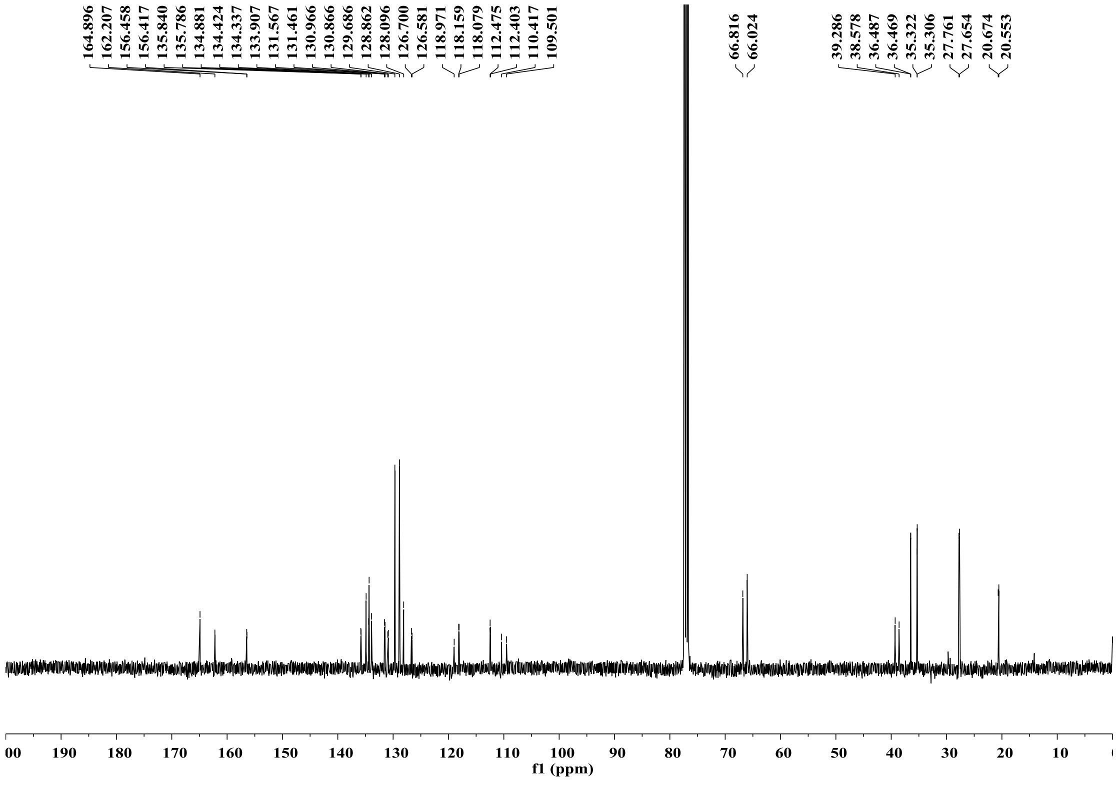
164.896
162.207
156.458
156.417
135.840
135.786
134.881
134.424
134.337
133.907
131.567
131.461
130.966
130.866
129.686
128.862
128.096
126.700
126.581
118.971
118.159
118.079
112.475
112.403
110.417
109.501
66.816
66.024
39.286
38.578
36.487
36.469
35.322
35.306
27.761
27.654
20.674
20.553
00 190 180 170 160 150 140 130 120 110 100 90 80 70 60 50 40 30 20 10
f1 (ppm)

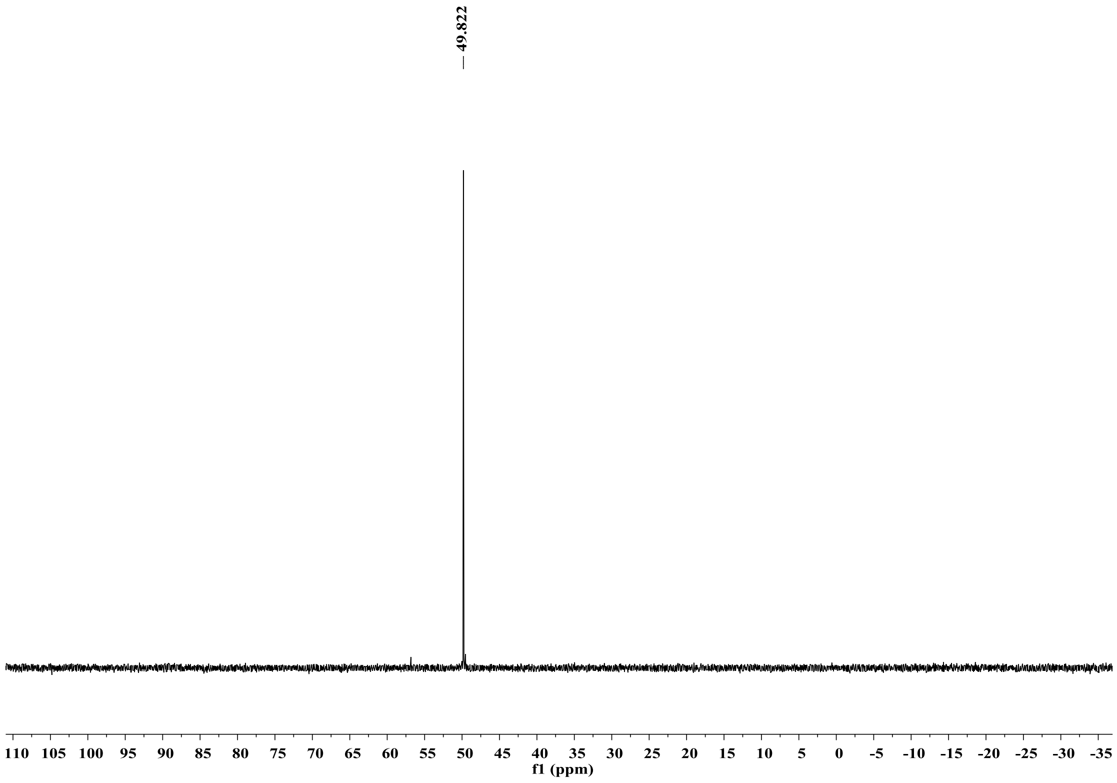

^1H NMR/^{13}C NMR/^{31}P NMR of product 3c'

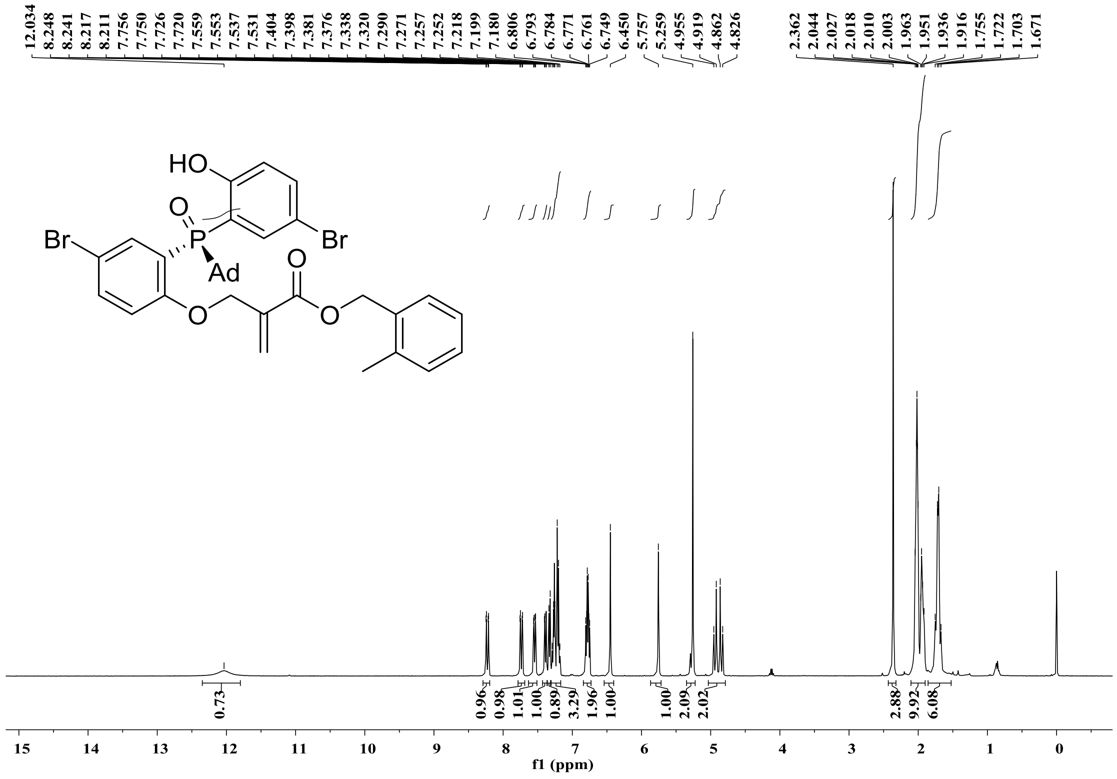

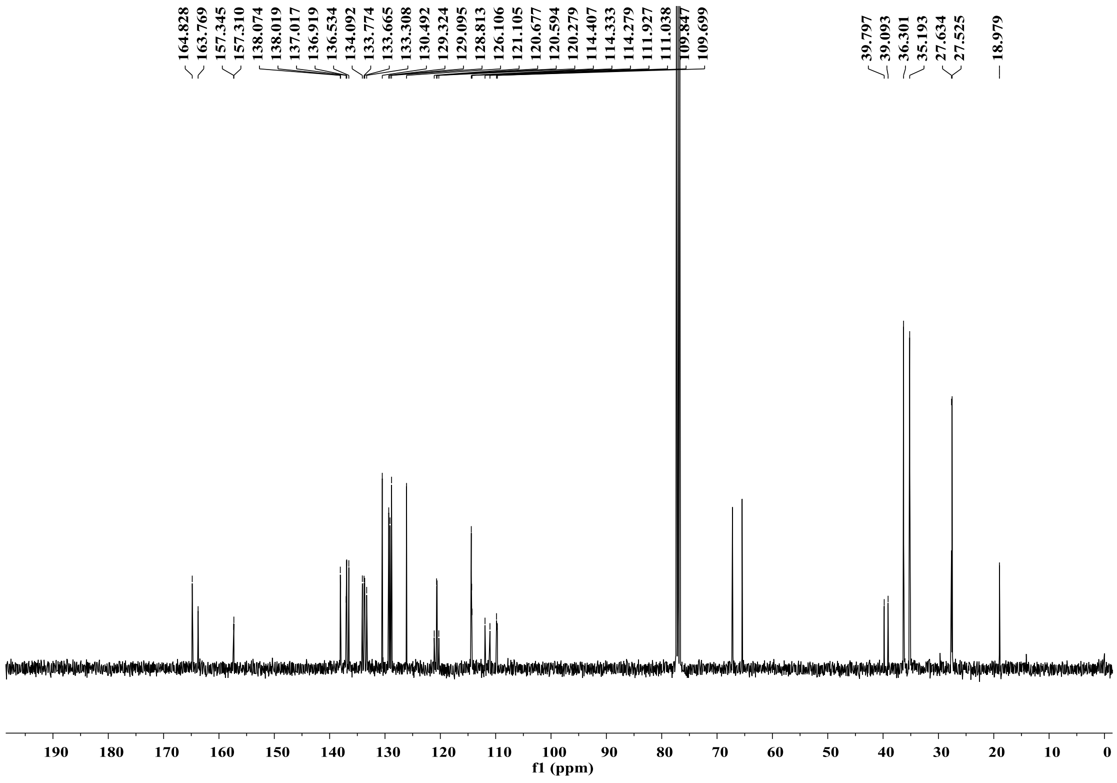

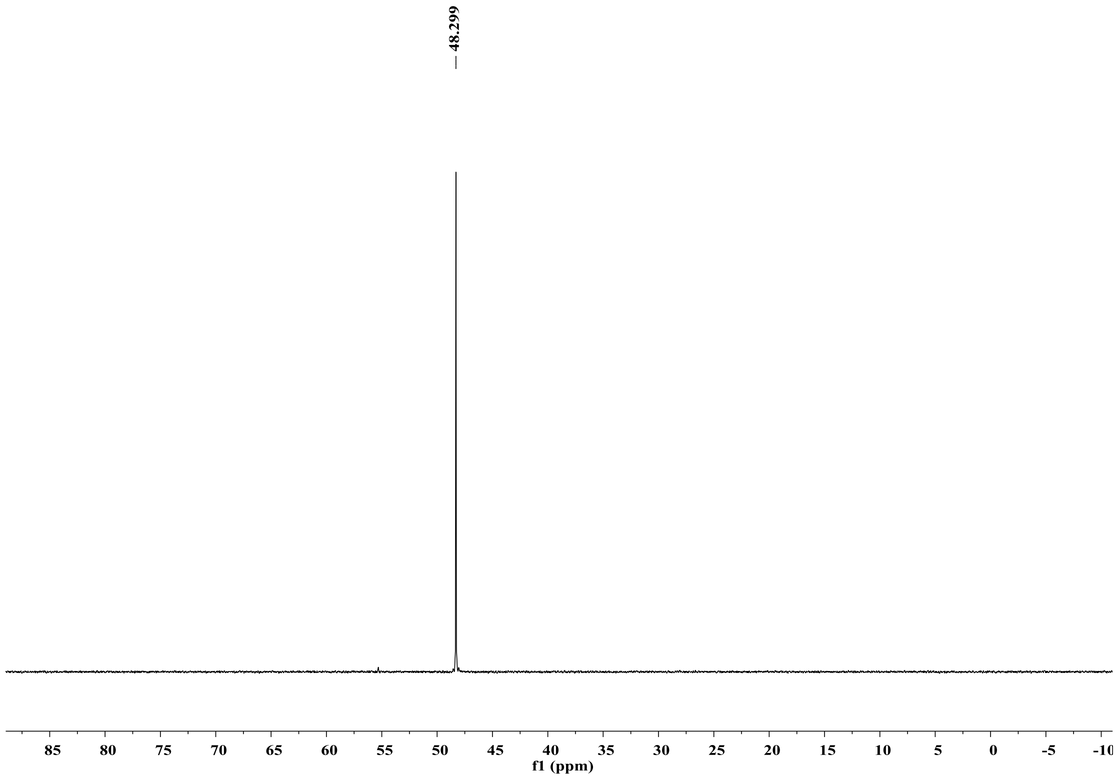

^{1}H NMR/^{13}C NMR/^{31}P NMR of product 3d’

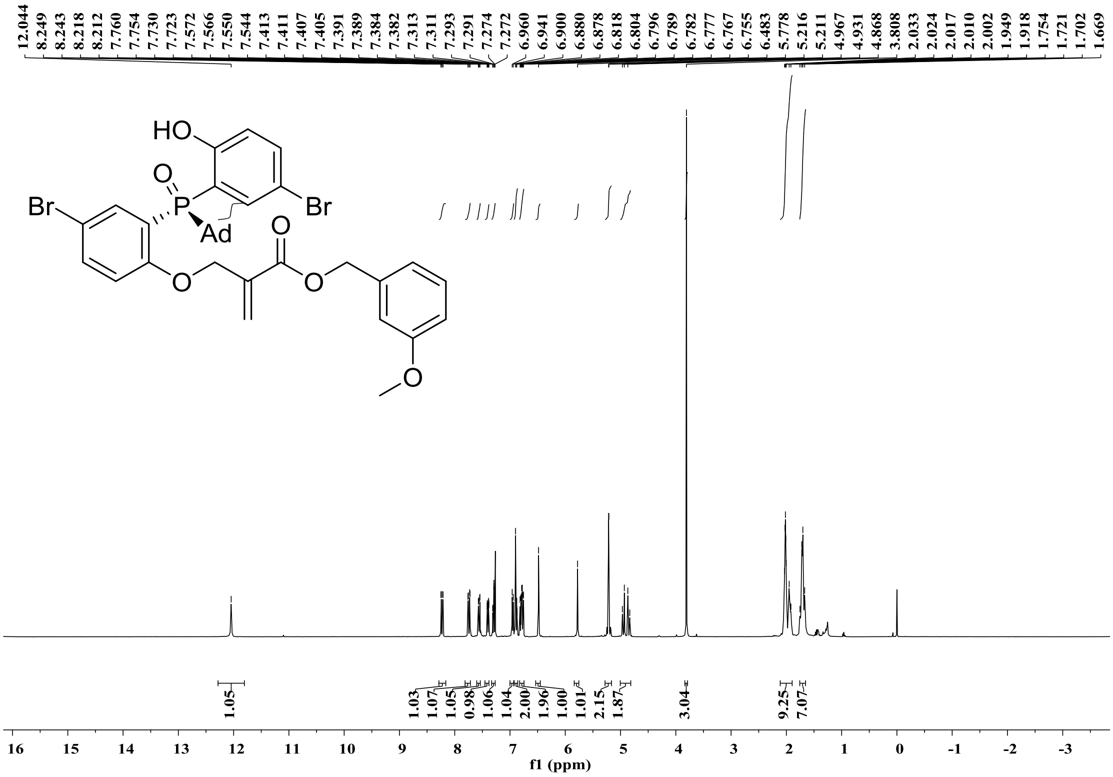
12.044 8.249 8.243 8.218 8.212 7.760 7.754 7.730 7.723 7.572 7.566 7.550 7.544 7.413 7.411 7.407 7.405 7.391 7.389 7.384 7.382 7.313 7.311 7.293 7.291 7.274 7.272 6.960 6.941 6.900 6.880 6.878 6.818 6.804 6.796 6.789 6.782 6.777 6.767 6.755 6.483 5.778 5.216 5.211 4.967 4.931 4.868 3.808 2.033 2.024 2.017 2.010 2.002 1.949 1.918 1.754 1.721 1.702 1.669
HO
O
P
Br
Br
Ad
O
O
O
O
1.05
1.03 1.07 1.05 0.98 1.06 1.04 2.00 1.96 1.00 1.01 2.15 1.87
3.04
9.25 7.07
16 15 14 13 12 11 10 9 8 7 6 5 4 3 2 1 0 -1 -2 -3
f1 (ppm)

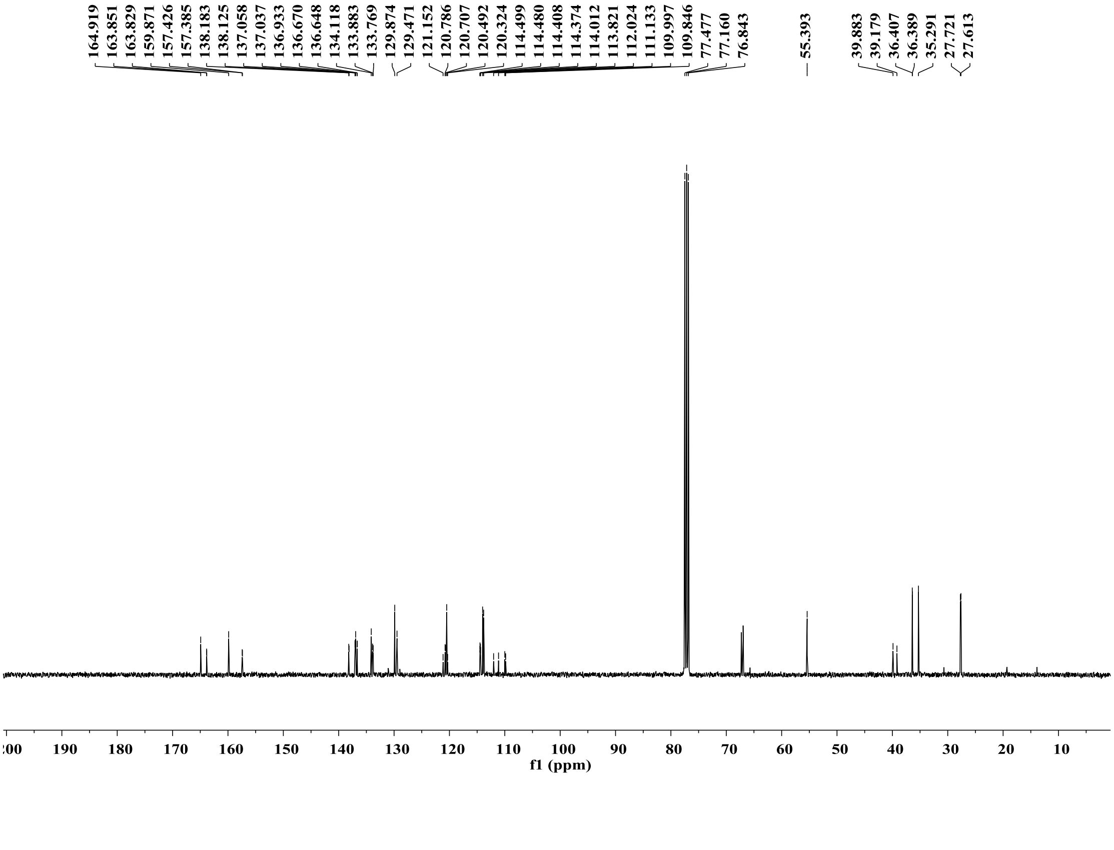
164.919 163.851 163.829 159.871 157.426 157.385 138.183 138.125 137.058 137.037 136.933 136.670 136.648 134.118 133.883 133.769 129.874 129.471 121.152 120.786 120.707 120.492 120.324 114.499 114.480 114.408 114.374 114.012 113.821 112.024 111.133 109.997 109.846 77.477 77.160 76.843
55.393
39.883 39.179 36.407 36.389 35.291 27.721 27.613
200 190 180 170 160 150 140 130 120 110 100 90 80 70 60 50 40 30 20 10
f1 (ppm)

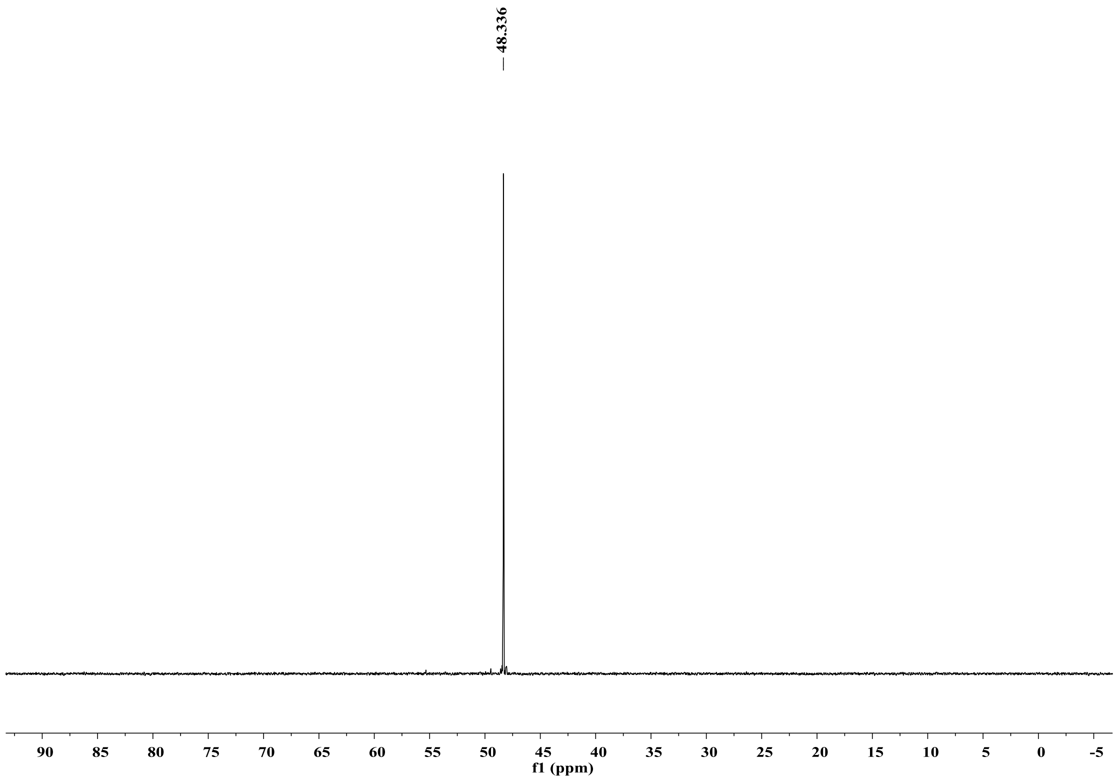

^{1}H NMR/^{13}C NMR/^{31}P NMR/^{19}F NMR of product 3e'

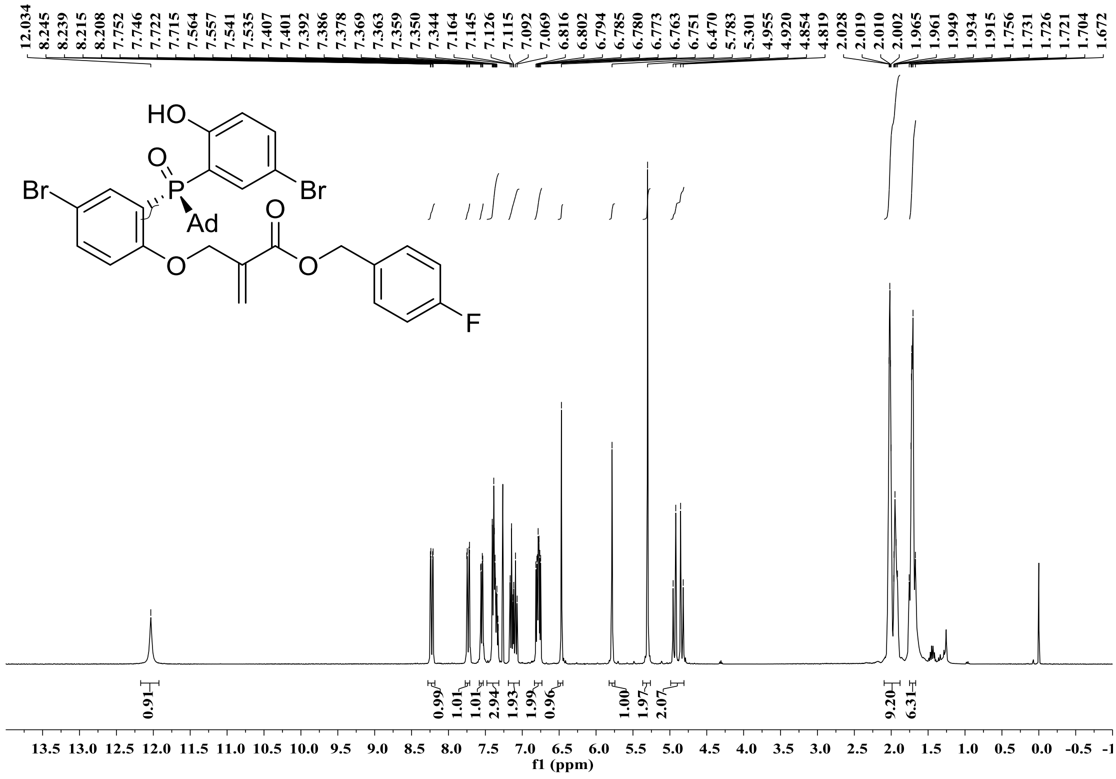

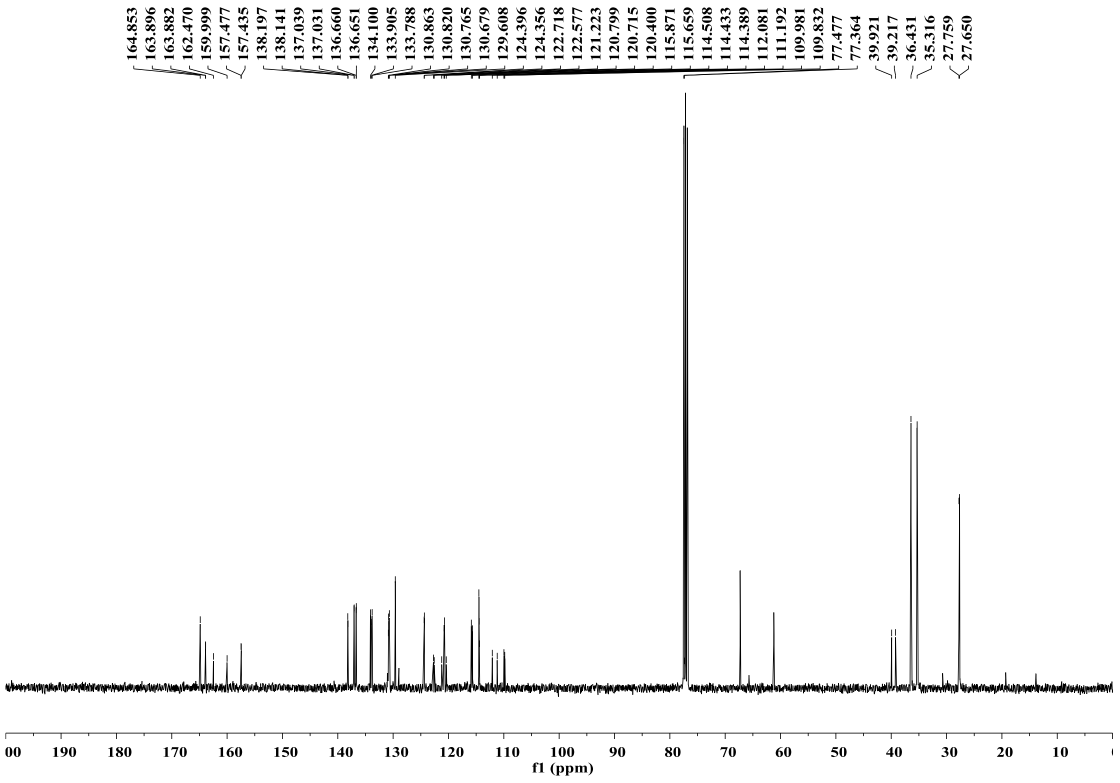

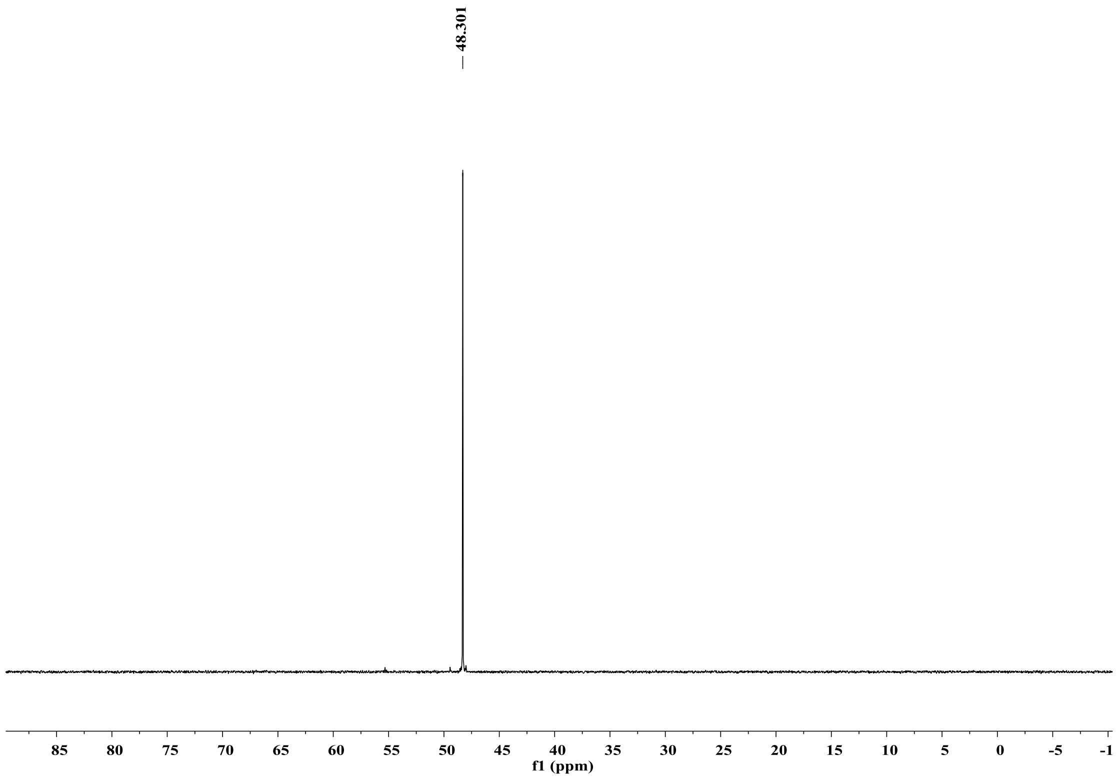

^{1}H NMR/^{13}C NMR/^{31}P NMR of product 4a'

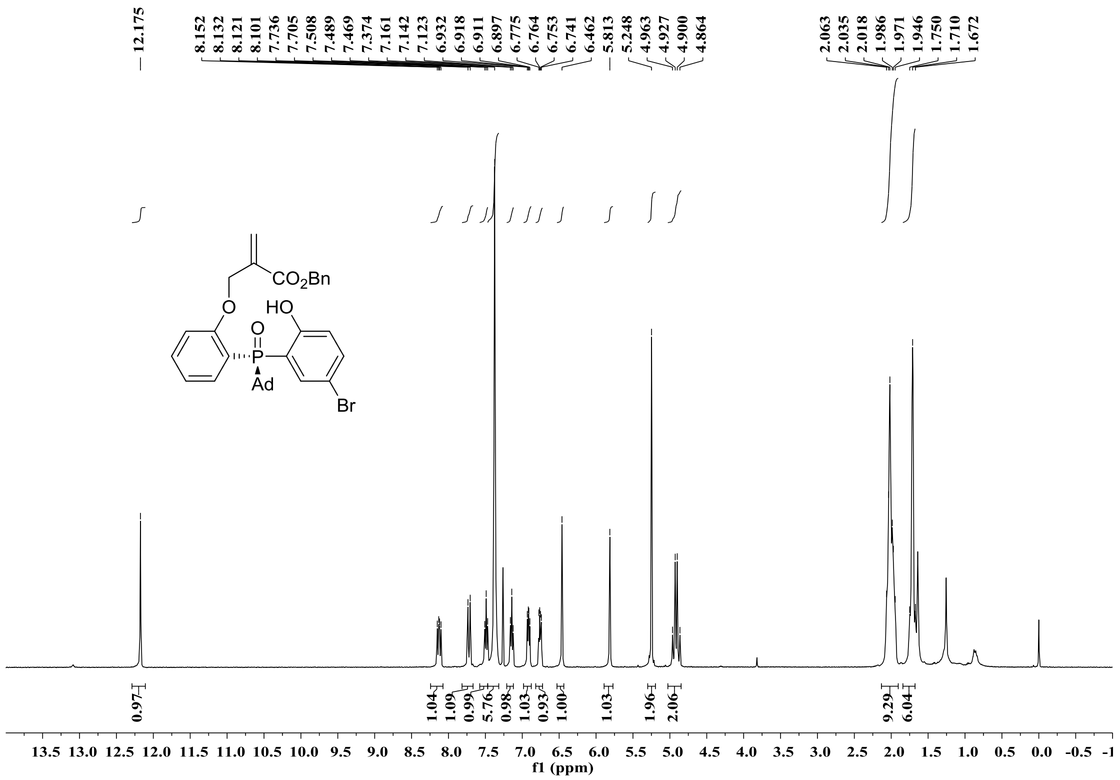
12.175
8.152
8.132
8.121
8.101
7.736
7.705
7.508
7.489
7.469
7.374
7.161
7.142
7.123
6.932
6.918
6.911
6.897
6.775
6.764
6.753
6.741
6.462
5.813
5.248
4.963
4.927
4.900
4.864
2.063
2.035
2.018
1.986
1.971
1.946
1.750
1.710
1.672
CO2Bn
O
HO
O
P
Ad
Br
0.97
1.04
1.09
0.99
5.76
0.98
1.03
0.93
1.00
1.03
1.96
2.06
9.29
6.04
f1 (ppm)

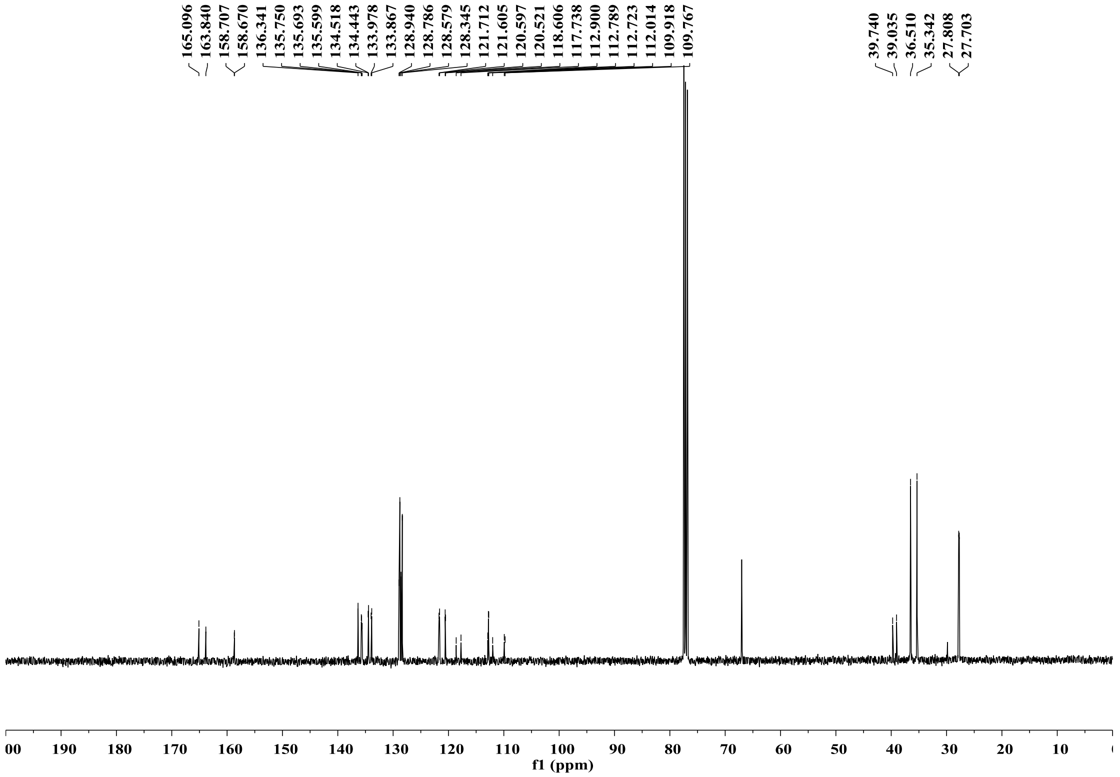
165.096
163.840
158.707
158.670
136.341
135.750
135.693
135.599
134.518
134.443
133.978
133.867
128.940
128.786
128.579
128.345
121.712
121.605
120.597
120.521
118.606
117.738
112.900
112.789
112.723
112.014
109.918
109.767
39.740
39.035
36.510
35.342
27.808
27.703
f1 (ppm)

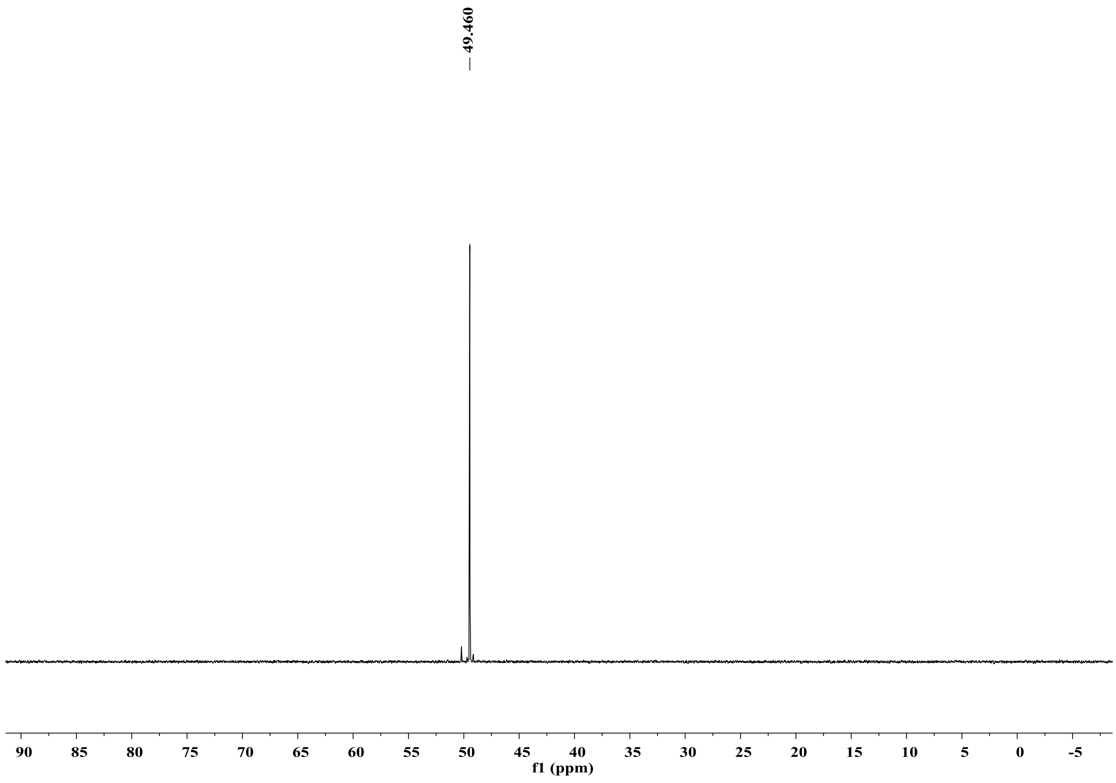

^{1}H NMR/^{13}C NMR/^{31}P NMR of product 4a

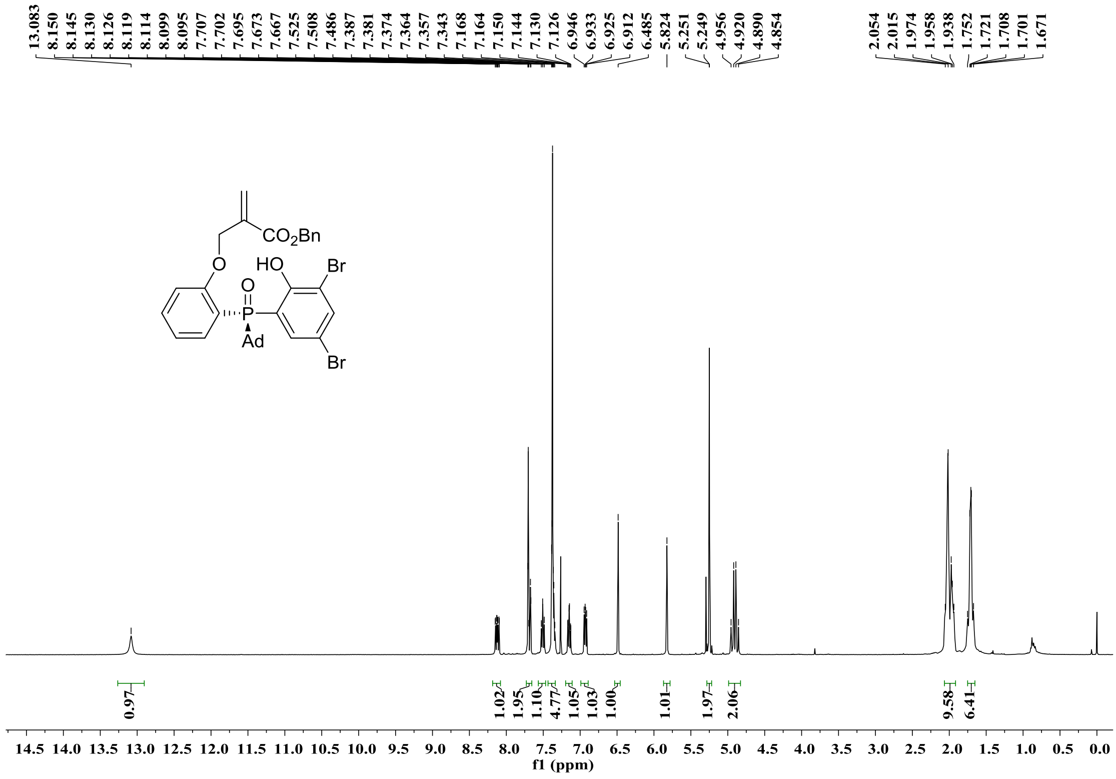

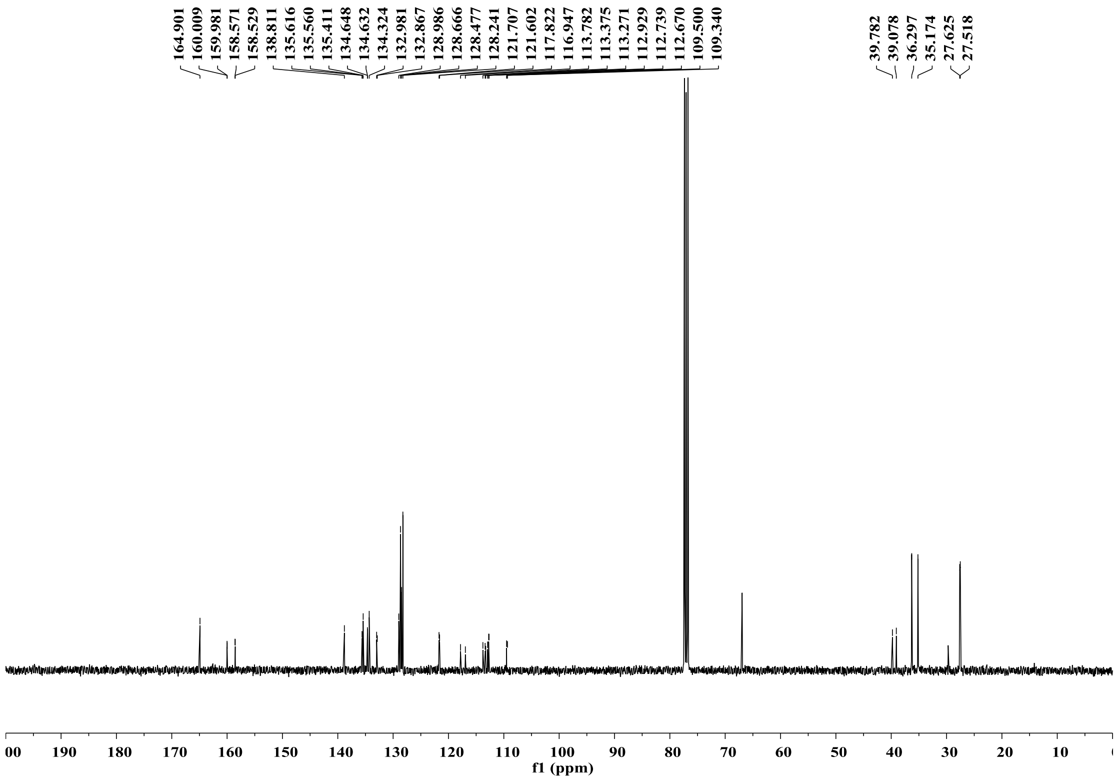

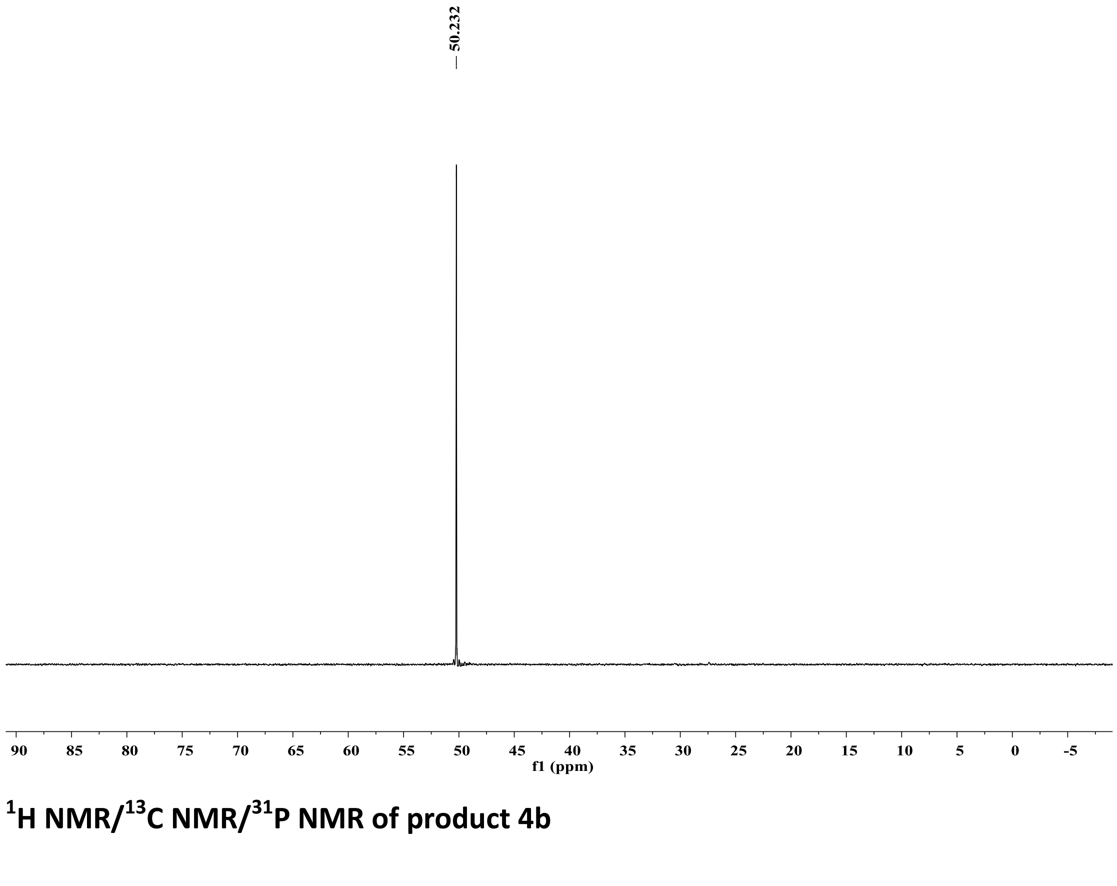

^{1}H NMR/^{13}C NMR/^{31}P NMR of product 4b

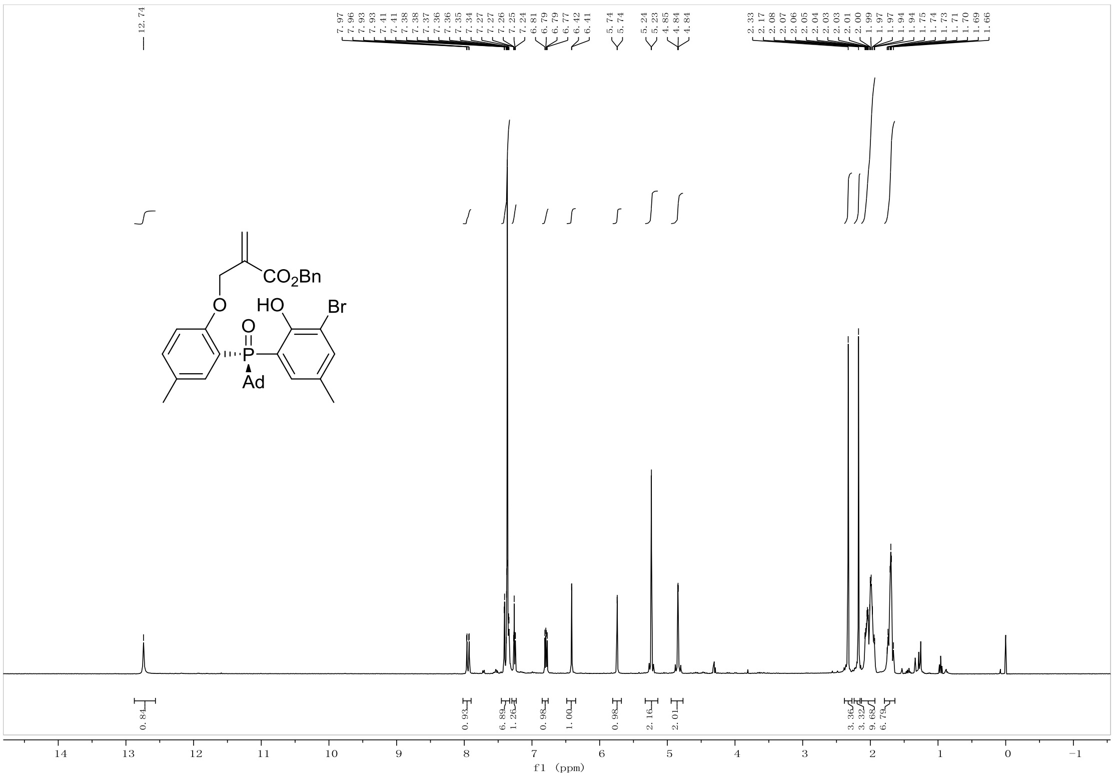

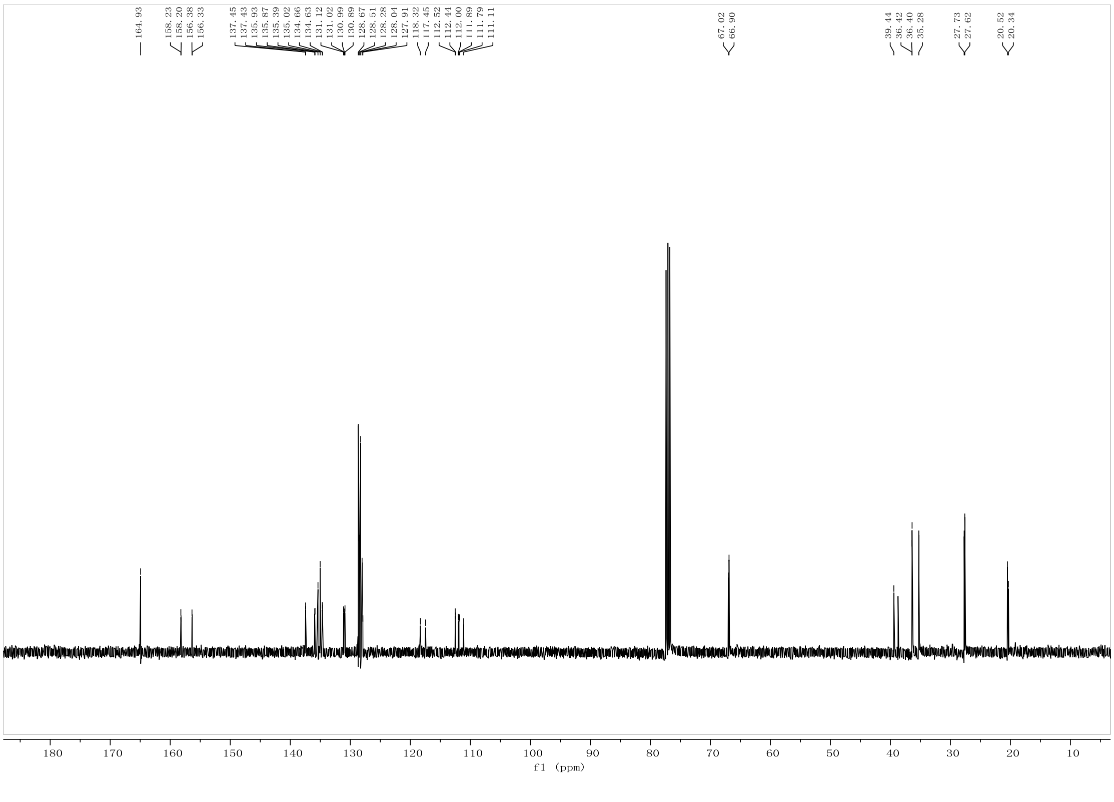

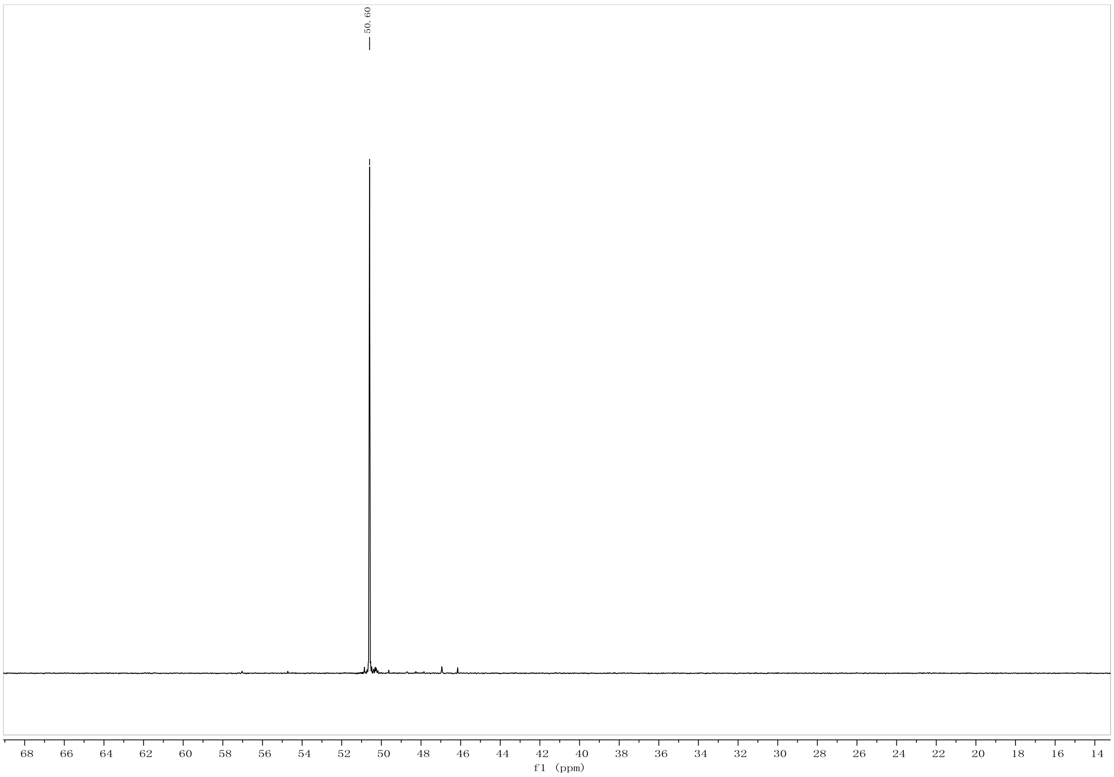

^{1}H NMR/^{13}C NMR/^{31}P NMR of product 5a

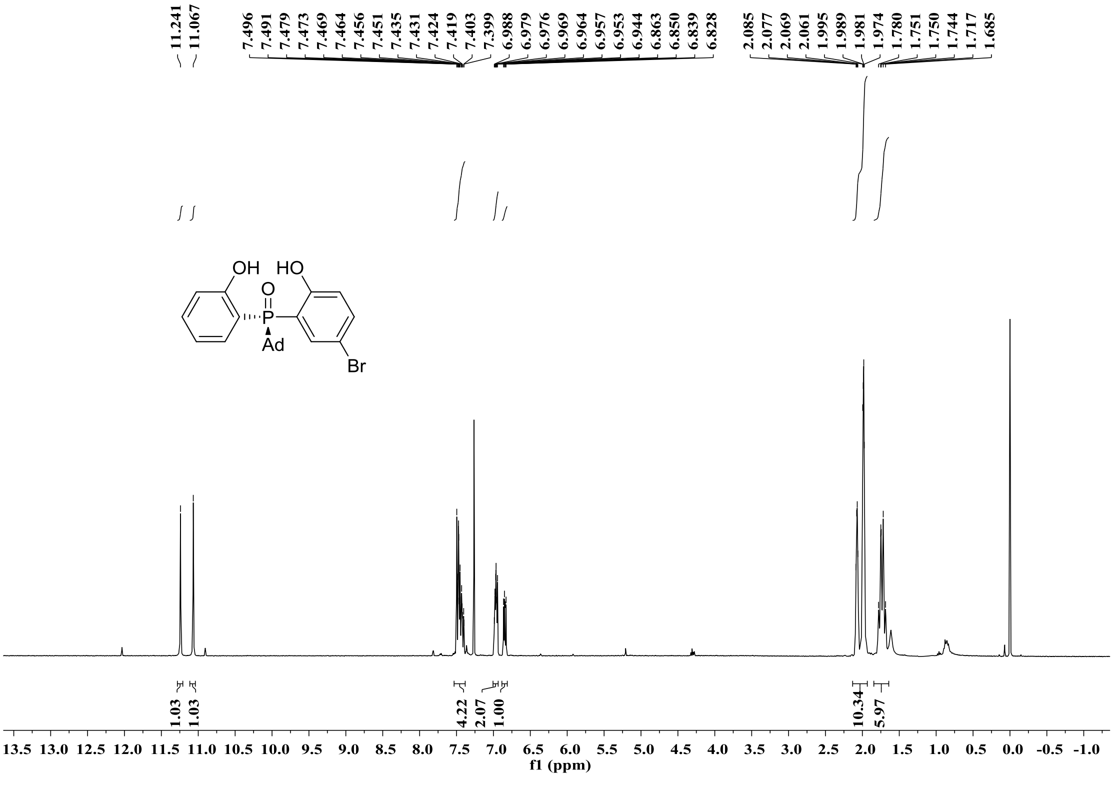

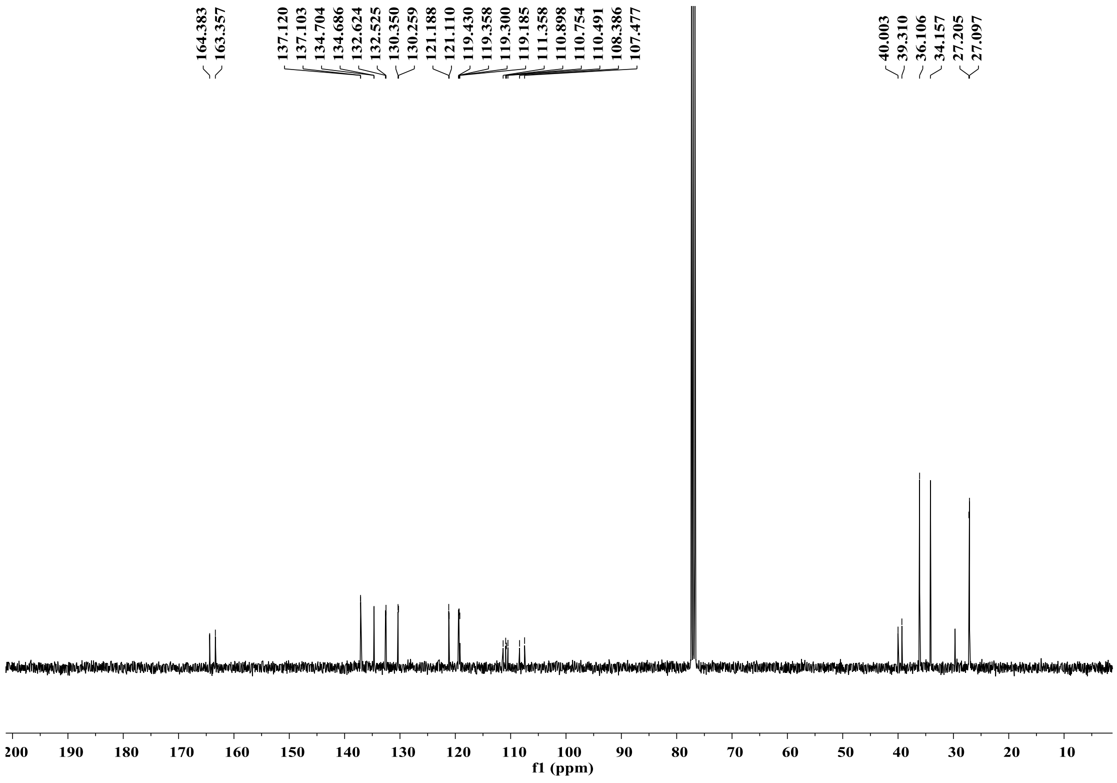

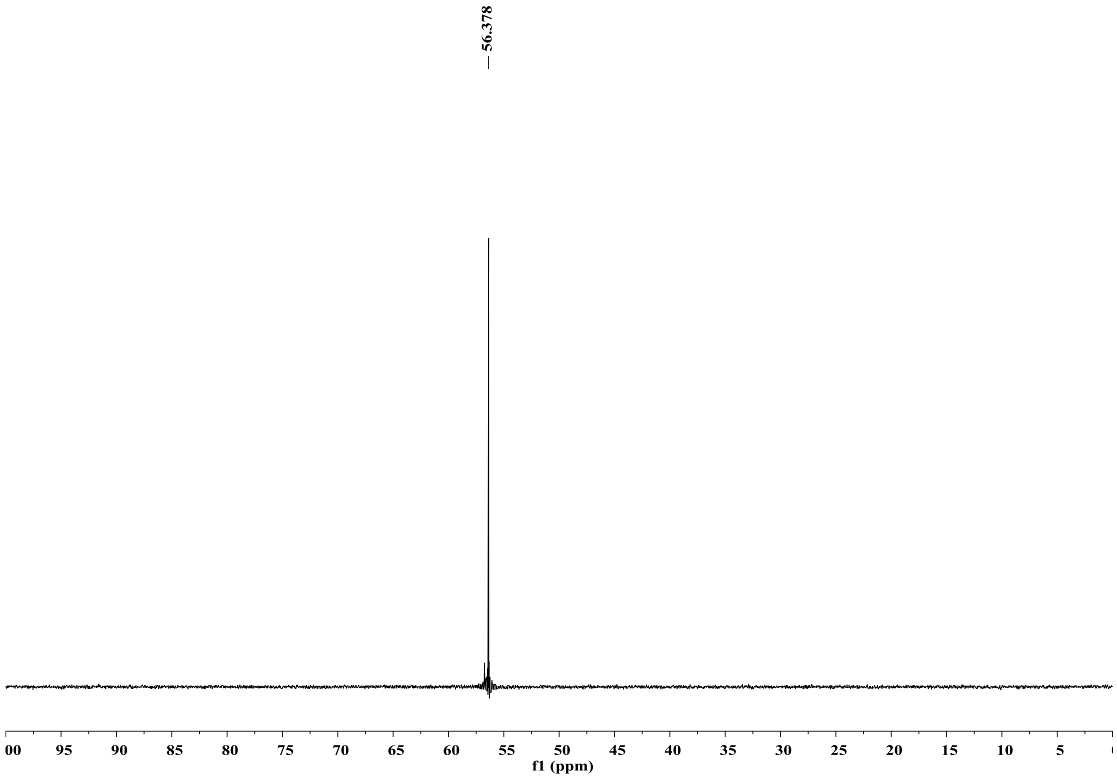

^{1}H NMR/^{13}C NMR/^{31}P NMR of product 5b

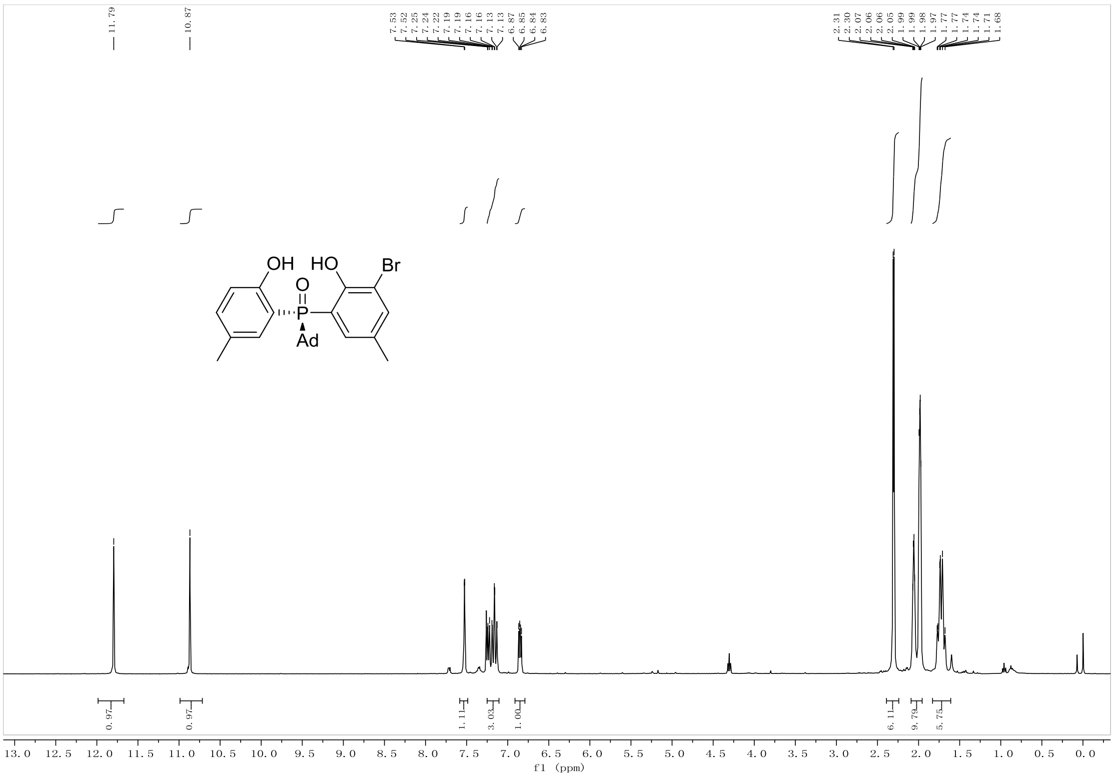
11.79
10.87
7.53 7.52 7.25 7.24 7.22 7.19 7.16 7.16 7.13 7.13 6.87 6.85 6.84 6.83
2.31 2.30 2.07 2.06 2.06 2.05 1.99 1.99 1.98 1.97 1.77 1.77 1.74 1.74 1.71 1.68
OH
HO
Br
O
P
Ad
0.97
0.97
1.11
3.03
1.00
6.11
9.79
5.75
f1 (ppm)

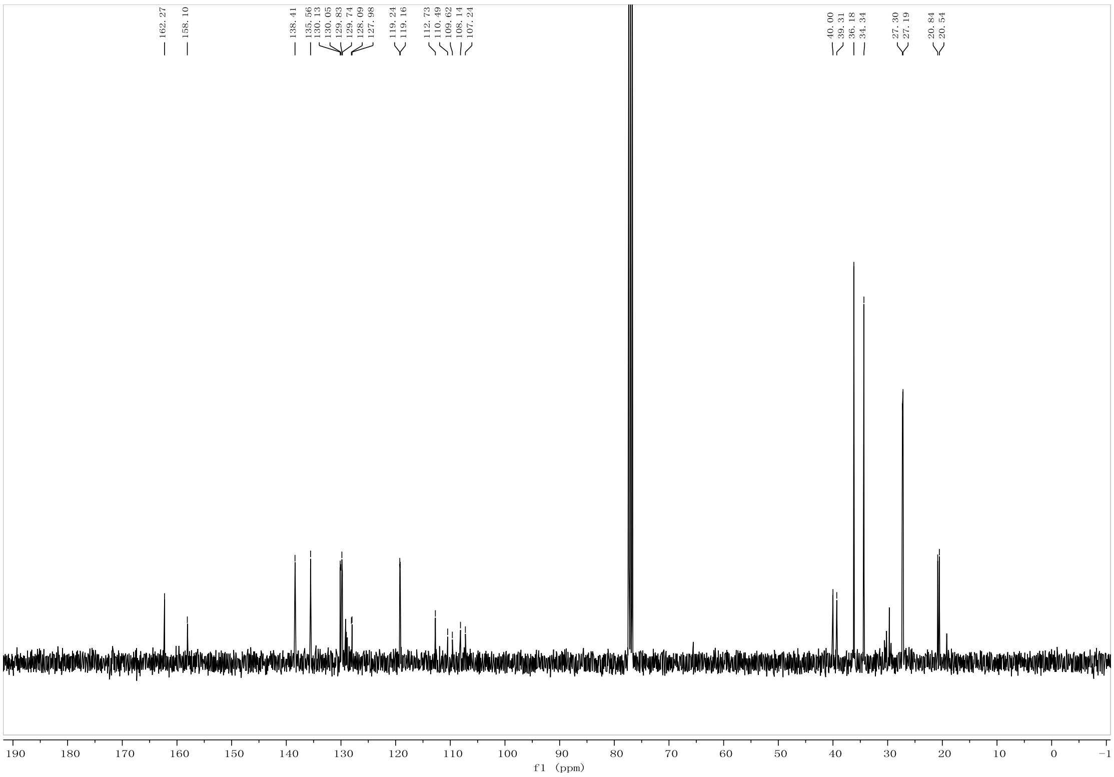
162.27
158.10
138.41 135.56 130.13 130.05 129.83 129.74 128.09 127.98
119.24 119.16
112.73 110.49 109.62 108.14 107.24
40.00 39.31 36.18 34.34
27.30 27.19
20.84 20.54
f1 (ppm)

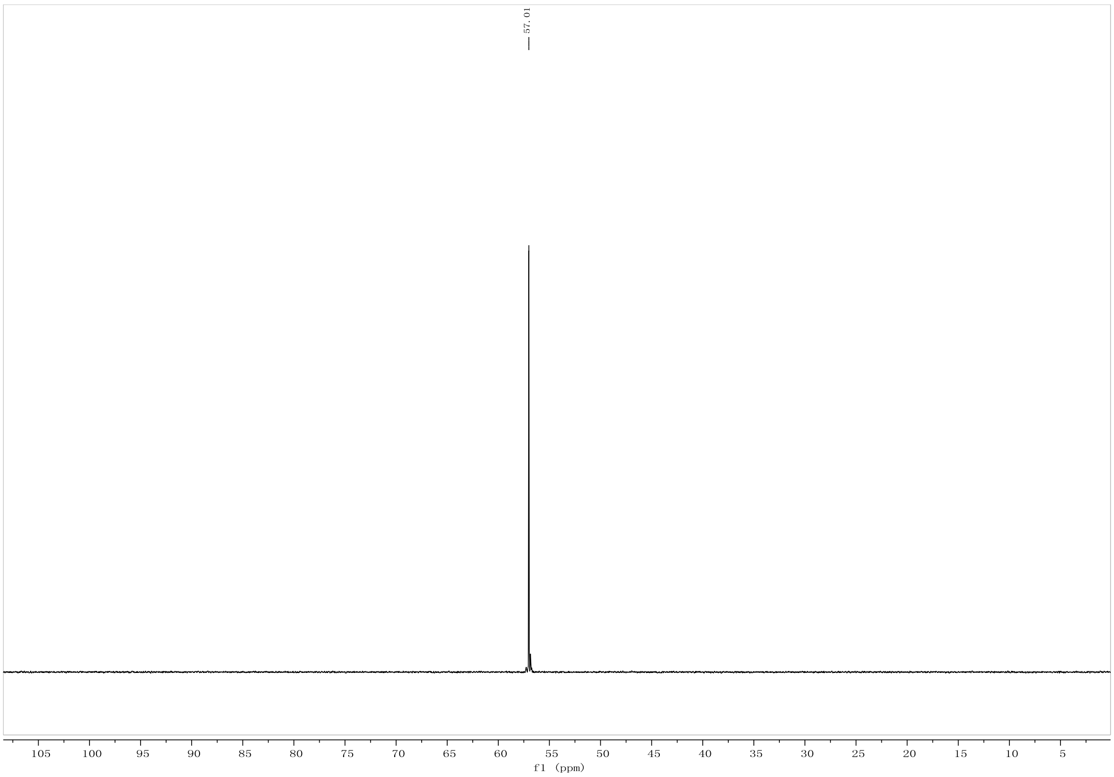

^{1}H NMR/^{13}C NMR/^{31}P NMR of product 7a

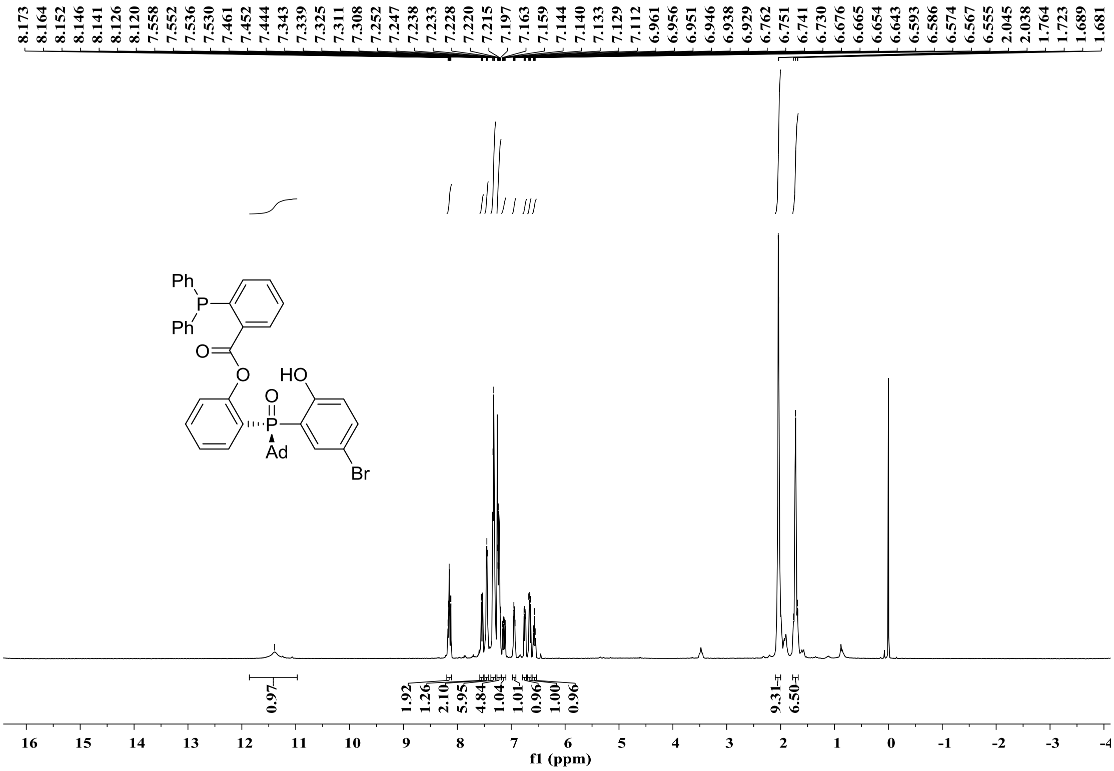

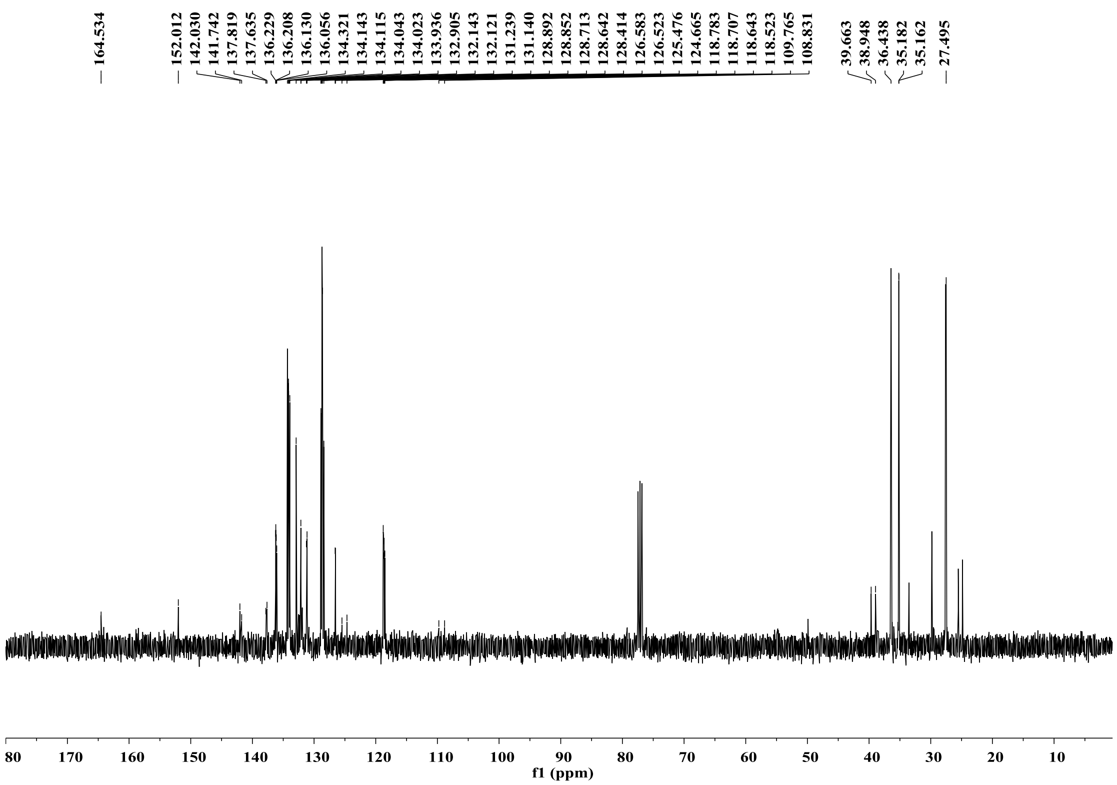

^{1}H NMR/^{13}C NMR/^{31}P NMR of product 7b

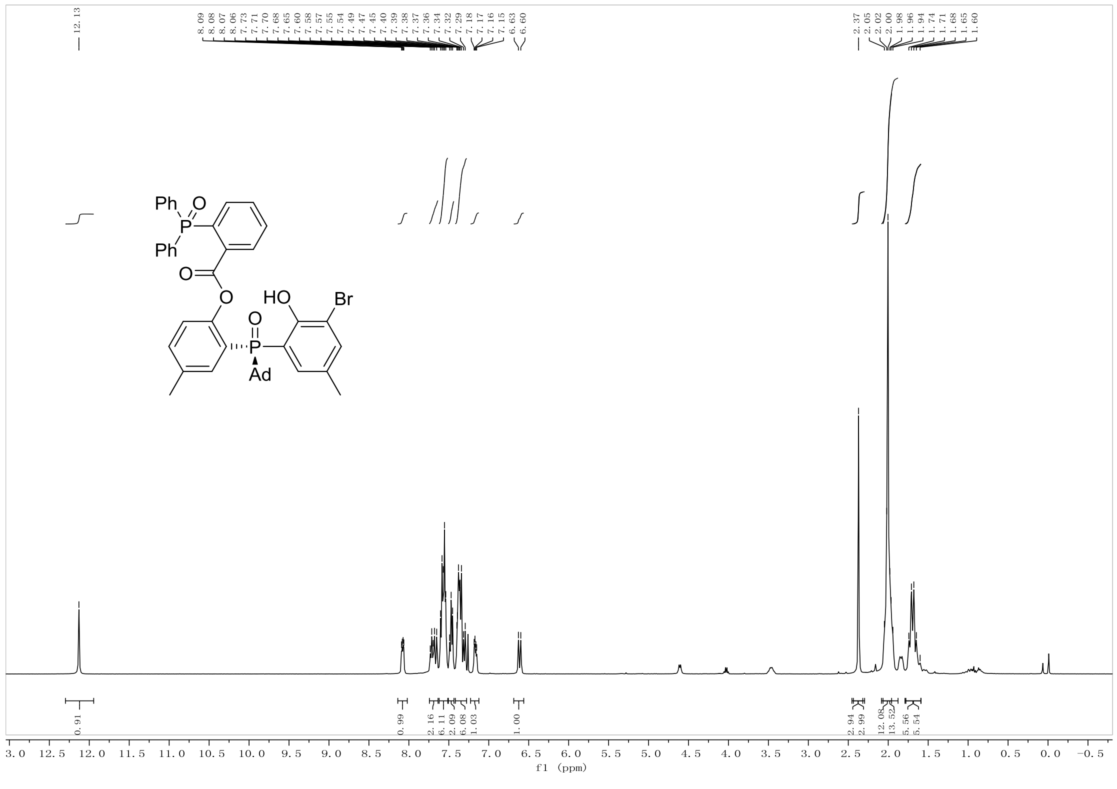

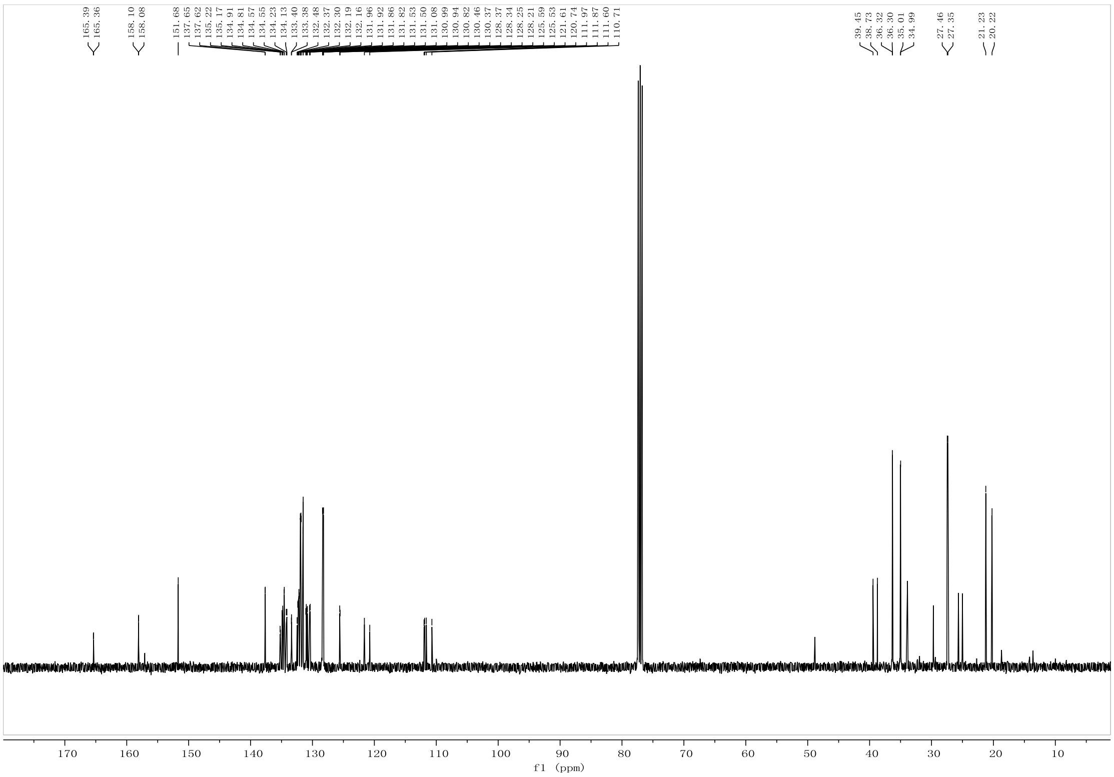

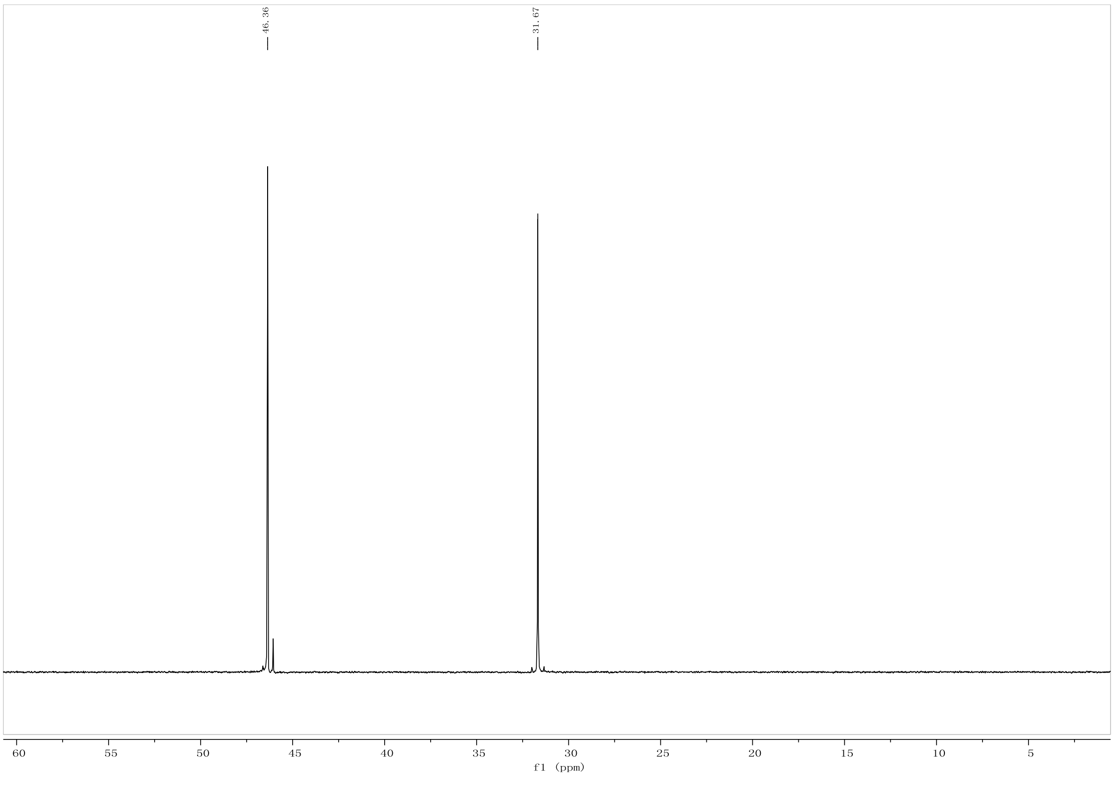

^{1}H NMR/^{13}C NMR/^{31}P NMR of product 12

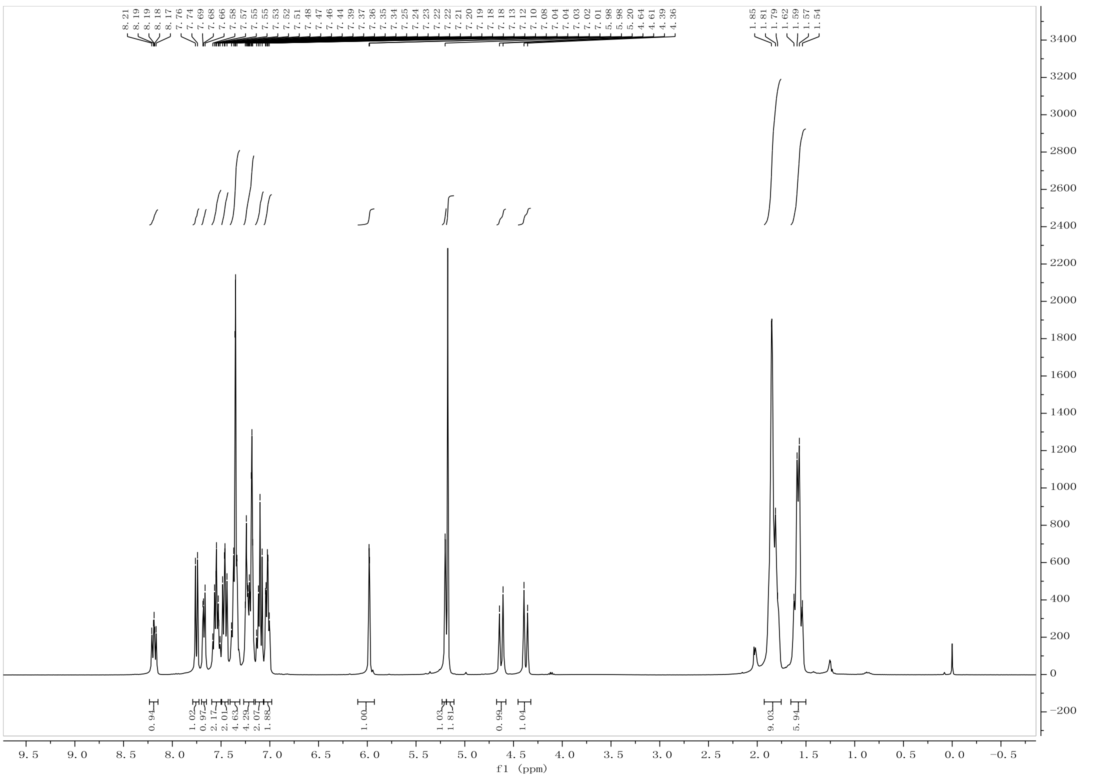

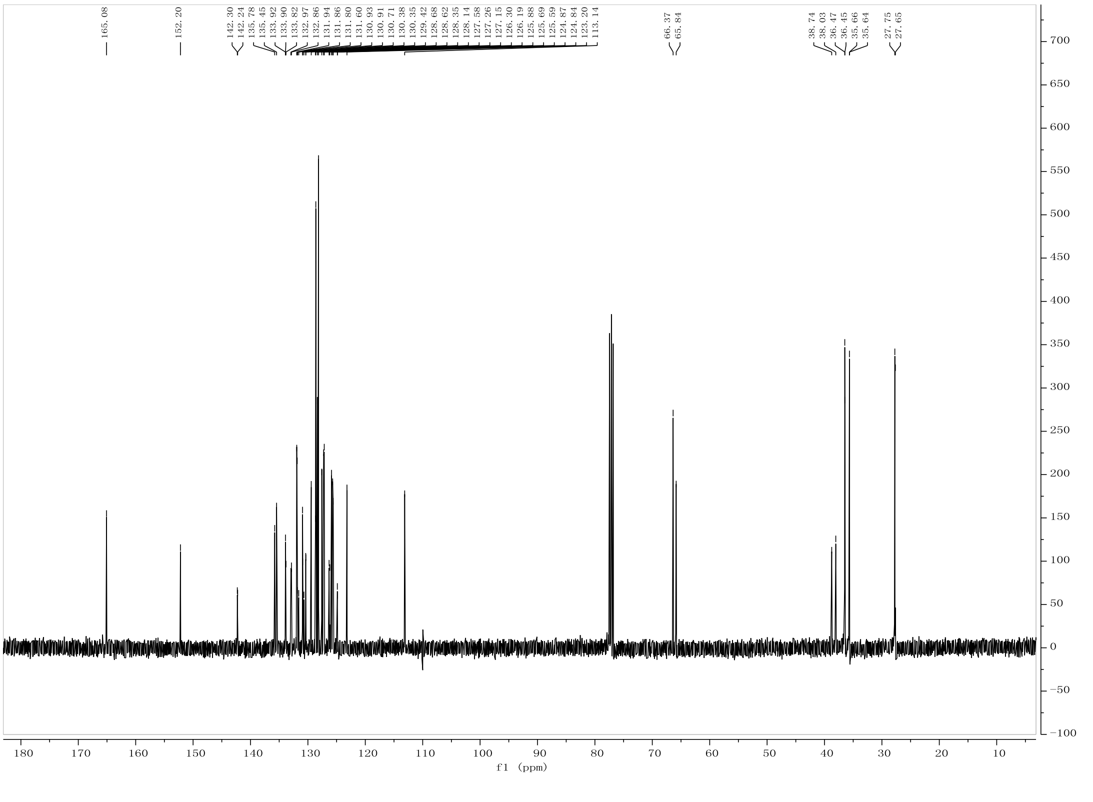

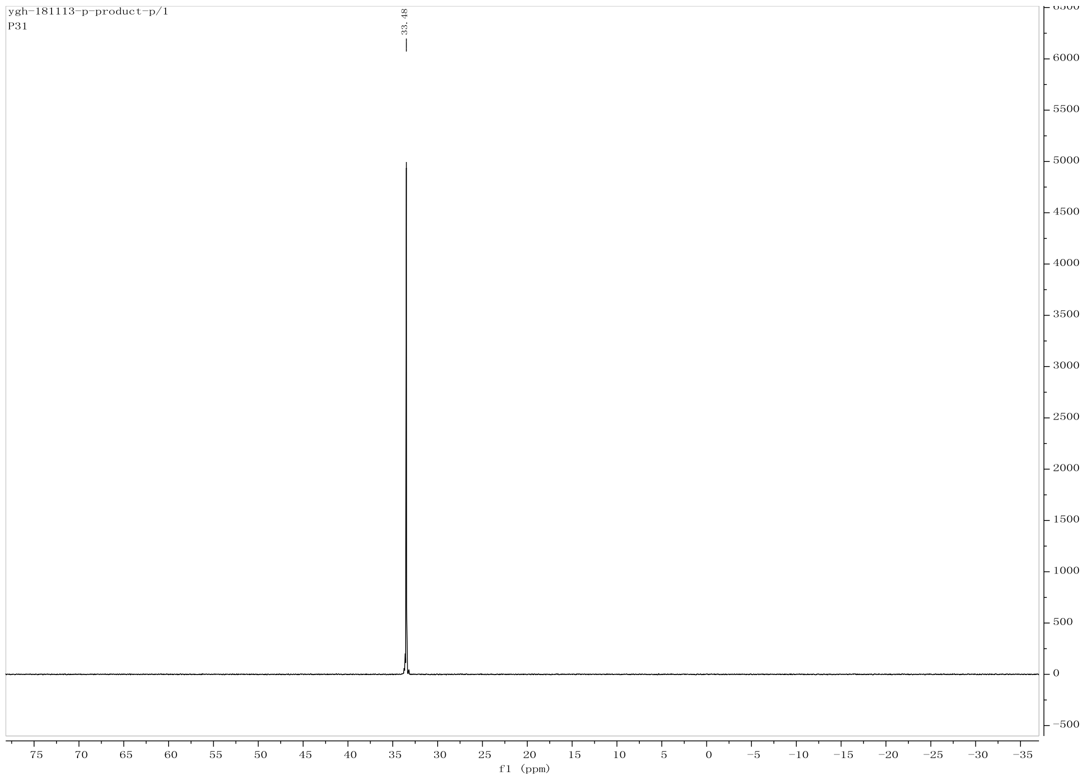

HPLC spectra

HPLC spectra of product 3e

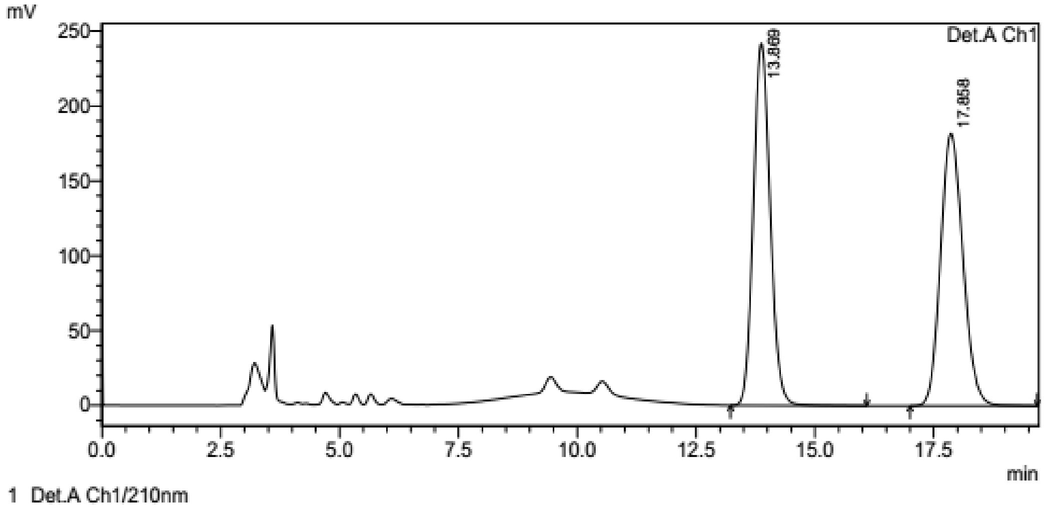

PeakTable

Detector A Ch1 210nm

Peak#	Ret. Time	Area	Height	Area %	Height %
1	13.869	5941443	241874	49.860	57.076
2	17.858	5974757	181903	50.140	42.924
Total		11916200	423777	100.000	100.000

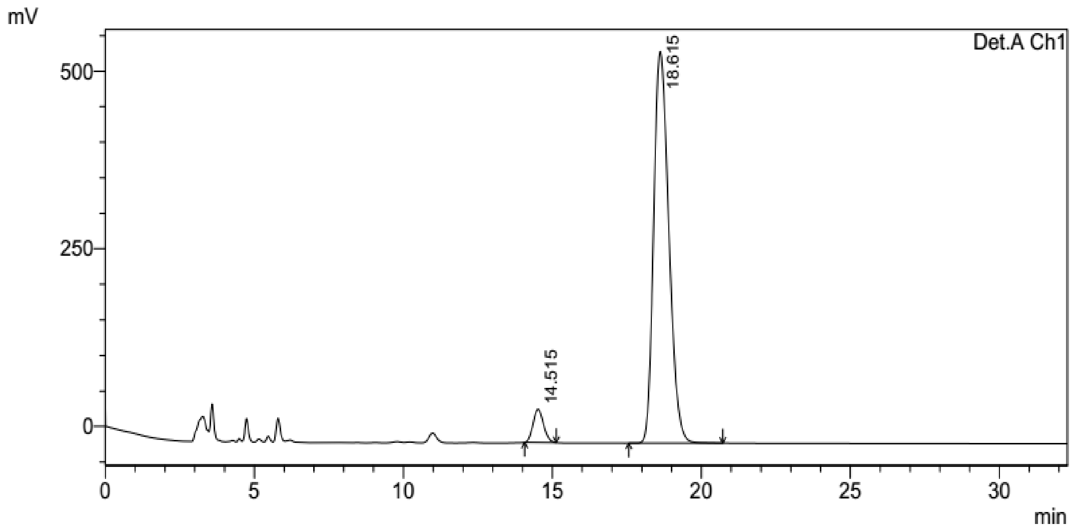

1 Det.A Ch1/210nm

PeakTable

Detector A Ch1 210nm

Peak#	Ret. Time	Area	Height	Area %	Height %
1	14.515	1148429	46518	5.565	7.778
2	18.615	19489146	551585	94.435	92.222
Total		20637575	598103	100.000	100.000

HPLC spectra of product 3f

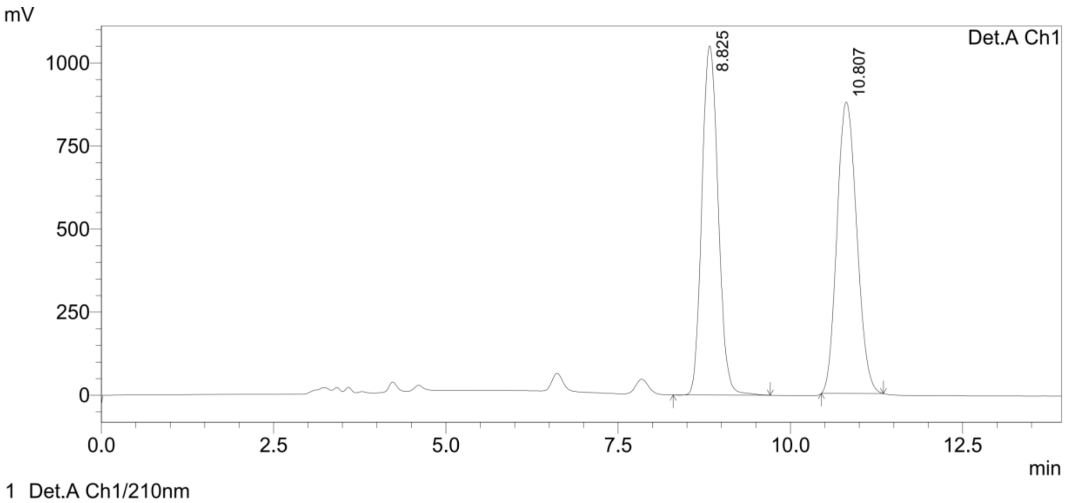

1 Det.A Ch1/210nm

PeakTable

Detector A Ch1 210nm

Peak#	Ret. Time	Area	Height	Area %	Height %
1	8.825	17305975	1050865	49.628	54.516
2	10.807	17565579	876766	50.372	45.484
Total		34871555	1927631	100.000	100.000

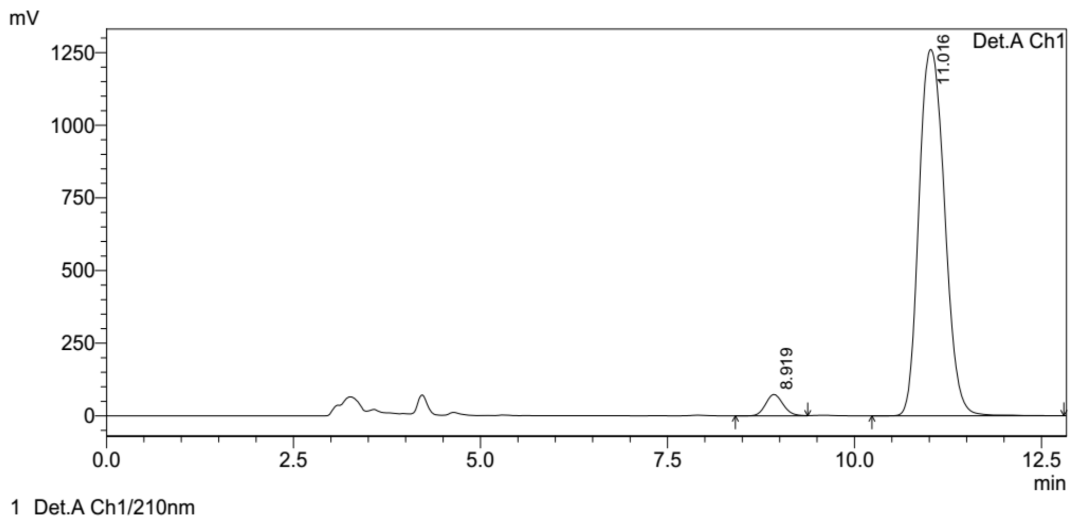

PeakTable

Detector A Ch1 210nm

Peak#	Ret. Time	Area	Height	Area %	Height %
1	8.919	1200650	73610	3.844	5.517
2	11.016	30036970	1260517	96.156	94.483
Total		31237620	1334127	100.000	100.000

HPLC spectra of product 3g

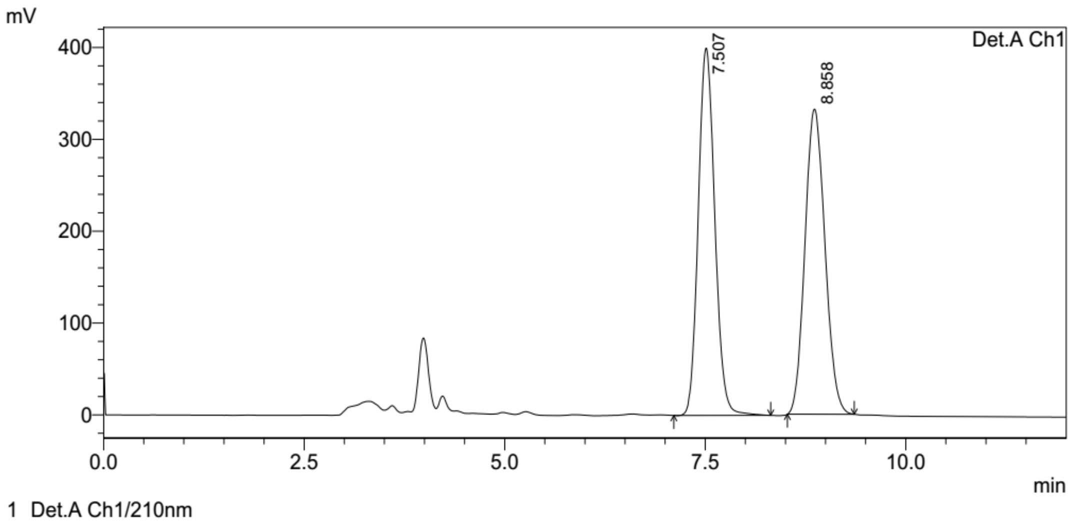

PeakTable

Detector A Ch1 210nm

Peak#	Ret. Time	Area	Height	Area %	Height %
1	7.507	5743022	399980	49.858	54.626
2	8.858	5775632	332234	50.142	45.374
Total		11518654	732214	100.000	100.000

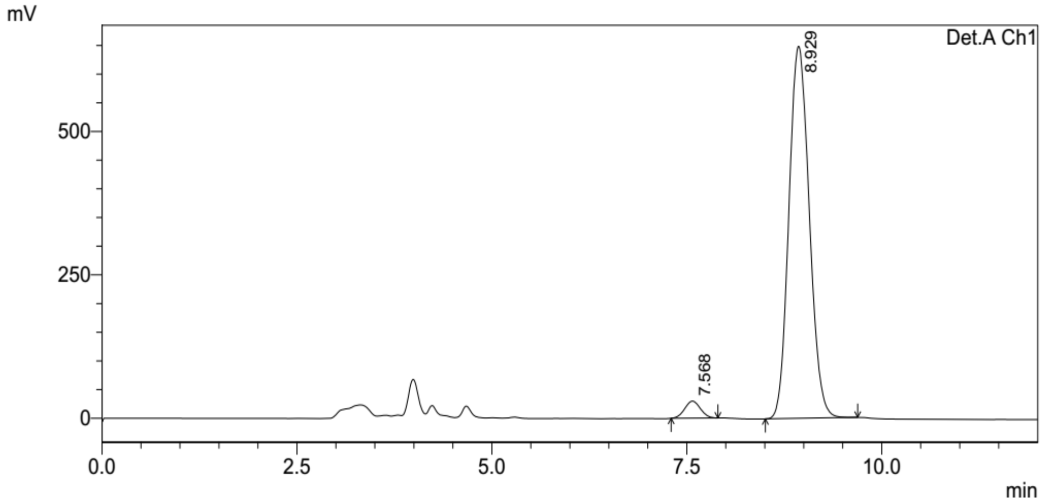

1 Det.A Ch1/210nm

PeakTable

Detector A Ch1 210nm

Peak#	Ret. Time	Area	Height	Area %	Height %
1	7.568	415418	29656	3.398	4.371
2	8.929	11810872	648786	96.602	95.629
Total		12226290	678443	100.000	100.000

HPLC spectra of product 3h

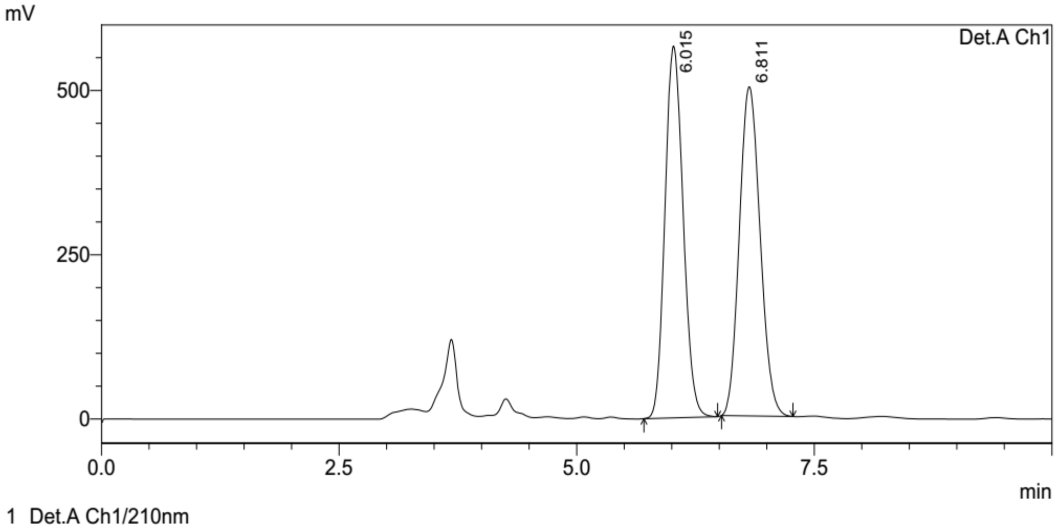

1 Det.A Ch1/210nm

PeakTable

Detector A Ch1 210nm

Peak#	Ret. Time	Area	Height	Area %	Height %
1	6.015	7533561	565954	49.698	53.043
2	6.811	7625256	501015	50.302	46.957
Total		15158817	1066969	100.000	100.000

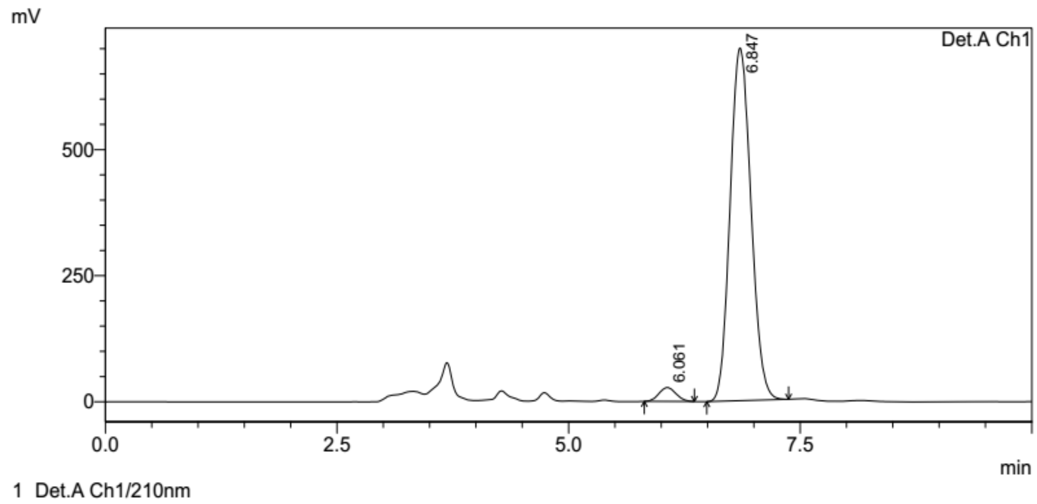

PeakTable

Detector A Ch1 210nm

Peak#	Ret. Time	Area	Height	Area %	Height %
1	6.061	353997	27550	3.124	3.789
2	6.847	10978469	699581	96.876	96.211
Total		11332466	727131	100.000	100.000

HPLC spectra of product 3i

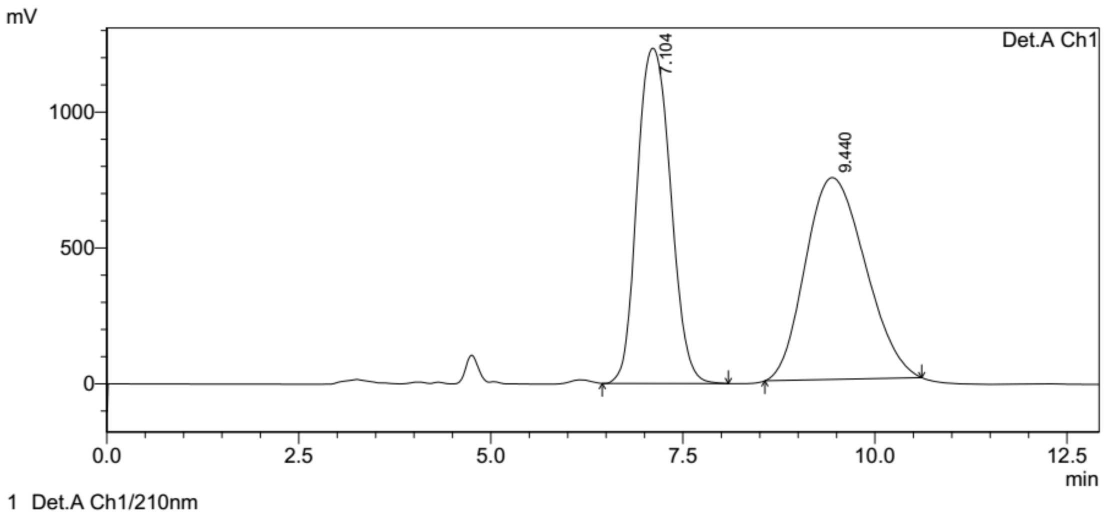

PeakTable

Detector A Ch1 210nm

Peak#	Ret. Time	Area	Height	Area %	Height %
1	7.104	37427107	1233500	48.163	62.422
2	9.440	40281402	742572	51.837	37.578
Total		77708509	1976073	100.000	100.000

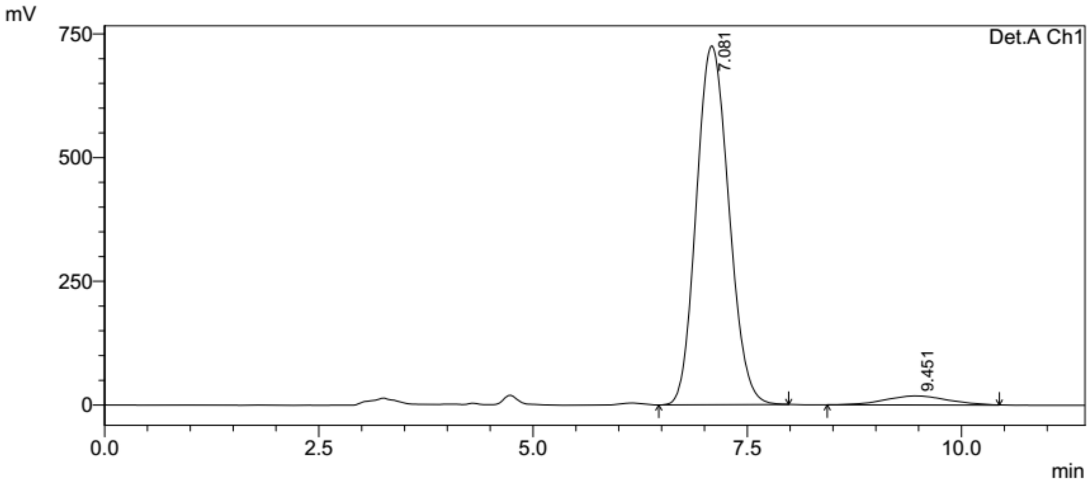

1 Det.A Ch1/210nm

PeakTable

Detector A Ch1 210nm

Peak#	Ret. Time	Area	Height	Area %	Height %
1	7.081	19131846	725295	95.334	97.541
2	9.451	936377	18281	4.666	2.459
Total		20068223	743576	100.000	100.000

HPLC spectra of product 3j

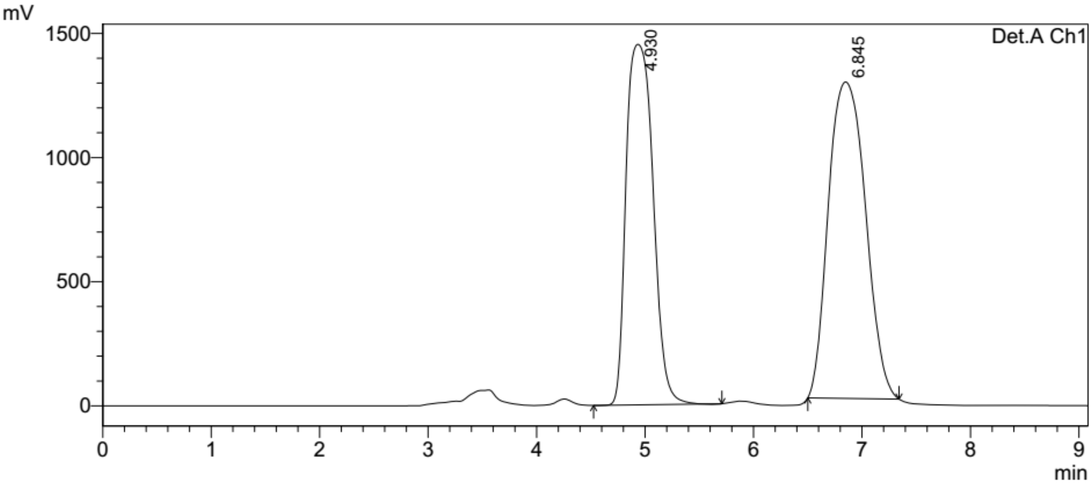

1 Det.A Ch1/210nm

PeakTable

Detector A Ch1 210nm

Peak#	Ret. Time	Area	Height	Area %	Height %
1	4.930	25102885	1452162	45.230	53.265
2	6.845	30398049	1274135	54.770	46.735
Total		55500934	2726298	100.000	100.000

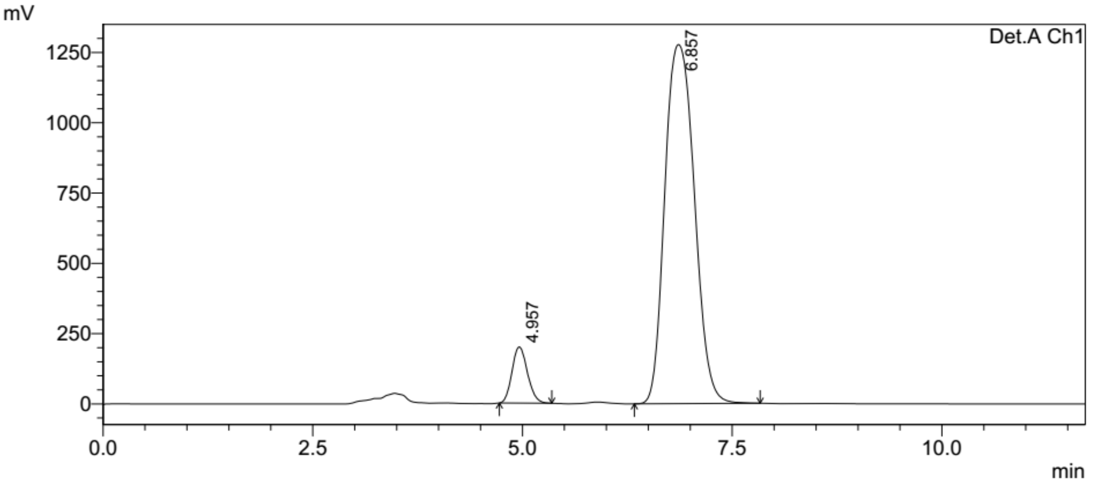

PeakTable

Detector A Ch1 210nm

Peak#	Ret. Time	Area	Height	Area %	Height %
1	4.957	2564047	199998	7.572	13.538
2	6.857	31298370	1277291	92.428	86.462
Total		33862417	1477289	100.000	100.000

HPLC spectra of product 3k

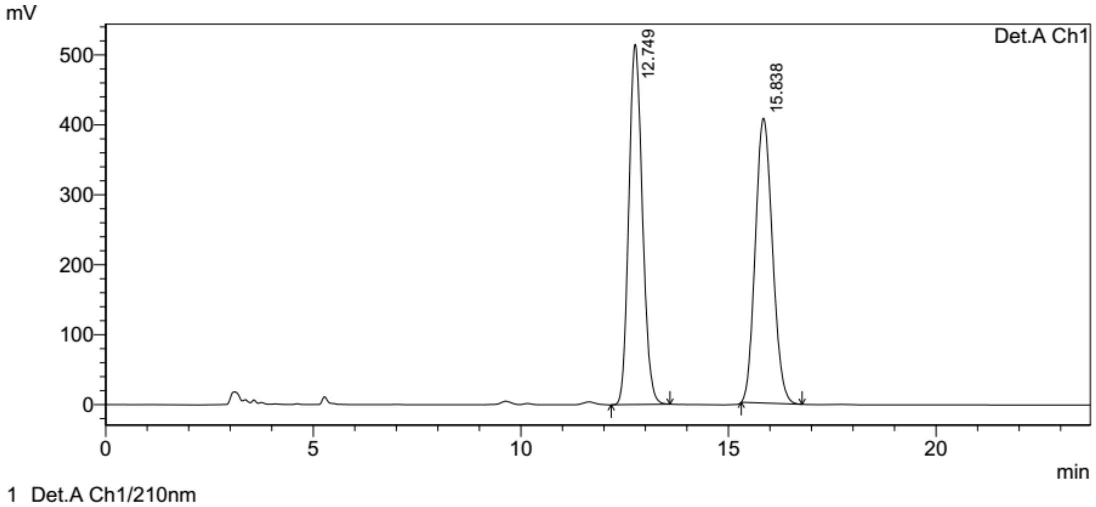

PeakTable

Detector A Ch1 210nm

Peak#	Ret. Time	Area	Height	Area %	Height %
1	12.749	11722986	515091	50.060	55.846
2	15.838	11694948	407247	49.940	44.154
Total		23417935	922338	100.000	100.000

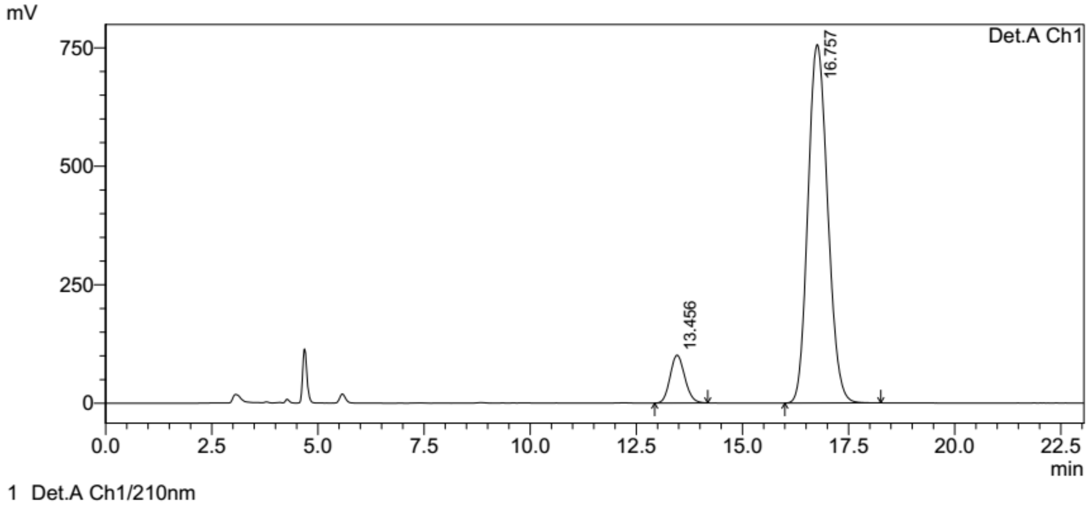

PeakTable

Detector A Ch1 210nm

Peak#	Ret. Time	Area	Height	Area %	Height %
1	13.456	2434729	101126	9.048	11.792
2	16.757	24472884	756452	90.952	88.208
Total		26907613	857577	100.000	100.000

HPLC spectra of product 3l

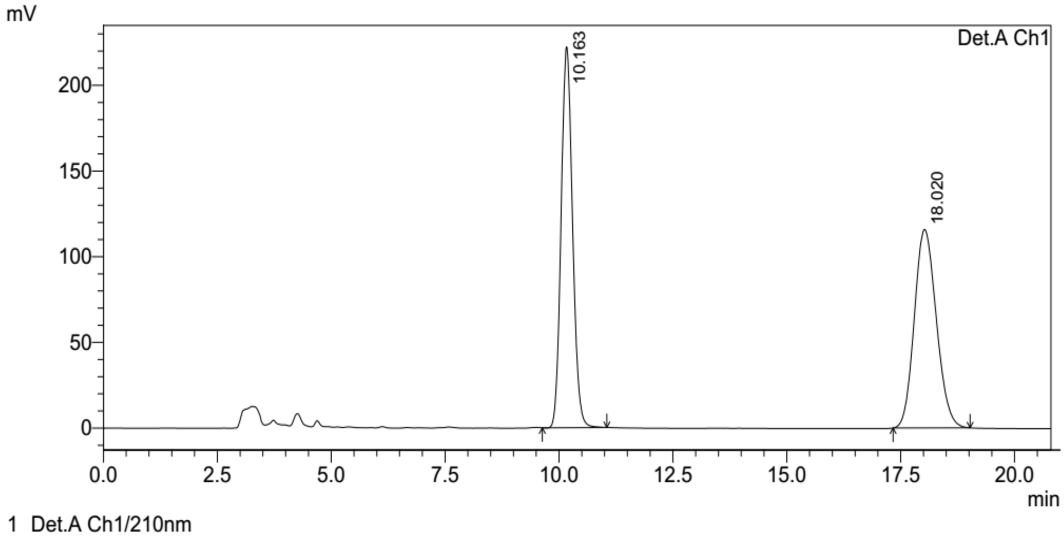

PeakTable

Detector A Ch1 210nm

Peak#	Ret. Time	Area	Height	Area %	Height %
1	10.163	3893371	222255	49.972	65.743
2	18.020	3897768	115812	50.028	34.257
Total		7791139	338067	100.000	100.000

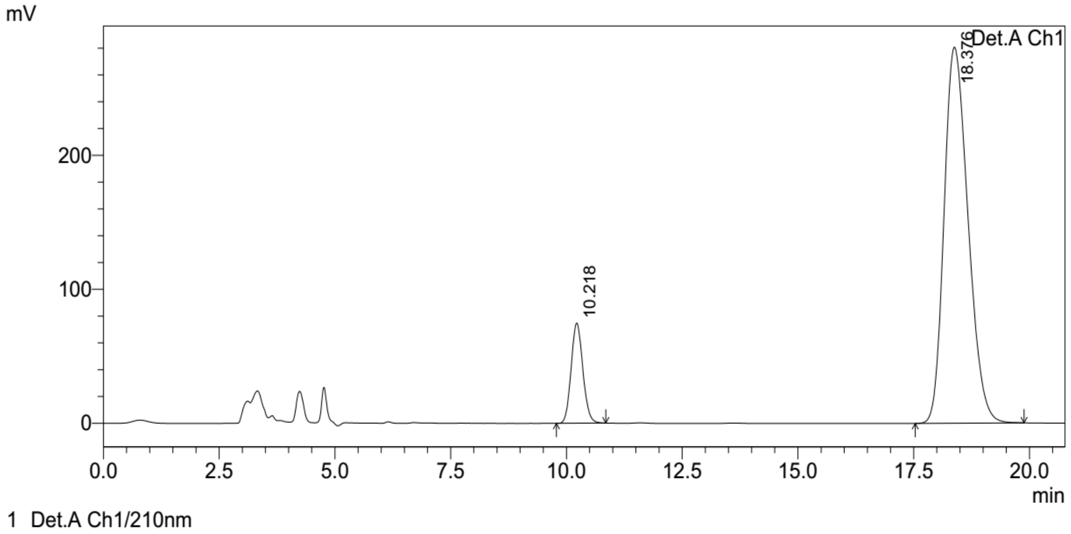

PeakTable

Detector A Ch1 210nm

Peak#	Ret. Time	Area	Height	Area %	Height %
1	10.218	1315795	74778	11.540	21.035
2	18.376	10086211	280708	88.460	78.965
Total		11402006	355486	100.000	100.000

HPLC spectra of product 3m

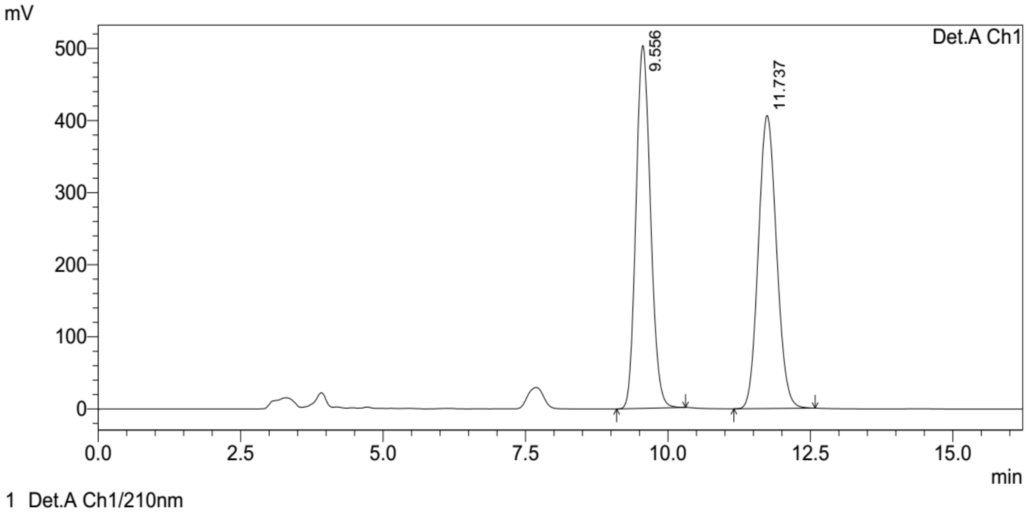

PeakTable

Detector A Ch1 210nm

Peak#	Ret. Time	Area	Height	Area %	Height %
1	9.556	8934832	503415	49.740	55.318
2	11.737	9028197	406623	50.260	44.682
Total		17963029	910038	100.000	100.000

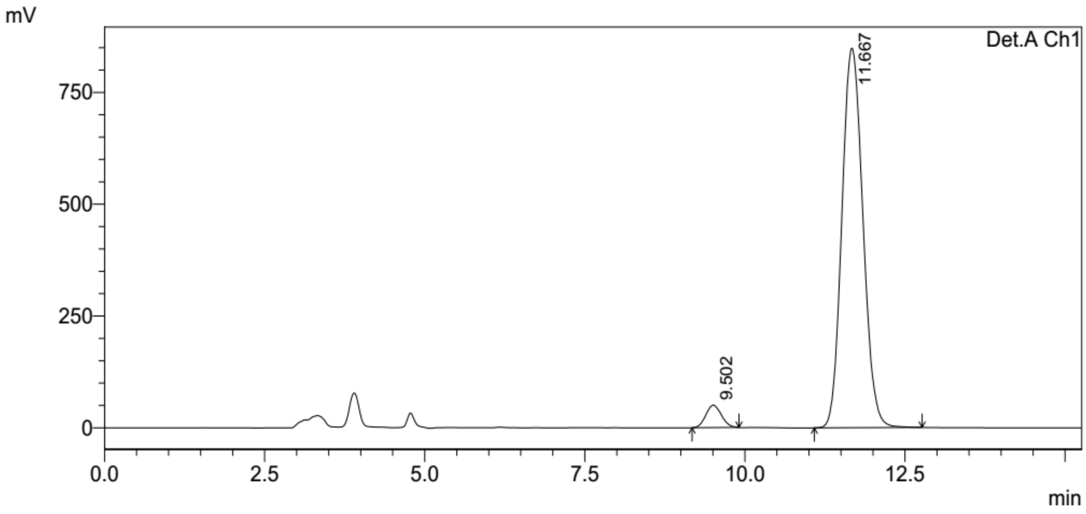

1 Det.A Ch1/210nm

PeakTable

Detector A Ch1 210nm

Peak#	Ret. Time	Area	Height	Area %	Height %
1	9.502	851882	50405	4.212	5.608
2	11.667	19373278	848366	95.788	94.392
Total		20225160	898771	100.000	100.000

HPLC spectra of product 3n

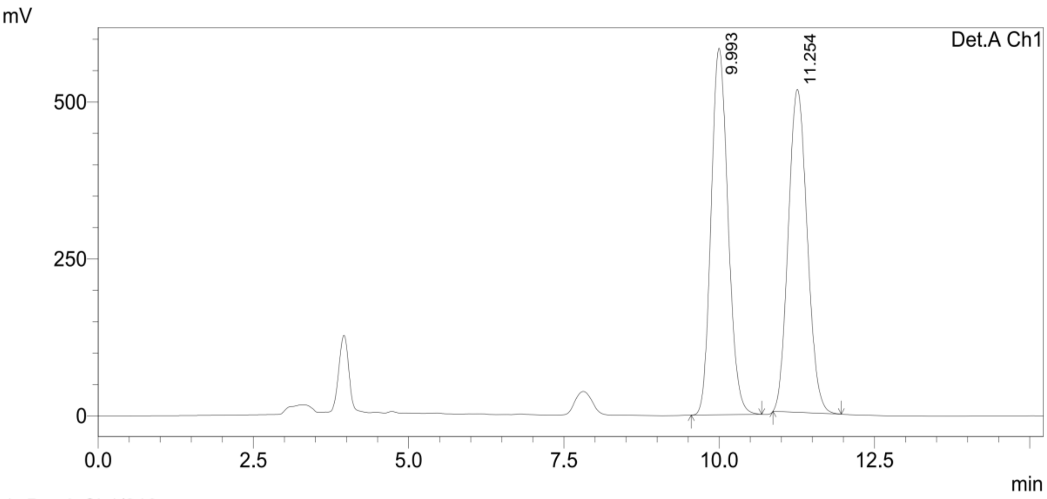

1 Det.A Ch1/210nm

PeakTable

Detector A Ch1 210nm

Peak#	Ret. Time	Area	Height	Area %	Height %
1	9.993	11085953	584125	50.317	53.173
2	11.254	10946236	514403	49.683	46.827
Total		22032189	1098528	100.000	100.000

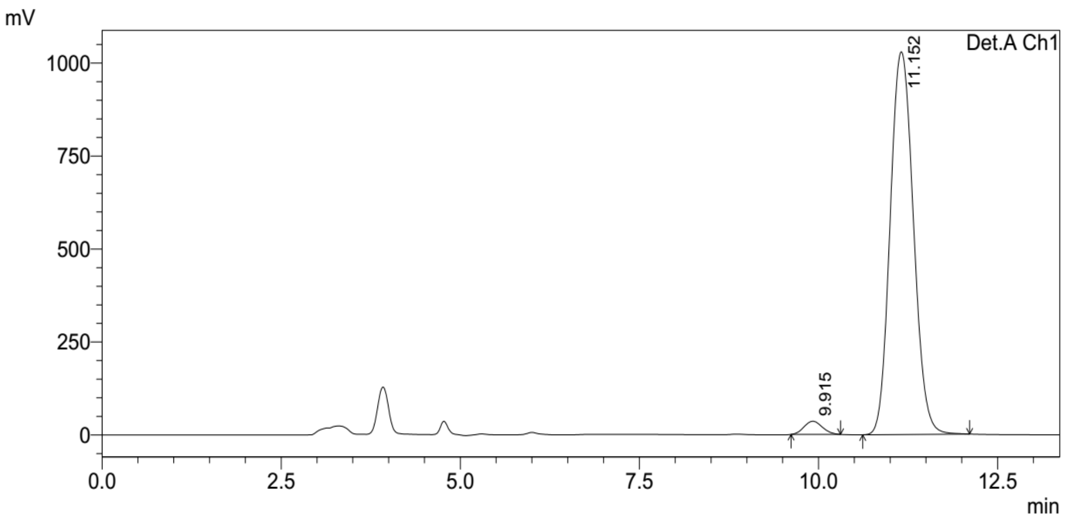

1 Det.A Ch1/210nm

PeakTable

Detector A Ch1 210nm

Peak#	Ret. Time	Area	Height	Area %	Height %
1	9.915	631074	35588	2.645	3.342
2	11.152	23231338	1029397	97.355	96.658
Total		23862412	1064985	100.000	100.000

HPLC spectra of product 3o

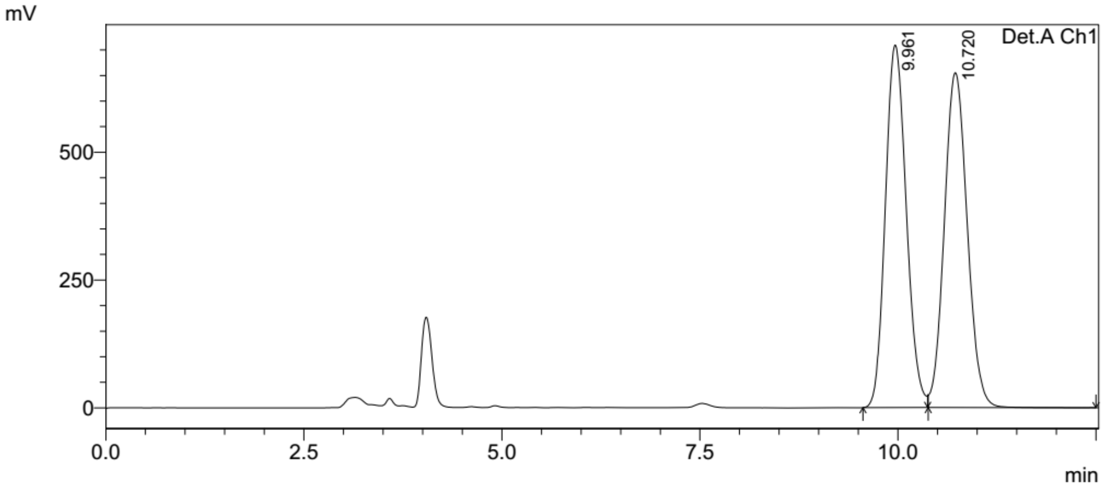

1 Det.A Ch1/210nm

PeakTable

Detector A Ch1 210nm

Peak#	Ret. Time	Area	Height	Area %	Height %
1	9.961	13031912	709134	49.768	51.982
2	10.720	13153673	655055	50.232	48.018
Total		26185585	1364189	100.000	100.000

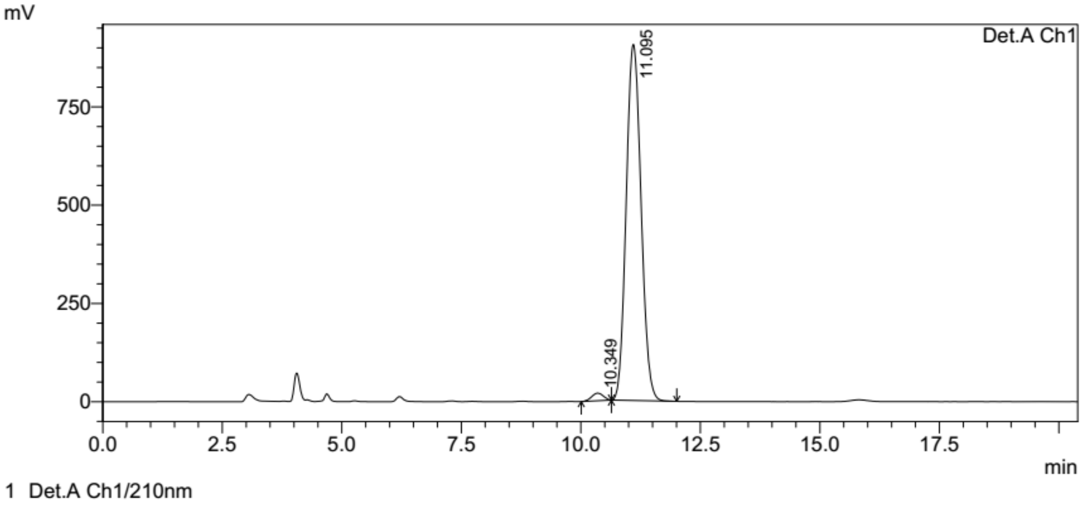

PeakTable

Detector A Ch1 210nm

Peak#	Ret. Time	Area	Height	Area %	Height %
1	10.349	324603	19338	1.631	2.090
2	11.095	19580920	905756	98.369	97.910
Total		19905523	925095	100.000	100.000

HPLC spectra of product 3p

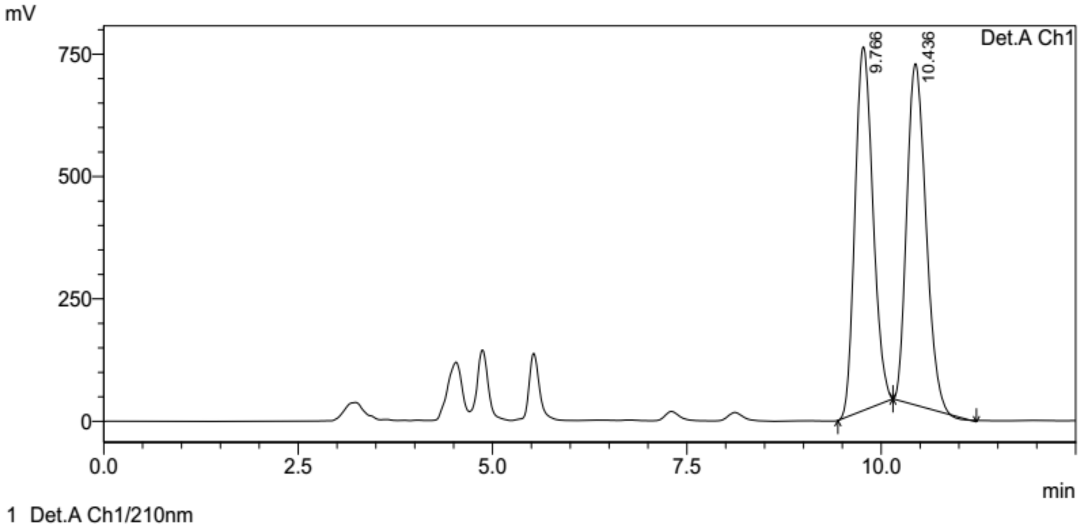

PeakTable

Detector A Ch1 210nm

Peak#	Ret. Time	Area	Height	Area %	Height %
1	9.766	11897780	743291	49.959	51.596
2	10.436	11917357	697307	50.041	48.404
Total		23815137	1440598	100.000	100.000

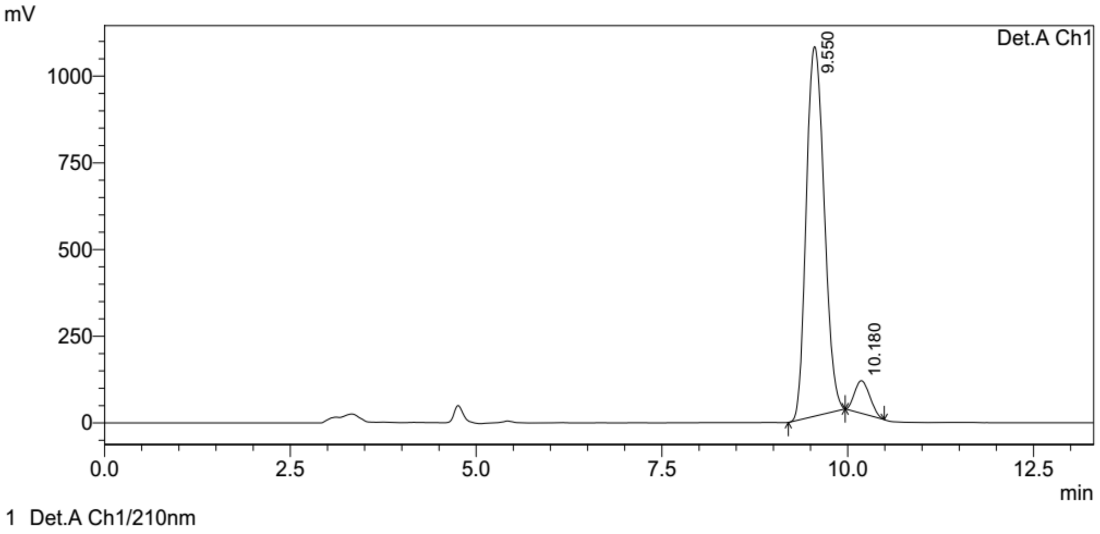

PeakTable

Detector A Ch1 210nm

Peak#	Ret. Time	Area	Height	Area %	Height %
1	9.550	17928717	1065508	93.025	91.891
2	10.180	1344394	94026	6.975	8.109
Total		19273111	1159534	100.000	100.000

HPLC spectra of product 3q

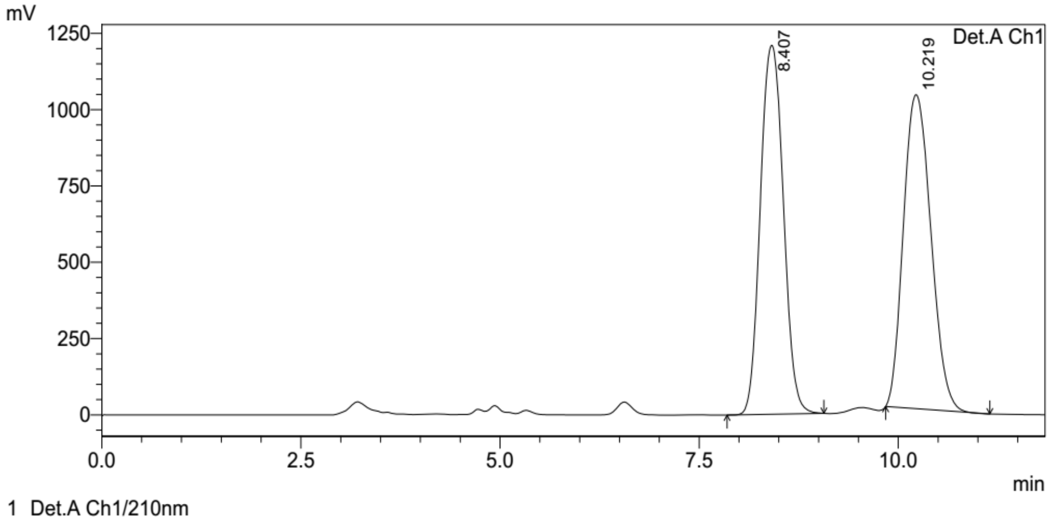

PeakTable

Detector A Ch1 210nm

Peak#	Ret. Time	Area	Height	Area %	Height %
1	8.407	24339184	1208909	49.627	54.031
2	10.219	24705424	1028507	50.373	45.969
Total		49044608	2237416	100.000	100.000

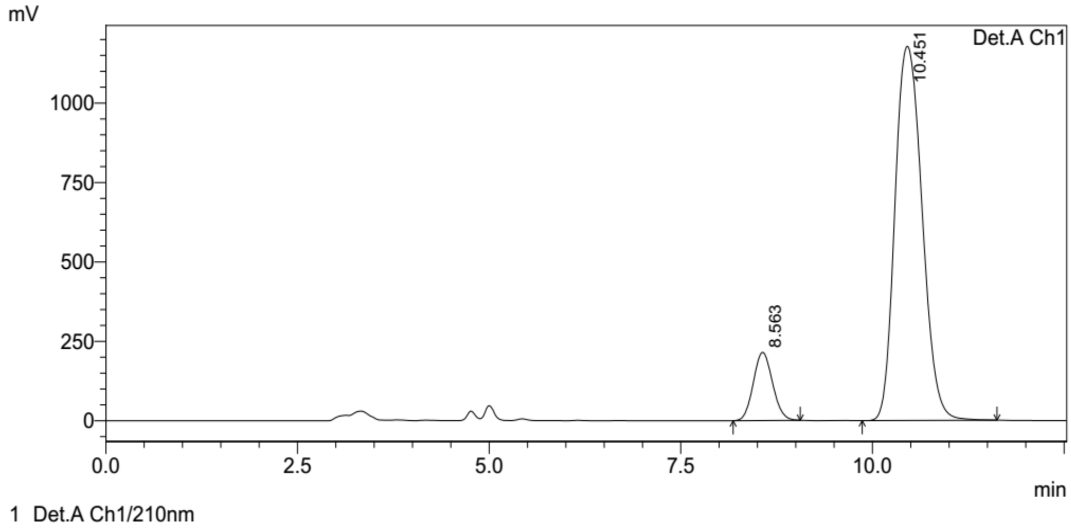

PeakTable

Detector A Ch1 210nm

Peak#	Ret. Time	Area	Height	Area %	Height %
1	8.563	3752436	214746	11.448	15.421
2	10.451	29026743	1177796	88.552	84.579
Total		32779180	1392542	100.000	100.000

HPLC spectra of product 3r

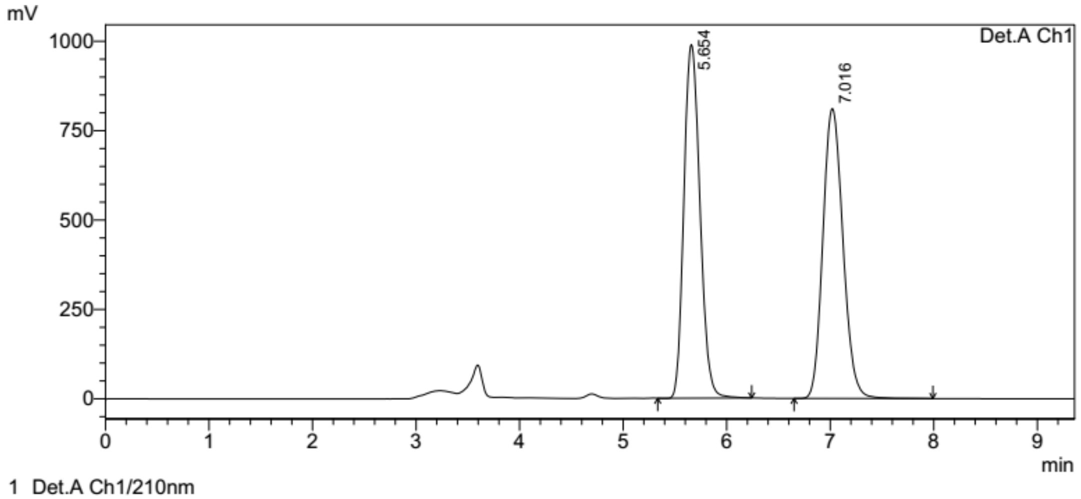

PeakTable

Detector A Ch1 210nm

Peak#	Ret. Time	Area	Height	Area %	Height %
1	5.654	10665687	988819	49.394	54.949
2	7.016	10927570	810718	50.606	45.051
Total		21593257	1799538	100.000	100.000

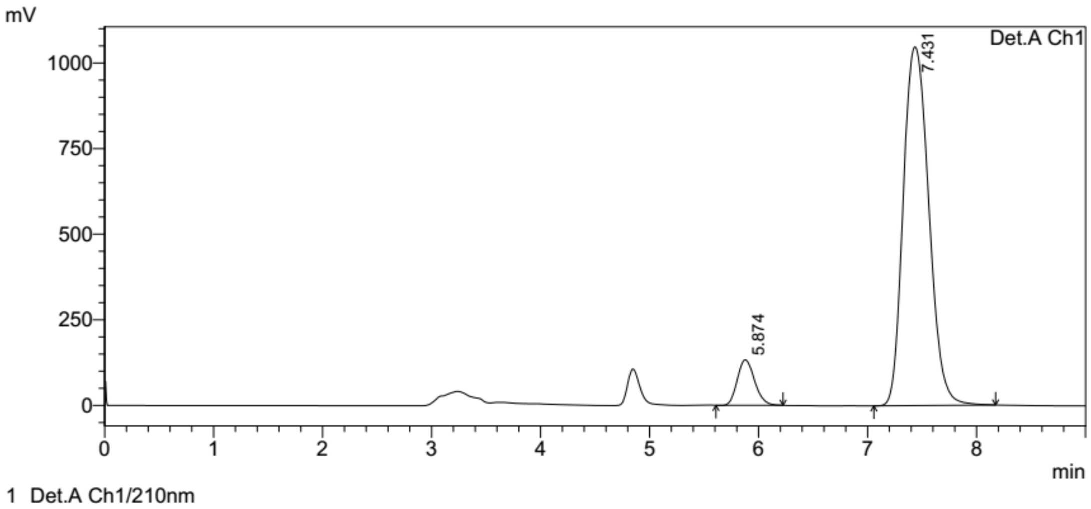

PeakTable

Detector A Ch1 210nm

Peak#	Ret. Time	Area	Height	Area %	Height %
1	5.874	1466055	132773	7.895	11.251
2	7.431	17104220	1047346	92.105	88.749
Total		18570275	1180119	100.000	100.000

HPLC spectra of product 3s

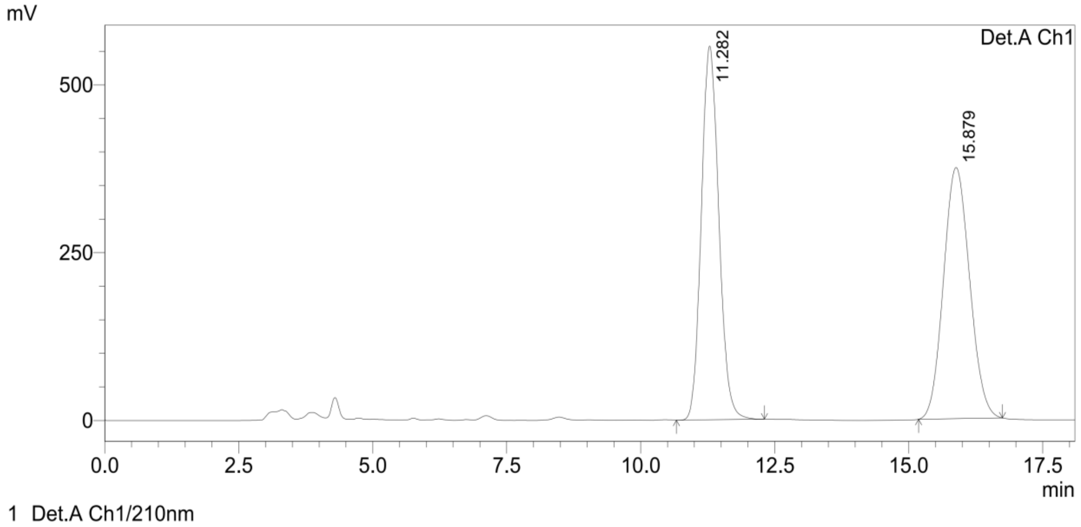

PeakTable

Detector A Ch1 210nm

Peak#	Ret. Time	Area	Height	Area %	Height %
1	11.282	12684804	556730	49.963	59.794
2	15.879	12703527	374356	50.037	40.206
Total		25388331	931086	100.000	100.000

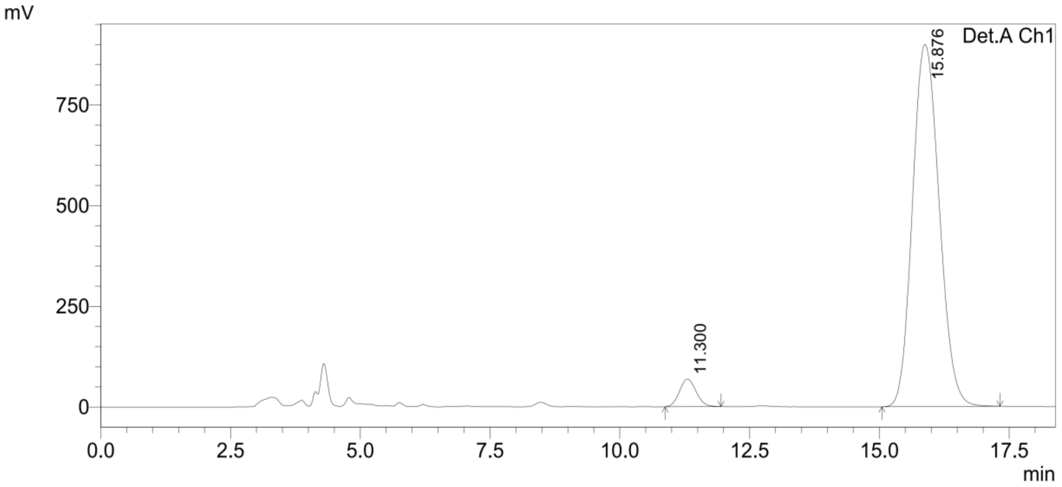

1 Det.A Ch1/210nm

PeakTable

Detector A Ch1 210nm

Peak#	Ret. Time	Area	Height	Area %	Height %
1	11.300	1527129	68735	4.518	7.097
2	15.876	32270281	899706	95.482	92.903
Total		33797410	968441	100.000	100.000

HPLC spectra of product 3t

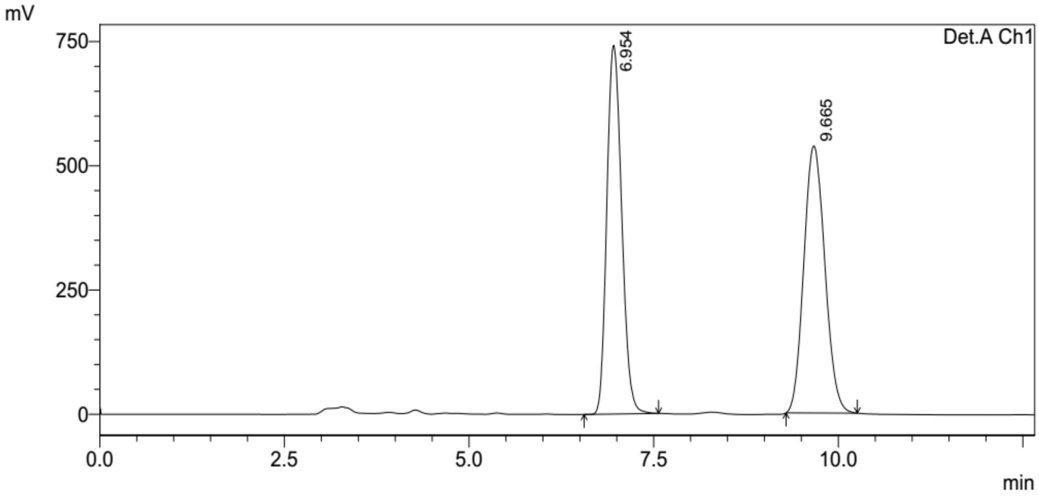

1 Det.A Ch1/210nm

PeakTable

Detector A Ch1 210nm

Peak#	Ret. Time	Area	Height	Area %	Height %
1	6.954	10371411	742098	49.263	57.989
2	9.665	10681831	537632	50.737	42.011
Total		21053241	1279731	100.000	100.000

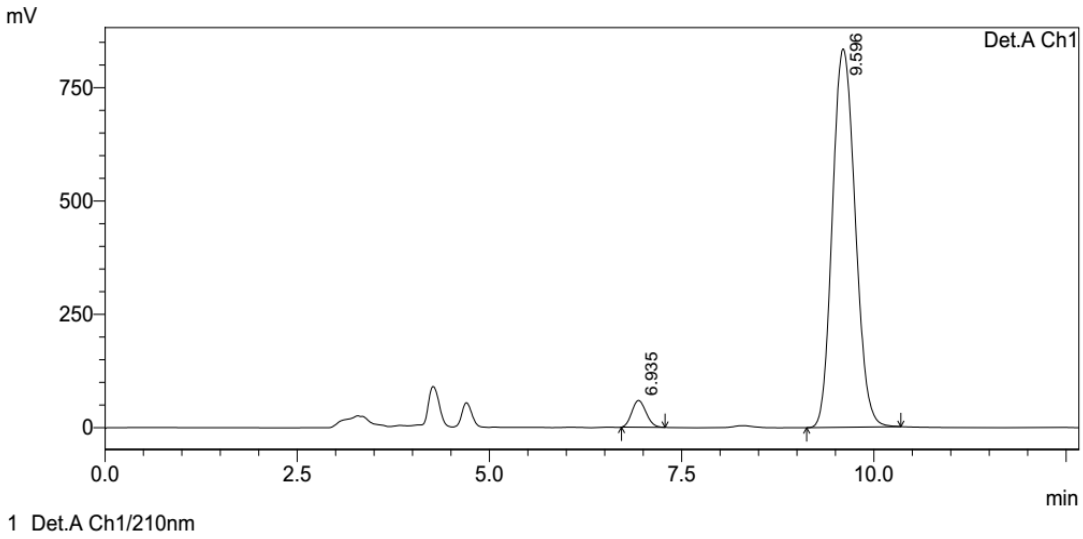

PeakTable

Detector A Ch1 210nm

Peak#	Ret. Time	Area	Height	Area %	Height %
1	6.935	761360	59083	4.240	6.609
2	9.596	17194415	834954	95.760	93.391
Total		17955776	894036	100.000	100.000

HPLC spectra of product 3u

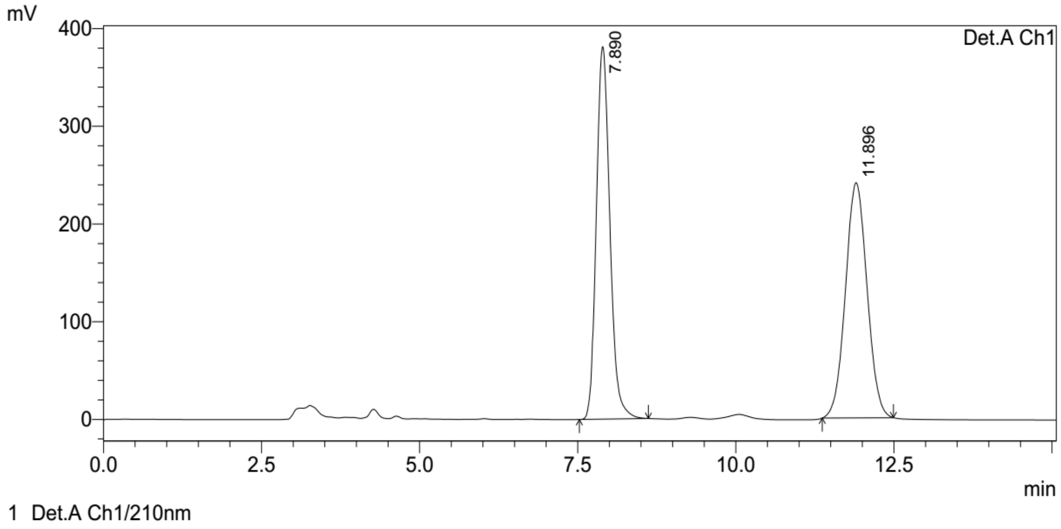

PeakTable

Detector A Ch1 210nm

Peak#	Ret. Time	Area	Height	Area %	Height %
1	7.890	5678731	381371	49.799	61.298
2	11.896	5724571	240784	50.201	38.702
Total		11403302	622156	100.000	100.000

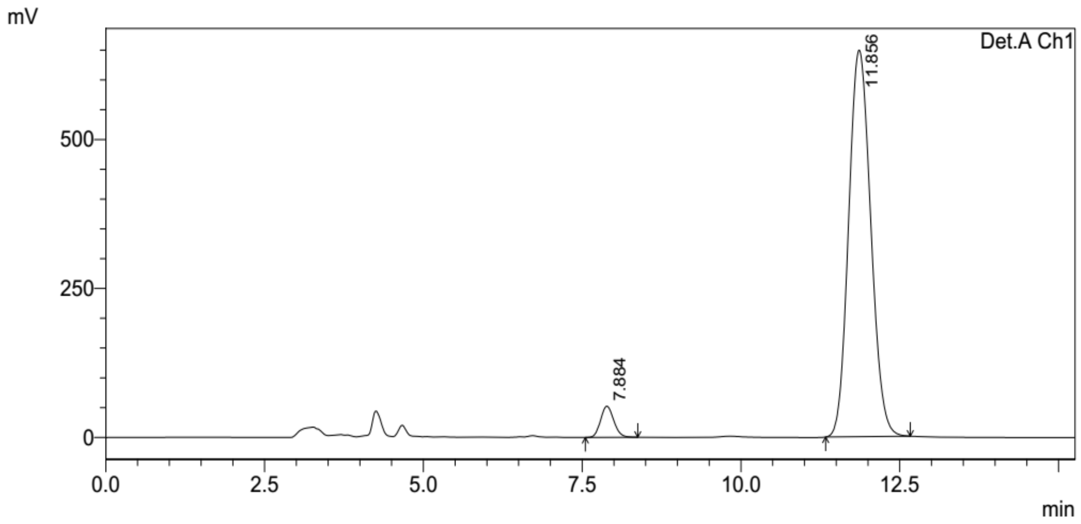

1 Det.A Ch1/210nm

PeakTable

Detector A Ch1 210nm

Peak#	Ret. Time	Area	Height	Area %	Height %
1	7.884	753341	52243	4.580	7.451
2	11.856	15694148	648912	95.420	92.549
Total		16447489	701155	100.000	100.000

HPLC spectra of product 3v

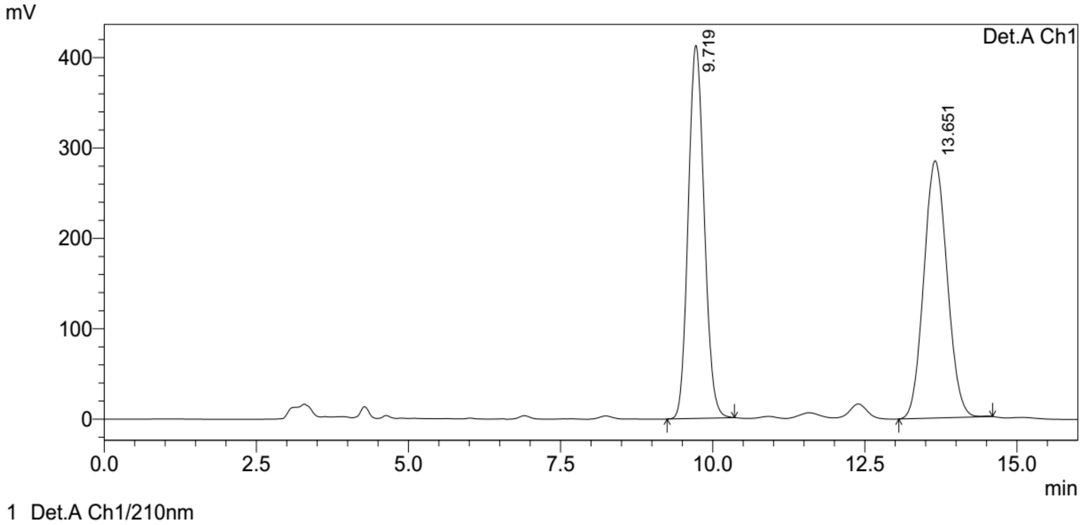

1 Det.A Ch1/210nm

PeakTable

Detector A Ch1 210nm

Peak#	Ret. Time	Area	Height	Area %	Height %
1	9.719	7554737	412959	49.853	59.195
2	13.651	7599315	284667	50.147	40.805
Total		15154053	697626	100.000	100.000

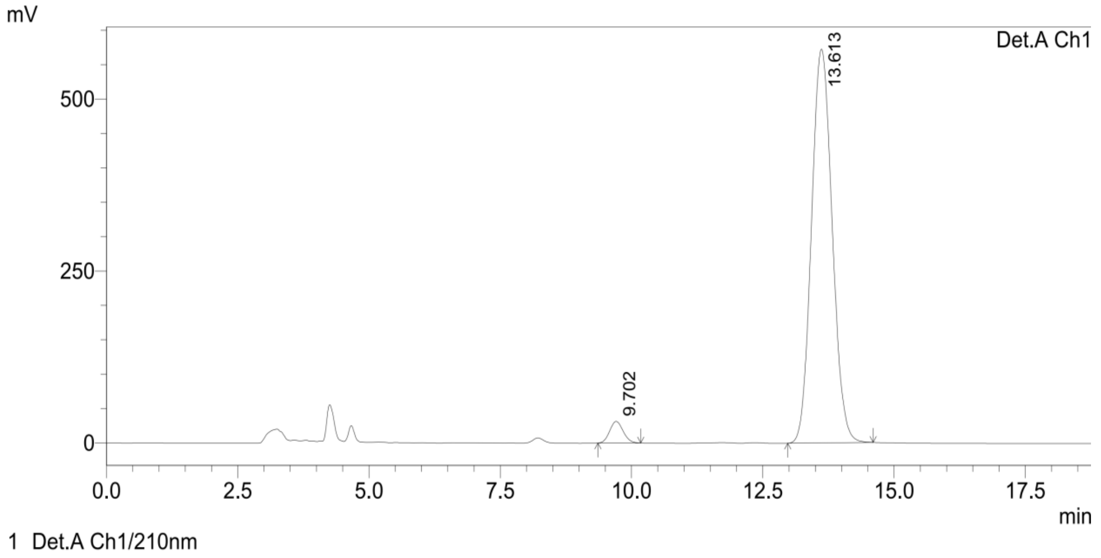

PeakTable

Detector A Ch1 210nm

Peak#	Ret. Time	Area	Height	Area %	Height %
1	9.702	560714	31406	3.455	5.200
2	13.613	15666446	572531	96.545	94.800
Total		16227160	603936	100.000	100.000

HPLC spectra of product 3w

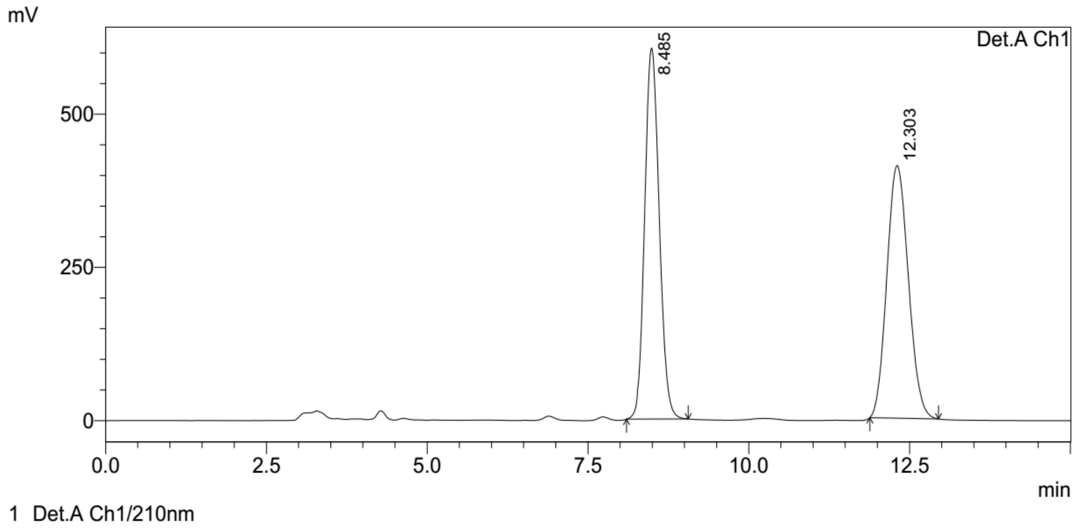

PeakTable

Detector A Ch1 210nm

Peak#	Ret. Time	Area	Height	Area %	Height %
1	8.485	9537754	605468	49.848	59.498
2	12.303	9596040	412162	50.152	40.502
Total		19133794	1017630	100.000	100.000

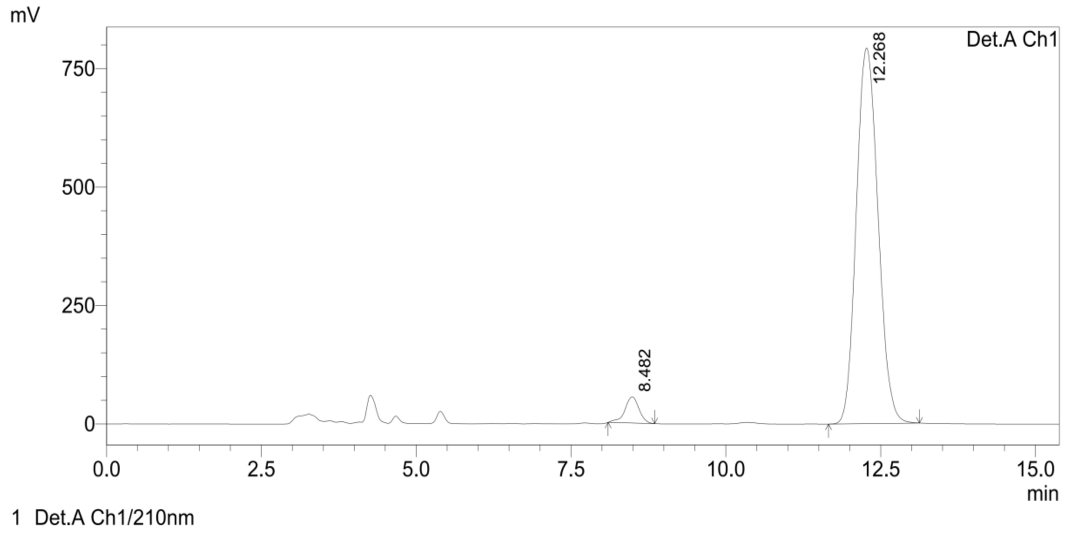

PeakTable

Detector A Ch1 210nm

Peak#	Ret. Time	Area	Height	Area %	Height %
1	8.482	870205	55328	4.301	6.524
2	12.268	19362041	792725	95.699	93.476
Total		20232247	848053	100.000	100.000

HPLC spectra of product 3x

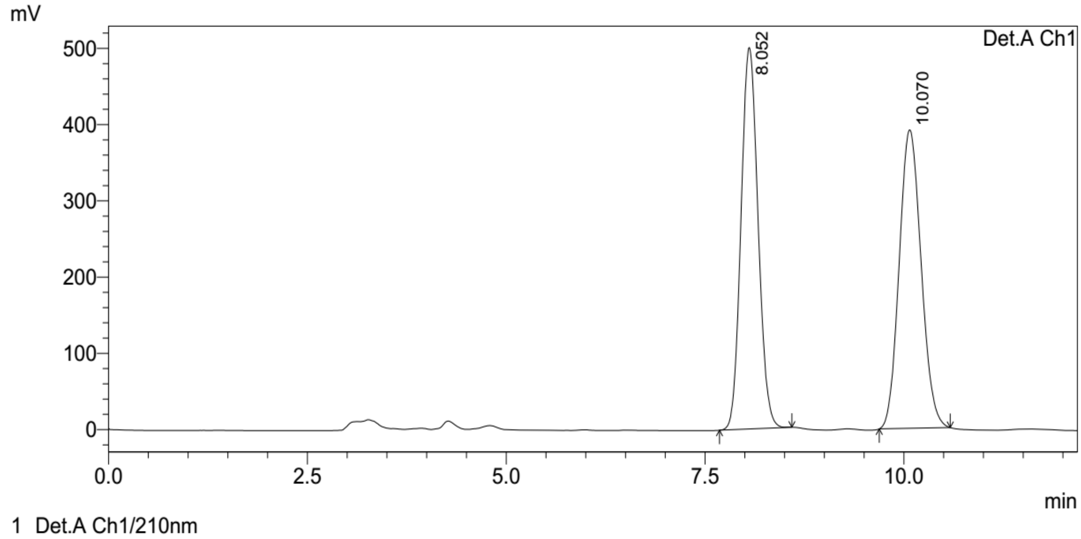

PeakTable

Detector A Ch1 210nm

Peak#	Ret. Time	Area	Height	Area %	Height %
1	8.052	7443515	500419	49.806	56.105
2	10.070	7501601	391514	50.194	43.895
Total		14945116	891933	100.000	100.000

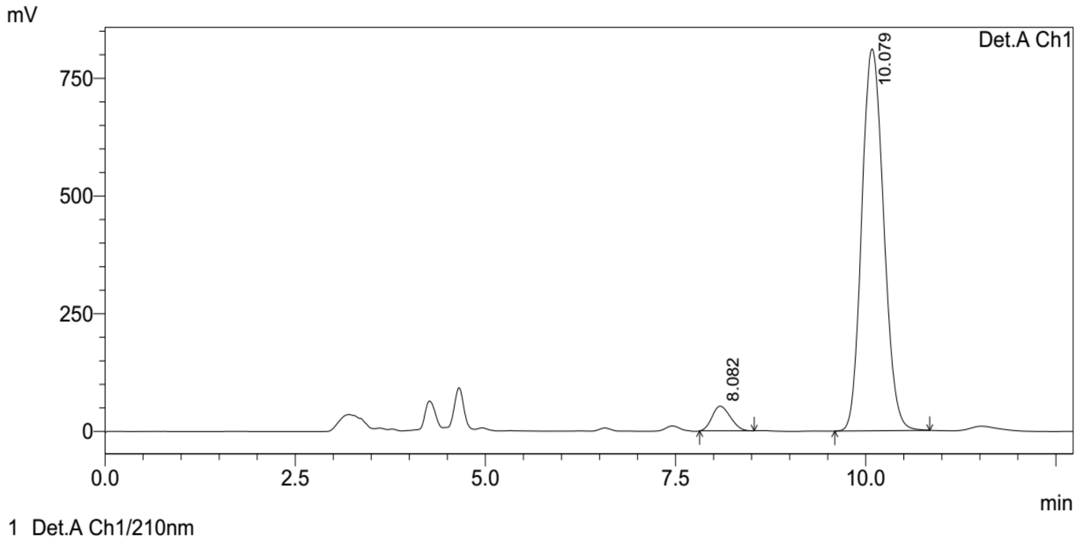

PeakTable

Detector A Ch1 210nm

Peak#	Ret. Time	Area	Height	Area %	Height %
1	8.082	873794	52382	5.097	6.065
2	10.079	16267889	811296	94.903	93.935
Total		17141682	863678	100.000	100.000

HPLC spectra of product 3y

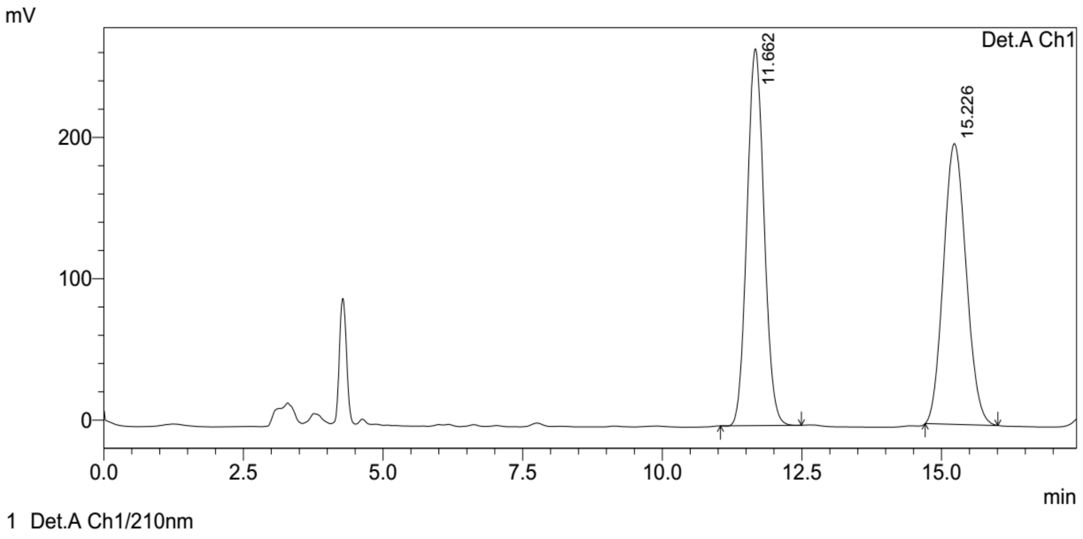

PeakTable

Detector A Ch1 210nm

Peak#	Ret. Time	Area	Height	Area %	Height %
1	11.662	5677958	266672	50.294	57.317
2	15.226	5611490	198589	49.706	42.683
Total		11289448	465261	100.000	100.000

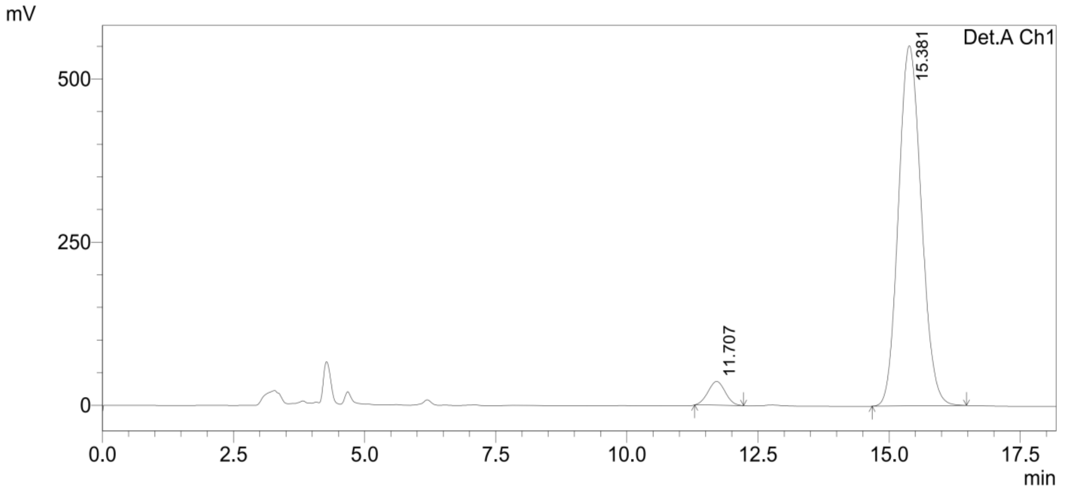

1 Det.A Ch1/210nm

PeakTable

Detector A Ch1 210nm

Peak#	Ret. Time	Area	Height	Area %	Height %
1	11.707	835653	36486	4.759	6.203
2	15.381	16724602	551756	95.241	93.797
Total		17560255	588242	100.000	100.000

HPLC spectra of product 3z

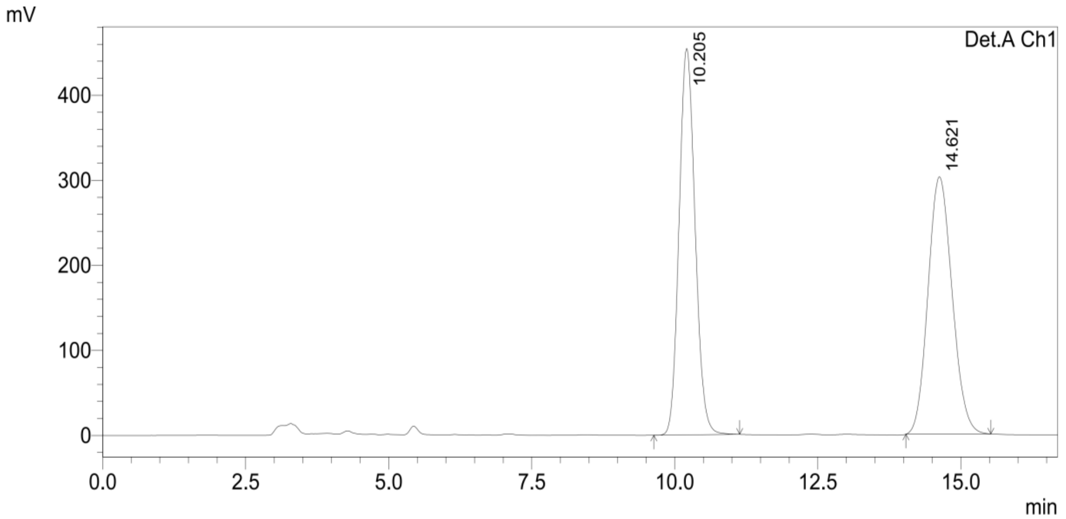

1 Det.A Ch1/210nm

PeakTable

Detector A Ch1 210nm

Peak#	Ret. Time	Area	Height	Area %	Height %
1	10.205	8857667	454380	49.952	60.034
2	14.621	8874662	302491	50.048	39.966
Total		17732329	756871	100.000	100.000

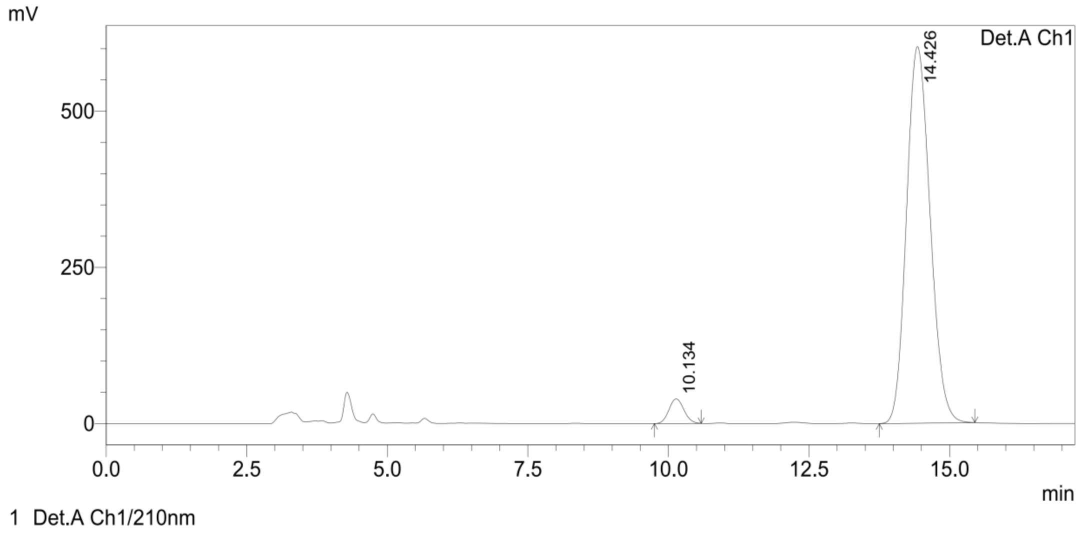

PeakTable

Detector A Ch1 210nm

Peak#	Ret. Time	Area	Height	Area %	Height %
1	10.134	732336	39522	3.992	6.153
2	14.426	17611489	602802	96.008	93.847
Total		18343826	642324	100.000	100.000

HPLC spectra of product 3a’

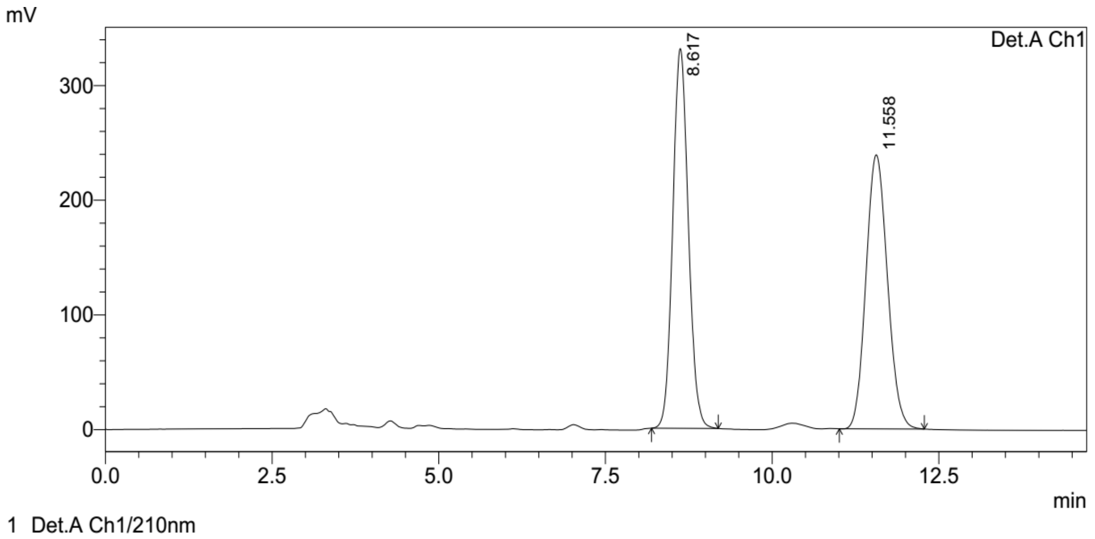

PeakTable

Detector A Ch1 210nm

Peak#	Ret. Time	Area	Height	Area %	Height %
1	8.617	5401692	331060	50.229	58.079
2	11.558	5352372	238954	49.771	41.921
Total		10754064	570015	100.000	100.000

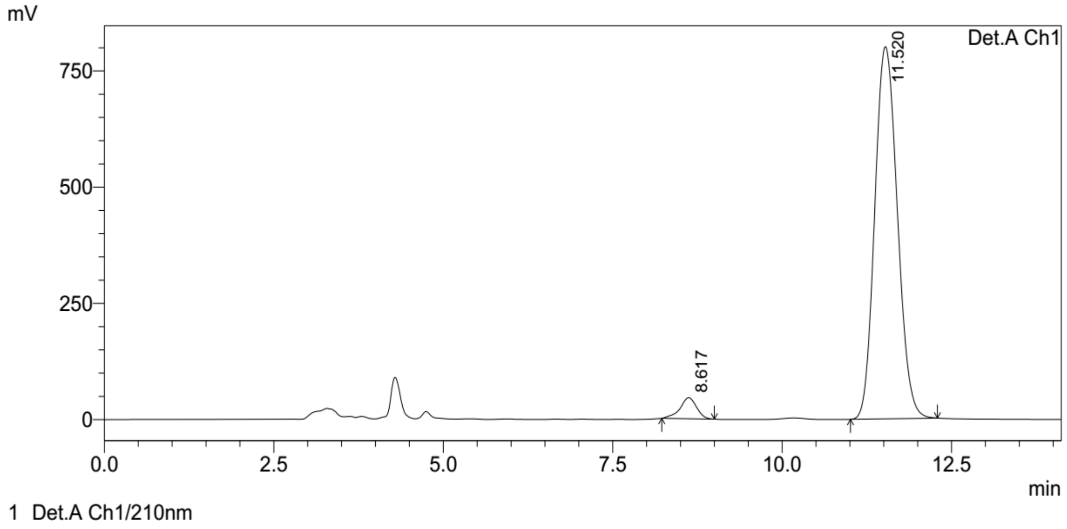

PeakTable

Detector A Ch1 210nm

Peak#	Ret. Time	Area	Height	Area %	Height %
1	8.617	770731	45123	3.980	5.334
2	11.520	18592362	800790	96.020	94.666
Total		19363093	845912	100.000	100.000

HPLC spectra of product 3b’

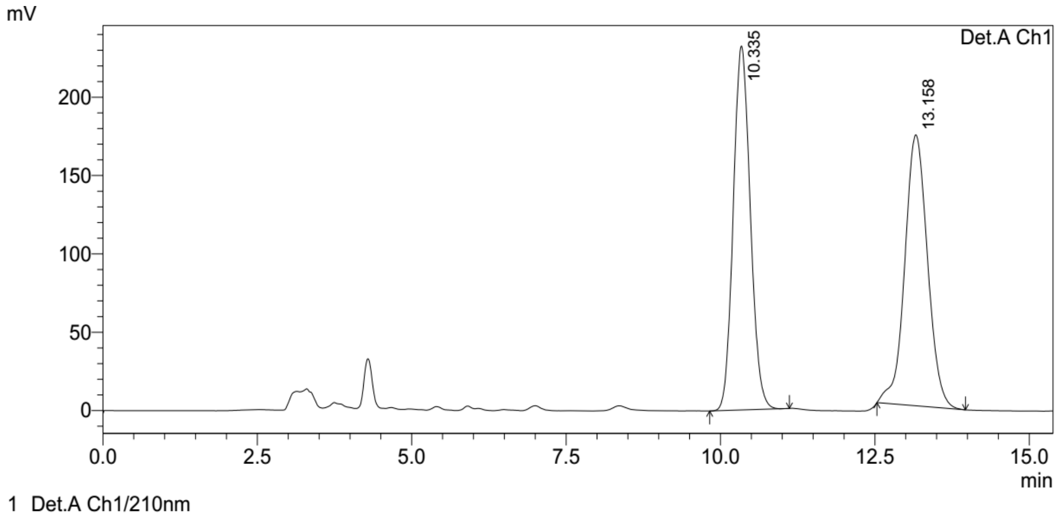

PeakTable

Detector A Ch1 210nm

Peak#	Ret. Time	Area	Height	Area %	Height %
1	10.335	4461960	232289	50.133	57.336
2	13.158	4438268	172847	49.867	42.664
Total		8900227	405136	100.000	100.000

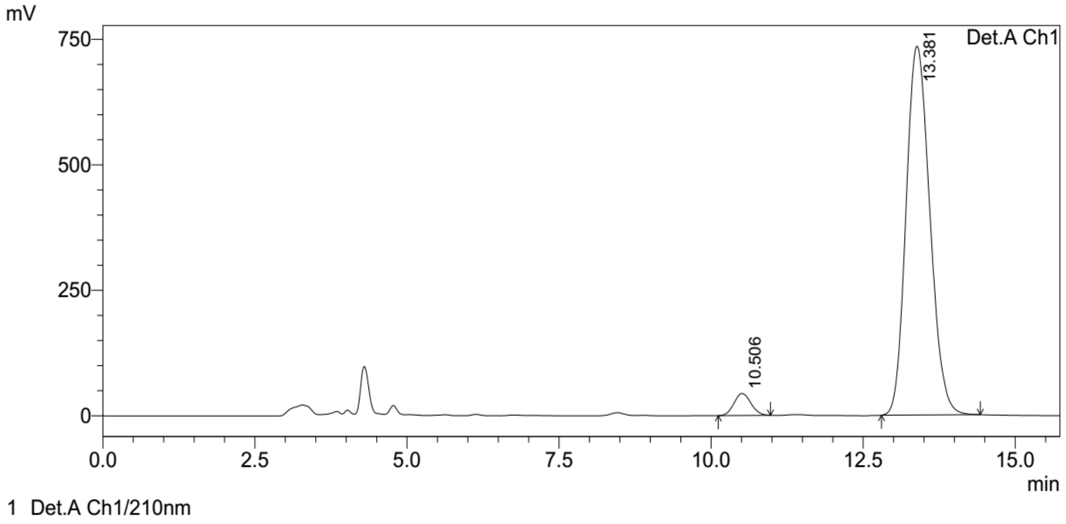

PeakTable

Detector A Ch1 210nm

Peak#	Ret. Time	Area	Height	Area %	Height %
1	10.506	851330	43935	4.084	5.641
2	13.381	19996032	734862	95.916	94.359
Total		20847362	778797	100.000	100.000

HPLC spectra of product 3c'

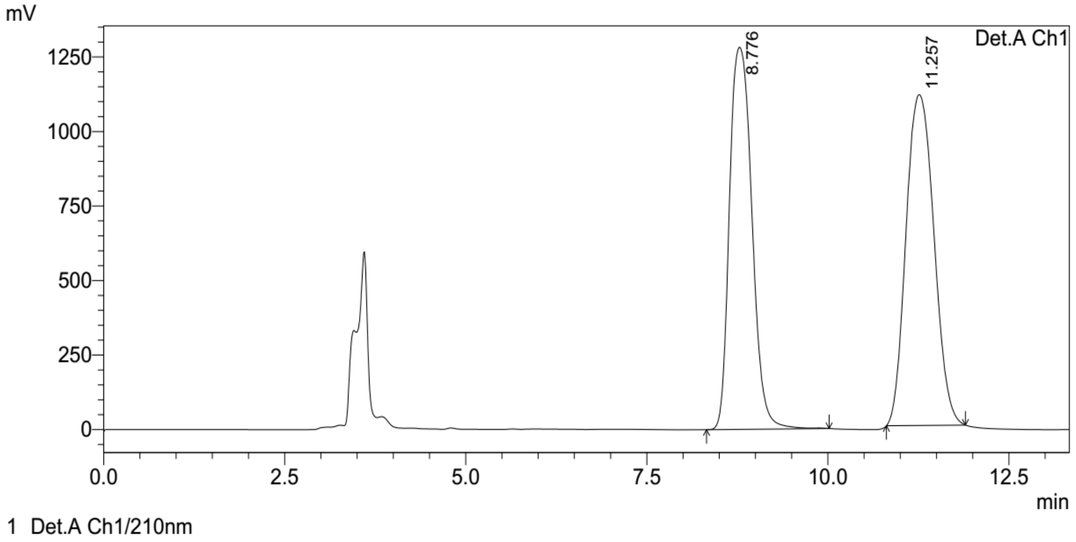

PeakTable

Detector A Ch1 210nm

Peak#	Ret. Time	Area	Height	Area %	Height %
1	8.776	27664752	1281882	48.145	53.596
2	11.257	29797103	1109863	51.855	46.404
Total		57461854	2391745	100.000	100.000

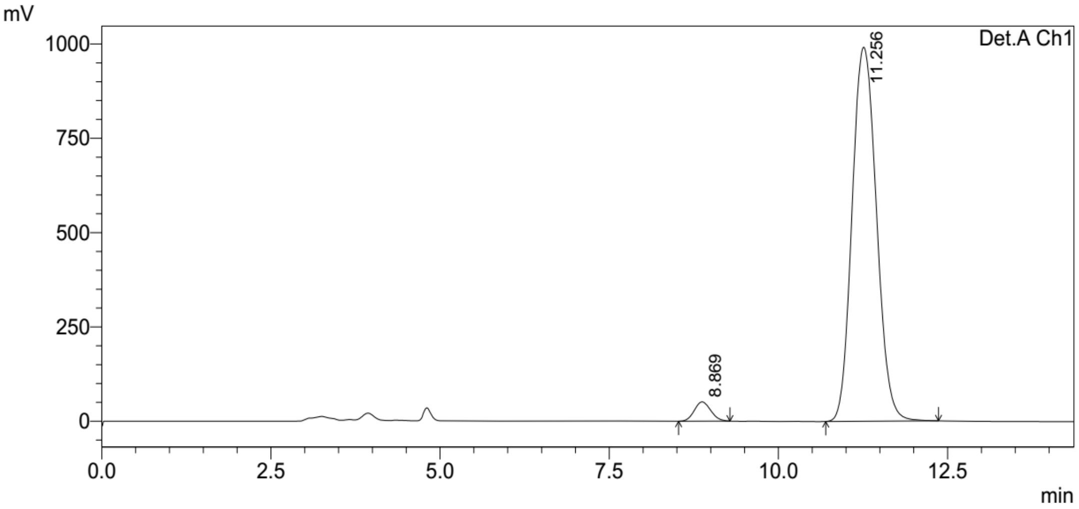

1 Det.A Ch1/210nm

PeakTable

Detector A Ch1 210nm

Peak#	Ret. Time	Area	Height	Area %	Height %
1	8.869	867458	51276	3.369	4.916
2	11.256	24882114	991674	96.631	95.084
Total		25749572	1042950	100.000	100.000

HPLC spectra of product 3d'

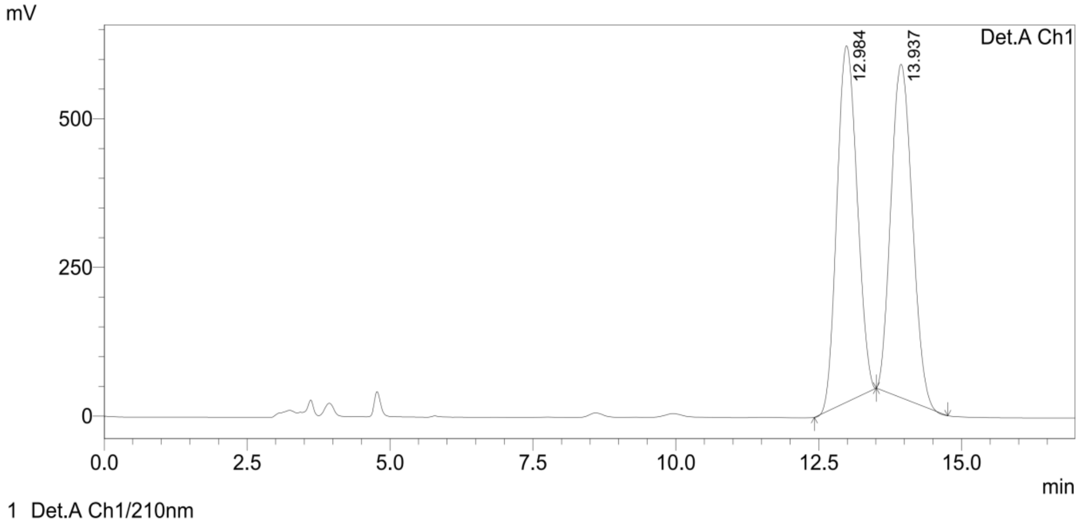

1 Det.A Ch1/210nm

PeakTable

Detector A Ch1 210nm

Peak#	Ret. Time	Area	Height	Area %	Height %
1	12.984	14727886	599674	50.250	51.679
2	13.937	14581260	560705	49.750	48.321
Total		29309146	1160379	100.000	100.000

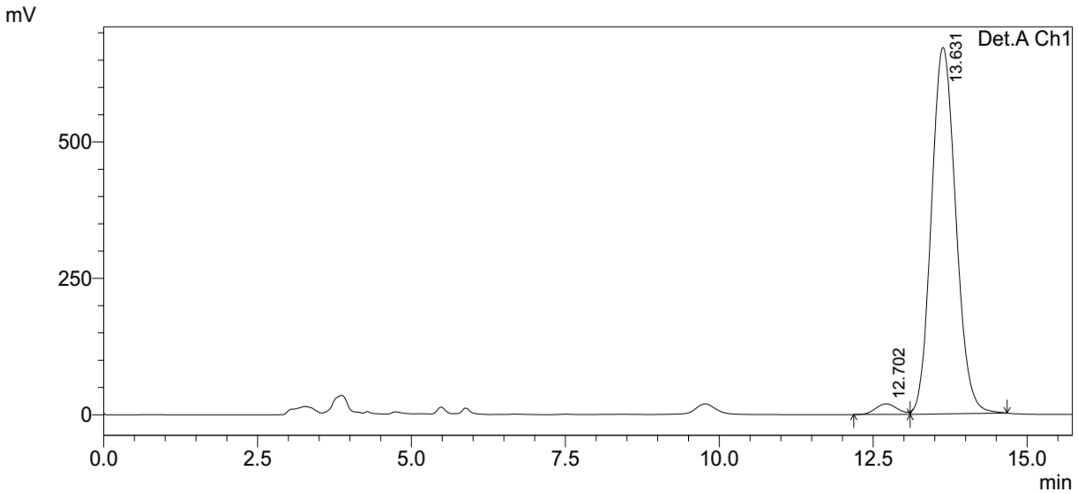

1 Det.A Ch1/210nm

PeakTable

Detector A Ch1 210nm

Peak#	Ret. Time	Area	Height	Area %	Height %
1	12.702	475113	19382	2.500	2.803
2	13.631	18526346	672016	97.500	97.197
Total		19001459	691397	100.000	100.000

HPLC spectra of product 3e'

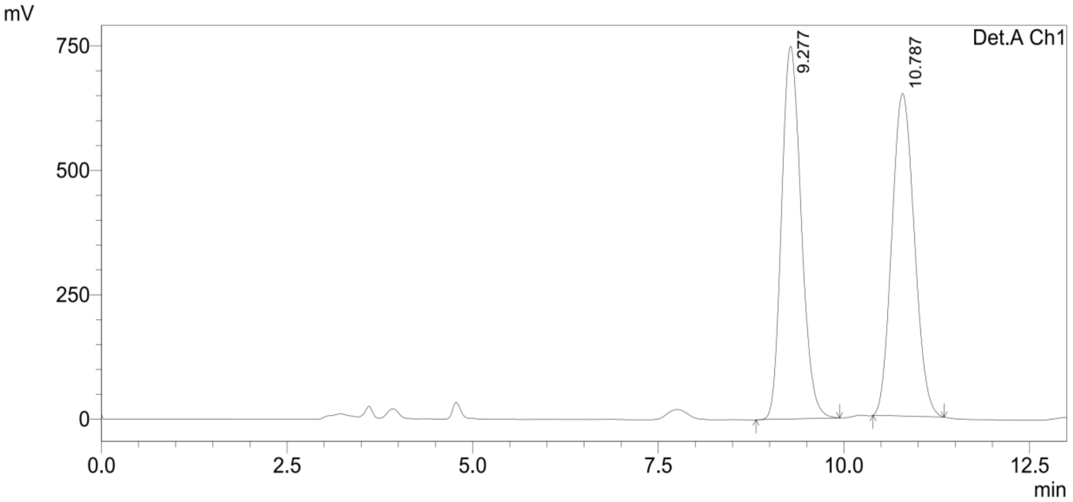

1 Det.A Ch1/210nm

PeakTable

Detector A Ch1 210nm

Peak#	Ret. Time	Area	Height	Area %	Height %
1	9.277	13739684	748923	50.123	53.606
2	10.787	13672385	648154	49.877	46.394
Total		27412069	1397077	100.000	100.000

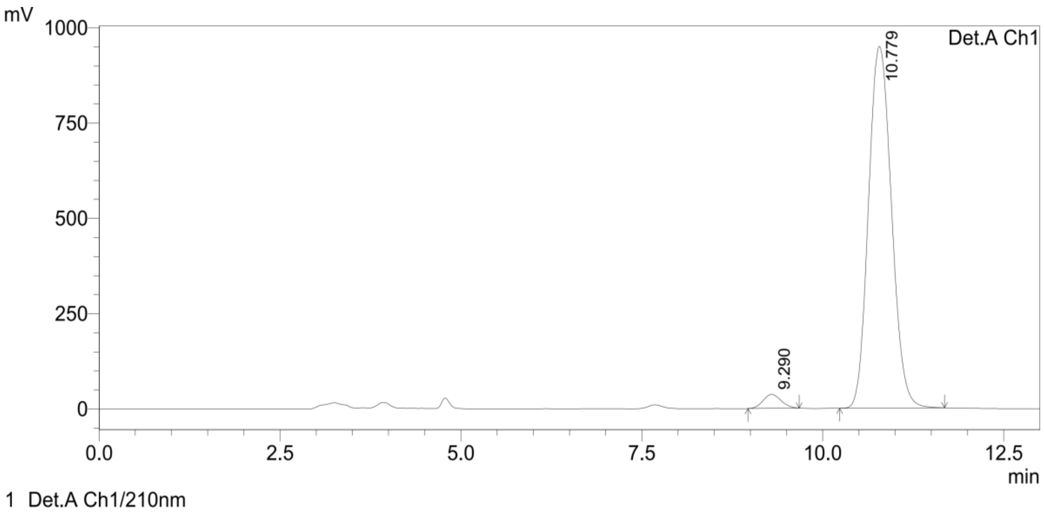

PeakTable

Detector A Ch1 210nm

Peak#	Ret. Time	Area	Height	Area %	Height %
1	9.290	632150	36559	2.922	3.710
2	10.779	21003368	948945	97.078	96.290
Total		21635518	985503	100.000	100.000

HPLC spectra of product 4a’. (Synthetic transformations)

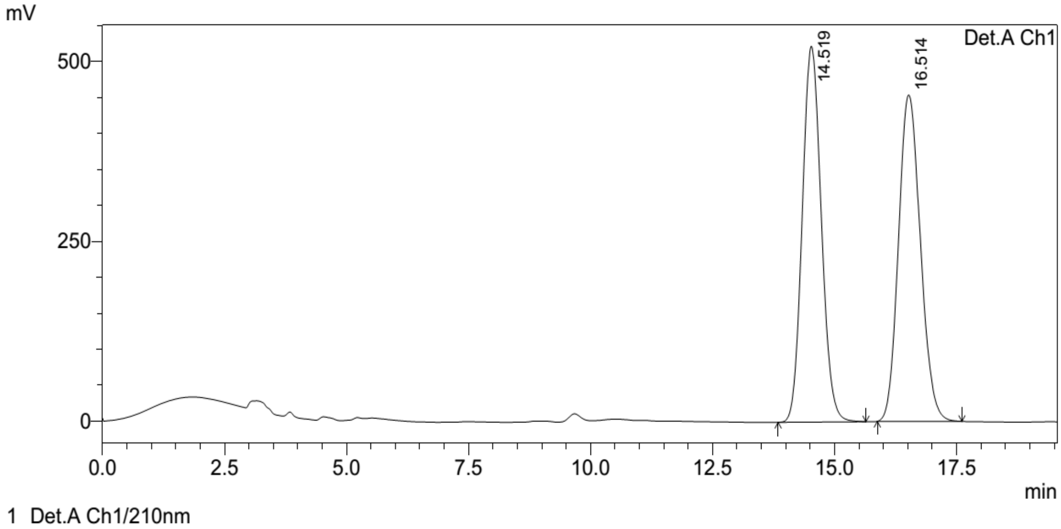

PeakTable

Detector A Ch1 210nm

Peak#	Ret. Time	Area	Height	Area %	Height %
1	14.519	14152763	522517	49.996	53.509
2	16.514	14155080	453978	50.004	46.491
Total		28307843	976495	100.000	100.000

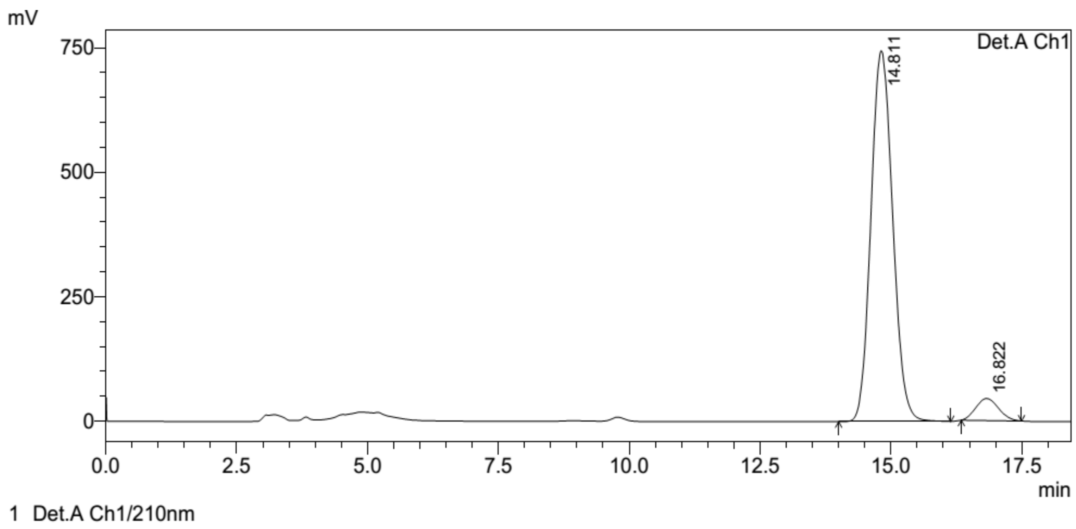

PeakTable

Detector A Ch1 210nm

Peak#	Ret. Time	Area	Height	Area %	Height %
1	14.811	21525356	745016	94.129	94.362
2	16.822	1342572	44513	5.871	5.638
Total		22867928	789529	100.000	100.000

HPLC spectra of product 4a. (Synthetic transformations)

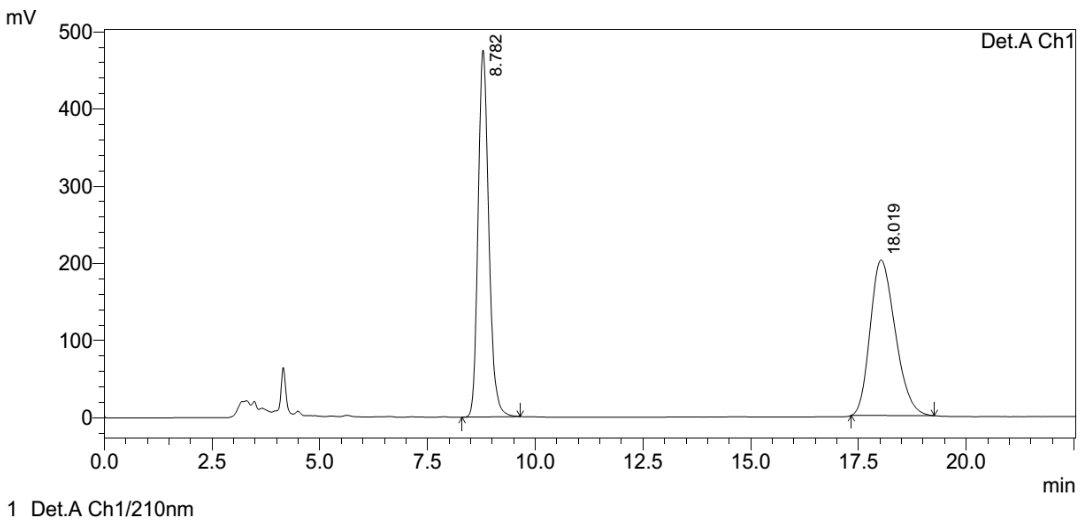

PeakTable

Detector A Ch1 210nm

Peak#	Ret. Time	Area	Height	Area %	Height %
1	8.782	8194008	475457	50.114	70.232
2	18.019	8156609	201519	49.886	29.768
Total		16350618	676976	100.000	100.000

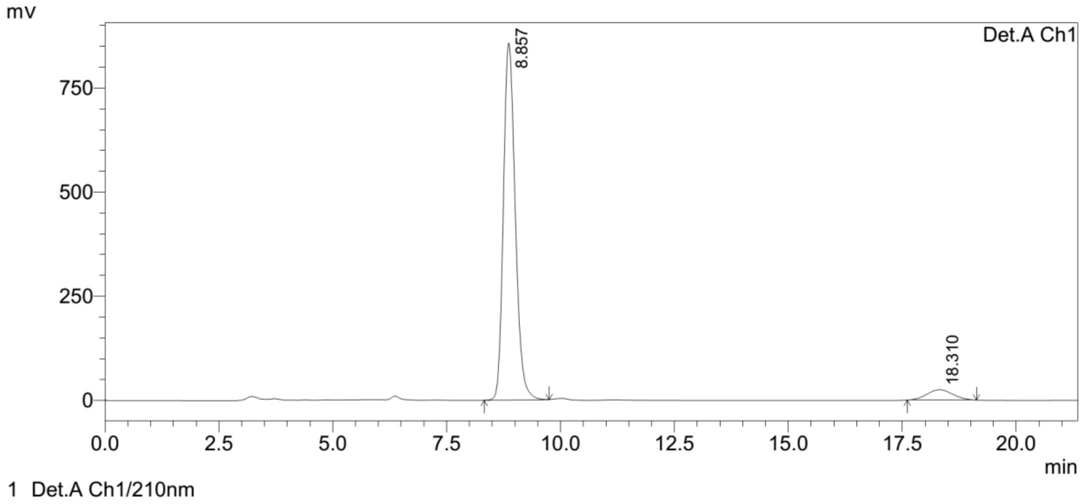

PeakTable

Detector A Ch1 210nm

Peak#	Ret. Time	Area	Height	Area %	Height %
1	8.857	15506465	856532	94.039	97.173
2	18.310	982882	24922	5.961	2.827
Total		16489347	881454	100.000	100.000

HPLC spectra of product 4b. (Synthetic transformations)

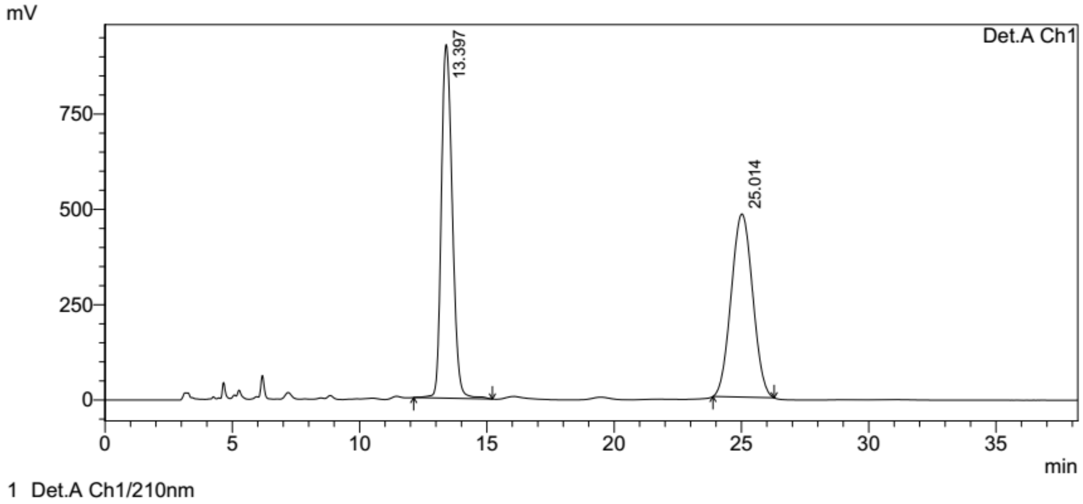

PeakTable

Detector A Ch1 210nm

Peak#	Ret. Time	Area	Height	Area %	Height %
1	13.397	27811702	927355	49.710	65.885
2	25.014	28136463	480177	50.290	34.115
Total		55948165	1407532	100.000	100.000

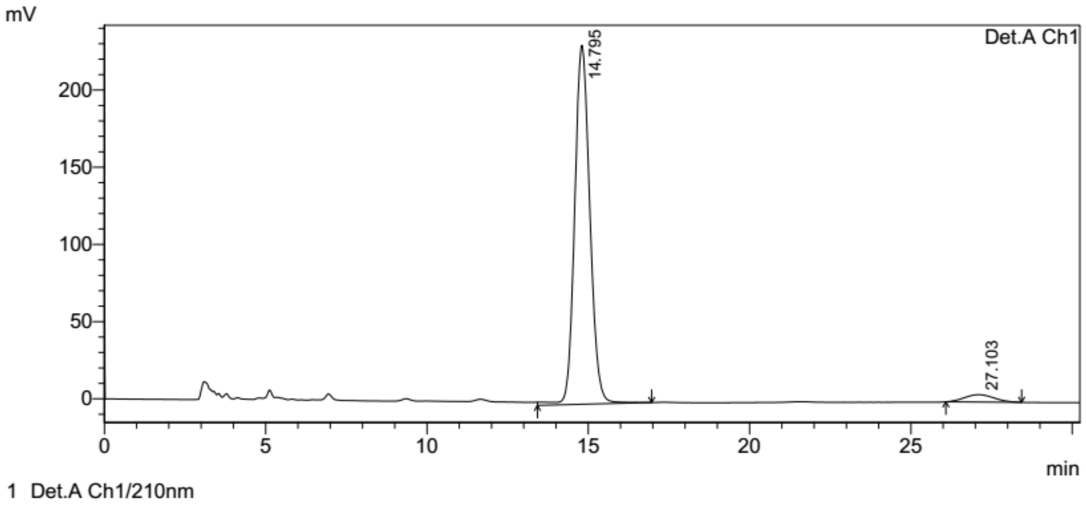

PeakTable

Detector A Ch1 210nm

Peak#	Ret. Time	Area	Height	Area %	Height %
1	14.795	7643231	232574	96.138	97.978
2	27.103	307028	4801	3.862	2.022
Total		7950259	237375	100.000	100.000

HPLC spectra of product 5a. (Synthetic transformations)

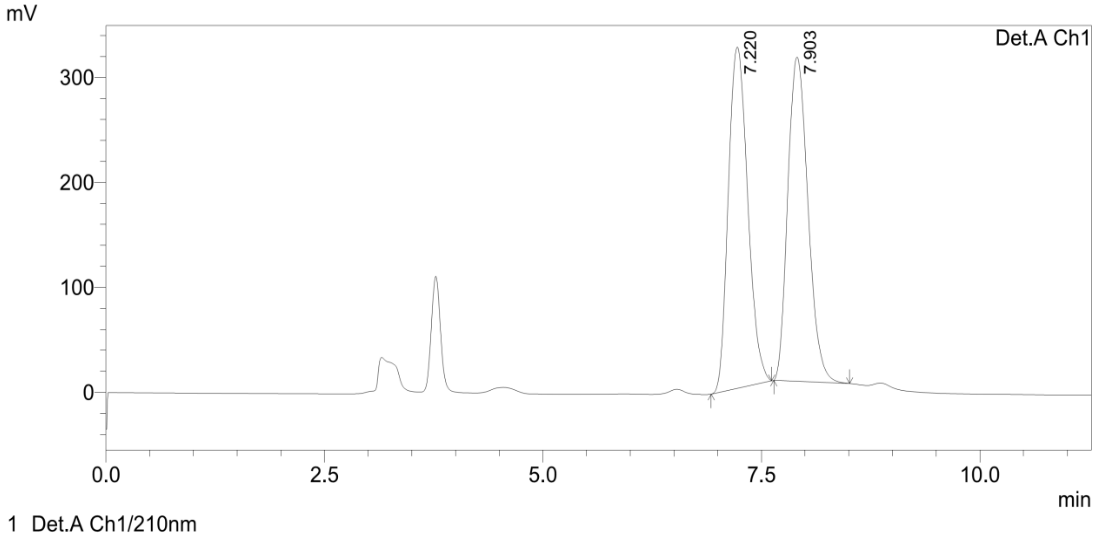

PeakTable

Detector A Ch1 210nm

Peak#	Ret. Time	Area	Height	Area %	Height %
1	7.220	5035805	325379	50.316	51.296
2	7.903	4972522	308932	49.684	48.704
Total		10008327	634312	100.000	100.000

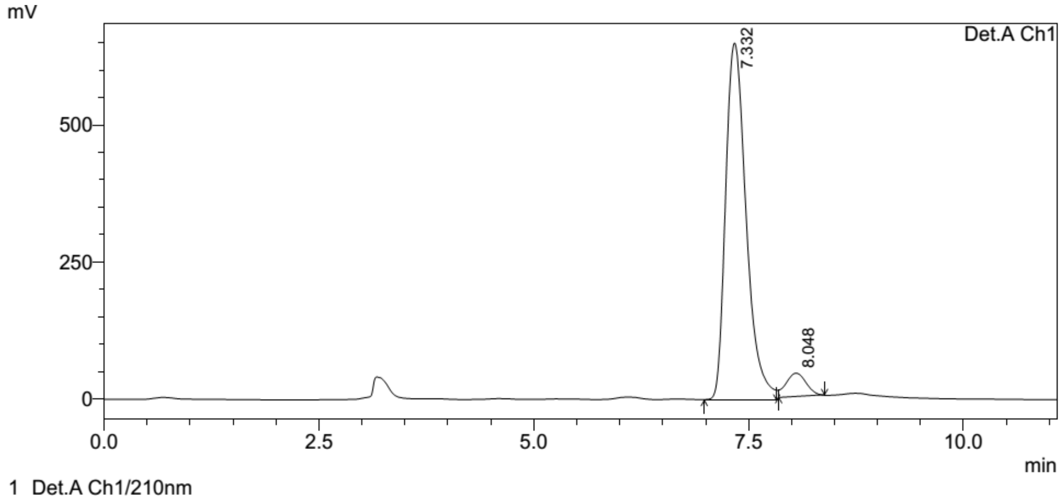

PeakTable

Detector A Ch1 210nm

Peak#	Ret. Time	Area	Height	Area %	Height %
1	7.332	10853202	651162	94.081	93.856
2	8.048	682782	42625	5.919	6.144
Total		11535983	693787	100.000	100.000

HPLC spectra of product 7a. (Synthetic transformations)

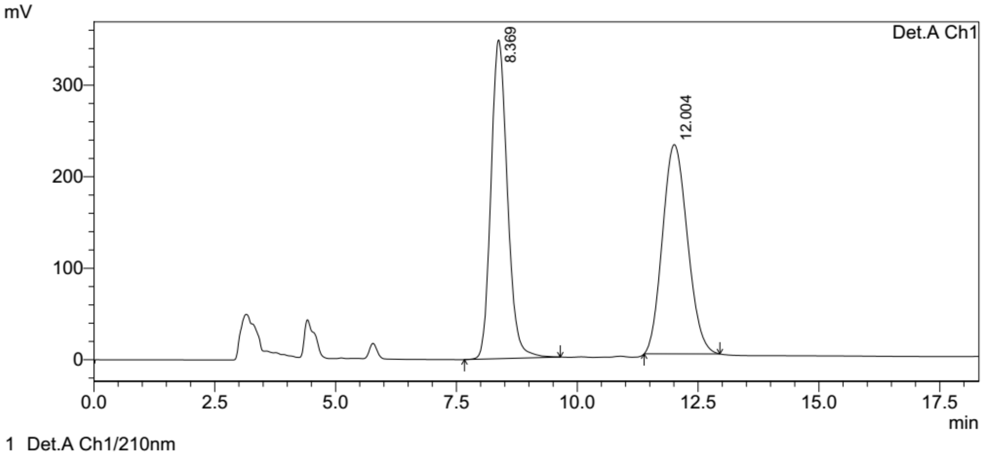

PeakTable

Detector A Ch1 210nm

Peak#	Ret. Time	Area	Height	Area %	Height %
1	8.369	8238769	348360	50.052	60.367
2	12.004	8221493	228709	49.948	39.633
Total		16460261	577069	100.000	100.000

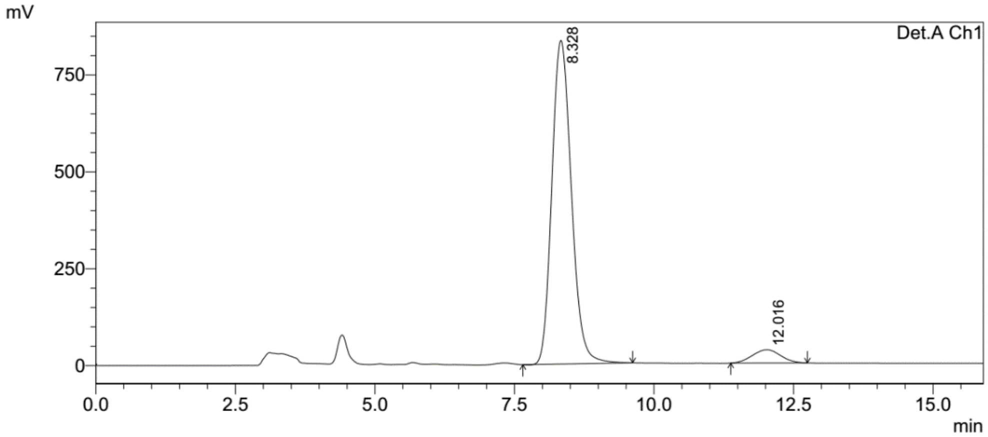

1 Det.A Ch1/210nm

PeakTable

Detector A Ch1 210nm

Peak#	Ret. Time	Area	Height	Area %	Height %
1	8.328	20307640	835393	94.388	96.060
2	12.016	1207532	34261	5.612	3.940
Total		21515172	869654	100.000	100.000

HPLC spectra of product 7b. (Synthetic transformations)

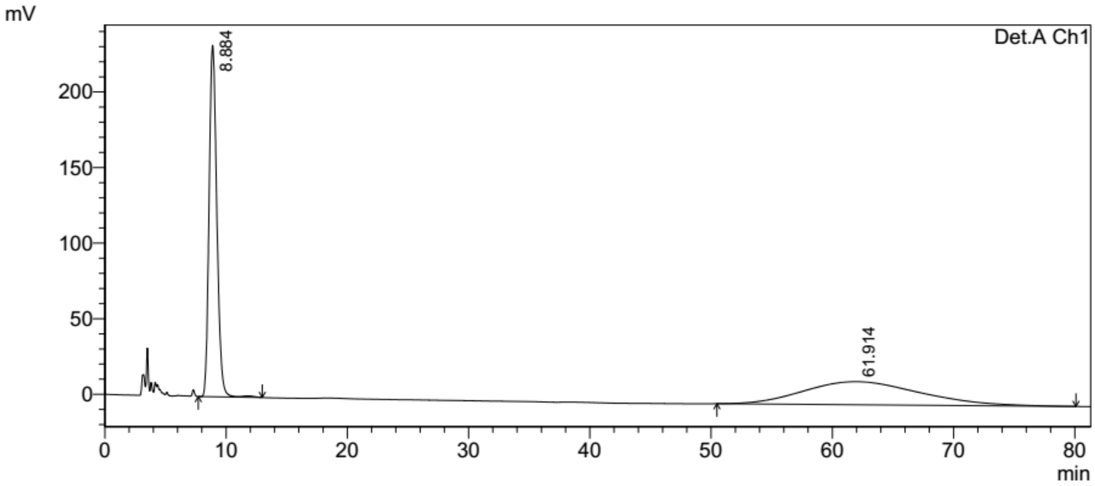

1 Det.A Ch1/210nm

PeakTable

Detector A Ch1 210nm

Peak#	Ret. Time	Area	Height	Area %	Height %
1	8.884	10329428	232560	50.090	93.834
2	61.914	10292408	15283	49.910	6.166
Total		20621837	247842	100.000	100.000

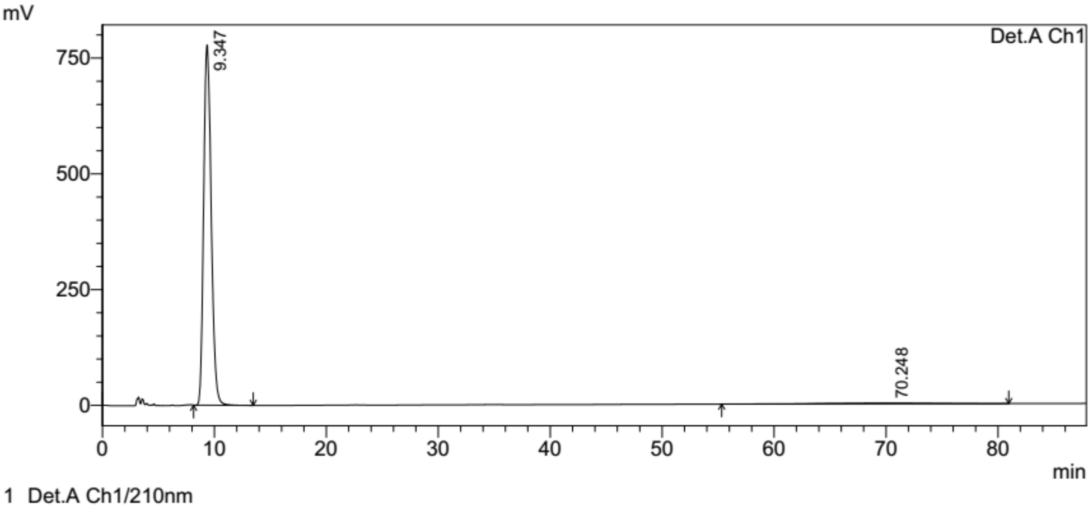

PeakTable

Detector A Ch1 210nm

Peak#	Ret. Time	Area	Height	Area %	Height %
1	9.347	36633046	777928	96.864	99.778
2	70.248	1185935	1729	3.136	0.222
Total		37818981	779657	100.000	100.000

HPLC spectra of product 11'. (Synthetic transformations)

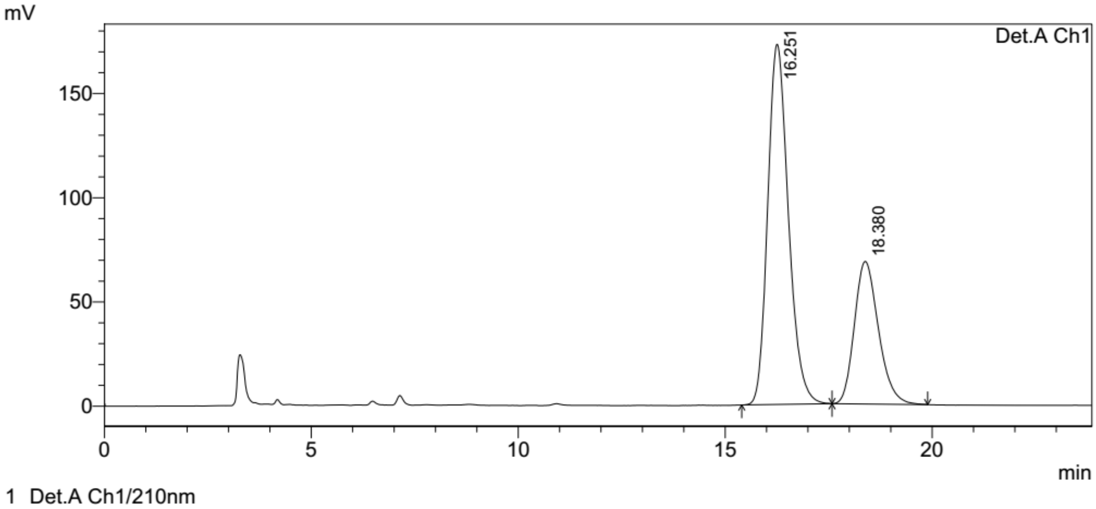

PeakTable

Detector A Ch1 210nm

Peak#	Ret. Time	Area	Height	Area %	Height %
1	16.251	6003184	172800	68.455	71.640
2	18.380	2766386	68406	31.545	28.360
Total		8769570	241206	100.000	100.000

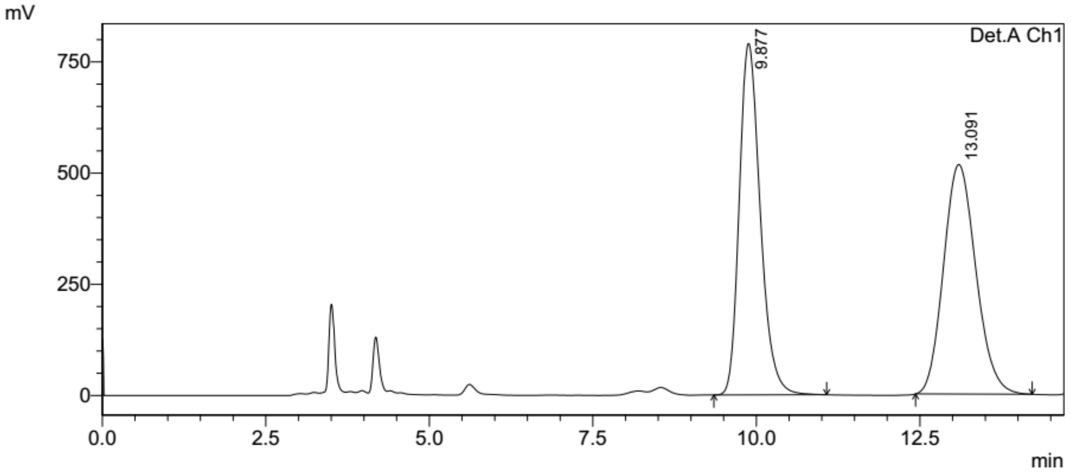

1 Det.A Ch1/210nm

PeakTable

Detector A Ch1 210nm

Peak#	Ret. Time	Area	Height	Area %	Height %
1	9.877	17619911	790083	49.832	60.488
2	13.091	17738548	516103	50.168	39.512
Total		35358459	1306186	100.000	100.000

HPLC spectra of product 12. (Synthetic transformations)

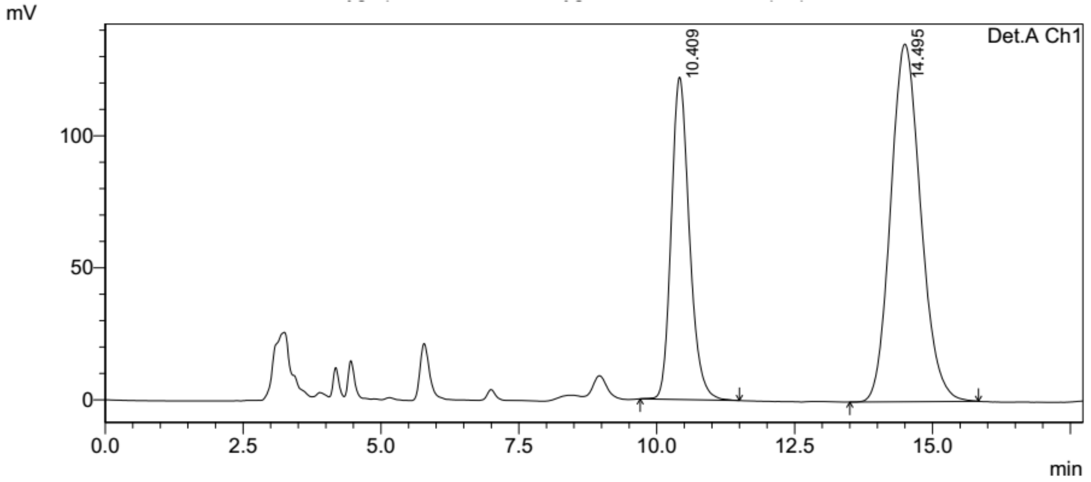

1 Det.A Ch1/210nm

PeakTable

Detector A Ch1 210nm

Peak#	Ret. Time	Area	Height	Area %	Height %
1	10.409	2793881	122122	35.191	47.401
2	14.495	5145408	135514	64.809	52.599
Total		7939289	257636	100.000	100.000

HPLC spectra of product 9. (Synthetic transformations)

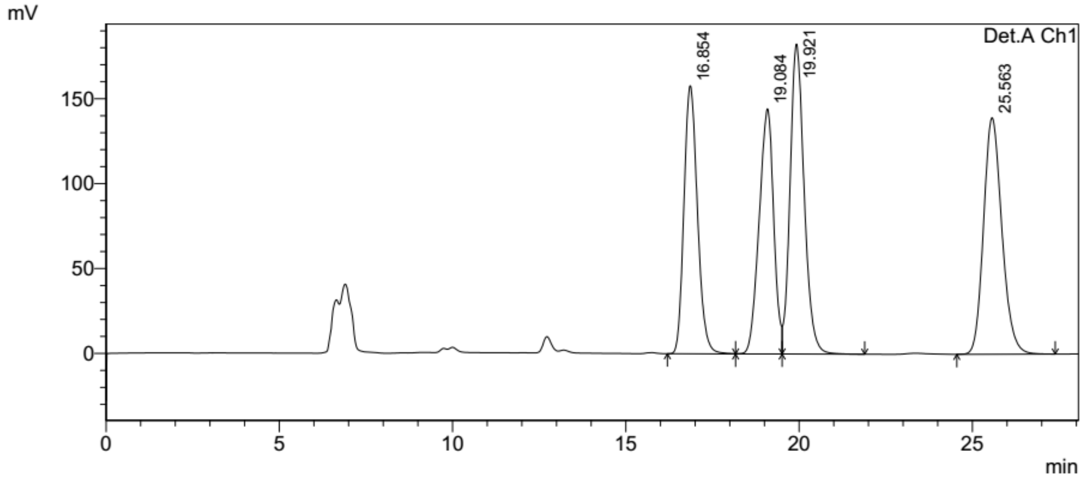

1 Det.A Ch1/210nm

PeakTable

Detector A Ch1 210nm

Peak#	Ret. Time	Area	Height	Area %	Height %
1	16.854	4276677	157879	22.760	25.292
2	19.084	4221241	144376	22.465	23.129
3	19.921	5169506	182634	27.511	29.258
4	25.563	5123167	139325	27.265	22.320
Total		18790591	624214	100.000	100.000

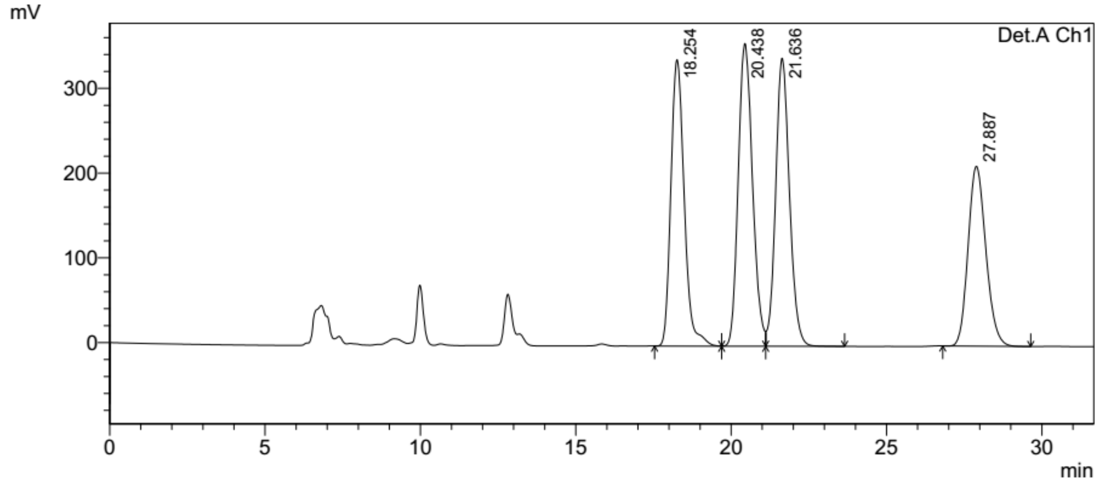

1 Det.A Ch1/210nm

PeakTable

Detector A Ch1 210nm

Peak#	Ret. Time	Area	Height	Area %	Height %
1	18.254	10107288	338051	24.947	27.097
2	20.438	11433920	357328	28.221	28.642
3	21.636	10540863	339996	26.017	27.253
4	27.887	8433402	212200	20.815	17.009
Total		40515473	1247575	100.000	100.000

HPLC spectra of product 10. (Synthetic transformations)

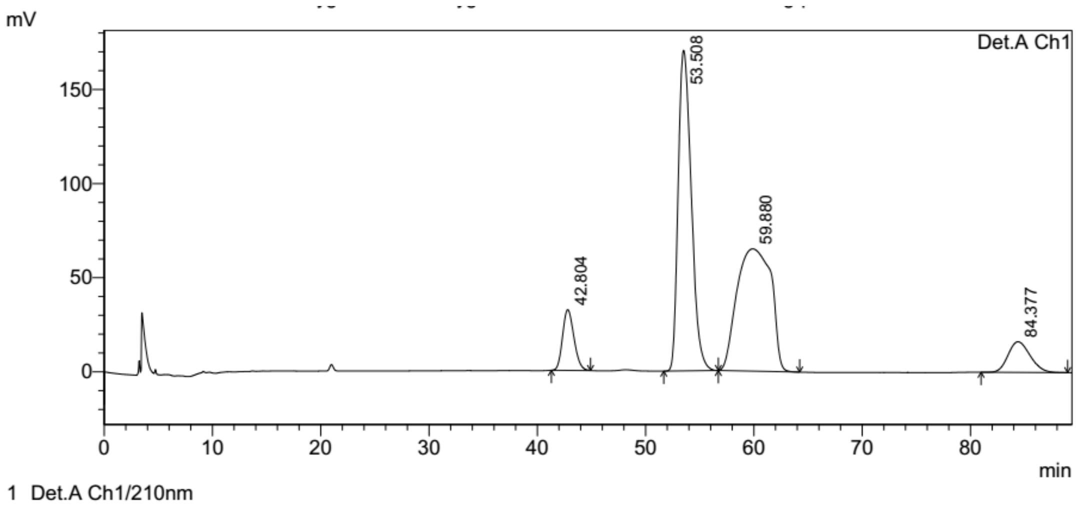

PeakTable

Detector A Ch1 210nm

Peak#	Ret. Time	Area	Height	Area %	Height %
1	42.804	2353148	32270	7.052	11.368
2	53.508	14356887	170338	43.027	60.006
3	59.880	14341513	64974	42.981	22.889
4	84.377	2315390	16286	6.939	5.737
Total		33366938	283868	100.000	100.000

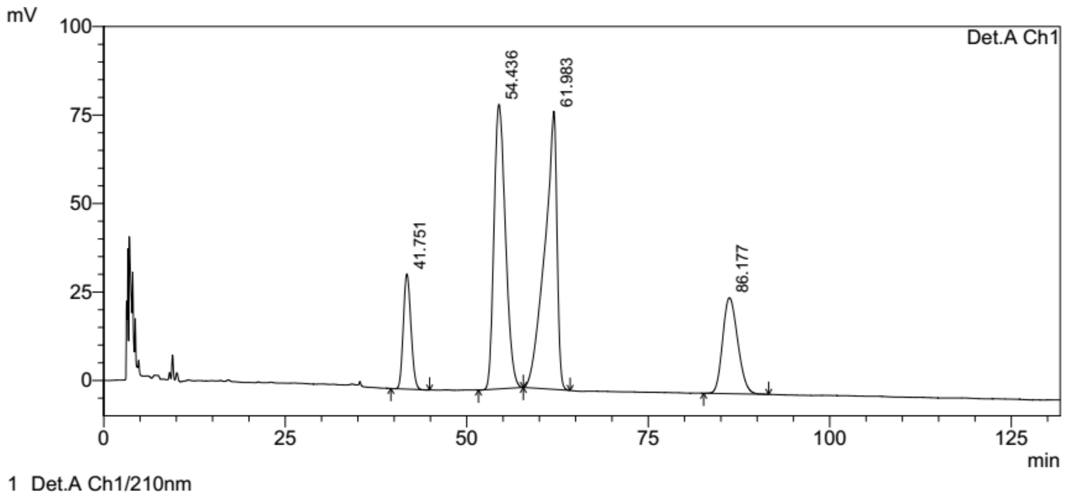

PeakTable

Detector A Ch1 210nm

Peak#	Ret. Time	Area	Height	Area %	Height %
1	41.751	2371657	32549	9.718	14.887
2	54.436	8836415	80377	36.208	36.762
3	61.983	9276155	78606	38.009	35.953
4	86.177	3920691	27106	16.065	12.398
Total		24404918	218638	100.000	100.000

HPLC spectra of product 5a-5f. (LFER analysis)

HPLC spectra of product 5a

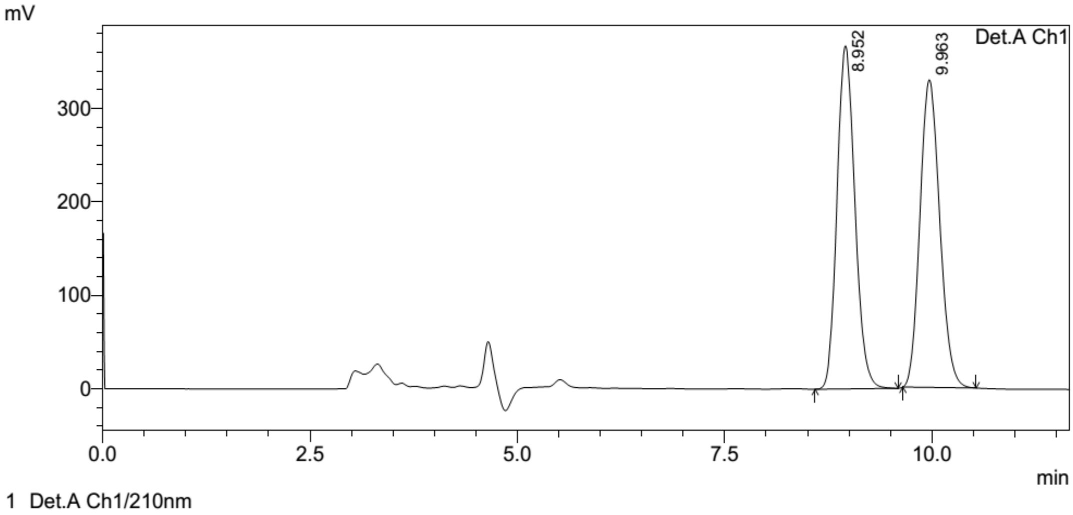

PeakTable

Detector A Ch1 210nm

Peak#	Ret. Time	Area	Height	Area %	Height %
1	8.952	5524525	367141	50.238	52.715
2	9.963	5472278	329322	49.762	47.285
Total		10996803	696464	100.000	100.000

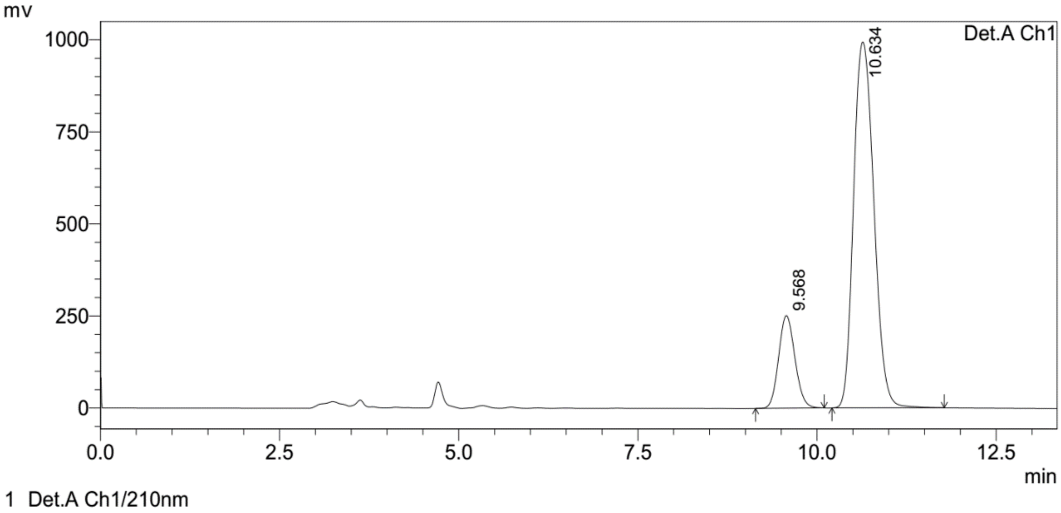

PeakTable

Detector A Ch1 210nm

Peak#	Ret. Time	Area	Height	Area %	Height %
1	9.568	3970749	251453	16.870	20.197
2	10.634	19565977	993546	83.130	79.803
Total		23536727	1244999	100.000	100.000

HPLC spectra of product 5b

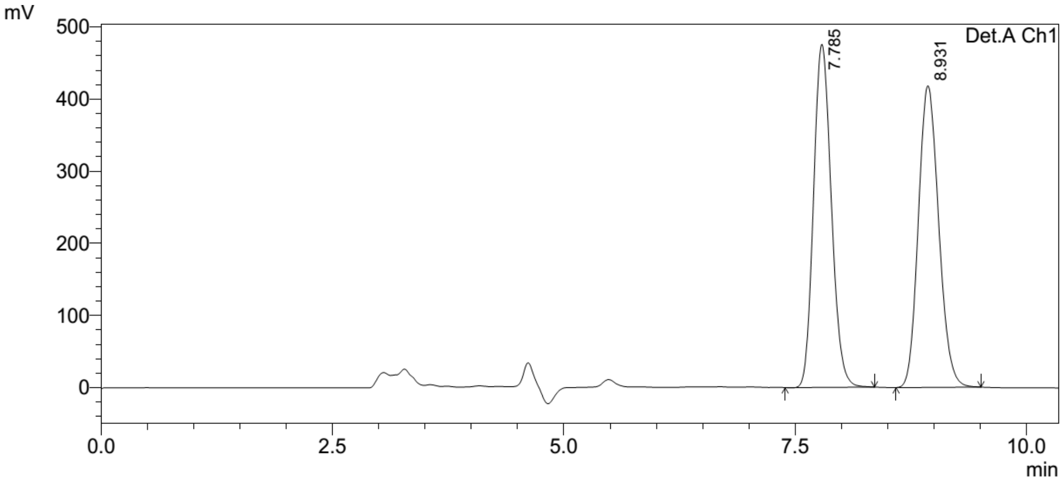

1 Det.A Ch1/210nm

PeakTable

Detector A Ch1 210nm

Peak#	Ret. Time	Area	Height	Area %	Height %
1	7.785	6347772	475713	49.741	53.209
2	8.931	6413778	418338	50.259	46.791
Total		12761550	894051	100.000	100.000

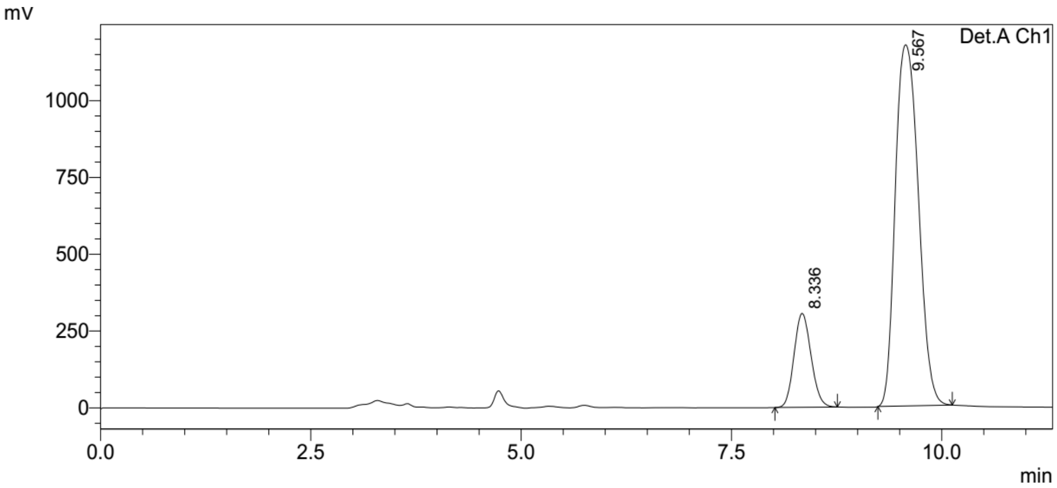

1 Det.A Ch1/210nm

PeakTable

Detector A Ch1 210nm

Peak#	Ret. Time	Area	Height	Area %	Height %
1	8.336	4298571	305904	16.065	20.647
2	9.567	22458604	1175713	83.935	79.353
Total		26757175	1481617	100.000	100.000

HPLC spectra of product 5c

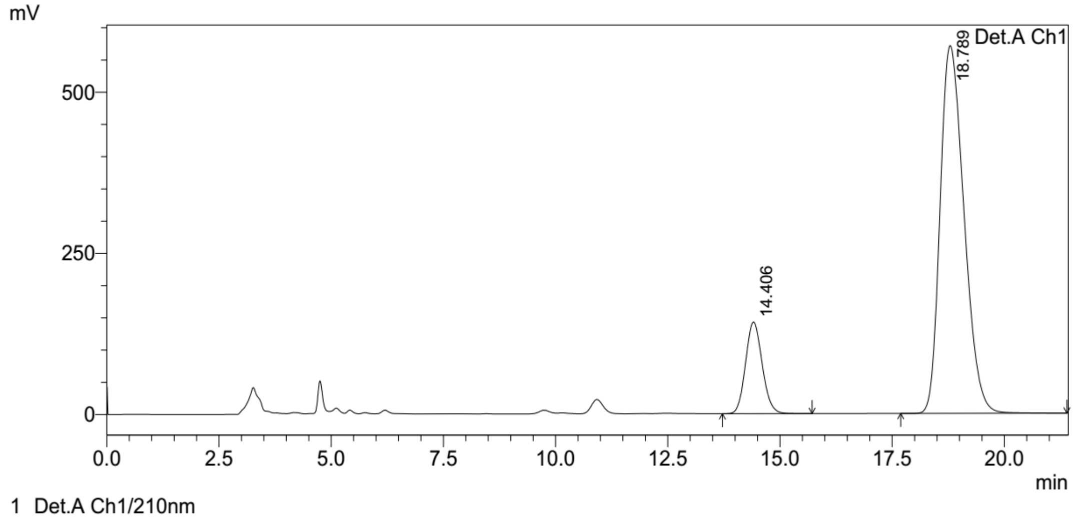

PeakTable

Detector A Ch1 210nm

Peak#	Ret. Time	Area	Height	Area %	Height %
1	14.406	3659633	142209	14.779	19.952
2	18.789	21103243	570543	85.221	80.048
Total		24762876	712752	100.000	100.000

HPLC spectra of product 5d

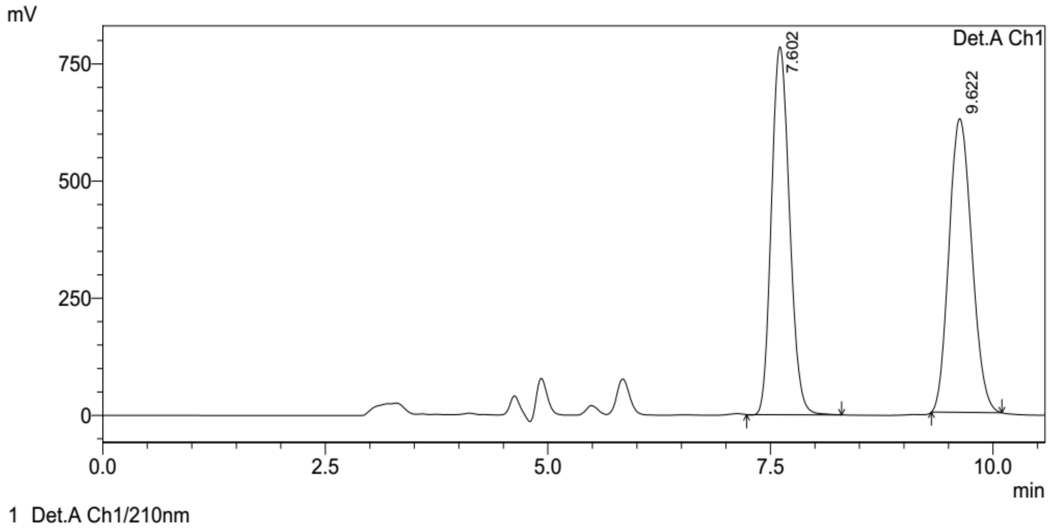

PeakTable

Detector A Ch1 210nm

Peak#	Ret. Time	Area	Height	Area %	Height %
1	7.602	11120895	785186	49.813	55.601
2	9.622	11204193	626988	50.187	44.399
Total		22325088	1412174	100.000	100.000

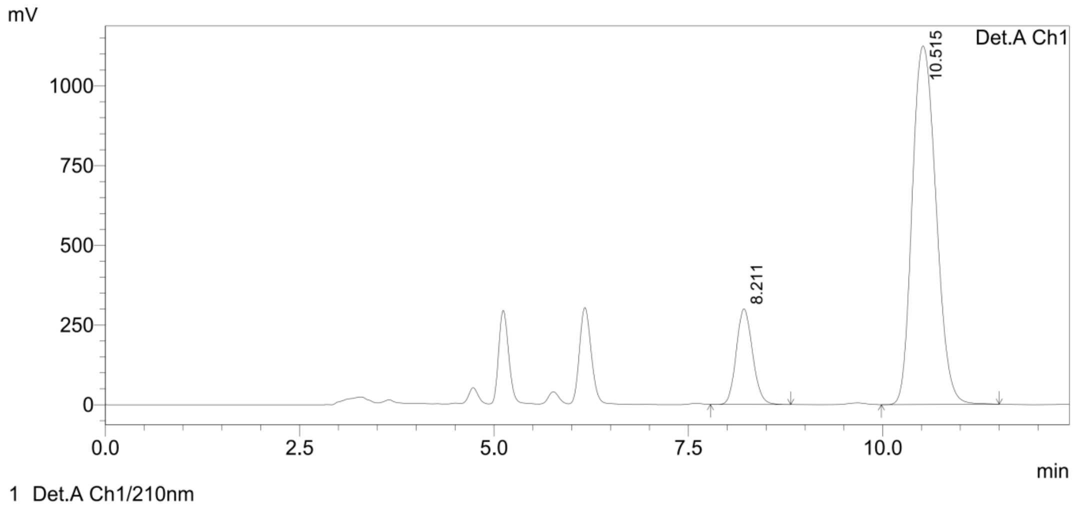

PeakTable

Detector A Ch1 210nm

Peak#	Ret. Time	Area	Height	Area %	Height %
1	8.211	4372825	299915	15.420	21.052
2	10.515	23984717	1124737	84.580	78.948
Total		28357542	1424651	100.000	100.000

HPLC spectra of product 5e

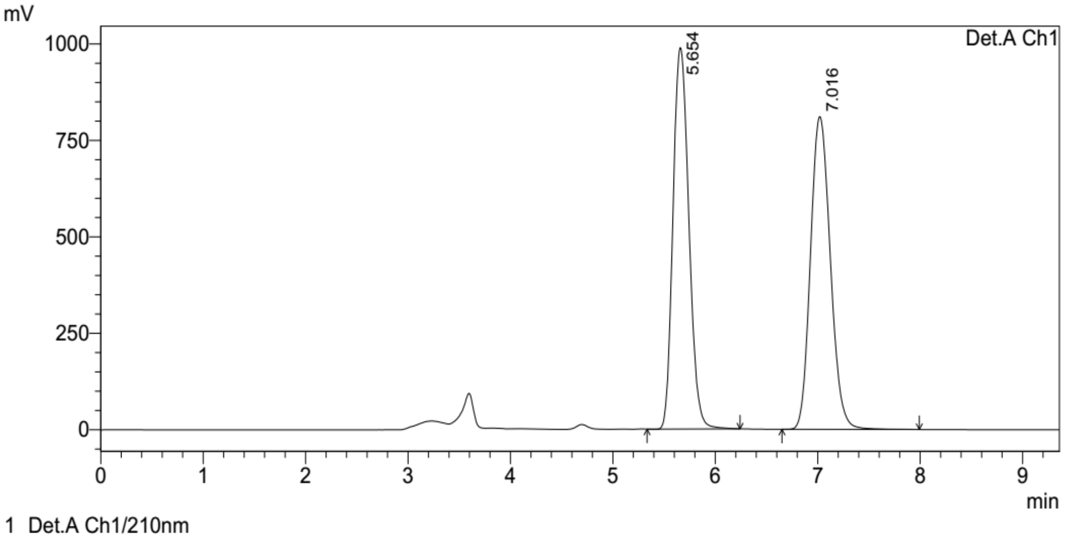

PeakTable

Detector A Ch1 210nm

Peak#	Ret. Time	Area	Height	Area %	Height %
1	5.654	10665687	988819	49.394	54.949
2	7.016	10927570	810718	50.606	45.051
Total		21593257	1799538	100.000	100.000

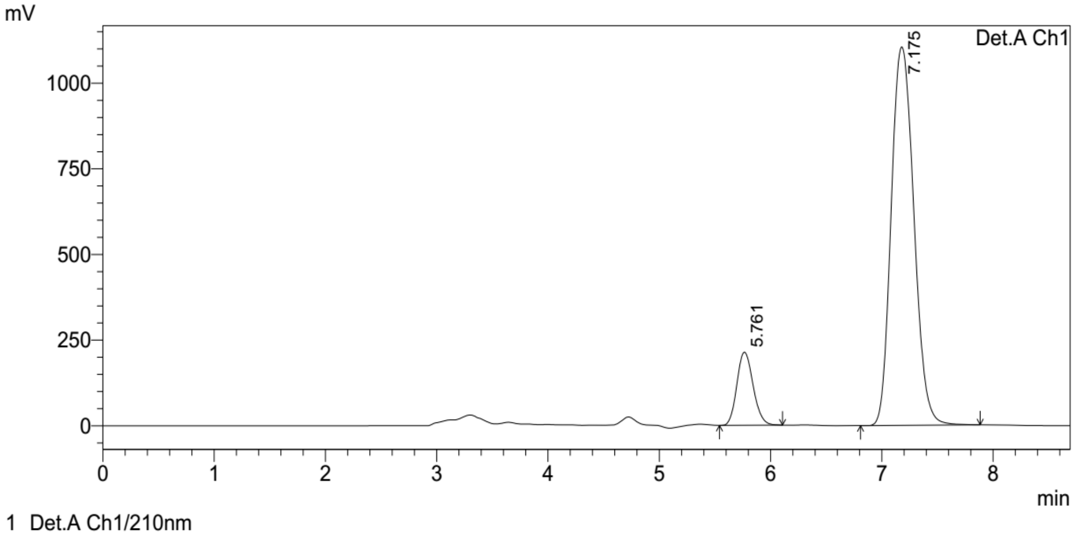

PeakTable

Detector A Ch1 210nm

Peak#	Ret. Time	Area	Height	Area %	Height %
1	5.761	2169419	213811	12.164	16.219
2	7.175	15665076	1104450	87.836	83.781
Total		17834495	1318261	100.000	100.000

HPLC spectra of product 5f

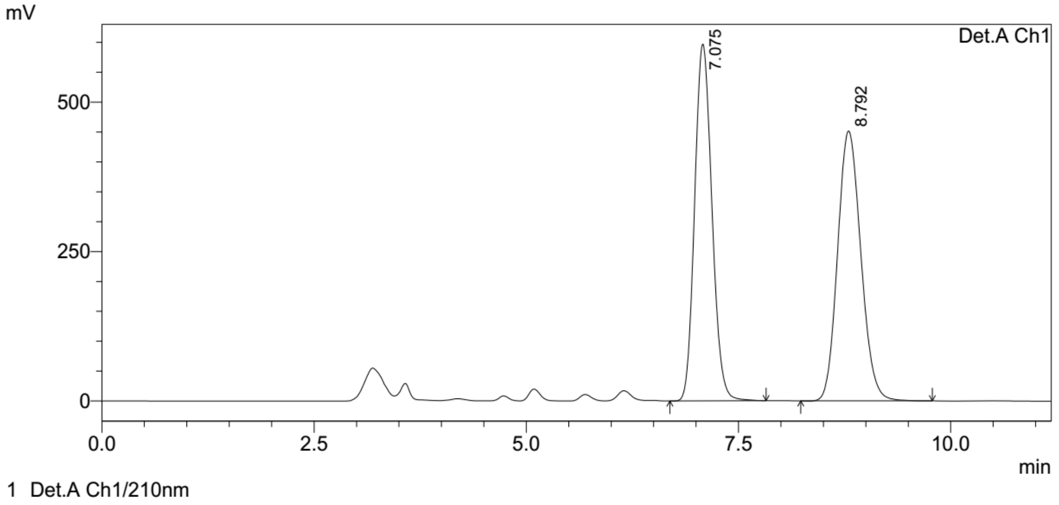

PeakTable

Detector A Ch1 210nm

Peak#	Ret. Time	Area	Height	Area %	Height %
1	7.075	8463083	596717	49.565	56.928
2	8.792	8611559	451488	50.435	43.072
Total		17074642	1048205	100.000	100.000

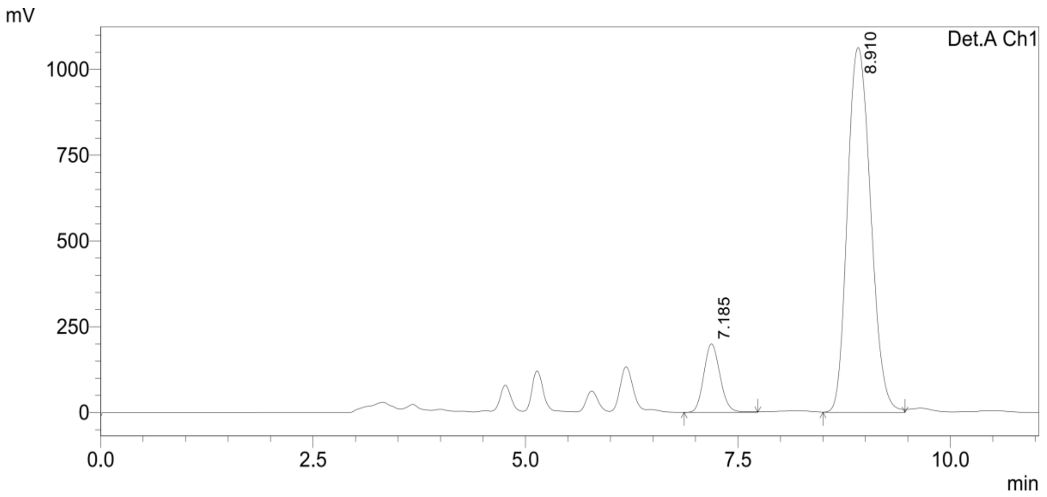

1 Det.A Ch1/210nm

PeakTable

Detector A Ch1 210nm

Peak#	Ret. Time	Area	Height	Area %	Height %
1	7.185	2658308	199586	11.571	15.800
2	8.910	20315156	1063613	88.429	84.200
Total		22973464	1263199	100.000	100.000
